# Sex-dimorphism in Cardiac Nutrigenomics: effect of *Trans *fat and/or Monosodium Glutamate consumption

**DOI:** 10.1186/1471-2164-12-555

**Published:** 2011-11-12

**Authors:** Kate S Collison, Marya Z Zaidi, Zakia Maqbool, Soad M Saleh, Angela Inglis, Nadine J Makhoul, Razan Bakheet, Mohammed Shoukri, Futwan A Al-Mohanna

**Affiliations:** 1 Cell Biology & Diabetes Research Unit, Department of Biological & Medical Research, King Faisal Specialist Hospital & Research Centre, PO BOX 3354, Riyadh 11211, Saudi Arabia; 2 Department of Biostatistics, Epidemiology and Scientific Computing, King Faisal Specialist Hospital & Research Centre, Saudi Arabia

## Abstract

**Background:**

A paucity of information on biological sex-specific differences in cardiac gene expression in response to diet has prompted this present nutrigenomics investigation.

Sexual dimorphism exists in the physiological and transcriptional response to diet, particularly in response to high-fat feeding. Consumption of *Trans*-fatty acids (TFA) has been linked to substantially increased risk of heart disease, in which sexual dimorphism is apparent, with males suffering a higher disease rate. Impairment of the cardiovascular system has been noted in animals exposed to Monosodium Glutamate (MSG) during the neonatal period, and sexual dimorphism in the growth axis of MSG-treated animals has previously been noted. Processed foods may contain both TFA and MSG.

**Methods:**

We examined physiological differences and changes in gene expression in response to TFA and/or MSG consumption compared to a control diet, in male and female C57BL/6J mice.

**Results:**

Heart and % body weight increases were greater in TFA-fed mice, who also exhibited dyslipidemia (P < 0.05). Hearts from MSG-fed females weighed less than males (P < 0.05). 2-factor ANOVA indicated that the TFA diet induced over twice as many cardiac differentially expressed genes (DEGs) in males compared to females (P < 0.001); and 4 times as many male DEGs were downregulated including *Gata4*, *Mef2d *and *Srebf2*. Enrichment of functional Gene Ontology (GO) categories were related to transcription, phosphorylation and anatomic structure (P < 0.01). A number of genes were upregulated in males and downregulated in females, including pro-apoptotic histone deacetylase-2 (HDAC2). Sexual dimorphism was also observed in cardiac transcription from MSG-fed animals, with both sexes upregulating approximately 100 DEGs exhibiting sex-specific differences in GO categories. A comparison of cardiac gene expression between all diet combinations together identified a subset of 111 DEGs significant only in males, 64 DEGs significant in females only, and 74 transcripts identified as differentially expressed in response to dietary manipulation in both sexes.

**Conclusion:**

Our model identified major changes in the cardiac transcriptional profile of TFA and/or MSG-fed mice compared to controls, which was reflected by significant differences in the physiological profile within the 4 diet groups. Identification of sexual dimorphism in cardiac transcription may provide the basis for sex-specific medicine in the future.

## Background

There is growing evidence that sexual dimorphism exists in the physiological response to diet and other environmental factors [1], including differences in insulin and leptin sensitivity [2,3], particularly in response to high fat diets [4]. The heart requires high levels of ATP in order to maintain contractility, and this essential energy requirement is usually met by the oxidation of fatty acids (FAs), which are the most important source of energy for the adult heart. FAs have also been shown to be involved in signal transduction and the regulation of gene transcription [5]; however excessive FA uptake may cause detrimental lipotoxicity, resulting in deregulated cardiac function [6]. Several key studies have recently shown that considerable sex-dimorphism exists in both rodents and humans [7]; and have established sex-specific pathways in cardiac transcription in response to both pressure overload [8] and dilated cardiomyopathy [9].However there is a relative scarcity of information concerning sex-specific effects of diet on cardiac gene expression.

Dietary intake of added fats and oils has increased by over 60% during the past 35 years [10], and use of hydrogenated oils and shortenings in order to prolong shelf-life has resulted in an increase in *trans *fatty acid (TFA) consumption, which now accounts for between 1.7 - 8% of the global dietary fat intake (0.6 - 3% of total energy intake) [11]. TFA consumption has been linked to the increased risk of obesity [11], inflammation [12], coronary heart disease (CHD) [13] and type 2 diabetes [14]. Unlike saturated fat, food nutrition labeling does not set a recommended intake limit for *Trans*-fat, however one meta-analysis study found that a 2% increase in energy intake from TFA was associated with a 23% increase in the incidence of CHD [15]. Concern over the deleterious effects of dietary *Trans-*fat has prompted several countries to regulate the industrial production [16]; and retrospective meta-analysis of observational controlled dietary trials and prospective cohort studies suggests that replacement of *Trans*-fat with nonhydrogenated oils would result in significant reduction of the incidence of CHD [17]. Gender differences in the prevalence of CHD are well documented [18], and many factors appear to be responsible including lipid profile [19] and genetic susceptibility [20].

Neonatal administration of high doses of the food flavor enhancer Monosodium Glutamate (MSG) has also been shown to result in obesity [21] together with stunted heart growth and hypoplasticity in rodents [22]. Furthermore, the cardiovascular system of these animals have been shown to respond deficiently to certain challenges, including an attenuated blood pressure response to the systemic injection of nitric oxide synthase inhibitors, angiotensin II and other vasoactive compounds [23]. Injections of high doses of MSG (4 g/Kg body weight) cause ablation of the Arcuate Nucleus via glutamate-induced degeneration of those areas of the immature brain which are insufficiently protected by a mature blood-brain barrier, including the hypothalamic area [24]. This suggested that the hypothalamus may play an important role in the growth of the heart. It is now apparent that maternal administration of MSG can penetrate the placental barrier and distribute to the embryonic tissues of the fetus. Oral administration of MSG and ^3^H-labeled Glutamate (^3^H- Glu) as a tracer to pregnant mice resulted in marked elevations of ^3^H- Glu in the placenta and in fetal brain, liver and kidney [25]. MSG consumption has increased globally in recent years, with recent estimations of the current average daily intake believed to be up to 10 g/day [26]. Despite the widespread consumption of this common food flavor enhancer, which often occurs in processed and packaged foods in combination with *Trans*-fat, very few studies have addressed the effect of dietary *Trans*-fat and/or MSG consumption on cardiac gene expression. Furthermore, although sexual dimorphism at the growth hormone axis has been demonstrated in MSG-treated rodents [27], little is known about sex-specific gene expression in response to exposure to MSG.

The aim of this work was to establish the effect of dietary TFA and/or MSG on cardiac gene expression in an *in vivo *animal model, and to examine sex-specific differences as well as commonalities of gene expression in response to diet. The amount of oral MSG used in this study reflects current consumption levels [26,28] and is 30 - 40 times less that the level previously reported to induce neuronal damage when injected neonatally [21,23]. Since it has previously been ascertained that MSG excitotoxicity occurs only when the blood brain barrier is vulnerable, for example neonatally [24,25], and because developmental programming of cardiac gene expression may be affected by maternal nutritional balance [29], we bred our study animals from females previously established on these diets for 3 weeks prior to mating, in accordance with our previous studies [30]. Exposure to these diets occurred throughout the study, in order to mimic as closely as possible the situation that occurs in humans. The results of our microarray analysis revealed considerable sexual dimorphism in response to the dietary interventions that we tested, with males appearing to exhibit more differential gene expression compared to females.

## Results

### Consumption of *Trans*-fat and/or MSG results in sex-specific changes in physiological characteristics

**Figure 1:**
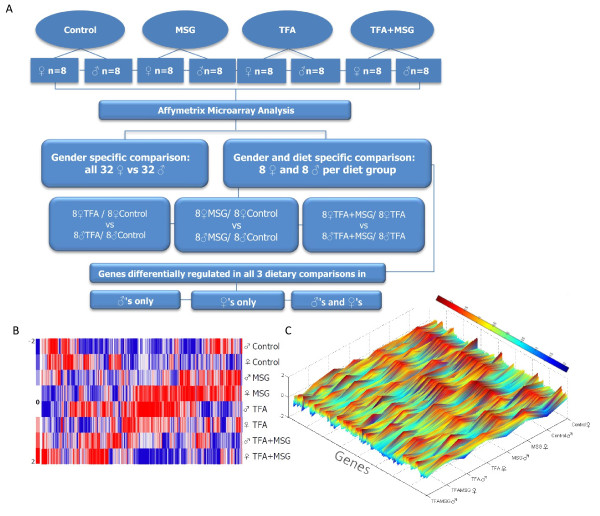
**Diet effects on cardiac gene expression**. **A: **Experimental design and statistical model. Eight samples per diet group were hybridized onto Affymetrix 1.0ST GeneChip arrays. **B**: 2D heatmap of 6791 sex-specific cardiac genes (P < 0.01). **C**: 3D heatmap of standardized expression values of DEGs with an FDR adjusted ANOVA p-value of <.01 for sex and diet.

Figure 1A shows our experimental design and microarray analysis. There were no significant differences in food and water intake between the 4 different diet groups or between the sexes (data not shown). Averaged daily MSG intake was 91.21 ± 4.63 and 99.04 ± 2.5 mg/Kg body weight in males and females respectively. *Trans*-fat (TFA) feeding resulted in elevated weight gain (6-32 weeks) and greater heart weights in both sexes (Table 1, P < 0.001), and females weighed less than males (P ≤ 0.05). Additionally, male mice in the MSG and *Trans*-fat diet groups had significantly greater weight gain and heart **: **body weight ratios compared to females (P < 0.001). Analysis of significant sex-specific differences in response to diet indicated that females in the MSG and the MSG+TFA diet group had smaller hearts and lower fasting HDL-C levels compared to males. Females in the TFA diet group also had higher fasting triglyceride and HOMA-IR values compared to female control diet mice. Conversely TFA feeding elevated male T-CHOL and HDL-C levels. There was no apparent difference in the histological appearance of cardiac tissues from the four diet groups (online Additional File 1).

**Table 1:** Body characteristics

	Control	MSG	TFA	TFA+MSG	P-Value
Body weight (g)					
*Male*	29.58 ± 2.49	27.89 ± 1.83	34.38 ± 4.05	34.04 ± 5.18	0.500
*Female*	22.04 ± 1.81*	20.95 ± 1.7**	24.45 ± 2.33*	22.95 ± 1.97*	0.638
Weight change (%)					
*Male*	58.69 ± 2.51^a^	45.41 ± 1.83^b^	79.72 ± 2.3^c^	75.55 ± 3.18^c^	<.0001
*Female*	46.15 ± 2.13^a^***	37.11 ± 1.84^a^**	61.7 ± 3.6^b^***	45.81 ± 2.8^a^***	<.0001
Heart Weight (mg)					
*Male*	127.4 ± 5.28^a^	144 ± 5.15^ab^	156.55 ± 3.34^b^	155 ± 4.96^b^	<.0001
*Female*	115.2 ± 4.71^a^	106.36 ± 4.34^a^***	144 ± 5.6^b^	138.5 ± 6.12^b^*	<.0001
Heart: body weight ratio					
*Male*	4.67 ± 0.2^a^	5.27 ± 0.04^b^	4.16 ± 0.13^a^	4.27 ± 0.2^a^	<.0001
*Female*	5.38 ± 0.24^a^*	2.33 ± 0.11^b^***	1.96 ± 0.12^b^***	5.46 ± 0.31^a^**	<.0001
Tg (mg/dL)					
*Male*	80.93 ± 7.79^a^	132.13 ± 12.63^b^	98.6 ± 11.1^ab^	112.7 ± 14.87^ab^	0.024
*Female*	77.47 ± 5.75^a^	106.13 ± 5.66^ab^	123.75 ± 15.5^b^	94 ± 11.3^ab^	0.019
Fasting Insulin (μIU/ml)					
*Male*	7.34 ± 0.43^a^	12.31 ± 0.95^b^	9.07 ± 0.64^ab^	11.44 ± 1.73^ab^	0.014
*Female*	7.34 ± 1.3	7.6 ± 1.72*	7.77 ± 1.08	8.9 ± 0.86	0.833
Fasting Glucose (mM)					
*Male*	2.93 ± 0.59	2.97 ± 0.47	4.24 ± 1.05	3.33 ± 0.78	0.585
*Female*	2.41 ± 0.31	2.78 ± 0.3	3.27 ± 0.66	2.83 ± 0.36	0.579
HOMA-IR					
*Male*	0.96 ± 0.08^a^	1.62 ± 0.07^b^	1.71 ± 0.14^b^	1.7 ± 0.09^b^	<.0001
*Female*	0.79 ± 0.11^a^	0.93 ± 0.08^ab^***	1.13 ± 0.07^b^**	1.12 ± 0.07^ab^***	0.027
T-CHOL (mg/dL)					
*Male*	119.2 ± 2.92^a^	118 ± 1.45^a^	124.67 ± 3.41^ab^	131.67 ± 1.17^b^	0.003
*Female*	121.6 ± 3.86	118 ± 3.97	130 ± 0.94	124 ± 2.97*	0.087
HDL-C (mg/dL)					
*Male*	59.18 ± 5.34^a^	69.95 ± 2.53^ab^	80.8 ± 5.44^b^	84.53 ± 3.26^b^	0.002
*Female*	49.46 ± 3.61^a^	49.42 ± 4.9^a^**	72.53 ± 4.24^b^	36.66 ± 6.57^a^***	0.001

^abc ^Uncommon letters indicate difference in means between diet groups within a row (P-value <0.05).

Differences between genders within a diet group for each variable are denoted by *,** and *** for p-values <0.05, <0.01 & <0.001

### Sexual dimorphism in cardiac gene expression

We used Affymetrix Mouse Gene 1.0 ST expression arrays to determine differences in global cardiac gene/Expressed Sequence Tags (ESTs) in response to diet, in both males and females. Using a False Discovery Rate with a significance level set at 0.05, we identified two subsets of genes/ESTs that were differentially expressed between males (n = 1803) and females (n = 1985) at various stringency levels (absolute fold change > 2.0 > 2.5, > 3.0, adjusted probability value <0.05, Figure 1B and Additional File 2 Table S2a and S2b). To better understand the relevance of these sex-dimorphic genes we used a program based on Gene Ontology (GO) categories of biological processes, molecular function and cellular component. Additional File 3, Table S3 lists the main functional GO categories of transcripts that were differentially expressed between the sexes with a stringency of ± 1.5-fold change in expression (P < 0.01). DEGs were further characterized as being relatively up-regulated either in males or in females. Increased transcription of male genes in the "Biological processes" category included genes involved in cellular macromolecule metabolism, anatomical structure development and blood vessel development. These genes included *Notch4*, interstitial matrix gene *Col3a1 *(type 3 collagen alpha 1), fibulin 1 (*Fbln1*) and integrin αV (*Itgav*). The predominant male-specific KEGG pathways included the regulation of actin cytoskeleton and antigen processing pathways (Additional File 3, Table S3). Conversely female-specific transcription were enriched in the categories of cellular and metabolic processes, including mitochondrial carbonic anhydrase 5b (*Car5B*), glutathione S-transferase 4 (*Gstm4*) and magnesium transporter 1 (*Magt1*). One KEGG-pathway for Cysteine and methionine metabolism was also upregulated.

### Chromosomal localization of differentially expressed cardiac genes

**Figure 2:**
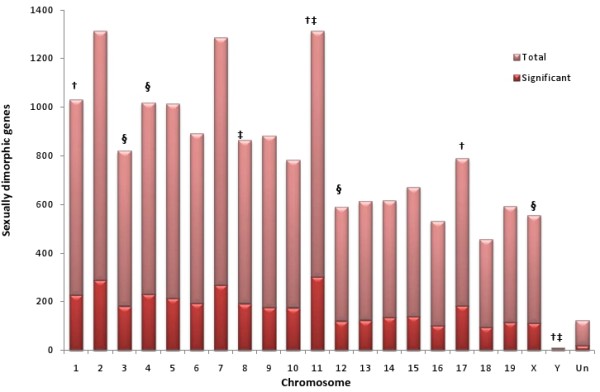
**Chromosomal enrichment analysis shows sex-specific differences**. Bar chart represents the chromosomal distribution of differentially expressed genes in the male vs. female data. Light bars represent the significant genes on each chromosome; the number of genes assayed on each chromosome is represented by the total height of each bar. Chromosomes that passed the test for enrichment by Fisher's exact test (P < 0.05) are symbolized as †, ‡, § for male vs. female, male biased and female biased gene subsets, respectively.

To investigate a possible non-random chromosomal distribution of the DEGs, we used the Fisher exact test to compare the frequencies of significant genes on each chromosome against the total number of genes interrogated on each chromosome. Chromosomes 8,11 and Y were enriched for male-biased genes whereas chromosomes 3,4,12 and X contained more female-biased genes (Figure 2, P < 0.05). Notably we found four genes encoded on the Y chromosome which were male-specific (*Sry, Ddx3y*, *Eif2s3y*, and *Uty*), and 86 female-biased X-linked genes including *Ace2*, dystrophin, and several transcription factors such as *Tceal1*, *Tceal8 *and *Eif2s3x*.

### *Trans*-fat feeding induces sex-specific alterations in cardiac gene expression

**Figure 3:**
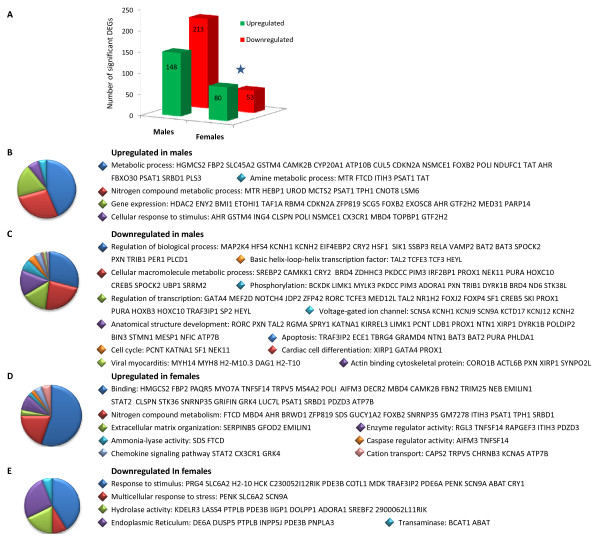
***Trans*-fat feeding induces sex-specific alterations in cardiac gene expression**. **A: **Bar chart indicates differences in numbers of significant DEGs affected by the TFA diet in males and females, * P < 0.001 by Fisher's exact test. **B-E**: Sex-specific differences in functional ontology of genes deregulated by the TFA diet. Pie charts show the number of genes that were upregulated in males **B**; downregulated in males **C**; upregulated in females **D**; downregulated in females **E**. Functional categories of DEGs were obtained using GO annotations from DAVID.

**Figure 4:**
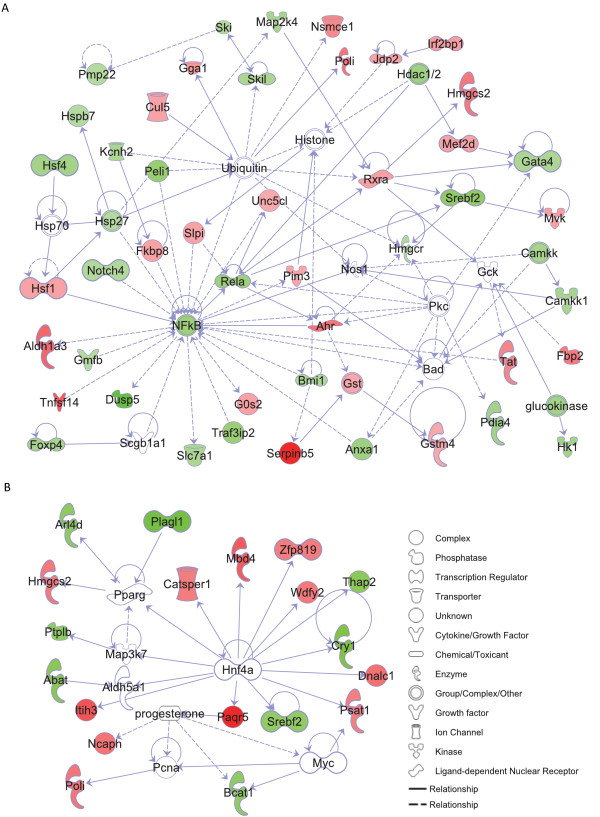
***Trans*-fat alters the expression of genes associated with cardiovascular disease and development in a sex-specific manner**. Functional relationship network representation of **A: **a subset of TFA-induced male DEGs relating to cardiovascular disease and lipid metabolism. **B: **subset of TFA-induced female DEGs relating to cardiovascular development and lipid metabolism (P < 0.01, stringency ≥ ± 1.5-fold change in expression).

In order to identify the subset of genes and ESTs with a fold difference of ± 1.5 or more within male or female comparisons for mice fed the TFA diet versus control, we used the results from a 2-way ANOVA model described in the methods section. We identified 361 DEGs (148 upregulated and 213 downregulated) that were responsive to diet in males; and approximately half as many diet-responsive DEGs in females: 133 (80 upregulated and 53 downregulated; P <0.01, Figure 3A, Additional File 4, Table S4). Specifically, the *Trans*-fat diet significantly downregulated the expression of many male cardiac genes involved in the regulation of biological processes**, **transcription and phosphorylation. Conversely females upregulated the expression of genes with functional ontologies relating to binding, nitrogen compound metabolism and ketone metabolism (Figure 3B-E, P < 0.01). Significant male DEGs downregulated by TFA consumption included key transcription modulator sterol regulatory element binding factor 2 (*Srebf2*, 3.5-fold, P < 0.01), transcription factors *Mef2d *(myocyte enhancer factor 2D, 2-fold) and *Gata4 *(2-fold); vasoconstrictive alpha-2b adrenergic receptor (2.8-fold); major cardiac Na+ channel *Scn5a *(2-fold), heat shock transcription factor 4 (*Hsf4*: 9.8-fold), calcium-regulating enzyme phospholipase C δ (1.7-fold), and circadian clock gene *Per1 *(period homolog 1: 2.6-fold). Signaling molecules calcium/calmodulin-dependent protein kinase kinase 1α (*Camkk1*) and mitogen-acrivated protein kinase kinase 4 (*Map2k4) *expression were also halved in male TFA mice. Conversely pro-hypertrophic *Hdac2 *(histone deacetylase 2), which represses *Mef2 *activity, was upregulated in males and downregulated in females. Other genes with this pattern of sex-dimorphic expression included plastin 3 (*Pls3*, 2.3-fold) and tachykinin 3 (*Tacr3*: 2.4-fold). In both sexes, the TFA diet led to increased expression of fatty acid oxidizing 3-hydroxy-3-methylglutaryl CoA synthase (Hmgcs2) and fructose bisphosphatase 2 (*Fbp*2); however expression of TNF superfamily member LIGHT/Tnfsf14 was increased 7-fold in male *Trans*-fat hearts compared to control, whereas it was only increase 2-fold in female *Trans*-fat hearts. Diet-induced changes in female hearts were less significant, with only 84 DEGs significantly up-regulated, including progestin adipoQ receptor family member V (Paqr5: 2.6-fold) and RAS guanyl releasing protein 2 (Rasgrp2, 2.2-fold). Enrichment analysis of functional GO pathways within this subset revealed that females exhibited a four-fold less down-regulation of cardiac genes with classified biological function than males. We next investigated the relationship between these genes using Ingenuity Pathways Analysis (IPA). A network of 50 DEGs relating to cardiovascular disease and lipid metabolism among others was identified as being significantly affected by the TFA diet in cardiac tissue from male mice (Figure 4A), whereas a network of 25 functionally related genes involved in female lipid metabolism and cardiovascular development is shown in Figure 4B (P < 0.01).

### Monosodium Glutamate consumption alters cardiac gene expression with sex-specific differences

A different pattern of gene expression was induced by MSG feeding, which again showed significant sex-specific differences in cardiac gene expression. 2-way ANOVA analysis revealed a subset of 153 male cardiac DEGs (98 upregulated and 55 downregulated) which were responsive to dietary MSG compared to control diet, and 174 female DEGs (117 upregulated and 57 downregulated). (Figure 1C and Additional File 5, Table S5, P < 0.01). Unlike *Trans*-fat, MSG upregulated more cardiac DEGs and down-regulated less. Genes/ESTs upregulated in males but not females in response to MSG included *Ing4 *(inhibitor of growth family member 4), glutathione S-transferase 4 and pancreatic lipase (*Pnlip*). Transcripts enriched for female-biased expression included myosin 7a (*Myo7a*), *Pdzd3 *(PDZ domain containing 3) and aldehyde dehydrogenase gene *Aldh1a3*. Functional analysis of Gene Ontologies of these subsets showed increased expression of male cellular and metabolic processes, whilst decreasing expression of phosphorylation-related DEGs (Additional File 6, Table S6, P < 0.01). Conversely females upregulated more genes/ESTs with developmental and anatomical function ontologies. Genes/ESTs that were upregulated in both sexes included ionotropic glutamate receptor *Grik3 *(2.5-fold in males and 1.7-fold in females); nebulin which regulates actin filament length (2.3-fold and 1.8-fold in males and females respectively).

### MSG alters *Trans*-fat induced cardiac gene expression

In order to address the question as to whether the addition of MSG to a *Trans*-fat diet could affect cardiac gene expression, we next identified a subset of 338 DEGs which showed significant sex-dimorphism in response to these two diets. 197 DEGs (117 upregulated and 81 downregulated) in males showed significant response to the inclusion of MSG in the *Trans *fat diet, and 141 (60 upregulated, 81 downregulated) in females (Figure 1C and Additional File 7 Table S7, P < 0.01). Of note in both sexes, expression of lipogenic stearoyl-coA desaturase 4 (*Scd4*) was upregulated in TFA+MSG diet group hearts compared to TFA alone (1.6- and 1.4-fold respectively, P < 0.01) and was accompanied by an increase in Myosin H1, magnesium transporter *Slc41a1*, and apoptosis-inducing BCL2-interacting killer (*Bik*). Downregulated DEGs included fibrulin, sclerostin and dystrophin, together with fatty acid-oxidizing enzymes phospholipase A2 (*Pla2g15*) and aldehyde dehydrogenase (*Aldh1a3*). Significant differentially expressed cardiac transcripts in TFA + MSG-fed versus TFA-fed mice were examined for enrichment of GO and KEGG classifications (Additional File 8, Table S8). In males, major functional GO categories included metabolism, biosynthesis and transcription, whereas enrichment of female DEGs upregulated by MSG feeding again fell into the categories of developmental and anatomical structural function.

### Cellular positional mapping reveals sex-specific differences in diet-deregulated gene expression

**Figure 5:**
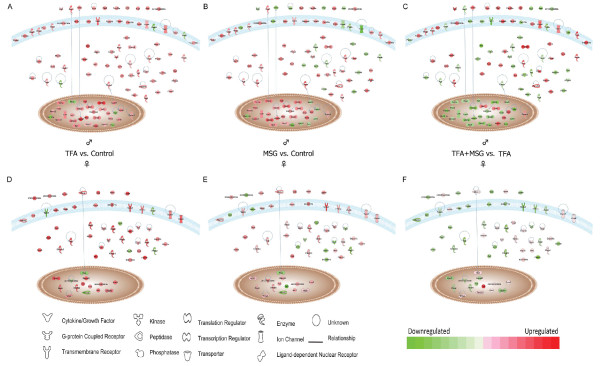
**Sexual dimorphism in global changes in cardiac gene transcription in response to all dietary manipulations co-analyzed**. Cellular positional mapping of genes deregulated by diet in males (A-C) and females (D-F) for either of the comparisons: control vs. TFA (A,D), control vs. MSG (B,E), or TFA vs. TFA+MSG (C,F; P < 0.01).

**Figure 6:**
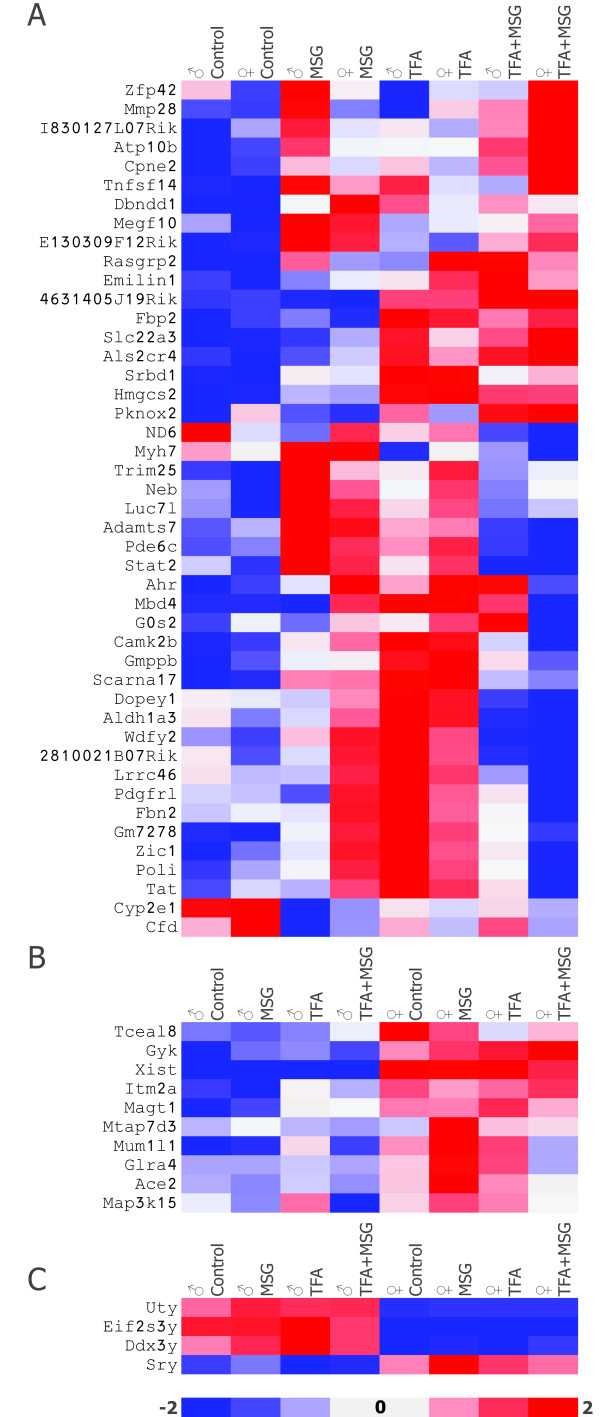
**Dietary manipulation results in deregulation of a subset of genes shared by both sexes**. **A**: Genes differentially regulated in both with a fold change of ≥ ± 1.5 for either of the comparisons control vs MSG, control vs TFA or TFA vs TFA+MSG. (P < 0.01). **B**: A selection of X-linked and **C**: Y-linked cardiac genes analyzed by microarray. Heatmap shows genes represented horizontally and diet groups represented by the vertical rows: red color signals genes with increased expression whilst blue indicated a reduction of expression, with a stringency ≥ ± 1.5-fold change.

**Figure 7:**
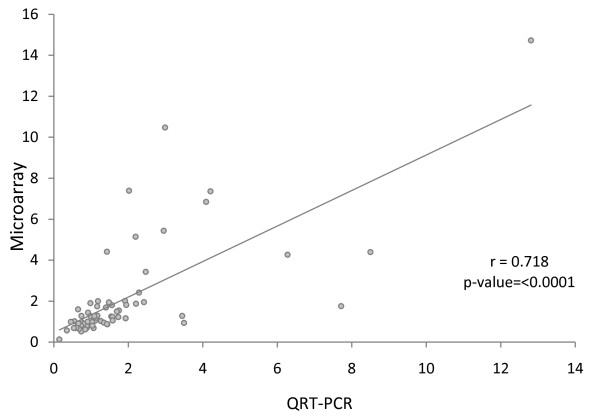
**Correlation of QRT-PCR and microarray data**. Scatter plot shows Correlation of the ratios from the microarray and real-time PCR data set. Genes that differed significantly (P < 0.01) in their regulation between the diet groups' microarray analysis were selected and validated with the same samples by real-time PCR analysis. Ratios of expressions between the diet comparisons calculated from the microarray data set correlated well with the ratio calculated from the real-time PCR data (r = 0.718, P < 0.0001). A complete list of these genes is shown in Additional File 11.

A final analysis of significant diet-induced deregulation of cardiac gene expression between all diet combinations co-analyzed yielded a cohort of 97 mapable genes which were deregulated by all dietary manipulations only in males (Figure 5 and Additional File 9, Table S9), and half that number of genes which were only deregulated in females (Figure 5 and Additional File 10, Table S10). Interestingly in this male subset, expression of genes previously demonstrated to be upregulated by TFA feeding (Figure 5A) were downregulated in animals consuming the MSG-enriched diets (Figure 5B and 5C, P < 0.01). Male-specific genes in this subset included stress-related proteins *Dnaja1 *and *Pdia4*, together with phosphodiesterase 6a and histone *H1e *. Females displayed a slightly divergent pattern (Figure 5D-F). Additionally 74 transcripts were identified as being significantly differentially expressed in both sexes in response to all dietary manipulations. Figure 6 shows a heatmap of a selection based on functional relevance, together with a number of X-linked and Y-linked diet-induced DEGs, P < 0.01 with a stringency of ± 1.5-fold change in expression. Eighteen genes were randomly chosen for further analyzed by QRT PCR based on biological relevance. Pearson correlation coefficients between the microarray analysis and QRT PCR were calculated. Ratios of expressions between the diet comparisons calculated from the microarray data set correlated well with the ratio calculated from the real-time PCR data (Figure 7, r = 0.718, P < 0.0001). A complete list of these genes and PCR primers is given in Additional File 11, Table S11.

## Discussion

Our results indicate that dietary manipulation prenatally, and over the first 8 months of life can markedly influence cardiac gene expression in a sex-specific manner. Sexual dimorphism in gene expression exists even in the healthy heart [7], and chromosome 4 has been shown to have more female-biased differential cardiac gene expression [9], whereas male-biased enrichment was found in chromosomes 3 and 14. In addition to the X and Y chromosomes which segregated as anticipated, we found female-biased chromosomal enrichment on mouse chromosomes 3, 4 and 12; and male-biased expression on chromosomes 8 and 11. Interestingly, a locus on murine chromosome 4 has been associated with markers of the Metabolic Syndrome, including body weight, insulin, leptin and triglyceride [31].

The main outcome from our study was that *Trans*-fat induced more than twice as many DEGs in males compared to females, and downregulated four times as many genes, including a number that have been implicated in cellular pathways leading to hypertrophy such as *Gata4 *and *Mef2d *[32]. Expression of *Gata4*, previously identified as being of importance in stress responsiveness [33], was halved in males in response to the TFA diet. *Gata4 *regulates transcription of a large number of cardiac genes via binding to the consensus 5'-WGATAR-3' sequence, and is implicated in cardiac muscle apoptosis, where under conditions of oxidative stress *Gata4 *downregulation is preceded by MEK/ERK-dependent phosphorylation [33]. Cardiac hypertrophy and CHD are mediated by transcription factor integration of upstream stress signals, and *Mef2d*-deficient mice have an attenuated hypertrophic response to cardiac stress [34]. Mef2d activity is repressed by the histone deacetylase Hdac2, which we found to be increased in male TFA mice. Histone deacetylases are also regulated by calmodulin and calmodulin kinase, and are among the negative regulators of cardiac hypertrophy which attenuates Mef2 transcriptional activity [32]. In the present study, we found a reduction of expression of *Camkk1 *and an increase in expression of *Camk2b*, suggesting that *Trans*-fat may deregulate both the calmodulin kinase/Mef2/Hdac pathway and the PKC/MAPK/GATA4 pathways of cardiac gene transcription.

*Trans*-fat feeding deregulated other stress-associated genes in males including phospholipase C δ1, the main cardiac isoform implicated in oxidative stress-induced REDOX signaling [35], and LIGHT/Tnfsf14, demonstrated to be upregulated in clinical and experimental heart failure [36]. Additionally, key circadian clock gene *Per1 *was decreased by more than half in the hearts of male TFA-fed mice. Downregulation of Per1 by glucose has been shown to be accompanied by changes in the expression of genes involved in lipid metabolism, transcription and cell cycle [37], such as sterol regulatory element binding factor (*Srebf2*), hydroxy-3-methylglutaryl CoA synthase (*Hmgcs*) and stearoyl-coA desaturase (*Scd*), all of which were differentially regulated by *Trans*-fat in our study. Whilst it is known that *Trans*-fat consumption is linked to cardiovascular disease [13], the mechanism behind this is incompletely understood. Elaidic acid, the main TFA in partially hydrogenated vegetable shortening, incorporates into cardiac tissues [38]; and it has been suggested that TFAs are oxidized at a slower rate than *cis*-fatty acids [39], resulting in the accumulation of reactive oxidation intermediates [40], which could lead to oxidative stress and the activation of the *Gata4, Mefd2 *and *Hdac*-associated transcriptional pathways. Taken together, our results suggest that *Trans*-fat feeding results in significant impairment of transcriptional pathways relating to cellular stress, particularly in males. It has recently been shown that male C57BL/6J mice are more vulnerable than females to the impact of a high-fat diet in terms of ensuing weight gain and deleterious metabolic peturbations [41]; and it is therefore possible that the sex-differences in *Trans *fat -induced cardiac gene expression seen in our study may have contributed to the resultant increase in male weight gain and heart: body ratio compared to females.

The MSG diet resulted in a different pattern of sexual dimorphism in cardiac transcription, with fairly equal numbers of DEGs being up- and down-regulated. However there were a number of genes which were overexpressed in males but not in females, and vice versa. The functional categories of overexpressed genes differed too, with females showing more enrichment of genes associated with developmental processes for example, whereas male upregulated transcripts were enriched for cellular and metabolic function. Neonatal injections of MSG result in smaller hearts with less DNA synthesizing activity [22]; and hypotension has been observed in female, but not male rats treated neonatally with MSG [42]. In our study, female hearts from MSG-diet group mice weighed significantly less than male hearts, however an examination of genes deregulated in our MSG mice did not provide many clues as to the mechanism responsible for this apparent difference.

We have previously shown that MSG alters *Trans *fat -induced hepatic and adipose tissue gene expression with the result that several key transcripts involved in ß-oxidation of lipids were downregulated in TFA+MSG treated mice [30]; and in our present study we found that the combination of TFA+MSG decreased the expression of several fatty acid-oxidizing enzymes such as phospholipase A2 and aldehyde dehydrogenase,whilst increasing the expression of lipogenic cardiac-specific stearoyl-coA desaturase 4 (Scd4), previously shown to be regulated by leptin and dietary factors [43].

When we compared cardiac gene transcription between all dietary combinations co-analyzed, we again found that more significant DEGs were induced in males compared to females. Our analysis identified a subset of over 100 transcripts which significantly changed only in male mice in response to diet, and approximately half this number of transcripts differentially expressed in females only, and a similar number of shared DEGs were common to both sexes. This suggests that males may have a higher cardiac transcriptional response to dietary manipulation, and indeed greater transcriptional response to exercise has previously been demonstrated in males but not females [44]. Several theories have been put forward as to this disparity, the cardioprotective effect of estrogen being one possibility [45], especially in light of epidemiological data indicating a lower incidence of female CHD, at least until menopause, after which this dissimilarity declines. However, this is unlikely to account for all the sex-specific differences observed, since after correcting for lifestyle factors, the discrepancy in CHD rates was reduced [46].

By using microarrays to analyze diet-induced cardiac gene expression patterns, we have identified genes which are differentially regulated and may play important roles in the development of CHD associated with *Trans*-fat consumption. However there are some issues to consider when interpreting the results from the present study. For the microarray experiments, we employed a pooling design in order to reduce biological variation, and to lower the costs associated with sample size and chip availability [47]. In order to address a possible loss of sensitivity and an increase in the incidence of false positives, we employed stringent criteria for the identification of the diet-regulated DEGs. One limitation of this study is the fact that these experiments were only performed on C57Bl/6J mice of both sexes. It would be advantageous but beyond the scope of the present study, to ascertain whether similar results could be obtained using a different genetic background, or possibly a higher animal model.

## Conclusion

We have found substantial evidence for sexual dimorphism in response to dietary manipulation, with males apparently showing the greater cardiac transcriptional response. In particular, we found that *Trans*-fat feeding in male mice resulted in deregulation of the expression of genes involved in cellular stress pathways. Excessive TFA consumption is believed to be a risk factor in human heart disease, the incidence of which appears to be higher in male patients. Our present observations may provoke further studies which could result in sex-specific medical intervention in the future.

## Methods

### Animals and diets

Our study animals were bred from C57BL/6J mice obtained from The Jackson Laboratory (Maine, USA). Female breeders were housed/caged and fed a standard chow diet until 6 weeks of age whereupon they were placed on one of 4 different dietary regimens for a period of 3 weeks prior to mating as described previously [30]. The four diet regimens used in this study were: [1] Standard Chow (**Control diet**) with *ad lib *drinking water. [2] Standard Chow, with *ad lib *drinking water containing 0.64 g/L Monosodium Glutamate (**MSG diet**). [3]*Trans*-fat diet of 20% (w/w) Partially Hydrogenated Vegetable Shortening (Test Diet #5C4M containing 8.68% w/w *Trans *fatty acids; Test Diet Purina, USA), with *ad lib *drinking water (**TFA diet**). [4]*Trans*-fat Diet #5C4M together with *ad lib *drinking water containing 0.64 g/L Monosodium Glutamate (**TFA+MSG diet**). See Table 2 for diet composition. Following mating, the 4 groups of dams were maintained on their respective diets throughout the gestation and nursing period. Male and female offspring (n = 20 per diet group) used in the following experiments were weighed, weaned onto the same diets and maintained on their respective dietary regimens until they reached 32 weeks of age. Average body weight was assessed at 6 and 32 weeks of age; water intake and food consumption was assessed at 6 and again at 28-30 weeks. The breeding and care of the animals were in accordance with the protocols approved by the Animal Care and Use Committee of the King Faisal Specialist Hospital & Research Centre.

**Table 2:** Composition of experimental diets

Ingredients (g/100g dietary weight)	Control	MSG	TFA	TFA+MSG
Protein (%)	22.5	22.5	19.1	19.1
Carbohydrate (%)	64.2	64.2	37.7	37.7
Fat (%) (Ether extract)	5	5	28	28
Fiber	3	3	4.3	4.3
Vitamins, Minerals & Ash	5.3	5.3	10.9	10.9
Energy (kcal/g)	3.36	3.36	4.39	4.39
MSG (mg/Kg bodyweight) provided in drinking water	0	95.13	0	95.13

**Caption: **Table 2: Diet composition.

### Measurement of fasting serum cholesterols, triglyceride, glucose, insulin and HOMA-IR levels

Serum Triglyceride (TG), T-CHOL and HDL-C concentrations were measured in overnight fasted 32-week old mice using the Reflovet Plus instrument (Roche, F. Hoffmann-La Roche Ltd, Basel, Switzerland) according to the manufacturer's instructions. Overnight fasting blood glucose was measured using the Ascensia Contour glucometer (Bayer HealthCare, IN, USA). Fasting Serum insulin was measured using the ultrasensitive mouse insulin ELISA kit from Mercodia (Uppsala, Sweden), as described previously [30]. Homeostatic Model Assessment Index (HOMA-IR) values, a measure of insulin resistance, were calculated according to the established formula: (fasting serum insulin μIU/ml) * (fasting serum glucose mM)/22.5 [48].

### RNA isolation

Animals were euthanized at 32 weeks of age by xylazine/ketamine intramuscular injection, and the hearts were rapidly removed and rinsed in saline solution. After weighing, the hearts were snap-frozen for RNA extraction. Total RNA was prepared from snap-frozen cardiac tissues from 32-week-old male and female mice (n = 8 per diet group) using Qiagen RNeasy Kit (Quiagen, CA, USA) according to the manufacturer's instructions and stored at -80°C. The integrity of total RNA was measured using a 2100 Bioanalyzer instrument and an RNA 6000 Nano LabChip assay (Agilent Technologies, CA, USA). RNA concentrations were determined by absorption at 260-nm wavelength with an ND-1000 spectrometer (Nanodrop Technologies, DE, USA).

### Gene expression analysis

Gene expression in these 64 samples was analyzed using 16 GeneChip (R) Mouse Gene 1.0 ST arrays representing 28,853 genes. We used 2 chips per diet group, and applied pooled RNA from four mice per chip (see Figure 1 for design of the microarray experiments). Targets were prepared and microarrays were processed as described in the Affymetrix GeneChip Whole Transcript Expression Analysis manual using commercially available Affymetrix GeneChip WT cDNA Synthesis Kit, WT cDNA Amplification Kit, and WT Terminal Labeling Kit as per manufacturer's instructions. Briefly, approximately 200 ng of total RNA was used to synthesize double-stranded DNA with random hexamers tagged with a T7 promoter sequence. The cDNA was used as a template for *in vitro *transcription. In the second cycle cDNA synthesis, random primers were used in reverse transcription to convert the cRNA into single-stranded DNA, which was fragmented, labeled, and hybridized to the array for 16 hours using the Fluidics 450 station. Arrays were scanned using the Affymetrix 3000 7G scanner and GeneChip Operating Software version 1.4 to produce.CEL intensity files. This software also provided summary reports by which array QA metrics were evaluated including average background, average signal, and 3'/5' expression ratios for spike-in controls, β-actin, and GAPDH. Microarray data was deposited at the MIAME compliant NCBI gene expression hybridization array data repository (GEO: http://ncbi.nlm.nih.gov/geo) under accession #GSE22881.

### Data analysis

Morphological and biochemical data were analyzed using one-way analysis of variance (ANOVA) followed by Tukey's test. Differences were considered significant at P < 0.05. For identification of differentially expressed genes (hereafter termed DEGs), microarray analysis was performed using the Partek genomic suite software version 6.3 (Partek, MO, USA) as previously described [30]. Probe set data were summarized and background adjusted using the GC-Robust Multi-Array (GCRMA) algorithm [49]. This method is used to reduce discrepancies in hybridization patterns that might result from variables in target amplification, hybridization conditions, staining or probe array lot. All data were normalized using non-linear transformation termed Quantile Normalization to improve the linearity, normality & homoscedasticity of the data, which was further filtered to remove noise and extreme expression values. A 2-way analysis of variance (ANOVA) was performed to detect significant differences among the diet groups and sexes. Probability values were adjusted for multiple testing using the False Discovery Rate (FDR) method [50].This method uses a controlled FDR while adjusting for testing simultaneously across multiple subgroup comparisons of sex and diet groups. Contrasts were included in the model based on the comparison of interest. All further sublists were created using genes that passed the FDR adjusted ANOVA p-value as well as fold change criteria. See Figure 1 for the design and statistical analysis of the microarray experiments. All resultant genes and expressed sequence tags (ESTs) meeting the criteria for differential expression were ascribed genome-wide significance using the Database for Annotation, Visualization, and Integrated Discovery (DAVID) annotation [51]. Testing for chromosomal enrichment of the DEGs was performed using the Fisher exact test. For detection of Gene Ontology (http://www.geneontology.org) categories and Kyoto Encyclopedia of Genes and Genomes (KEGG, http://www.kegg.com) pathways and pathway analysis, with significant overrepresentation of transcripts in diet groups compared to control. Heat maps of the gene/ESTs expression data were also generated using Partek. Networks of biologically related genes were created using Ingenuity Pathways Analysis (IPA: http://www.ingenuity.com). Sex-specific pathways identified from microarray analysis in the comparison of gene expression in *Trans*-fat fed mice compared to control were used to build literature-based male and female-specific pathways. In the two networks, genes or gene products are represented as colored nodes, and biological relationships between nodes are depicted as lines connecting the individual nodes. P ≤ 0.01 was considered significant, with a stringency of ± 1.5-fold difference in expression levels.

### Quantitative QPCR

Confirmation of microarray results was performed by quantitative QPCR as previously described [30]. Total RNA (2 μg) was reverse transcribed using Promega Reverse Transcription system (Promega, WI, USA). Subsequently, QPCR reactions were performed in triplicate using SYBR-Green 1 master mix (Applied Biosystems) and 10 ng cDNA as template. No template and no reverse transcriptase controls were included and products were analysed by gel electrophoresis. Real time RT-PCR values for each target gene were calculated as a ratio of target gene expression level to the 18-S ribosomal expression level in the same specimen. Statistical significance was assessed using a two-tailed t test assuming unequal variance of the biological replicates. Intron-spanning gene specific primers were designed using Primer 3 software and sequences are provided in Additional File 11. DEGs that differed significantly (P < 0.01) in their regulation between the diet groups' microarray analysis were selected, based on their biological relevance, and validated with the same samples by QRT- PCR analysis. Ratios of expressions between the diet comparisons were calculated from the microarray data set and Pearson correlation analysis between the QRT-PCR and microarray data were calculated.

## Abbreviations

KEGG: Kyoto Encyclopedia of Genes and Genome; MSG: Monosodium Glutamate; TFA: *Trans *Fatty Acid; DEGs: Differentially Expressed Genes, ESTs: Expressed Sequence Tags; GO:Gene Ontology;

## Authors' contributions

KSC devised the study, analyzed the data, wrote the manuscript and contributed to the figures and tables. MZZ analysed the data and contributed to the figures and tables; MS gave advice over statistical analysis; ZM performed the RNA extraction and Affymetrix hybridization; SMS performed the QRT-PCR; AI and NJM bred the experimental subjects, directed animal husbandry and performed all biochemical analysis, and FAA oversaw the project, contributed to the study design and critiqued the manuscript. All authors have read and approve this manuscript.

## Supplementary Material

Additional File 1 **Figure S1**. Histology of cardiac tissue. **A**: transverse section and **B**: longitudinal sections of hematoxin and eosin-stained cardiac tissue from mice in control, MSG, TFA and TFA+MSG diet groups. Scale is x790. Click here for file

Additional file 2 **Table S2a**. Differentially expressed cardiac genes in males compared to females (p ≤0.01) with a fold change of ≥ 1.5. Table S2b. Differentially expressed cardiac genes in females compared to males (p ≤0.01) with a fold change of ≥ 1.5. Click here for file

Additional file 3 **Table S3**. Gene ontologies & pathways enriched for differentially expressed genes comparing all males to all females. Click here for file

Additional file 4 **Table S4**. Differentially expressed genes in either males or females for the comparison TFA vs Control with respect to diet and sex (P < 0.01) and fold change ≥ ± 1.5. Click here for file

Additional file 5 **Table S5**. Differentially expressed genes in either males or females for the comparison MSG vs Control with respect to diet and sex (P < 0.01) and fold change ≥ ± 1.5. Click here for file

Additional file 6 **Table S6**. Gene ontologies enriched for differentially expressed genes comparing MSG to Control diet in males and females. Click here for file

Additional file 7 **Table S7**. Differentially expressed genes in either males or females for the comparison TFA+MSG vs TFA with respect to diet and sex (P < 0.01) and fold Change ≥ ± 1.5. Click here for file

Additional file 8 **Table S8**. Gene ontologies enriched for differentially expressed genes comparing TFA+MSG to TFA diet in males and females. Click here for file

Additional file 9 **Table S9**. Intensities of genes/ESTs differentially regulated only in males with a fold change of ≥ ± 1.5 for either of the comparisons Control vs MSG, Control vs TFA, or TFA vs TFA+MSG. Click here for file

Additional file 10 **Table S10**. Intensities of genes/ESTs differentially regulated only in females with a fold change of ≥ ± 1.5 for either of the comparisons Control vs MSG, Control vs TFA, or TFA vs TFA+MSG. Click here for file

Additional File 11 **Table S11**. Primers for genes selected for confirmatory QRT-PCRs. Click here for file
