# Supplementary figures and images for: Sex-dimorphism in Cardiac Nutrigenomics: effect of Trans fat and/or Monosodium Glutamate consumption
Source: BMC Genomics. 2011 Nov 12;12:555. doi: 10.1186/1471-2164-12-555 (PMC3238303; doi:10.1186/1471-2164-12-555)

**CONTROL**

**MSG**

**TFA**

**TFA+MSG**

**A**

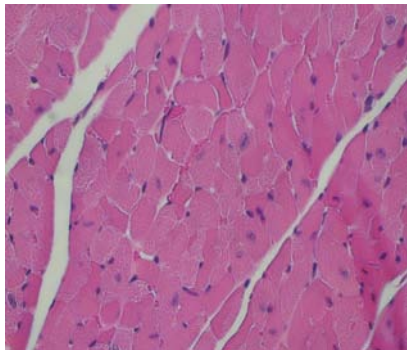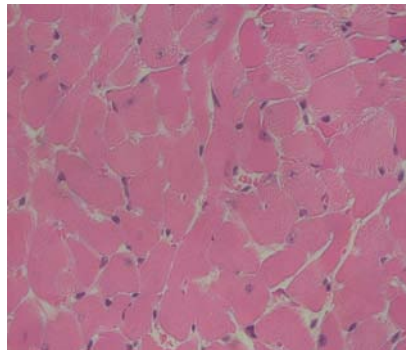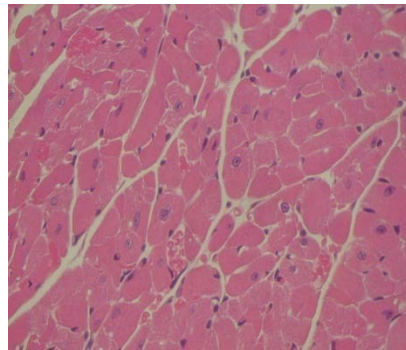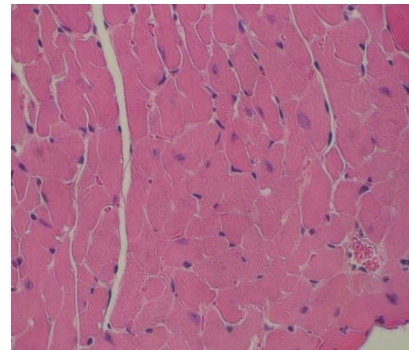

**B**

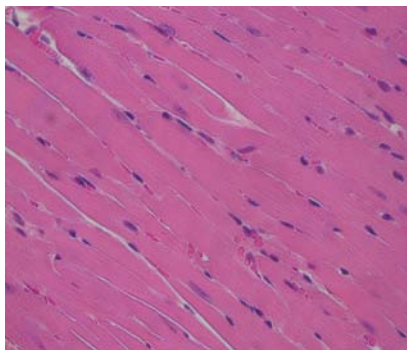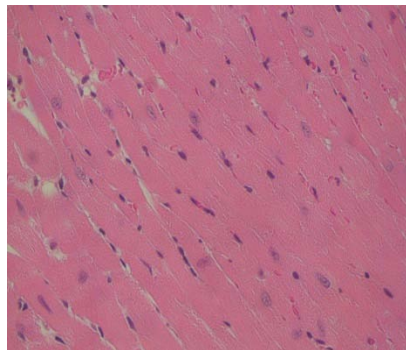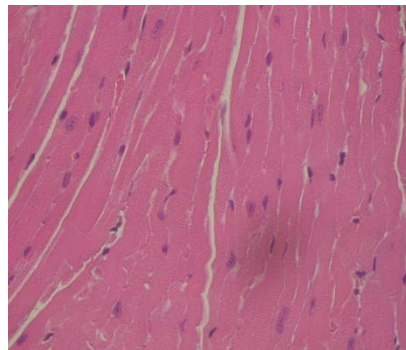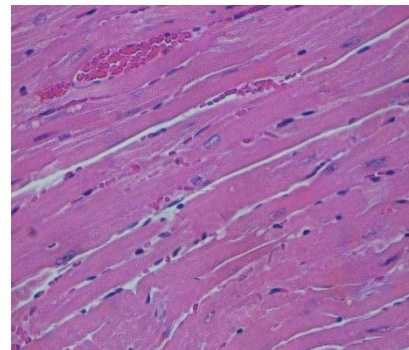

Supplement: Additional File 1 — Figure S1. Histology of cardiac tissue. A: transverse section and B: longitudinal sections of hematoxin and eosin-stained cardiac tissue from mice in control, MSG, TFA and TFA+MSG diet groups. Scale is x790. [file 1471-2164-12-555-S1.PDF]
