# Supplementary material for: Sex-dimorphism in Cardiac Nutrigenomics: effect of Trans fat and/or Monosodium Glutamate consumption
Source: BMC Genomics. 2011 Nov 12;12:555. doi: 10.1186/1471-2164-12-555 (PMC3238303; doi:10.1186/1471-2164-12-555)
Supplement: Additional file 2 — Table S2a. Differentially expressed cardiac genes in males compared to females (p ≤0.01) with a fold change of ≥ 1.5. Table S2b. Differentially expressed cardiac genes in females compared to males (p ≤0.01) with a fold change of ≥ 1.5. [file 1471-2164-12-555-S2.PDF]

Additional Table 2a. Differentially expressed cardiac genes in males compared to females (p ≤0.01) with a regulation of ≥ 1.5

DAVID GeneName	Symbol	Accession	Fold Change Male/Female
**Relatively upregulated in males > 3-fold**			
eukaryotic translation initiation factor 2, subunit 3, structural gene Y-linked	Eif2s3y	NM_012011	46.0
ubiquitously transcribed tetratricopeptide repeat gene, Y chromosome	Uty	NM_009484	12.8
caspase recruitment domain family, member 14	Card14	NM_130886	9.8
heat shock transcription factor 4	Hsf4	NM_011939	8.8
polymerase (DNA directed), mu	Polm	NM_017401	7.9
RIKEN cDNA 2700094K13 gene	2700094K13Rik	NM_001033166	7.8
complement component C7	C7	XM_904680	7.3
aldehyde dehydrogenase 1 family, member L2	Aldh1l2	NM_153543	7.3
carboxypeptidase A4	Cpa4	NM_027926	7.0
desmoglein 1 alpha	Dsg1a	NM_010079	6.3
a disintegrin and metallopeptidase domain 15 (metargidin)	Adam15	NM_001037722	6.1
cytochrome P450, family 11, subfamily b, polypeptide 2	Cyp11b2	NM_009991	5.9
neurobeachin-like 2	Nbeal2	BC157956	5.3
contactin associated protein-like 5C	Cntnap5c	NM_001081653	5.1
Prkr interacting protein 1 (IL11 inducible)	Prkrip1	NM_025774	4.9
DEAD (Asp-Glu-Ala-Asp) box polypeptide 3, Y-linked	Ddx3y	NM_012008	4.8
calpain 8	Capn8	NM_130890	4.5
solute carrier family 34 (sodium phosphate), member 3	Slc34a3	NM_080854	4.5
sterol regulatory element binding factor 2	Srebf2	NM_033218	4.4
RIKEN cDNA 6330549D23 gene	6330549D23Rik	NR_003619	4.3
visual system homeobox 1 homolog (zebrafish)	Vsx1	NM_054068	4.3
caspase recruitment domain family, member 11	Card11	NM_175362	4.2
cholecystokinin	Cck	NM_031161	4.1
predicted gene 5478	Gm5478	NR_003960	4.1
ankyrin repeat and IBR domain containing 1	Ankib1	NM_001003909	4.0
leucine rich repeat neuronal 4	Lrrn4	NM_177303	3.9
cDNA sequence BC039632	BC039632	BC147509	3.9
nuclear RNA export factor 3	Nxf3	NM_001024141	3.9
secreted and transmembrane 1B	Sectm1b	NM_026907	3.8
olfactory receptor 767	Olfr767	NM_146318	3.8
interferon beta 1, fibroblast	Ifnb1	NM_010510	3.8
thrombospondin 1	Thbs1	NM_011580	3.8
natural killer cell group 7 sequence	Nkg7	NM_024253	3.7
RIKEN cDNA 2310007L24 gene	2310007L24Rik	BC116233	3.7
Rho GDP dissociation inhibitor (GDI) gamma	Arhgdig	NM_008113	3.7
hypoxia inducible factor 3, alpha subunit	Hif3a	ENSMUST00000108492	3.7
pancreatic lipase	Pnlip	NM_026925	3.7
ring finger protein 169	Rnf169	AK173319	3.7
zinc finger protein 385A	Zfp385a	NM_013866	3.7
pleckstrin homology domain containing, family A member 7	Plekha7	NM_172743	3.7
CD27 antigen	Cd27	NM_001033126	3.7
retinoic acid receptor, alpha	Rara	NM_009024	3.6
collagen, type VI, alpha 2	Col6a2	NM_146007	3.6
murinoglobulin 1	Gm7298	XM_917532	3.6
matrix metallopeptidase 20 (enamelysin)	Mmp20	NM_013903	3.5
amyotrophic lateral sclerosis 2 (juvenile) homolog (human)	Als2	NM_028717	3.5
formin-like 1	Fmnl1	NM_019679	3.5
ankyrin repeat domain 13b	Ankrd13b	NM_172945	3.5

Additional Table 2a. Differentially expressed cardiac genes in males compared to females (p ≤0.01) with a regulation of ≥ 1.5

DAVID GeneName	Symbol	Accession	Fold Change Male/Female
discs, large (Drosophila) homolog-associated protein 2	Dlgap2	NM_172910	3.5
premature ovarian failure 1B	Pof1b	NM_181579	3.5
adrenergic receptor, alpha 2b	Adra2b	NM_009633	3.5
zinc finger protein of the cerebellum 5	Zic5	NM_022987	3.5
sodium channel, voltage-gated, type IX, alpha	Scn9a	NM_018852	3.4
myosin 1H	Myo1h	BC144867	3.4
Unknown	Gm13767	XM_001002717	3.4
calcium channel, voltage-dependent, alpha 2/delta subunit 4	Cacna2d4	NM_001033382	3.4
integrin beta 7	Itgb7	NM_013566	3.4
fibroblast growth factor 8	Fgf8	NM_010205	3.4
C-type lectin domain family 4, member a3	Clec4a3	ENSMUST00000117173	3.4
potassium voltage-gated channel, Isk-related subfamily, gene 4	Kcne4	NM_021342	3.3
docking protein 1	Dok1	NM_010070	3.3
nuclear receptor subfamily 4, group A, member 3	Nr4a3	NM_015743	3.3
RIKEN cDNA 2310057J16 gene	2310057J16Rik	NM_027171	3.3
arginyl aminopeptidase (aminopeptidase B)	Rnpep	NM_145417	3.3
tensin 3	Tns3	NM_001083587	3.3
GON-4-like protein (GON-4 homolog)	Gon4l	NM_027389	3.3
TRH-degrading enzyme	Trhde	NM_146241	3.3
T-cell acute lymphocytic leukemia 2	Tal2	NM_009317	3.3
methylenetetrahydrofolate dehydrogenase (NADP+ dependent) 1-like	Mthfd1l	NM_172308	3.2
ATP-binding cassette, sub-family A (ABC1), member 14	Abca14	NM_026458	3.2
epithelial splicing regulatory protein 2	Esrp2	NM_176838	3.2
ATP/GTP binding protein-like 2	Agbl2	NM_178755	3.2
otoancorin	Otoa	NM_139310	3.2
RNA binding motif protein 19	Rbm19	NM_028762	3.2
myelin transcription factor 1	Myt1	NM_008665	3.2
Ras association (RalGDS/AF-6) domain family (N-terminal) member 7	Rassf7	NM_025886	3.2
zinc finger protein 37	Zfp37	NM_009554	3.2
WD repeat domain 17	Wdr17	NM_028220	3.2
ADAMTS-like 3	Adamtsl3	XM_984557	3.1
forkhead box N1	Foxn1	NM_008238	3.1
RIKEN cDNA 4930579G22 gene	4930579G22Rik	NM_026916	3.1
Gem-interacting protein	Gmip	NM_198101	3.1
cadherin-like 26	Cdh26	NM_198656	3.1
proteoglycan 4 (megakaryocyte stimulating factor, articular superficial zone protein)	Prg4	NM_021400	3.1
RIKEN cDNA 2610101N10 gene	2610101N10Rik	NM_001114977	3.1
follistatin-like 1	Fstl1	NM_008047	3.1
solute carrier family 13 (sodium/sulfate symporters), member 1	Slc13a1	NM_019481	3.1
polymerase (RNA) II (DNA directed) polypeptide A	Polr2a	NM_009089	3.1
FXYD domain-containing ion transport regulator 5	Fxyd5	NM_001111073	3.1
pregnancy-specific glycoprotein 29	Psg29	NM_054064	3.0
kelch-like 1 (Drosophila)	Klhl1	NM_053105	3.0
homeo box A10	Hoxa10	NM_008263	3.0
ankyrin repeat domain 36	Ankrd36	NM_023816	3.0
ATP-binding cassette, sub-family A (ABC1), member 3	Abca3	NM_013855	3.0
gamma-aminobutyric acid (GABA) A receptor, pi	Gabrp	NM_146017	3.0
matrilin 4	Matn4	NM_013592	3.0
coiled-coil domain containing 33	Ccdc33	NM_029212	3.0
anoctamin 2	Ano2	NM_153589	3.0

Additional Table 2a. Differentially expressed cardiac genes in males compared to females (p ≤0.01) with a regulation of ≥ 1.5

DAVID GeneName	Symbol	Accession	Fold Change Male/Female
**Relatively upregulated in males > 2.5-fold**			
JAZF zinc finger 1	Jazf1	NM_173406	2.9
monocyte to macrophage differentiation-associated 2	Mmd2	NM_175217	2.9
Fc receptor-like B	Fcrlb	NM_001029984	2.9
Hedgehog-interacting protein	Hhip	NM_020259	2.9
solute carrier family 45, member 3	Slc45a3	ENSMUST00000065693	2.9
GIPC PDZ domain containing family, member 3	Gipc3	NM_148951	2.9
vesicle-associated membrane protein 5	Vamp5	NM_016872	2.9
zinc finger protein 169	Zfp169	NM_026450	2.9
cAMP responsive element binding protein 5	Creb5	ENSMUST00000114409	2.9
lipoma HMGIC fusion partner-like 2	Lhfpl2	NM_172589	2.9
predicted gene 88	Gm88	BC147714	2.9
keratinocyte differentiation associated protein	Krtdap	NM_001033131	2.9
olfactory receptor 922	Olfr922	ENSMUST00000051004	2.9
a disintegrin-like and metallopeptidase (reprolysin type) with thrombospondin type 1 motif, 9	Adamts9	ENSMUST00000113438	2.9
RAB37, member of RAS oncogene family	Rab37	NM_021411	2.9
podoplanin	Pdpn	NM_010329	2.9
alanine-glyoxylate aminotransferase 2-like 1	Agxt2l1	NM_027907	2.9
AT rich interactive domain 1B (SWI-like)	Arid1b	NM_001085355	2.9
PH domain and leucine rich repeat protein phosphatase 2	Phlpp2	NM_001122594	2.9
CD248 antigen, endosialin	Cd248	NM_054042	2.9
TOX high mobility group box family member 2	Tox2	ENSMUST00000099110	2.8
ISL1 transcription factor, LIM/homeodomain	Isl1	NM_021459	2.8
core-binding factor, runt domain, alpha subunit 2, translocated to, 3 (human)	Cbfa2t3	NM_009824	2.8
olfactory receptor 1233	Olfr1233	NM_146972	2.8
retinoblastoma binding protein 8	Rbbp8	NM_001081223	2.8
Unknown	Lilrb4	NM_013532	2.8
bridging integrator 3	Bin3	NM_021328	2.8
stathmin 1	Stmn1	NM_019641	2.8
cadherin 8	Cdh8	NM_001039154	2.8
mcf.2 transforming sequence	Mcf2	ENSMUST00000063507	2.8
calcineurin binding protein 1	Cabin1	NM_172549	2.8
zinc finger protein 697	Zfp697	NM_172863	2.8
ankyrin repeat domain 34A	Ankrd34a	NM_001024851	2.8
coiled-coil domain containing 6	Ccdc6	NM_001111121	2.8
aldehyde oxidase 3-like 1	Aox3l1	NM_001008419	2.8
membrane-spanning 4-domains, subfamily A, member 7	Ms4a7	NM_027836	2.8
ADP-ribosylation factor-like 8A	Arl8a	NM_026823	2.7
GTPase, IMAP family member 5	Gimap5	NM_175035	2.7
sodium channel, voltage-gated, type V, alpha	Scn5a	NM_021544	2.7
tripartite motif-containing 61	Trim61	NM_153110	2.7
dishevelled associated activator of morphogenesis 2	Daam2	NM_001008231	2.7
cell division cycle associated 7 like	Cdca7l	NM_146040	2.7
7-dehydrocholesterol reductase	Dhcr7	NM_007856	2.7
rad and gem related GTP binding protein 2	Rem2	NM_080726	2.7
T-cell leukemia, homeobox 2	Tlx2	NM_009392	2.7
sparc/osteonectin, cwcv and kazal-like domains proteoglycan 1	Spock1	NM_009262	2.7
RGP1 retrograde golgi transport homolog (S. cerevisiae)	Rgp1	NM_172866	2.7
CCAAT/enhancer binding protein zeta	Cebpz	NM_001024806	2.7

Additional Table 2a. Differentially expressed cardiac genes in males compared to females (p ≤0.01) with a regulation of ≥ 1.5

DAVID GeneName	Symbol	Accession	Fold Change Male/Female
fibroblast growth factor 12	Fgf12	NM_183064	2.7
collagen, type XXII, alpha 1	Col22a1	XM_907370	2.7
versican	Vcan	NM_001081249	2.7
caspase 8	Casp8	NM_009812	2.7
olfactory receptor 389	Olfr389	NM_147009	2.7
SH3-binding domain kinase family, member 2	Sbk2	NM_001146329	2.7
potassium intermediate/small conductance calcium-activated channel, subfamily N, member 2	Kcnn2	NM_080465	2.7
Rho GTPase activating protein 26	Arhgap26	NM_175164	2.7
cDNA sequence BC017643	BC017643	BC017643	2.7
RIKEN cDNA 1110034A24 gene	1110034A24Rik	NM_027269	2.7
proteasome (prosome, macropain) 26S subunit, ATPase 2	Psmc2	NM_011188	2.7
forkhead box P4	Foxp4	NM_001110824	2.7
discoidin domain receptor family, member 2	Ddr2	NM_022563	2.7
zinc finger protein 213	Zfp213	NM_001033496	2.6
sideroflexin 2	Sfxn2	NM_053196	2.6
predicted gene 101	Gm101	NM_001115074	2.6
glutamic acid decarboxylase 2	Gad2	NM_008078	2.6
FMS-like tyrosine kinase 3	Flt3	NM_010229	2.6
proteasome (prosome, macropain) assembly chaperone 3	Psmg3	NM_025604	2.6
patched domain containing 1	Ptchd1	NM_001093750	2.6
RIKEN cDNA C230052I12 gene	C230052I12Rik	NM_178643	2.6
NLR family, pyrin domain containing 1B	Nlrp1b	NM_001040696	2.6
Rho-related BTB domain containing 2	Rhobtb2	NM_153514	2.6
caspase 8 associated protein 2	Casp8ap2	NM_011997	2.6
expressed sequence AW549877	AW549877	BC016504	2.6
nuclear factor of activated T-cells, cytoplasmic, calcineurin-dependent 1	Nfatc1	NM_198429	2.6
cytospin B	Cytsb	NM_001029936	2.6
mitogen-activated protein kinase kinase kinase 14	Map3k14	NM_016896	2.6
collagen, type VI, alpha 1	Col6a1	NM_009933	2.6
spectrin beta 3	Spnb3	NM_021287	2.6
podocalyxin-like 2	Podxl2	NM_176973	2.6
tumor necrosis factor receptor superfamily, member 23	Tnfrsf23	NM_024290	2.6
actin filament associated protein 1-like 2	Afap1l2	NM_146102	2.6
histone cluster 1, H1e	Hist1h1e	NM_015787	2.6
F-box protein 17	Fbxo17	NM_015796	2.6
nuclear factor, erythroid derived 2	Nfe2	NM_008685	2.6
RAB11 family interacting protein 1 (class I)	Rab11fip1	NM_001080813	2.6
lysyl oxidase-like 1	Loxl1	NM_010729	2.6
interleukin 27 receptor, alpha	Il27ra	NM_016671	2.5
Notch gene homolog 2 (Drosophila)	Notch2	NM_010928	2.5
bicaudal C homolog 1 (Drosophila)	Bicc1	NM_031397	2.5
ankyrin repeat domain 52	Ankrd52	NM_172790	2.5
potassium intermediate/small conductance calcium-activated channel, subfamily N, member 3	Kcnn3	NM_080466	2.5
procollagen C-endopeptidase enhancer protein	Pcolce	NM_008788	2.5
cripto, FRL-1, cryptic family 1	Cfc1	NM_007685	2.5
zinc finger, DHHC domain containing 8	Zdhhc8	NM_172151	2.5
low density lipoprotein receptor	Ldlr	NM_010700	2.5
deltex 2 homolog (Drosophila)	Dtx2	NM_023742	2.5
RIKEN cDNA 4933428G20 gene	4933428G20Rik	NM_021493	2.5

Additional Table 2a. Differentially expressed cardiac genes in males compared to females (p ≤0.01) with a regulation of ≥ 1.5

DAVID GeneName	Symbol	Accession	Fold Change Male/Female
RIKEN cDNA 4930500O05 gene	4930500O05Rik	NM_001085508	2.5
CMRF-35-like molecule 3	RP23-331L12.8	NM_199201	2.5
transmembrane protein 120A	Tmem120a	NM_172541	2.5
arylsulfatase B	Arsb	NM_009712	2.5
porcupine homolog (Drosophila)	Porcn	NM_016913	2.5
D site albumin promoter binding protein	Dbp	NM_016974	2.5
amyloid beta (A4) precursor protein binding, family A, member 1	Apba1	NM_177034	2.5
C-type lectin domain family 16, member A	Clec16a	NM_177562	2.5
uridine phosphorylase 1	Upp1	NM_009477	2.5
calmodulin regulated spectrin-associated protein 1	Camsap1	NM_001115076	2.5
leucine-rich repeats and IQ motif containing 4	Lrriq4	NM_026668	2.5
zinc finger, C3HC type 1	Zc3hc1	NM_172735	2.5
schlafen like 1	Slfnl1	NM_177570	2.5
Indian hedgehog	Ihh	NM_010544	2.5
family with sequence similarity 83, member C	Fam83c	BC113797	2.5
ring finger protein 150	Rnf150	NM_177378	2.5
upstream transcription factor 2	Usf2	NM_011680	2.5
PR domain containing 2, with ZNF domain	Prdm2	NM_001081355	2.5
phytanoyl-CoA hydroxylase interacting protein	Phyhip	NM_145981	2.5
alkaline ceramidase 2	Acer2	BC059819	2.5
serine incorporator 4	RP23-433P19.11	NM_001025371	2.5
dynein, axonemal, intermediate chain 1	Dnaic1	NM_175138	2.5
**Relatively upregulated in males >2-fold**			
protein tyrosine phosphatase, receptor type, J	Ptprj	NM_008982	2.4
myogenic factor 6	Myf6	NM_008657	2.4
zinc finger protein 398	Zfp398	NM_173034	2.4
actin-like 6B	Actl6b	NM_031404	2.4
growth differentiation factor 10	Gdf10	NM_145741	2.4
sperm associated antigen 6	Spag6	NM_015773	2.4
RIKEN cDNA 4933408J17 gene	4933408J17Rik	AK016739	2.4
solute carrier family 39 (metal ion transporter), member 11	Slc39a11	NM_027216	2.4
calpain 5	Capn5	NM_007602	2.4
protocadherin alpha 10	Pcdha10	NM_009961	2.4
calmodulin binding transcription activator 2	Camta2	NM_178116	2.4
hyperpolarization-activated, cyclic nucleotide-gated K+ 1	Hcn1	NM_010408	2.4
RIKEN cDNA 5133400G04 gene	5133400G04Rik	NM_029485	2.4
NK1 transcription factor related, locus 2 (Drosophila)	Nkx1-2	NM_009123	2.4
caspase 4, apoptosis-related cysteine peptidase	Casp4	NM_007609	2.4
polyhomeotic-like 1 (Drosophila)	Phc1	NM_007905	2.4
armadillo repeat gene deleted in velo-cardio-facial syndrome	Arvcf	NM_033474	2.4
sushi-repeat-containing protein; retinitis pigmentosa GTPase regulator	Srpx	NM_016911	2.4
fibulin 7	Fbln7	NM_024237	2.4
fibrous sheath-interacting protein 2	Fsip2	ENSMUST00000099963	2.4
agmatine ureohydrolase (agmatinase)	Agmat	NM_001081408	2.4
nanos homolog 2 (Drosophila)	Nanos2	NM_194064	2.4
La ribonucleoprotein domain family, member 1	Larp1	NM_028451	2.4
glyoxylate reductase/hydroxypyruvate reductase	Grhpr	NM_080289	2.4
growth arrest-specific 2 like 1	Gas2l1	NM_030228	2.4
IQ motif containing GTPase activating protein 1	Iqgap1	NM_016721	2.4
transient receptor potential cation channel, subfamily V, member 3	Trpv3	NM_145099	2.4

Additional Table 2a. Differentially expressed cardiac genes in males compared to females (p ≤0.01) with a regulation of ≥ 1.5

DAVID GeneName	Symbol	Accession	Fold Change Male/Female
CTD (carboxy-terminal domain, RNA polymerase II, polypeptide A) small phosp	Ctdsp1	NM_153088	2.4
transmembrane and coiled-coil domains 6	Tmco6	NM_028036	2.4
olfactory receptor 1166	Olfr1166	NM_146650	2.4
proteasome (prosome, macropain) subunit, beta type 10	Psmb10	NM_013640	2.4
signal-regulatory protein alpha	Sirpa	NM_007547	2.4
defensin beta 7	Defb7	NM_139220	2.4
serine dehydratase-like	Sdsl	NM_133902	2.4
bromodomain containing 3	Brd3	NM_001113574	2.4
mitogen-activated protein kinase kinase kinase kinase 3	Map4k3	NM_001081357	2.4
neuron navigator 2	Nav2	NM_175272	2.3
nuclear transcription factor-Y alpha	Nfya	NM_001110832	2.3
tetratricopeptide repeat domain 29	Ttc29	NM_183096	2.3
placental specific protein 1	Plac1	NM_019538	2.3
RAB11 family interacting protein 3 (class II)	Rab11fip3	NM_001162869	2.3
thrombospondin 4	Thbs4	NM_011582	2.3
solute carrier family 17 (sodium-dependent inorganic phosphate cotransporter), member 7	Slc17a7	NM_182993	2.3
complement factor properdin	Cfp	NM_008823	2.3
zinc finger and BTB domain containing 7a	Zbtb7a	NM_010731	2.3
C-type lectin domain family 4, member d	Clec4d	NM_010819	2.3
hexokinase 3	Hk3	NM_001033245	2.3
RIKEN cDNA 1700057K13 gene	1700057K13Rik	BC099580	2.3
gap junction protein, alpha 8	Gja8	NM_008123	2.3
Rho GTPase activating protein 19	Arhgap19	NM_027667	2.3
guanine deaminase	Gda	NM_010266	2.3
regulator of G-protein signaling 11	Rgs11	NM_001081069	2.3
inositol polyphosphate 5-phosphatase J	Inpp5j	NM_172439	2.3
SWI/SNF related, matrix associated, actin dependent regulator of chromatin, subfamily d, member 1	Smarcd1	NM_031842	2.3
serine (or cysteine) peptidase inhibitor, clade E, member 2	Serpine2	NM_009255	2.3
nuclear factor I/C	Nfic	NM_008688	2.3
homeo box C12	Hoxc12	ENSMUST00000055562	2.3
phosphatidylserine synthase 2	Ptdss2	NM_013782	2.3
Unknown	Gm14251	XM_001481161	2.3
nuclear factor of activated T-cells, cytoplasmic, calcineurin-dependent 2 interacting protein	Nfatc2ip	NM_010900	2.3
mitochondrial translational initiation factor 3	Mtif3	ENSMUST00000066675	2.3
translocase of inner mitochondrial membrane 50 homolog (yeast)	Timm50	NM_025616	2.3
eukaryotic translation initiation factor 4E binding protein 2	Eif4ebp2	NM_010124	2.3
interleukin 2 receptor, gamma chain	Il2rg	NM_013563	2.3
collagen, type XVIII, alpha 1	Col18a1	NM_001109991	2.3
growth arrest and DNA-damage-inducible, gamma interacting protein 1	Gadd45gip1	NM_183358	2.3
myeloid/lymphoid or mixed-lineage leukemia 2	Mll2	ENSMUST00000023741	2.3
cell division cycle 2 homolog A (S. pombe)	Cdc2a	NM_007659	2.3
AE binding protein 1	Aebp1	NM_009636	2.3
general transcription factor II I repeat domain-containing 1	Gtf2ird1	NM_020331	2.3
3-ketodihydrosphingosine reductase	Kdsr	NM_027534	2.3
lin-7 homolog B (C. elegans)	Lin7b	NM_011698	2.3
speedy homolog A (Xenopus laevis)	Spdya	NM_029254	2.3
leucine rich repeat containing 16B	Lrrc16b	NM_001024645	2.3
CCR4-NOT transcription complex, subunit 3	Cnot3	NM_146176	2.3

Additional Table 2a. Differentially expressed cardiac genes in males compared to females (p ≤0.01) with a regulation of ≥ 1.5

DAVID GeneName	Symbol	Accession	Fold Change Male/Female
zinc finger, HIT domain containing 2	Znhit2	NM_013859	2.3
homeo box C10	Hoxc10	NM_010462	2.3
zinc finger protein 282	Zfp282	NM_146175	2.3
glucocorticoid modulatory element binding protein 1	Gmeb1	NM_020273	2.3
BCL2/adenovirus E1B interacting protein 1	Bnip1	NM_172149	2.3
phosphatidylinositol transfer protein, membrane-associated 2	Pitpnm2	NM_011256	2.3
bromodomain containing 4	Brd4	NM_020508	2.3
MRV integration site 1	Mrvi1	NM_010826	2.3
tight junction associated protein 1	Tjap1	NM_028751	2.3
short stature homeobox 2	Shox2	NM_013665	2.3
myelin protein zero-like 2	Mpzl2	NM_007962	2.3
sterile alpha motif domain containing 4B	Samd4b	NM_175021	2.3
NUF2, NDC80 kinetochore complex component, homolog (S. cerevisiae)	Nuf2	NM_023284	2.3
chloride channel calcium activated 1	Clca1	NM_009899	2.3
regulating synaptic membrane exocytosis 2	Rims2	NM_053271	2.3
complement component 1, q subcomponent, beta polypeptide	C1qb	NM_009777	2.3
immunoglobulin superfamily, member 6	Igsf6	NM_030691	2.3
endoplasmic reticulum (ER) to nucleus signalling 1	Ern1	NM_023913	2.3
zinc finger, DHHC domain containing 9	Zdhhc9	NM_172465	2.3
PTK2 protein tyrosine kinase 2	Ptk2	NM_007982	2.3
olfactomedin-like 3	Olfml3	NM_133859	2.3
similar to tripartite motif protein TRIM30	A530023O14Rik	NM_175648	2.3
CXXC finger 4	Cxxc4	NM_001004367	2.3
PTK2 protein tyrosine kinase 2 beta	Ptk2b	NM_001162366	2.2
cDNA sequence BC034090	BC034090	AK129406	2.2
RIKEN cDNA 5033421C21 gene	5033421C21Rik	ENSMUST00000100890	2.2
bestrophin 3	Best3	NM_001007583	2.2
SWI/SNF related, matrix associated, actin dependent regulator of chromatin, subfamily a, member 4	Smarca4	NM_011417	2.2
Rho GTPase activating protein 20	Arhgap20	NM_175535	2.2
uridine-cytidine kinase 2	Uck2	NM_030724	2.2
predicted gene 12824	Gm12824	NM_001085549	2.2
growth factor receptor bound protein 2-associated protein 3	Gab3	NM_181584	2.2
family with sequence similarity 120, member A	Fam120a	NM_001033268	2.2
transmembrane protein 134	Tmem134	NM_001078649	2.2
KRIT1, ankyrin repeat containing	Krit1	NM_030675	2.2
deleted in bladder cancer 1 (human)	Dbc1	NM_019967	2.2
DAZ interacting protein 1-like	Dzip1l	NM_028258	2.2
ubiquitin-conjugating enzyme E2O	Ube2o	NM_173755	2.2
LIM homeobox protein 3	Lhx3	NM_001039653	2.2
complement component 4A (Rodgers blood group)	C4a	ENSMUST00000015595	2.2
BTB (POZ) domain containing 16	Btbd16	NM_001081038	2.2
zinc finger CCCH-type, antiviral 1-like	Zc3hav1l	NM_172467	2.2
RIKEN cDNA 8430427H17 gene	8430427H17Rik	NM_001001986	2.2
MAD homolog 3 (Drosophila)	Smad3	NM_016769	2.2
polymerase (DNA directed), kappa	Polk	NM_012048	2.2
tet oncogene family member 2	Tet2	NM_001040400	2.2
Ras association and DIL domains	Radil	NM_178702	2.2
notum pectinacetylesterase homolog (Drosophila)	Notum	NM_175263	2.2
serine peptidase inhibitor, Kazal type 7 (putative)	Spink7	ENSMUST00000076194	2.2
dermatan sulfate epimerase	Dse	NM_172508	2.2

Additional Table 2a. Differentially expressed cardiac genes in males compared to females (p ≤0.01) with a regulation of ≥ 1.5

DAVID GeneName	Symbol	Accession	Fold Change Male/Female
Harvey rat sarcoma virus oncogene 1	Hras1	NM_008284	2.2
Ras association (RalGDS/AF-6) domain family member 5	Rassf5	NM_018750	2.2
energy homeostasis associated	Enho	NM_027147	2.2
arylsulfatase G	Arsg	NM_028710	2.2
ankyrin repeat domain 27 (VPS9 domain)	Ankrd27	NM_145633	2.2
chemokine (C-X-C motif) ligand 14	Cxcl14	NM_019568	2.2
Unknown	LOC670326	XR_031858	2.2
RIKEN cDNA 2310021P13 gene	2310021P13Rik	BC151046	2.2
plexin D1	Plxnd1	NM_026376	2.2
synaptoporin	Synpr	NM_028052	2.2
activating transcription factor 6 beta	Atf6b	NM_017406	2.2
HORMA domain containing 2	Hormad2	NM_029458	2.2
keratin 81	Krt81	ENSMUST00000061185	2.2
erythrocyte protein band 4.1-like 1	Epb4.1l1	NM_013510	2.2
G protein-coupled receptor 183	Gpr183	NM_183031	2.2
TAF4A RNA polymerase II, TATA box binding protein (TBP)-associated factor	Taf4a	NM_001081092	2.2
DNA fragmentation factor, beta subunit	Dffb	NM_007859	2.2
retinoic acid receptor responder (tazarotene induced) 2	Rarres2	NM_027852	2.2
chondroadherin	Chad	NM_007689	2.2
desmocollin 3	Dsc3	NM_007882	2.2
interleukin 17 receptor B	Il17rb	NM_019583	2.2
expressed sequence AI451617	AI451617	NM_199146	2.2
endomucin	Emcn	NM_016885	2.2
transforming growth factor, beta 1	Tgfb1	NM_011577	2.2
microtubule-associated protein tau	Mapt	NM_001038609	2.2
myosin, heavy polypeptide 11, smooth muscle	Myh11	NM_013607	2.2
organic solute transporter beta	Ostb	NM_178933	2.2
alpha-kinase 1	Alpk1	ENSMUST00000029662	2.2
transmembrane protein 132E	Tmem132e	NM_023438	2.2
glycoprotein Ib, beta polypeptide	Gp1bb	NM_001001999	2.2
superoxide dismutase 3, extracellular	Sod3	NM_011435	2.2
pyrroline-5-carboxylate reductase 1	Pycr1	NM_144795	2.2
paired-Ig-like receptor A1	Pira2	NM_011089	2.2
WNK lysine deficient protein kinase 2	Wnk2	NM_029361	2.2
gem (nuclear organelle) associated protein 5	Gemin5	NM_172558	2.2
LAG1 homolog, ceramide synthase 6	Lass6	NM_172856	2.2
cDNA sequence BC018242	BC018242	NM_144935	2.1
transmembrane protein 98	Tmem98	NM_029537	2.1
tetratricopeptide repeat domain 28	Ttc28	BC046779	2.1
anoctamin 7	Ano7	NM_207031	2.1
interferon regulatory factor 2	Irf2	NM_008391	2.1
mitochondrial ribosomal protein S16	Mrps16	NM_025440	2.1
centrosomal protein 192	Cep192	BC064462	2.1
mitogen-activated protein kinase 8 interacting protein 1	Mapk8ip1	NM_011162	2.1
ATP-binding cassette, sub-family G (WHITE), member 4	Abcg4	AF378330	2.1
DEP domain containing 1a	Depdc1a	NM_029523	2.1
PHD finger protein 8	Phf8	NM_177201	2.1
membrane-spanning 4-domains, subfamily A, member 6D	Ms4a6d	NM_026835	2.1
ATPase, Na+/K+ transporting, beta 2 polypeptide	Atp1b2	NM_013415	2.1
E2F transcription factor 3	E2f3	NM_010093	2.1

Additional Table 2a. Differentially expressed cardiac genes in males compared to females (p ≤0.01) with a regulation of ≥ 1.5

DAVID GeneName	Symbol	Accession	Fold Change Male/Female
exportin 7	Xpo7	NM_023045	2.1
teashirt zinc finger family member 3	Tshz3	NM_172298	2.1
predicted gene 9907	Gm9907	NM_019680	2.1
predicted gene 11285; similar to 60S ribosomal protein L37a	Gm11285	XR_032696	2.1
Sp140 nuclear body protein	Sp140	NM_001013817	2.1
dynamin binding protein	Dnmbp	NM_028029	2.1
LIM-domain containing, protein kinase	Limk1	NM_010717	2.1
nuclear receptor coactivator 7	Ncoa7	NM_172495	2.1
zyxin	Zyx	NM_011777	2.1
beta-site APP cleaving enzyme 1	Bace1	NM_011792	2.1
immunoglobulin superfamily, DCC subclass, member 4	Igdcc4	NM_020043	2.1
RIKEN cDNA F730043M19 gene	F730043M19Rik	ENSMUST00000063828	2.1
aldo-keto reductase family 7, member A5 (aflatoxin aldehyde reductase)	Akr7a5	NM_025337	2.1
hyaluronic acid binding protein 2	Habp2	NM_146101	2.1
tenascin XB	Tnxb	NM_031176	2.1
Unknown	Gm608	NM_001029889	2.1
solute carrier family 35 (UDP-glucuronic acid/UDP-N-acetylgalactosamine dual transporter), member D1	Slc35d1	ENSMUST00000036195	2.1
RIKEN cDNA E130112L23 gene	E130112L23Rik	NM_198249	2.1
RAS p21 protein activator 4	Rasa4	NM_133914	2.1
tribbles homolog 1 (Drosophila)	Trib1	NM_144549	2.1
salvador homolog 1 (Drosophila)	Sav1	NM_022028	2.1
RIKEN cDNA 1700027A23 gene	1700027A23Rik	NM_029604	2.1
transformation/transcription domain-associated protein	Trrap	NM_001081362	2.1
Crm, cramped-like (Drosophila)	Cramp1l	NM_020608	2.1
family with sequence similarity 154, member B	Fam154b	NM_177894	2.1
glycerophosphodiester phosphodiesterase domain containing 5	Gdpd5	NM_201352	2.1
glutamate receptor, ionotropic, NMDA3B	Grin3b	NM_130455	2.1
early growth response 1	Egr1	NM_007913	2.1
phosphodiesterase 4B, cAMP specific	Pde4b	NM_019840	2.1
microtubule-associated protein 1 A	Mtap1a	NM_032393	2.1
zinc finger protein 532	Zfp532	NM_207255	2.1
PCTAIRE-motif protein kinase 1	Pctk1	NM_011049	2.1
Unknown	LOC433762	AY140896	2.1
cytoglobin	Cygb	NM_030206	2.1
lysine (K)-specific demethylase 3A	Kdm3a	NM_173001	2.1
plexin A2	Plxna2	NM_008882	2.1
SWI/SNF related, matrix associated, actin dependent regulator of chromatin, subfamily d, member 2	Smarcd2	NM_001130187	2.1
caspase 12	Casp12	NM_009808	2.1
family with sequence similarity 38, member A	Fam38a	ENSMUST00000064316	2.1
ankyrin repeat domain 24	Ankrd24	NM_027480	2.1
uveal autoantigen with coiled-coil domains and ankyrin repeats	Uaca	NM_028283	2.1
HLA-B associated transcript 2-like	Bat2l	NM_001159634	2.1
protein kinase C and casein kinase substrate in neurons 1	Pacsin1	NM_011861	2.1
solute carrier family 38, member 8	Slc38a8	NM_001009950	2.1
RanBP-type and C3HC4-type zinc finger containing 1	Rbck1	NM_001083921	2.1
beta-carotene 15,15'-monooxygenase	Bcmo1	NM_021486	2.1
kelch-like 3 (Drosophila)	Klhl3	ENSMUST00000091583	2.1
sema domain, transmembrane domain (TM), and cytoplasmic domain, (semaphorin) 6D	Sema6d	NM_172537	2.1

Additional Table 2a. Differentially expressed cardiac genes in males compared to females (p ≤0.01) with a regulation of ≥ 1.5

DAVID GeneName	Symbol	Accession	Fold Change Male/Female
sodium channel, voltage-gated, type IV, alpha	Scn4a	NM_133199	2.1
RIKEN cDNA 4732429D16 gene	Cd300ld	NM_145437	2.1
syntaxin binding protein 2	Stxbp2	NM_011503	2.1
UDP-Gal:betaGlcNAc beta 1,4-galactosyltransferase, polypeptide 5	B4galt5	NM_019835	2.1
calcium/calmodulin-dependent serine protein kinase (MAGUK family)	Cask	NM_009806	2.1
slit homolog 3 (Drosophila)	Slit3	NM_011412	2.1
mitochondrial ribosomal protein L54	Mrpl54	NM_025317	2.1
SPARC related modular calcium binding 1	Smoc1	NM_001146217	2.1
G-protein coupled receptor 3	Gpr3	NM_008154	2.1
sema domain, immunoglobulin domain (Ig), and GPI membrane anchor, (semaphorin) 7A	Sema7a	NM_011352	2.1
GTPase, IMAP family member 8	Gimap8	NM_212486	2.1
metallothionein 4	Mt4	NM_008631	2.1
olfactory receptor 70	Olfr70	NM_019485	2.1
reversion-inducing-cysteine-rich protein with kazal motifs	Reck	NM_016678	2.1
RIKEN cDNA 1700019A02 gene	1700019A02Rik	ENSMUST00000072235	2.1
PRP40 pre-mRNA processing factor 40 homolog B (yeast)	Prpf40b	NM_018786	2.1
guanine nucleotide binding protein, alpha 12	Gna12	NM_010302	2.1
MAP/microtubule affinity-regulating kinase 2	Mark2	NM_007928	2.1
mitogen-activated protein kinase kinase 2	Map2k2	NM_023138	2.1
TAO kinase 1	Taok1	NM_144825	2.1
diaphanous homolog 1 (Drosophila)	Diap1	NM_007858	2.1
keratin 13	Krt13	NM_010662	2.1
casein alpha s2-like B	Csn1s2b	NM_009973	2.1
nuclear assembly factor 1 homolog (S. cerevisiae)	Naf1	ENSMUST00000118009	2.1
cartilage intermediate layer protein 2	Cilp2	NM_026818	2.1
golgi-specific brefeldin A-resistance factor 1	Gbf1	NM_178930	2.1
chemokine (C-C motif) ligand 11	Ccl11	NM_011330	2.1
frizzled homolog 2 (Drosophila)	Fzd2	NM_020510	2.1
G protein-coupled receptor 146	Gpr146	NM_030258	2.1
protein phosphatase 2A, regulatory subunit B (PR 53)	Ppp2r4	NM_138748	2.1
DNA methyltransferase 3B	Dnmt3b	NM_001003961	2.1
basic helix-loop-helix family, member e40	Bhlhe40	NM_011498	2.1
zinc finger, DHHC domain containing 3	Zdhhc3	NM_026917	2.1
phospholipase C-like 1	Plcl1	NM_001114663	2.1
WD repeat domain 81	Wdr81	NM_138950	2.1
potassium voltage-gated channel, subfamily G, member 3	Kcng3	NM_153512	2.1
DENN/MADD domain containing 4B	Dennd4b	NM_201407	2.1
transforming growth factor, beta receptor associated protein 1	Tgfbrap1	NM_001013025	2.1
matrix metallopeptidase 9	Mmp9	NM_013599	2.1
arginine glutamic acid dipeptide (RE) repeats	Rere	NM_001085492	2.1
par-3 partitioning defective 3 homolog B (C. elegans)	Pard3b	NM_001081050	2.1
SH2B adaptor protein 1	Sh2b1	NM_001081459	2.1
GTP binding protein 6 (putative)	Gtpbp6	NM_145147	2.1
ELK1, member of ETS oncogene family	Elk1	NM_007922	2.1
ring finger protein 213	Rnf213	AK173199	2.1
runt related transcription factor 1	Runx1	NM_001111021	2.1
anoctamin 8	Ano8	BC027790	2.1
fascin homolog 2, actin-bundling protein, retinal (Strongylocentrotus purpuratus)	Fscn2	NM_172802	2.0
tight junction protein 1	Tjp1	NM_009386	2.0

Additional Table 2a. Differentially expressed cardiac genes in males compared to females (p ≤0.01) with a regulation of ≥ 1.5

DAVID GeneName	Symbol	Accession	Fold Change Male/Female
RIKEN cDNA 0610037P05 gene	0610037P05Rik	BC011300	2.0
tyrosine kinase with immunoglobulin-like and EGF-like domains 1	Tie1	NM_011587	2.0
mediator complex subunit 29	Med29	NM_026042	2.0
family with sequence similarity 171, member B	Fam171b	NM_175514	2.0
breakpoint cluster region	Bcr	NM_001081412	2.0
S100 calcium binding protein A6 (calcyclin)	S100a6	NM_011313	2.0
poly (ADP-ribose) polymerase family, member 4	Parp4	NM_001145978	2.0
monooxygenase, DBH-like 1	Moxd1	NM_021509	2.0
TSC22 domain family, member 4	Tsc22d4	NM_023910	2.0
myosin, heavy polypeptide 14	Myh14	NM_028021	2.0
N-acetylneuraminate pyruvate lyase 2 (putative)	Dhdpsl	NM_026152	2.0
collagen, type IV, alpha 2	Col4a2	NM_009932	2.0
KDM1 lysine (K)-specific demethylase 6B	Kdm6b	NM_001017426	2.0
angiotensin I converting enzyme (peptidyl-dipeptidase A) 1	Ace	NM_207624	2.0
v-rel reticuloendotheliosis viral oncogene homolog A (avian)	Rela	NM_009045	2.0
glypican 5	Gpc5	NM_175500	2.0
forkhead box K1	Foxk1	NM_199068	2.0
chymotrypsin-like elastase family, member 1	Cela1	NM_033612	2.0
RIKEN cDNA 2310044G17 gene	2310044G17Rik	BC026384	2.0
homeodomain interacting protein kinase 2	Hipk2	NM_001136065	2.0
GRAM domain containing 1B	Gramd1b	NM_172768	2.0
PHD finger protein 2	Phf2	NM_011078	2.0
vasodilator-stimulated phosphoprotein	Vasp	NM_009499	2.0
phosphatidylinositol-specific phospholipase C, X domain containing 1	Plcxd1	NM_207279	2.0
RIKEN cDNA 2310068J16 gene	2310068J16Rik	AK010113	2.0
proline-rich Gla (G-carboxyglutamic acid) polypeptide 2	Prrg2	NM_022999	2.0
parvin, beta	Parvb	NM_133167	2.0
sorting nexin 16	Snx16	NM_029068	2.0
leucine-rich repeats and immunoglobulin-like domains 1	Lrig1	NM_008377	2.0
mitochondrial ribosomal protein L20	Mrpl20	NM_025570	2.0
SET domain containing 1A	Setd1a	NM_178029	2.0
proteasome (prosome, macropain) 26S subunit, ATPase 3, interacting protein	Psmc3ip	NM_008949	2.0
metastasis associated 1	Mta1	NM_054081	2.0
nuclear receptor co-repressor 2	Ncor2	NM_011424	2.0
SAPS domain family, member 2	Saps2	NM_026813	2.0
colony stimulating factor 2 receptor, beta 2, low-affinity (granulocyte-macrophage)	Csf2rb2	NM_007781	2.0
neuroligin 2	Nlgn2	NM_198862	2.0
proline rich 7 (synaptic)	Prr7	NM_001030296	2.0
expressed sequence R74862	R74862	BC005669	2.0
family with sequence similarity 125, member B	Fam125b	BC049129	2.0
predicted gene 10530	Gm10530	ENSMUST00000097514	2.0
cytochrome b-245, beta polypeptide	Cybb	NM_007807	2.0
eukaryotic translation initiation factor 2C, 2	Eif2c2	NM_153178	2.0
RIKEN cDNA 1810063B07 gene	1810063B07Rik	NM_026209	2.0
zinc finger CCCH-type containing 18	Zc3h18	NM_001029993	2.0
ribosomal protein S14	Rps14	NM_020600	2.0
golgi associated, gamma adaptin ear containing, ARF binding protein 1	Gga1	NM_145929	2.0
centrosomal protein 170	Cep170	NM_001099637	2.0
RIKEN cDNA 2310014H01 gene	2310014H01Rik	NM_175242	2.0

Additional Table 2a. Differentially expressed cardiac genes in males compared to females (p ≤0.01) with a regulation of ≥ 1.5

DAVID GeneName	Symbol	Accession	Fold Change Male/Female
H19 fetal liver mRNA	H19	NR_001592	2.0
keratin 83	Krt83	NM_001003668	2.0
zinc finger protein 42	Zfp42	NM_009556	2.0
fructosamine 3 kinase	Fn3k	NM_001038699	2.0
T-cell immunoglobulin and mucin domain containing 4	Timd4	NM_178759	2.0
Ras interacting protein 1	Rasip1	NM_028544	2.0
family with sequence similarity 49, member B	Fam49b	NM_144846	2.0
endothelial cell-specific chemotaxis regulator	Ecscr	NM_001033141	2.0
G protein-coupled receptor 177	Gpr177	NM_026582	2.0
small optic lobes homolog (Drosophila)	Solh	BC058094	2.0
transducin (beta)-like 1 X-linked	Tbl1x	NM_020601	2.0
DnaJ (Hsp40) homolog, subfamily B, member 12	Dnajb12	NM_019965	2.0
RIKEN cDNA 6030419C18 gene	6030419C18Rik	BC096614	2.0
zinc finger, CCHC domain containing 14	Zcchc14	NM_080855	2.0
guanine nucleotide binding protein (G protein), alpha inhibiting 1	Gnai1	NM_010305	2.0
polymerase (DNA-directed), delta 3, accessory subunit	Pold3	NM_133692	2.0
a disintegrin and metallopeptidase domain 11	Adam11	NM_001110778	2.0
sterol regulatory element binding transcription factor 1	Srebf1	NM_011480	2.0
zinc finger, SWIM domain containing 6	Zswim6	NM_145456	2.0
microtubule-associated protein 6	Mtap6	NM_010837	2.0
sprouty homolog 4 (Drosophila)	Spry4	NM_011898	2.0
zinc finger and BTB domain containing 3	Zbtb3	NM_001098237	2.0
syntaxin 6	Stx6	NM_021433	2.0
protein kinase domain containing, cytoplasmic	Pkdcc	NM_134117	2.0
RIKEN cDNA 1810012P15 gene	1810012P15Rik	NM_001076681	2.0
NF-kappaB repressing factor	Nkrf	NM_029891	2.0
integrator complex subunit 1	Ints1	NM_026748	2.0
RIKEN cDNA 2310067B10 gene	2310067B10Rik	NM_028014	2.0
prospero-related homeobox 1	Prox1	NM_008937	2.0
tripartite motif-containing 3	Trim3	NM_018880	2.0
euchromatic histone methyltransferase 1	Ehmt1	NM_001012518	2.0
regulatory associated protein of MTOR, complex 1	Rptor	NM_028898	2.0
valyl-tRNA synthetase 2, mitochondrial (putative)	Vars2	NM_175137	2.0
adaptor protein complex AP-2, alpha 1 subunit	Ap2a1	NM_007458	2.0
multiple EGF-like-domains 8	Megf8	NM_001160400	2.0
interferon activated gene 205	Ifi205	NM_172648	2.0
mitochondrial ribosomal protein L17	Mrpl17	NM_025301	2.0
RIKEN cDNA 9430020K01 gene	9430020K01Rik	NM_001081963	2.0
GATS protein-like 2	Gatsl2	NM_030719	2.0
glycerol phosphate dehydrogenase 2, mitochondrial	Gpd2	NM_001145820	2.0
trafficking protein, kinesin binding 1	Trak1	NM_175114	2.0
development and differentiation enhancing factor 2	Ddef2	NM_001098168	2.0
major facilitator superfamily domain containing 4	Mfsd4	NM_172510	2.0
zinc finger, CCHC domain containing 2	Zcchc2	NM_001122676	2.0
anterior pharynx defective 1a homolog (C. elegans)	Aph1a	NM_146104	2.0
serologically defined colon cancer antigen 3	Sdccag3	NM_026563	2.0
GRAM domain containing 4	Gramd4	NM_172611	2.0
formin-like 3	Fmnl3	NM_011711	2.0
splicing factor, arginine/serine-rich 4 (SRp75)	Sfrs4	NM_020587	2.0
macrophage galactose N-acetyl-galactosamine specific lectin 2	Mgl2	NM_145137	2.0

Additional Table 2a. Differentially expressed cardiac genes in males compared to females (p ≤0.01) with a regulation of ≥ 1.5

DAVID GeneName	Symbol	Accession	Fold Change Male/Female
**Relatively upregulated in males >1.5-fold**			
coronin, actin binding protein, 2B	Coro2b	NM_175484	1.9
TNFAIP3 interacting protein 1	Tnip1	NM_021327	1.9
HD domain containing 2	Hddc2	NM_027168	1.9
Casitas B-lineage lymphoma-like 1	Cbll1	NM_134048	1.9
RIKEN cDNA 2010002N04 gene	2010002N04Rik	NM_134133	1.9
zinc finger protein 598	Zfp598	NM_183149	1.9
sema domain, immunoglobulin domain (Ig), short basic domain, secreted, (semaphorin) 3C	Sema3c	NM_013657	1.9
spondin 2, extracellular matrix protein	Spon2	NM_133903	1.9
glutaredoxin 5 homolog (S. cerevisiae)	Glrx5	NM_028419	1.9
procollagen lysine, 2-oxoglutarate 5-dioxygenase 2	Plod2	NM_001142916	1.9
adenosine deaminase, RNA-specific	Adar	NM_019655	1.9
nuclear factor of activated T-cells 5	Nfat5	NM_133957	1.9
late cornified envelope 3B	Lce3b	NM_025501	1.9
cDNA sequence BC057079	BC057079	NM_001081184	1.9
furin (paired basic amino acid cleaving enzyme)	Furin	NM_011046	1.9
G protein-coupled receptor kinase-interactor 1	Git1	NM_001004144	1.9
sphingosine-1-phosphate receptor 2	S1pr2	NM_010333	1.9
ring finger protein 214	Rnf214	NM_178709	1.9
inscuteable homolog (Drosophila)	Insc	NM_173767	1.9
checkpoint with forkhead and ring finger domains	Chfr	NM_172717	1.9
arginyl aminopeptidase (aminopeptidase B)-like 1	Rnpepl1	NM_181405	1.9
bromodomain adjacent to zinc finger domain, 2A	Baz2a	NM_054078	1.9
fibronectin 1	Fn1	NM_010233	1.9
protocadherin gamma subfamily A, 1	Pcdhga1	NM_033584	1.9
MON2 homolog (yeast)	Mon2	NM_153395	1.9
WD repeat domain 38	Wdr38	NM_029687	1.9
arylsulfatase A	Arsa	NM_009713	1.9
nidogen 2	Nid2	NM_008695	1.9
RIKEN cDNA 4732471D19 gene	4732471D19Rik	NM_176987	1.9
asparagine-linked glycosylation 8 homolog (yeast, alpha-1,3-glucosyltransferase)	Alg8	NM_199035	1.9
eukaryotic translation initiation factor 2C, 4	Eif2c4	NM_153177	1.9
thyroid hormone receptor alpha	Thra	NM_178060	1.9
cartilage intermediate layer protein, nucleotide pyrophosphohydrolase	Cilp	NM_173385	1.9
malignant fibrous histiocytoma amplified sequence 1	Mfhas1	NM_001081279	1.9
upstream transcription factor 1	Usf1	NM_009480	1.9
CDC42 GTPase-activating protein	Cdgap	NM_020260	1.9
breast cancer anti-estrogen resistance 1	Bcar1	NM_009954	1.9
actin, alpha 1, skeletal muscle	Acta1	NM_009606	1.9
syntaxin 11	Stx11	ENSMUST00000042861	1.9
chloride intracellular channel 1	Clic1	NM_033444	1.9
solute carrier family 16 (monocarboxylic acid transporters), member 2	Slc16a2	NM_009197	1.9
transmembrane and coiled-coil domains 7	Tmco7	NM_173037	1.9
cytochrome P450, family 3, subfamily a, polypeptide 44	Cyp3a44	NM_177380	1.9
adaptor protein complex AP-1, beta 1 subunit	Ap1b1	NM_007454	1.9
mediator of DNA damage checkpoint 1	Mdc1	NM_001010833	1.9
nuclear receptor subfamily 3, group C, member 2	Nr3c2	NM_001083906	1.9
tubulin polymerization-promoting protein family member 2	Tppp2	NM_001128634	1.9
small EDRK-rich factor 1	Serf1	NM_011353	1.9

Additional Table 2a. Differentially expressed cardiac genes in males compared to females (p ≤0.01) with a regulation of ≥ 1.5

DAVID GeneName	Symbol	Accession	Fold Change Male/Female
Jun dimerization protein 2	Jdp2	AB034697	1.9
transformation related protein 53	Trp53	NM_011640	1.9
zinc finger protein 3	Zfp3	NM_177565	1.9
NIMA (never in mitosis gene a)-related expressed kinase 4	Nek4	NM_011849	1.9
coiled-coil domain containing 93	Ccdc93	NM_001025156	1.9
low-density lipoprotein receptor-related protein 10	Lrp10	NM_022993	1.9
protein O-fucosyltransferase 2	Pofut2	NM_030262	1.9
SWI/SNF related, matrix associated, actin dependent regulator of chromatin, subfamily c, member 1	Smarcc1	NM_009211	1.9
cell division cycle 45 homolog (S. cerevisiae)-like	Cdc45l	NM_009862	1.9
CREB regulated transcription coactivator 3	Crtc3	NM_173863	1.9
mediator of RNA polymerase II transcription, subunit 25 homolog (yeast)	Med25	NM_029365	1.9
exostoses (multiple)-like 3	Extl3	NM_018788	1.9
family with sequence similarity 125, member A	Fam125a	NM_028617	1.9
Werner syndrome homolog (human)	Wrn	NM_011721	1.9
fermitin family homolog 3 (Drosophila)	Fermt3	NM_153795	1.9
transient receptor potential cation channel, subfamily V, member 4	Trpv4	NM_022017	1.9
BMP-binding endothelial regulator	Bmper	NM_028472	1.9
disabled homolog 2 (Drosophila) interacting protein	Dab2ip	NM_001114125	1.9
diacylglycerol kinase zeta	Dgkz	ENSMUST00000111304	1.9
B-cell CLL/lymphoma 9	Bcl9	NM_029933	1.9
androgen binding protein gamma	Abpg	NM_178308	1.9
netrin G2	Ntng2	NM_133500	1.9
WW domain binding protein 7	Wbp7	NM_029274	1.9
cleavage and polyadenylation specific factor 7	Cpsf7	NM_172302	1.9
serine/threonine kinase 32B	Stk32b	NM_022416	1.9
protein phosphatase 1, regulatory (inhibitor) subunit 16A	Ppp1r16a	NM_033371	1.9
CTF18, chromosome transmission fidelity factor 18 homolog (S. cerevisiae)	Chtf18	NM_145409	1.9
solute carrier family 39 (zinc transporter), member 14	Slc39a14	NM_001135151	1.9
intercellular adhesion molecule 4, Landsteiner-Wiener blood group	Icam4	NM_023892	1.9
SH3/ankyrin domain gene 3	Shank3	NM_021423	1.9
RAB guanine nucleotide exchange factor (GEF) 1	Rabgef1	NM_019983	1.9
B lymphoid kinase	Blk	NM_007549	1.9
zinc finger, MIZ-type containing 1	Zmiz1	NM_183208	1.9
integrin alpha 9	Itga9	NM_133721	1.9
mixed lineage kinase domain-like	Mlkl	NM_029005	1.9
synaptosomal-associated protein 29	Snap29	NM_023348	1.9
zinc and ring finger 1	Znrf1	NM_133206	1.9
CD68 antigen	Cd68	NM_009853	1.9
predicted gene 5577	Gm5577	AK020089	1.9
plexin domain containing 1	Plxdc1	NM_028199	1.9
Ttk protein kinase	Ttk	NM_009445	1.9
jumonji domain containing 1C	Jmjd1c	NM_207221	1.9
methylthioribose-1-phosphate isomerase homolog (S. cerevisiae)	Mri1	NM_026423	1.9
1-acylglycerol-3-phosphate O-acyltransferase 2 (lysophosphatidic acid acyltransferase, beta)	Agpat2	NM_026212	1.9
family with sequence similarity 53, member A	Fam53a	NM_178390	1.9
calcium/calmodulin-dependent protein kinase II alpha	Camk2a	NM_009792	1.9
ADP-ribosylation factor 5	Arf5	NM_007480	1.9
dimethylarginine dimethylaminohydrolase 1	Ddah1	NM_026993	1.9

Additional Table 2a. Differentially expressed cardiac genes in males compared to females (p ≤0.01) with a regulation of ≥ 1.5

DAVID GeneName	Symbol	Accession	Fold Change Male/Female
zinc fingers and homeoboxes 3	Zhx3	NM_177263	1.9
ankyrin repeat domain 39	Ankrd39	NM_026241	1.9
tachykinin receptor 1	Tacr1	NM_009313	1.9
RAS protein-specific guanine nucleotide-releasing factor 1	Rasgrf1	NM_011245	1.9
DEAD (Asp-Glu-Ala-Asp) box polypeptide 54	Ddx54	NM_028041	1.9
acyl-CoA synthetase long-chain family member 5	Acsl5	NM_027976	1.9
regulator of G-protein signaling 20	Rgs20	NM_021374	1.9
BTB (POZ) domain containing 6	Btbd6	NM_201646	1.9
WAS/WASL interacting protein family, member 2	Wipf2	NM_197940	1.9
cadherin 1	Cdh1	NM_009864	1.9
multimerin 2	Mmrn2	NM_153127	1.9
Unknown	Gp49a	NM_008147	1.9
ATPase type 13A3	Atp13a3	NM_001128096	1.9
myosin VB	Myo5b	NM_201600	1.9
myocardin	Myocd	NM_145136	1.9
zinc finger, NFX1-type containing 1	Znfx1	NM_001033196	1.9
Unknown	Rpl37a	NM_009084	1.9
Unknown	LOC100047867	XM_001479852	1.9
chemokine (C-C motif) ligand 9	Ccl9	NM_011338	1.9
mesenchyme homeobox 1	Meox1	NM_010791	1.9
transmembrane protein 63a	Tmem63a	NM_144794	1.9
DEAH (Asp-Glu-Ala-His) box polypeptide 16	Dhx16	NM_026987	1.9
zinc finger protein, multitype 1	Zfpm1	NM_009569	1.9
RIKEN cDNA 2310014D11 gene	2310014D11Rik	AK009333	1.9
claudin 15	Cldn15	NM_021719	1.9
leprecan 1	Lepre1	NM_019782	1.9
similar to 60S ribosomal protein L37a	LOC640991	XM_918258	1.9
Unknown	BC059842	FJ998170	1.9
LIM and SH3 protein 1	Lasp1	NM_010688	1.9
Hus1 homolog b (S. pombe)	Hus1b	ENSMUST00000102943	1.9
Unknown	Camk2g	NM_178597	1.9
RIKEN cDNA 9430038I01 gene	9430038I01Rik	NM_029886	1.9
unc-5 homolog C (C. elegans)-like	Unc5cl	NM_152823	1.9
potassium voltage-gated channel, shaker-related subfamily, beta member 2	Kcnab2	NM_010598	1.9
RIKEN cDNA E030049G20 gene	E030049G20Rik	NM_001081756	1.9
tankyrase 1 binding protein 1	Tnks1bp1	NM_001081260	1.9
tousled-like kinase 1	Tlk1	NM_172664	1.9
calpain 3	Capn3	NM_007601	1.9
nuclear receptor interacting protein 2	Nrip2	NM_001162858	1.9
deleted in liver cancer 1	Dlc1	NM_015802	1.9
DNA-damage-inducible transcript 4-like	Ddit4l	NM_030143	1.9
septin 9	40430	NM_001113486	1.9
RIKEN cDNA 9430025M13 gene	9430025M13Rik	AK034698	1.9
mediator complex subunit 13-like	Med13l	NM_172424	1.9
sphingomyelin synthase 2	Sgms2	NM_028943	1.9
aminolevulinate, delta-, dehydratase	Alad	NM_008525	1.9
intersectin 1 (SH3 domain protein 1A)	Itsn1	NM_010587	1.9
family with sequence similarity 78, member B	Fam78b	NM_001160262	1.9
zinc finger protein 827	Zfp827	NM_178267	1.9
translocase of inner mitochondrial membrane 22 homolog (yeast)	Timm22	NM_019818	1.9

Additional Table 2a. Differentially expressed cardiac genes in males compared to females (p ≤0.01) with a regulation of ≥ 1.5

DAVID GeneName	Symbol	Accession	Fold Change Male/Female
c-src tyrosine kinase	Csk	NM_007783	1.9
thrombospondin, type I, domain containing 7A	Thsd7a	ENSMUST00000122369	1.9
PHD finger protein 21B	Phf21b	NM_001081166	1.9
COMM domain containing 7	Commd7	NM_133850	1.8
chemokine (C-C motif) ligand 21A	Ccl21a	NM_011124	1.8
BAT2 domain containing 1	Bat2d	NM_001081290	1.8
synaptopodin 2-like	Synpo2l	NM_175132	1.8
scribbled homolog (Drosophila)	Scrib	NM_134089	1.8
AT rich interactive domain 5B (MRF1-like)	Arid5b	NM_023598	1.8
guanine nucleotide binding protein (G protein), beta 2	Gnb2	NM_010312	1.8
expressed sequence AU040320	AU040320	NM_001035526	1.8
KDM3B lysine (K)-specific demethylase 3B	Kdm3b	NM_001081256	1.8
syntrophin, basic 1	Sntb1	BC003748	1.8
transmembrane serine protease 6	Tmprss6	NM_027902	1.8
predicted gene 2292	Gm2292	XM_001478816	1.8
Rho guanine nucleotide exchange factor (GEF) 5	Arhgef5	NM_133674	1.8
HLA-B-associated transcript 3	Bat3	NM_057171	1.8
plexin C1	Plxnc1	NM_018797	1.8
macrophage galactose N-acetyl-galactosamine specific lectin 1	Mgl1	NM_010796	1.8
interleukin 8 receptor, beta	Il8rb	NM_009909	1.8
Unknown	9130213B05Rik	EF564364	1.8
inositol polyphosphate-5-phosphatase E	Inpp5e	NM_033134	1.8
phosphodiesterase 4A, cAMP specific	Pde4a	NM_183408	1.8
zinc finger protein 362	Zfp362	NM_001081098	1.8
YTH domain containing 2	Ythdc2	NM_001163013	1.8
glycogen synthase kinase 3 beta	Gsk3b	NM_019827	1.8
melanoma antigen, family D, 1	Maged1	NM_019791	1.8
ELAV (embryonic lethal, abnormal vision, Drosophila)-like 1 (Hu antigen R)	Elavl1	NM_010485	1.8
cytohesin 4	Cyth4	NM_028195	1.8
dual serine/threonine and tyrosine protein kinase	Dstyk	NM_172516	1.8
guanylate cyclase activator 1a (retina)	Guca1a	NM_008189	1.8
a disintegrin-like and metallopeptidase (reprolysin type) with thrombospondin type 1 motif, 13	Adamts13	NM_001001322	1.8
midnolin	Midn	NM_021565	1.8
neurotrophin receptor associated death domain	Nradd	NM_026012	1.8
RIKEN cDNA 2410025L10 gene	2410025L10Rik	NM_001142642	1.8
DiGeorge syndrome critical region gene 6	Dgcr6	NM_010047	1.8
abhydrolase domain containing 8	Abhd8	NM_022419	1.8
adenylate cyclase 4	Adcy4	NM_080435	1.8
MAP-kinase activating death domain	Madd	NM_145527	1.8
RIKEN cDNA C030046E11 gene	C030046E11Rik	NM_001081319	1.8
polymerase (DNA directed) sigma	Pols	NM_198600	1.8
GATA zinc finger domain containing 2A	Gatad2a	NM_145596	1.8
ataxin 7-like 3	Atxn7l3	NM_001098836	1.8
KDEL (Lys-Asp-Glu-Leu) containing 2	Kdelc2	NM_212445	1.8
triple functional domain (PTPRF interacting)	Trio	NM_001081302	1.8
collagen, type I, alpha 2	Col1a2	NM_007743	1.8
fascin homolog 1, actin bundling protein (Strongylocentrotus purpuratus)	Fscn1	NM_007984	1.8
LEM domain containing 2	Lemd2	NM_146075	1.8

Additional Table 2a. Differentially expressed cardiac genes in males compared to females (p ≤0.01) with a regulation of ≥ 1.5

DAVID GeneName	Symbol	Accession	Fold Change Male/Female
nuclear factor of activated T-cells, cytoplasmic, calcineurin-dependent 3	Nfatc3	NM_010901	1.8
integrin alpha 6	Itga6	NM_008397	1.8
leucine-rich repeat kinase 2	Lrrk2	NM_025730	1.8
phospholipid scramblase 3	Plscr3	NM_023564	1.8
interleukin 4 induced 1	Il4i1	NM_010215	1.8
palladin, cytoskeletal associated protein	Palld	NM_001081390	1.8
general transcription factor III A	Gtf3a	NM_025652	1.8
FEV (ETS oncogene family)	Fev	NM_153111	1.8
protein phosphatase 2, regulatory subunit B (B56), beta isoform	Ppp2r5b	NM_198168	1.8
coiled-coil and C2 domain containing 1A	Cc2d1a	NM_145970	1.8
SH2B adaptor protein 2	Sh2b2	NM_018825	1.8
potassium voltage gated channel, Shaw-related subfamily, member 4	Kcnc4	NM_145922	1.8
cytochrome P450, family 4, subfamily f, polypeptide 39	Cyp4f39	NM_177307	1.8
mannoside acetylglucosaminyltransferase 4, isoenzyme B	Mgat4b	NM_145926	1.8
CDK5 and Abl enzyme substrate 1	Cables1	NM_001146287	1.8
RIKEN cDNA A930011G23 gene	A930011G23Rik	ENSMUST00000074365	1.8
collagen, type V, alpha 1	Col5a1	NM_015734	1.8
mediator of RNA polymerase II transcription, subunit 12 homolog (yeast)-like	Med12l	NM_177855	1.8
C1q and tumor necrosis factor related protein 1	C1qtnf1	NM_019959	1.8
N-deacetylase/N-sulfotransferase (heparan glucosaminyl) 2	Ndst2	NM_010811	1.8
protein tyrosine phosphatase, receptor type, U	Ptpru	NM_011214	1.8
ankyrin repeat domain 16	Ankrd16	NM_177268	1.8
tripartite motif-containing 47	Trim47	NM_172570	1.8
phosphofructokinase, liver, B-type	Pfkl	NM_008826	1.8
serine/arginine repetitive matrix 2	Srrm2	NM_175229	1.8
SECIS binding protein 2-like	Secisbp2l	NM_177608	1.8
eukaryotic translation initiation factor 4, gamma 1	Eif4g1	NM_145941	1.8
FYVE, RhoGEF and PH domain containing 5	Fgd5	NM_172731	1.8
F-box and leucine-rich repeat protein 17	Fbxl17	ENSMUST00000024761	1.8
ATPase family, AAA domain containing 5	Atad5	NM_001029856	1.8
formin-like 2	Fmnl2	NM_172409	1.8
CaM kinase-like vesicle-associated	Camkv	NM_145621	1.8
phosphodiesterase 1C	Pde1c	NM_011054	1.8
ankyrin repeat domain 11	Ankrd11	NM_001081379	1.8
glycine amidinotransferase (L-arginine:glycine amidinotransferase)	Gatm	NM_025961	1.8
RIKEN cDNA D630039A03 gene	D630039A03Rik	BC095953	1.8
kinesin family member 1C	Kif1c	NM_153103	1.8
PHD finger protein 12	Phf12	NM_174852	1.8
immunoglobulin-like domain containing receptor 1	Ildr1	NM_134109	1.8
RNA binding motif protein 24	Rbm24	NM_001081425	1.8
chromodomain helicase DNA binding protein 4	Chd4	NM_145979	1.8
lymphocyte antigen 9	Ly9	NM_008534	1.8
HEG homolog 1 (zebrafish)	Heg1	NM_175256	1.8
Unknown	LOC638149	XR_033821	1.8
CD14 antigen	Cd14	NM_009841	1.8
ubiquitin carboxyl-terminal esterase L4	Uchl4	NM_033607	1.8
myeloid/lymphoid or mixed-lineage leukemia 1	Mll1	NM_001081049	1.8
Pbx/knotted 1 homeobox	Pknox1	NR_027493	1.8
RAS-like, family 10, member B	Rasl10b	NM_001013386	1.8
syntaxin binding protein 1	Stxbp1	NM_001113569	1.8

Additional Table 2a. Differentially expressed cardiac genes in males compared to females (p ≤0.01) with a regulation of ≥ 1.5

DAVID GeneName	Symbol	Accession	Fold Change Male/Female
E2F transcription factor 1	E2f1	ENSMUST00000000894	1.8
ropporin 1-like	Ropn1l	NM_145852	1.8
Rho GTPase-activating protein	Grit	NM_177379	1.8
lipin 2	Lpin2	NM_022882	1.8
WD repeat domain 37	Wdr37	NM_172445	1.8
insulin-like growth factor I receptor	Igf1r	NM_010513	1.8
DCN1, defective in cullin neddylation 1, domain containing 1 (S. cerevisiae)	Dcun1d1	NM_033623	1.8
Kruppel-like factor 5	Klf5	NM_009769	1.8
CD55 antigen	Cd55	NM_010016	1.8
unc-119 homolog (C. elegans)	Unc119	NM_011676	1.8
SH3 domain binding glutamic acid-rich protein-like 3	Sh3bgrl3	NM_080559	1.8
immunoglobulin superfamily, member 11	Igsf11	NM_170599	1.8
eukaryotic translation initiation factor 5B	Eif5b	NM_198303	1.8
RIKEN cDNA 9330159F19 gene	9330159F19Rik	NM_001162537	1.8
WAP four-disulfide core domain 12	Wfdc12	NM_138684	1.8
branched chain aminotransferase 1, cytosolic	Bcat1	NM_001024468	1.8
EFR3 homolog B (S. cerevisiae)	Efr3b	NM_001082483	1.8
urotensin 2 receptor	Uts2r	NM_145440	1.8
ring finger protein 217	Rnf217	NM_001146349	1.8
paired related homeobox 1	Prrx1	NM_011127	1.8
Unknown	Gm9743	NR_027316	1.8
RIKEN cDNA 1110001D15 gene	Dvwa	NM_026763	1.8
suppressor of cytokine signaling 7	Socs7	NM_138657	1.8
family with sequence similarity 120, member C	Fam120c	NM_198105	1.8
phosphatidylinositol glycan anchor biosynthesis, class G	Pigg	NM_001081234	1.8
solute carrier family 9 (sodium/hydrogen exchanger), member 1	Slc9a1	NM_016981	1.8
predicted gene 10397	Gm10397	ENSMUST00000100823	1.8
Notch gene homolog 4 (Drosophila)	Notch4	NM_010929	1.8
developmental pluripotency associated 1	Dppa1	NM_178247	1.8
myosin, heavy polypeptide 8, skeletal muscle, perinatal	Myh8	NM_177369	1.8
Dicer1, Dcr-1 homolog (Drosophila)	Dicer1	NM_148948	1.8
syndecan 3	Sdc3	NM_011520	1.8
smoothelin-like 2	Smtnl2	NM_177776	1.8
RIKEN cDNA 1110006G14 gene	1110006G14Rik	ENSMUST00000032900	1.8
FCH domain only 1	Fcho1	NM_028715	1.8
RIKEN cDNA 3425401B19 gene	3425401B19Rik	ENSMUST00000096038	1.8
AF4/FMR2 family, member 1	Aff1	NM_001080798	1.8
histocompatibility 2, T region locus 10	H2-T10	NM_010395	1.8
RIKEN cDNA E330021D16 gene	E330021D16Rik	BC099944	1.8
fibulin 1	Fbln1	NM_010180	1.8
BCL2-antagonist/killer 1	Bak1	NM_007523	1.8
protein O-fucosyltransferase 1	Pofut1	NM_080463	1.8
predicted gene 15698	Gm15698	NR_003564	1.8
coactosin-like 1 (Dictyostelium)	Cotl1	NM_028071	1.8
pseudouridine synthase 1	Pus1	NM_001025561	1.8
kinesin family member 2A	Kif2a	NM_008442	1.8
ribosome binding protein 1	Rrbp1	NM_024281	1.8
Unknown	AU042671	ENSMUST00000065641	1.8
predicted gene 5105	Gm5105	NM_177860	1.8
endothelial PAS domain protein 1	Epas1	NM_010137	1.8

Additional Table 2a. Differentially expressed cardiac genes in males compared to females (p ≤0.01) with a regulation of ≥ 1.5

DAVID GeneName	Symbol	Accession	Fold Change Male/Female
calcyon neuron-specific vesicular protein	Caly	BC049663	1.8
peroxisomal biogenesis factor 6	Pex6	NM_145488	1.8
NEDD4 binding protein 1	N4bp1	NM_030563	1.8
homeodomain interacting protein kinase 1	Hipk1	NM_010432	1.8
RIKEN cDNA 9030418K01 gene	9030418K01Rik	NM_001081289	1.8
RIKEN cDNA 1700021K19 gene	1700021K19Rik	BC067390	1.8
FYVE, RhoGEF and PH domain containing 6	Fgd6	NM_053072	1.8
RIKEN cDNA 1190005I06 gene	1190005I06Rik	BC027666	1.8
carcinoembryonic antigen-related cell adhesion molecule 1	Ceacam1	NM_001039185	1.8
heat shock factor 2	Hsf2	NM_008297	1.8
zinc finger protein 239	Zfp239	NM_001001792	1.8
myeloid cell nuclear differentiation antigen	Mnda	NM_001033450	1.8
SRY-box containing gene 12	Sox12	NM_011438	1.8
filamin, beta	Flnb	NM_134080	1.8
tubby-like protein 3	Tulp3	NM_011657	1.8
ski sarcoma viral oncogene homolog (avian)	Ski	NM_011385	1.8
stromal interaction molecule 1	Stim1	NM_009287	1.8
TYRO protein tyrosine kinase binding protein	Tyrobp	NM_011662	1.8
serine/threonine kinase 16	Stk16	NM_011494	1.8
RIKEN cDNA 1700019L03 gene	1700019L03Rik	BC118617	1.8
HtrA serine peptidase 1	Htra1	NM_019564	1.8
acyl-CoA synthetase family member 3	Acsf3	NM_144932	1.8
ribosomal protein L26	Rpl26	NM_009080	1.8
TRAF-interacting protein with forkhead-associated domain	Tifa	NM_145133	1.8
epidermal growth factor-containing fibulin-like extracellular matrix protein 2	Efemp2	NM_021474	1.8
RIKEN cDNA E130311K13 gene	E130311K13Rik	NM_177856	1.8
phospholipid transfer protein	Pltp	NM_011125	1.8
eyes absent 4 homolog (Drosophila)	Eya4	NM_010167	1.8
RIKEN cDNA 1700084C01 gene	1700084C01Rik	BC150846	1.8
actinin, alpha 1	Actn1	NM_134156	1.8
protocadherin 19	Pcdh19	NM_001105245	1.8
GCN1 general control of amino-acid synthesis 1-like 1 (yeast)	Gcn1l1	NM_172719	1.8
aminoacylase 1	Acy1	NM_025371	1.8
claudin 1	Cldn1	NM_016674	1.8
dynamin 1	Dnm1	NM_010065	1.8
Unknown	LOC100039359	ENSMUST00000100834	1.8
calcium/calmodulin-dependent protein kinase kinase 1, alpha	Camkk1	NM_018883	1.8
Dr1 associated protein 1 (negative cofactor 2 alpha)	Drap1	NM_024176	1.8
histocompatibility 2, M region locus 10.3	H2-M10.3	NM_201608	1.8
WW domain binding protein 11	Wbp11	NM_021714	1.8
component of Sp100-rs	Csprs	BC071209	1.8
coiled-coil domain containing 90A	Ccdc90a	NM_001081059	1.7
GTPase, IMAP family member 1	Gimap1	NM_008376	1.7
DEP domain containing 1B	Depdc1b	NM_178683	1.7
solute carrier family 41, member 1	Slc41a1	NM_173865	1.7
Rtf1, Paf1/RNA polymerase II complex component, homolog (S. cerevisiae)	Rtf1	NM_030112	1.7
aldhehyde dehydrogenase family 5, subfamily A1	Aldh5a1	NM_172532	1.7
expressed sequence AU021034	AU021034	BC120488	1.7
La ribonucleoprotein domain family, member 5	Larp5	NM_172585	1.7

Additional Table 2a. Differentially expressed cardiac genes in males compared to females (p ≤0.01) with a regulation of ≥ 1.5

DAVID GeneName	Symbol	Accession	Fold Change Male/Female
NADH dehydrogenase (ubiquinone) 1 alpha subcomplex 11	Ndufa11	NM_027244	1.7
TAO kinase 3	Taok3	ENSMUST00000111978	1.7
pleiomorphic adenoma gene-like 1	Plagl1	NM_009538	1.7
proline rich 12	Prr12	NM_175022	1.7
trinucleotide repeat containing 6C	Tnrc6c	NM_198022	1.7
family with sequence similarity 98, member B	Fam98b	NM_026620	1.7
microtubule associated monoxygenase, calponin and LIM domain containing 2	Mical2	NM_177282	1.7
proprotein convertase subtilisin/kexin type 6	Pcsk6	NM_011048	1.7
LIM domain binding 3	Ldb3	NM_011918	1.7
spermatogenesis associated 7	Spata7	NM_178914	1.7
forkhead box A2	Foxa2	NM_010446	1.7
pogo transposable element with ZNF domain	Pogz	NM_172683	1.7
expressed sequence AI848100	AI848100	BC138379	1.7
multiple inositol polyphosphate histidine phosphatase 1	Minpp1	NM_010799	1.7
retinoblastoma binding protein 5	Rbbp5	NM_172517	1.7
dystrophia myotonica-protein kinase	Dmpk	NM_032418	1.7
secreted acidic cysteine rich glycoprotein	Sparc	NM_009242	1.7
collagen, type II, alpha 1	Col2a1	NM_031163	1.7
expressed sequence AU016765	AU016765	ENSMUST00000097295	1.7
RIKEN cDNA 2900026A02 gene	2900026A02Rik	BC147338	1.7
vascular cell adhesion molecule 1	Vcam1	NM_011693	1.7
Ras and Rab interactor 3	Rin3	NM_177620	1.7
T-box 2	Tbx2	NM_009324	1.7
Rho GTPase activating protein 28	Arhgap28	NM_172964	1.7
glucosamine (N-acetyl)-6-sulfatase	Gns	NM_029364	1.7
neogenin	Neo1	NM_008684	1.7
chromatin modifying protein 2A	Chmp2a	NM_026885	1.7
hyperpolarization-activated, cyclic nucleotide-gated K+ 2	Hcn2	NM_008226	1.7
procollagen-lysine, 2-oxoglutarate 5-dioxygenase 1	Plod1	NM_011122	1.7
A kinase (PRKA) anchor protein 10	Akap10	NM_019921	1.7
pleckstrin homology domain containing, family G (with RhoGef domain) member 2	Plekhg2	NM_138752	1.7
BCL6 interacting corepressor	Bcor	NM_029510	1.7
secretory carrier membrane protein 5	Scamp5	NM_020270	1.7
SR-related CTD-associated factor 1	Scaf1	NM_001008422	1.7
apolipoprotein A-V	Apoa5	NM_080434	1.7
amidohydrolase domain containing 2	Amdhd2	NM_172935	1.7
cathepsin E	Ctse	NM_007799	1.7
frequenin homolog (Drosophila)	Freq	NM_019681	1.7
podocalyxin-like	Podxl	NM_013723	1.7
Unknown	0610037D15Rik	BC022733	1.7
phosphatidylinositol 3-kinase, catalytic, beta polypeptide	Pik3cb	NM_029094	1.7
component of oligomeric golgi complex 1	Cog1	NM_013581	1.7
mitogen-activated protein kinase kinase kinase 7 interacting protein 2	Map3k7ip2	NM_138667	1.7
RIKEN cDNA 1500001M20 gene	1500001M20Rik	NM_026894	1.7
START domain containing 10	Stard10	NM_019990	1.7
tetratricopeptide repeat domain 13	Ttc13	NM_145607	1.7
leucine rich repeat containing 32	Lrrc32	NM_001113379	1.7
paraneoplastic antigen MA2	Pnma2	NM_175498	1.7
cytochrome P450, family 4, subfamily v, polypeptide 3	Cyp4v3	NM_133969	1.7

Additional Table 2a. Differentially expressed cardiac genes in males compared to females (p ≤0.01) with a regulation of ≥ 1.5

DAVID GeneName	Symbol	Accession	Fold Change Male/Female
rhomboid 5 homolog 2 (Drosophila)	Rhbdf2	NM_172572	1.7
actin binding LIM protein family, member 3	Ablim3	NM_198649	1.7
cAMP responsive element binding protein 3-like 2	Creb3l2	NM_178661	1.7
POU domain, class 4, transcription factor 1	Pou4f1	NM_011143	1.7
pleckstrin homology domain containing, family M (with RUN domain) member 1	Plekhm1	NM_183034	1.7
apolipoprotein E	Apoe	NM_009696	1.7
DEAH (Asp-Glu-Ala-His) box polypeptide 37	Dhx37	NM_203319	1.7
special AT-rich sequence binding protein 1	Satb1	NM_009122	1.7
heparan sulfate 6-O-sulfotransferase 1	Hs6st1	NM_015818	1.7
coiled-coil domain containing 97	Ccdc97	NM_028771	1.7
huntingtin interacting protein 1	Hip1	NM_146001	1.7
ST6 (alpha-N-acetyl-neuraminyl-2,3-beta-galactosyl-1,3)-N-acetylgalactosaminide alpha-2,6-sialyltransferase 4	St6galnac4	NM_011373	1.7
jumonji C domain-containing histone demethylase 1 homolog D (S. cerevisiae)	Jhdm1d	NM_001033430	1.7
alanyl (membrane) aminopeptidase	Anpep	NM_008486	1.7
solute carrier family 6 (neurotransmitter transporter, noradrenalin), member 2	Slc6a2	NM_009209	1.7
predicted gene 128	Gm128	BC092540	1.7
gasdermin C-like, pseudogene	Gsdmcl-ps	AK016931	1.7
vacuolar protein sorting 4b (yeast)	Vps4b	NM_009190	1.7
family with sequence similarity 102, member B	Fam102b	ENSMUST00000046924	1.7
mitogen-activated protein kinase-activated protein kinase 3	Mapkapk3	NM_178907	1.7
cofilin 1, non-muscle	Cfl1	NM_007687	1.7
dystrotelin	Dytn	NM_001081658	1.7
zinc finger protein 784	Zfp784	NM_001039532	1.7
ArfGAP with RhoGAP domain, ankyrin repeat and PH domain 3	Arap3	NM_139206	1.7
MKL/myocardin-like 2	Mkl2	NM_153588	1.7
THAP domain containing, apoptosis associated protein 1	Thap1	NM_199042	1.7
glutamine fructose-6-phosphate transaminase 2	Gfpt2	NM_013529	1.7
zinc finger, DHHC domain containing 18	Zdhhc18	NM_001017968	1.7
REX1, RNA exonuclease 1 homolog (S. cerevisiae)	Rexo1	NM_025852	1.7
ATPase, H+ transporting, lysosomal V0 subunit D1	Atp6v0d1	NM_013477	1.7
meningioma 1	Mn1	NM_001081235	1.7
histone deacetylase 7	Hdac7	NM_019572	1.7
GATA binding protein 4	Gata4	NM_008092	1.7
male enhanced antigen 1	Mea1	NM_010787	1.7
RIKEN cDNA 4732418C07 gene	4732418C07Rik	BC059213	1.7
dihydrofolate reductase	Dhfr	NM_010049	1.7
AP1 gamma subunit binding protein 1	Synrg	NM_001115009	1.7
zinc finger protein 106	Zfp106	NM_011743	1.7
family with sequence similarity 108, member C	Fam108c	NM_133722	1.7
ataxin 2-like	Atxn2l	NM_183020	1.7
schlafen 10	Slfn10	NM_181542	1.7
programmed cell death 6 interacting protein	Pdcd6ip	NM_011052	1.7
arrestin, beta 2	Arrb2	NM_145429	1.7
potassium channel tetramerisation domain containing 11	Kctd11	NM_153143	1.7
splA/ryanodine receptor domain and SOCS box containing 1	Spsb1	NM_029035	1.7
Unknown	Atn1	NM_007881	1.7
pericentriolar material 1	Pcm1	NM_023662	1.7
discoidin, CUB and LCCL domain containing 2	Dcbld2	NM_028523	1.7

Additional Table 2a. Differentially expressed cardiac genes in males compared to females (p ≤0.01) with a regulation of ≥ 1.5

DAVID GeneName	Symbol	Accession	Fold Change Male/Female
family with sequence similarity 62 (C2 domain containing), member B	Esyt2	NM_028731	1.7
zinc finger and BTB domain containing 7C	Zbtb7c	NM_145356	1.7
adaptor-related protein complex 2, sigma 1 subunit	Ap2s1	NM_198613	1.7
two pore segment channel 2	Tpcn2	NM_146206	1.7
transcription termination factor, RNA polymerase II	Ttf2	NM_001013026	1.7
sex comb on midleg homolog 1	Scmh1	NM_013883	1.7
tuberous sclerosis 2	Tsc2	NM_011647	1.7
glutamine-rich 1	Qrich1	NM_001114119	1.7
G protein-coupled receptor 153	Gpr153	NM_178406	1.7
MYST histone acetyltransferase (monocytic leukemia) 3	Myst3	NM_001081149	1.7
BTB (POZ) domain containing 3	Btbd3	NM_145534	1.7
hexosaminidase A	Hexa	NM_010421	1.7
cDNA sequence BC019943	BC019943	NM_001112729	1.7
whirlin	Whrn	NM_028640	1.7
mitchondrial ribosomal protein S7	Mrps7	NM_025305	1.7
katanin p60 (ATPase-containing) subunit A1	Katna1	NM_011835	1.7
BCL2-interacting killer	Bik	NM_007546	1.7
AT rich interactive domain 1A (SWI-like)	Arid1a	NM_001080819	1.7
RGM domain family, member A	Rgma	NM_177740	1.7
polymerase (RNA) I polypeptide E	Polr1e	NM_022811	1.7
TSC22 domain family, member 2	Tsc22d2	NM_001081229	1.7
nuclear factor I/X	Nfix	NM_001081982	1.7
membrane-spanning 4-domains, subfamily A, member 4C	Ms4a4c	NM_029499	1.7
protein phosphatase 2 (formerly 2A), regulatory subunit B (PR 52), alpha isoform	Ppp2r2a	NM_028032	1.7
zinc finger protein 821	Zfp821	NM_029468	1.7
Unknown	Lba1	BC086653	1.7
zinc finger protein 597	Zfp597	NM_001033159	1.7
RIKEN cDNA E130309D14 gene	E130309D14Rik	BC150886	1.7
predicted gene 10524	Gm10524	ENSMUST00000097503	1.7
thrombospondin, type I, domain containing 4	Thsd4	NM_001040426	1.7
RIO kinase 1 (yeast)	Riok1	NM_024242	1.7
SET binding protein 1	Setbp1	NM_053099	1.7
UDP-N-acetyl-alpha-D-galactosamine:polypeptide N-acetylgalactosaminyltransferase 10	Galnt10	NM_134189	1.7
nuclear transcription factor, X-box binding-like 1	Nfxl1	NM_133921	1.7
matrix metallopeptidase 23	Mmp23	NM_011985	1.7
netrin 4	Ntn4	NM_021320	1.7
elongation factor RNA polymerase II 2	Ell2	NM_138953	1.7
RIKEN cDNA 1700007K09 gene	1700007K09Rik	NM_027037	1.7
echinoderm microtubule associated protein like 1	Eml1	NM_001043335	1.7
growth factor receptor bound protein 14	Grb14	NM_016719	1.7
huntingtin interacting protein 1 related	Hip1r	NM_145070	1.7
sushi domain containing 2	Susd2	NM_027890	1.7
Unknown	40426	NM_213614	1.7
potassium channel tetramerisation domain containing 17	Kctd17	NM_001081367	1.7
SH3 and PX domains 2A	Sh3pxd2a	NM_008018	1.7
RNA polymerase II associated protein 3	Gm5697	XR_004787	1.7
TNFRSF1A-associated via death domain	Tradd	NM_001033161	1.7
potassium channel, subfamily K, member 1	Kcnk1	NM_008430	1.7
lysosomal-associated membrane protein 1	Lamp1	NM_010684	1.7

Additional Table 2a. Differentially expressed cardiac genes in males compared to females (p ≤0.01) with a regulation of ≥ 1.5

DAVID GeneName	Symbol	Accession	Fold Change Male/Female
protein tyrosine phosphatase, non-receptor type 9	Ptpn9	NM_019651	1.7
mitochondrial ribosomal protein S18A	Mrps18a	NM_026768	1.7
pleckstrin homology domain containing, family M (with RUN domain) member 2	Plekhm2	NM_001033150	1.7
fibulin 2	Fbln2	NM_007992	1.7
selectin, endothelial cell	Sele	NM_011345	1.7
ribosomal protein L36	Rpl36	NM_018730	1.7
multiple EGF-like-domains 11	Megf11	NM_172522	1.7
SMG1 homolog, phosphatidylinositol 3-kinase-related kinase (C. elegans)	Smg1	NM_001031814	1.7
neuronal pentraxin 1	Nptx1	NM_008730	1.7
Sec31 homolog B (S. cerevisiae)	Sec31b	NM_001033343	1.7
fibroblast growth factor receptor 3	Fgfr3	NM_008010	1.7
ADAMTS-like 5	Adamtsl5	NM_001113548	1.7
sal-like 4 (Drosophila)	Sall4	NM_175303	1.7
hemopoietic cell kinase	Hck	NM_010407	1.7
guanylate kinase 1	Guk1	NM_008193	1.7
formin homology 2 domain containing 3	Fhod3	NM_175276	1.7
src homology 2 domain-containing transforming protein B	Shb	NM_001033306	1.7
progestin and adipoQ receptor family member IX	Paqr9	NM_198414	1.7
four and a half LIM domains 3	Fhl3	NM_010213	1.7
rhomboid family 1 (Drosophila)	Rhbdf1	NM_010117	1.7
Braf transforming gene	Braf	NM_139294	1.7
mitochondrial ribosomal protein L28	Mrpl28	NM_024227	1.7
mannosyl-oligosaccharide glucosidase	Mogs	NM_020619	1.7
aarF domain containing kinase 1	Adck1	NM_028105	1.7
grainyhead-like 3 (Drosophila)	Grhl3	NM_001013756	1.7
phosphofurin acidic cluster sorting protein 2	Pacs2	NM_001081170	1.7
protein kinase C, alpha	Prkca	NM_011101	1.7
predicted gene 5149	Gm5149	ENSMUST00000092878	1.7
dishevelled 3, dsh homolog (Drosophila)	Dvl3	NM_007889	1.7
lysocardiolipin acyltransferase 1	Lclat1	NM_001081071	1.7
Yip1 interacting factor homolog B (S. cerevisiae)	Yif1b	NM_029887	1.7
RIKEN cDNA 9030624J02 gene	9030624J02Rik	BC043674	1.7
transmembrane protein 127	Tmem127	NM_175145	1.7
predicted gene 1614	Gm1614	ENSMUST00000097619	1.7
MAD homolog 1 (Drosophila)	Smad1	NM_008539	1.7
kelch domain containing 2	Klhdc2	NM_027117	1.7
ubiquitin specific peptidase 31	Usp31	NM_001033173	1.7
secretin	Sct	NM_011328	1.7
myeloid/lymphoid or mixed-lineage leukemia (trithorax homolog, Drosophila)	Mllt3	NM_027326	1.7
Rac/Cdc42 guanine nucleotide exchange factor (GEF) 6	Arhgef6	NM_152801	1.7
RALBP1 associated Eps domain containing protein 2	Reps2	NM_178256	1.7
SET domain containing 6	Setd6	NM_001035123	1.7
biorientation of chromosomes in cell division 1-like	Bod1l	NM_001081422	1.7
glycosyltransferase 25 domain containing 1	Glt25d1	NM_146211	1.7
complement component 5a receptor 1	C5ar1	NM_007577	1.7
ephrin B3	Efnb3	NM_007911	1.7
Rho guanine nucleotide exchange factor (GEF) 15	Arhgef15	NM_177566	1.7
family with sequence similarity 78, member A	Fam78a	BC137951	1.7

Additional Table 2a. Differentially expressed cardiac genes in males compared to females (p ≤0.01) with a regulation of ≥ 1.5

DAVID GeneName	Symbol	Accession	Fold Change Male/Female
retinoic acid induced 1	Rai1	NM_009021	1.7
H1 histone family, member X	H1fx	NM_198622	1.7
myosin X	Myo10	NM_019472	1.7
ribonuclease P/MRP 30 subunit (human)	Rpp30	NM_019428	1.7
surfactant associated protein B	Sftpb	NM_147779	1.7
kinesin family member 21A	Kif21a	NM_001109040	1.7
structural maintenance of chromosomes 4	Smc4	NM_133786	1.7
cDNA sequence BC017647	BC017647	NM_145430	1.7
protocadherin 1	Pcdh1	NM_029357	1.7
upstream binding protein 1	Ubp1	NM_001083319	1.7
family with sequence similarity 13, member B	Fam13b	BC031465	1.7
Fc receptor, IgE, high affinity I, gamma polypeptide	Fcer1g	NM_010185	1.7
centrosomal protein 350	Cep350	BC089561	1.7
RIKEN cDNA 4930402H24 gene	4930402H24Rik	BC052447	1.7
nuclear receptor subfamily 1, group D, member 1	Nr1d1	NM_145434	1.7
angiomotin-like 1	Amotl1	NM_001081395	1.7
WW domain binding protein 2	Wbp2	NM_016852	1.7
RIKEN cDNA 6430526N21 gene	6430526N21Rik	NM_001033383	1.7
myeloid/lymphoid or mixed-lineage leukemia (trithorax homolog, Drosophila); translocated to, 6	Mllt6	NM_139311	1.7
multiple EGF-like-domains 6	Megf6	NM_001162977	1.7
nudix (nucleoside diphosphate linked moiety X)-type motif 6	Nudt6	NM_153561	1.7
UPF3 regulator of nonsense transcripts homolog A (yeast)	Upf3a	NM_025924	1.7
protein kinase C, epsilon	Prkce	NM_011104	1.7
coiled-coil domain containing 50	Ccdc50	NM_026202	1.7
RecQ protein-like 4	Recql4	NM_058214	1.7
filamin binding LIM protein 1	Fblim1	NM_133754	1.7
ATPase family, AAA domain containing 3A	Atad3a	NM_179203	1.7
Sjogren's syndrome nuclear autoantigen 1	Ssna1	NM_023464	1.7
G protein-coupled receptor 151	Gpr151	NM_181543	1.7
natriuretic peptide receptor 2	Npr2	NM_173788	1.7
Pbx/knotted 1 homeobox 2	Pknox2	NM_148950	1.7
mitochondria-associated protein involved in granulocyte-macrophage colony-s	Magmas	NM_025571	1.7
RIKEN cDNA A730018C14 gene	A730018C14Rik	ENSMUST00000050754	1.7
neural precursor cell expressed, developmentally down-regulated gene 1	Nedd1	NM_008682	1.7
protein inhibitor of activated STAT 3	Pias3	NM_146135	1.7
splA/ryanodine receptor domain and SOCS box containing 4	Spsb4	NM_145134	1.7
RNA binding motif, single stranded interacting protein 2	Rbms2	NM_019711	1.7
large tumor suppressor 2	Lats2	NM_015771	1.7
TBC1 domain family, member 9B	Tbc1d9b	NM_029745	1.7
solute carrier organic anion transporter family, member 5A1	Slco5a1	NM_172841	1.7
DEAH (Asp-Glu-Ala-His) box polypeptide 33	Dhx33	NM_178367	1.7
xylosyltransferase II	Xylt2	NM_145828	1.7
castor homolog 1, zinc finger (Drosophila)	Casz1	NM_001159344	1.7
low density lipoprotein receptor-related protein 1	Lrp1	NM_008512	1.7
AT hook, DNA binding motif, containing 1	Ahdc1	NM_146155	1.7
odd Oz/ten-m homolog 3 (Drosophila)	Odz3	NM_011857	1.7
activating signal cointegrator 1 complex subunit 1	Ascc1	NM_026937	1.7
ubiquitin-conjugating enzyme E2R 2	Ube2r2	NM_026275	1.7
platelet derived growth factor, alpha	Pdgfa	NM_008808	1.7

Additional Table 2a. Differentially expressed cardiac genes in males compared to females (p ≤0.01) with a regulation of ≥ 1.5

DAVID GeneName	Symbol	Accession	Fold Change Male/Female
myosin XVIIIA	Myo18a	ENSMUST00000108375	1.7
RIKEN cDNA 4933408B17 gene	4933408B17Rik	NM_177773	1.7
oxysterol binding protein	Osbp	NM_001033174	1.7
Unknown	BC037112	BC099925	1.7
heat shock protein 12B	Hspa12b	NM_028306	1.7
LysM, putative peptidoglycan-binding, domain containing 2	Lysmd2	NM_027309	1.7
poly (ADP-ribose) polymerase family, member 3	Parp3	NM_145619	1.7
malic enzyme 1, NADP(+)-dependent, cytosolic	Me1	NM_008615	1.7
SH3 domain containing ring finger 1	Sh3rf1	NM_021506	1.7
Rho guanine nucleotide exchange factor (GEF) 10	Arhgef10	NM_172751	1.7
paroxysmal nonkinesiogenic dyskinesia	Pnkd	NM_025580	1.6
coiled-coil domain containing 134	Ccdc134	NM_172428	1.6
sphingosine-1-phosphate receptor 3	S1pr3	NM_010101	1.6
EH domain binding protein 1-like 1	Ehbp1l1	NM_001114597	1.6
widely-interspaced zinc finger motifs	Wiz	NM_212438	1.6
protein phosphatase 1, catalytic subunit, alpha isoform	Ppp1ca	NM_031868	1.6
DnaJ (Hsp40) homolog, subfamily B, member 14	Dnajb14	NM_001033155	1.6
RIKEN cDNA 2810474O19 gene	2810474O19Rik	NM_026054	1.6
major facilitator superfamily domain containing 10	Mfsd10	NM_026660	1.6
v-abl Abelson murine leukemia viral oncogene homolog 2 (arg, Abelson-related gene)	Abl2	NM_001136104	1.6
anoctamin 1, calcium activated chloride channel	Ano1	NM_178642	1.6
retinoblastoma binding protein 4	Rbbp4	NM_009030	1.6
SLIT-ROBO Rho GTPase activating protein 2	Srgap2	NM_001081011	1.6
zinc finger protein 202	Zfp202	NM_030713	1.6
discs, large homolog-associated protein 4 (Drosophila)	Dlgap4	NM_146128	1.6
dedicator of cytokinesis 7	Dock7	NM_026082	1.6
par-3 (partitioning defective 3) homolog (C. elegans)	Pard3	NM_001013581	1.6
ankyrin repeat domain 28	Ankrd28	NM_001024604	1.6
zinc finger and BTB domain containing 1	Zbtb1	NM_178744	1.6
integrin alpha 5 (fibronectin receptor alpha)	Itga5	NM_010577	1.6
family with sequence similarity 109, member A	Fam109a	NM_175474	1.6
mitogen-activated protein kinase kinase kinase 4	Map3k4	NM_011948	1.6
cytochrome b-561	Cyb561	NM_007805	1.6
zinc finger with KRAB and SCAN domains 1	Zkscan1	NM_133906	1.6
2'-5' oligoadenylate synthetase 2	Oas2	NM_145227	1.6
G protein-coupled receptor kinase 6	Grk6	NM_001038018	1.6
mitogen-activated protein kinase kinase kinase 1	Map3k1	NM_011945	1.6
per-hexamer repeat gene 4	Phxr4	X12806	1.6
deformed epidermal autoregulatory factor 1 (Drosophila)	Deaf1	NR_027769	1.6
odd Oz/ten-m homolog 2 (Drosophila)	Odz2	NM_011856	1.6
Unknown	Gm5068	XM_204772	1.6
poly (ADP-ribose) polymerase family, member 8	Parp8	NM_001081009	1.6
epidermal growth factor receptor pathway substrate 15	Eps15	NM_007943	1.6
transmembrane protein 110	Tmem110	NM_028839	1.6
L antigen family, member 3	Lage3	NM_025410	1.6
RAB, member of RAS oncogene family-like 4	Rabl4	NM_025931	1.6
Cnksr family member 3	Cnksr3	NM_172546	1.6
ankyrin repeat and SOCS box-containing 7	Asb7	NR_003961	1.6
predicted gene 9881	Gm9881	ENSMUST00000068783	1.6
cerebral cavernous malformation 2 homolog (human)	Ccm2	NM_146014	1.6

Additional Table 2a. Differentially expressed cardiac genes in males compared to females (p ≤0.01) with a regulation of ≥ 1.5

DAVID GeneName	Symbol	Accession	Fold Change Male/Female
fibrosin	Fbrs	NM_010183	1.6
SWI/SNF related, matrix associated, actin dependent regulator of chromatin, subfamily d, member 3	Smarcd3	NM_025891	1.6
DNA segment, Chr 18, ERATO Doi 653, expressed	D18Ertd653e	BC096371	1.6
phospholipase A2, group XIIB	Pla2g12b	NM_023530	1.6
zinc finger E-box binding homeobox 1	Zeb1	NM_011546	1.6
inhibitor of growth family, member 4	Ing4	NM_133345	1.6
xin actin-binding repeat containing 1	Xirp1	NM_001081339	1.6
v-crk sarcoma virus CT10 oncogene homolog (avian)-like	Crkl	NM_007764	1.6
taspase, threonine aspartase 1	Tasp1	NM_175225	1.6
PFTAIRE protein kinase 1	Pftk1	NM_011074	1.6
RIKEN cDNA 3200002M19 gene	3200002M19Rik	BC006736	1.6
activity-dependent neuroprotective protein	Adnp	NM_009628	1.6
protein phosphatase 2, regulatory subunit B (B56), delta isoform	Ppp2r5d	NM_009358	1.6
BCL2-associated athanogene 2	Bag2	NM_145392	1.6
patatin-like phospholipase domain containing 7	Pnpla7	NM_146251	1.6
centrosomal protein 68	Cep68	NM_172260	1.6
PHD finger protein 19	Phf19	NM_028716	1.6
reticulon 4	Rtn4	NM_194054	1.6
pumilio 1 (Drosophila)	Pum1	NM_030722	1.6
RIKEN cDNA 1300018J18 gene	1300018J18Rik	NM_027905	1.6
E1A binding protein p300	Ep300	NM_177821	1.6
copine III	Cpne3	NM_027769	1.6
CDC2-related kinase, arginine/serine-rich	Crkrs	NM_001109626	1.6
zinc finger protein 628	Zfp628	NM_170759	1.6
transketolase	Tkt	NM_009388	1.6
PX domain containing serine/threonine kinase	Pxk	NM_178279	1.6
coronin, actin binding protein 1B	Coro1b	NM_011778	1.6
RIKEN cDNA 2810428I15 gene	2810428I15Rik	BC028495	1.6
THAP domain containing 4	Thap4	NM_025920	1.6
splicing factor 1	Sf1	NM_001110791	1.6
death associated protein kinase 1	Dapk1	NM_029653	1.6
protein phosphatase 1, regulatory subunit 9B	Ppp1r9b	NM_172261	1.6
Ras association (RalGDS/AF-6) domain family member 3	Rassf3	NM_138956	1.6
polymerase (RNA) mitochondrial (DNA directed)	Polrmt	NM_172551	1.6
protein arginine methyltransferase 10 (putative)	Prmt10	NM_001081240	1.6
phospholipase C, delta 1	Plcd1	NM_019676	1.6
excision repair cross-complementing rodent repair deficiency, complementation group 6	Ercc6	NM_001081221	1.6
leucine rich repeat containing 4	Lrrc4	NM_138682	1.6
pleckstrin homology domain containing, family H (with MyTH4 domain) member 3	Plekhh3	NM_146030	1.6
feminization 1 homolog b (C. elegans)	Fem1b	NM_010193	1.6
transportin 2 (importin 3, karyopherin beta 2b)	Tnpo2	NM_145390	1.6
RIKEN cDNA 2310040G07 gene	2310040G07Rik	ENSMUST00000101090	1.6
hemicentin 2	Hmcn2	ENSMUST00000074390	1.6
laminin, gamma 1	Lamc1	NM_010683	1.6
NADH dehydrogenase (ubiquinone) 1 beta subcomplex, 10	Ndufb10	NM_026684	1.6
eukaryotic translation initiation factor 5A2	Eif5a2	NM_177586	1.6
SHANK-associated RH domain interacting protein	Sharpin	NM_025340	1.6
RIKEN cDNA 9930021J03 gene	9930021J03Rik	AK220440	1.6

Additional Table 2a. Differentially expressed cardiac genes in males compared to females (p ≤0.01) with a regulation of ≥ 1.5

DAVID GeneName	Symbol	Accession	Fold Change Male/Female
nuclear factor of kappa light polypeptide gene enhancer in B-cells 1, p105	Nfkb1	NM_008689	1.6
serine/threonine kinase 38	Stk38	NM_134115	1.6
SET and MYND domain containing 2	Smyd2	NM_026796	1.6
leiomodin 3 (fetal)	Lmod3	NM_001081157	1.6
slingshot homolog 3 (Drosophila)	Ssh3	NM_198113	1.6
inositol 1,4,5-triphosphate receptor 2	Itpr2	NM_019923	1.6
mutS homolog 3 (E. coli)	Msh3	NM_010829	1.6
folliculin interacting protein 2	Fnip2	NM_001162999	1.6
G protein-coupled receptor 161	Gpr161	NM_001081126	1.6
RIKEN cDNA B230380D07 gene	B230380D07Rik	NM_172772	1.6
death-associated protein	Dap	NM_146057	1.6
atonal homolog 8 (Drosophila)	Atoh8	NM_153778	1.6
alanine-glyoxylate aminotransferase 2-like 2	Agxt2l2	NM_028398	1.6
RNA binding motif, single stranded interacting protein 1	Rbms1	NM_001141932	1.6
tyrosyl-tRNA synthetase 2 (mitochondrial)	Yars2	NM_198246	1.6
RIKEN cDNA A830039N20 gene	A830039N20Rik	BC038501	1.6
mitochondrial ribosomal protein L11	Mrpl11	NM_025553	1.6
Unknown	Gm10515	ENSMUST00000097433	1.6
STEAP family member 3	Steap3	NM_001085409	1.6
neural precursor cell expressed, developmentally down-regulated gene 4-like	Nedd4l	NM_001114386	1.6
collagen, type III, alpha 1	Col3a1	NM_009930	1.6
membrane-associated ring finger (C3HC4) 5	40242	NM_027314	1.6
apoptosis-inducing, TAF9-like domain 1	Apitd1	NM_027263	1.6
cysteine rich transmembrane BMP regulator 1 (chordin like)	Crim1	NM_015800	1.6
myosin ID	Myo1d	NM_177390	1.6
ST3 beta-galactoside alpha-2,3-sialyltransferase 3	St3gal3	NM_009176	1.6
RIKEN cDNA E130306D19 gene	E130306D19Rik	NM_001013377	1.6
predicted gene 5142	Gm5142	NM_001004158	1.6
Unknown	Akap2	NM_001035533	1.6
RIKEN cDNA 4933407C03 gene	4933407C03Rik	BC158118	1.6
leucine-rich PPR-motif containing	Lrpprc	NM_028233	1.6
zinc finger, MYND domain containing 19	Zmynd19	NM_026021	1.6
potassium inwardly-rectifying channel, subfamily J, member 9	Kcnj9	NM_008429	1.6
tetraspanin 5	Tspan5	NM_019571	1.6
eukaryotic translation elongation factor 1 gamma	Eef1g	NM_026007	1.6
RIKEN cDNA C030044B11 gene	C030044B11Rik	NR_015601	1.6
von Willebrand factor A domain containing 3A	Vwa3a	NM_177697	1.6
ATP-binding cassette, sub-family B (MDR/TAP), member 4	Abcb4	NM_008830	1.6
Unknown	LOC674623	XR_031213	1.6
KRI1 homolog (S. cerevisiae)	Kri1	NM_145416	1.6
leucine-rich repeats and calponin homology (CH) domain containing 1	Lrch1	NM_001033439	1.6
bone morphogenetic protein 1	Bmp1	NM_009755	1.6
ligase III, DNA, ATP-dependent	Lig3	NM_010716	1.6
Rho GTPase activating protein 18	Arhgap18	NM_176837	1.6
DAZ associated protein 1	Dazap1	NM_133188	1.6
apolipoprotein B mRNA editing enzyme, catalytic polypeptide 3	Apobec3	NM_001160415	1.6
parathymosin	Ptms	NM_026988	1.6
mitogen-activated protein kinase 8 interacting protein 3	Mapk8ip3	NM_013931	1.6
nuclear receptor subfamily 2, group F, member 2	Nr2f2	NM_009697	1.6

Additional Table 2a. Differentially expressed cardiac genes in males compared to females (p ≤0.01) with a regulation of ≥ 1.5

DAVID GeneName	Symbol	Accession	Fold Change Male/Female
RIKEN cDNA 2410131K14 gene	2410131K14Rik	NM_001081236	1.6
transducin-like enhancer of split 3, homolog of Drosophila E(spl)	Tle3	NM_001083927	1.6
RIKEN cDNA B230312A22 gene	B230312A22Rik	BC046771	1.6
transforming growth factor, beta 3	Tgfb3	NM_009368	1.6
catenin (cadherin associated protein), delta 1	Ctnnd1	NM_007615	1.6
RIKEN cDNA 1110032A13 gene	1110032A13Rik	BC064066	1.6
zinc finger protein 687	Zfp687	NM_030074	1.6
U7 small nuclear RNA	Rnu7	NR_024201	1.6
H6 homeo box 1	Hmx1	NM_010445	1.6
glial cell line derived neurotrophic factor family receptor alpha 4	Gfra4	ENSMUST00000110235	1.6
ring finger protein 44	Rnf44	NR_027395	1.6
neurolysin (metallopeptidase M3 family)	Nln	NM_029447	1.6
UBX domain protein 7	Ubxn7	NM_177633	1.6
solute carrier family 22 (organic anion transporter), member 8	Slc22a8	NM_031194	1.6
metastasis suppressor 1-like	Mtss1l	NM_198625	1.6
SET domain containing 5	Setd5	NM_028385	1.6
LanC (bacterial lantibiotic synthetase component C)-like 2	Lancl2	NM_133737	1.6
bone marrow stromal cell antigen 1	Bst1	NM_009763	1.6
wingless related MMTV integration site 10b	Wnt10b	NM_011718	1.6
deoxyuridine triphosphatase	Dut	NM_001159646	1.6
extraembryonic, spermatogenesis, homeobox 1	Esx1	NM_007957	1.6
mesoderm posterior 1	Mesp1	NM_008588	1.6
WAS/WASL interacting protein family, member 3	Wipf3	BC044880	1.6
coiled-coil domain containing 34	Ccdc34	NM_026613	1.6
calcium homeostasis endoplasmic reticulum protein	Cherp	NM_138585	1.6
protein tyrosine phosphatase, non-receptor type 3	Ptpn3	NM_011207	1.6
latent transforming growth factor beta binding protein 2	Ltbp2	NM_013589	1.6
RIKEN cDNA 4930432M17 gene	4930432M17Rik	ENSMUST00000098646	1.6
hexose-6-phosphate dehydrogenase (glucose 1-dehydrogenase)	H6pd	NM_173371	1.6
glutamate-cysteine ligase, catalytic subunit	Gclc	NM_010295	1.6
nucleoporin 205	Nup205	NM_027513	1.6
fibroblast growth factor receptor-like 1	Fgfrl1	NM_054071	1.6
AP2 associated kinase 1	Aak1	NM_001040106	1.6
kyphoscoliosis peptidase	Ky	NM_024291	1.6
plastin 1 (I-isoform)	Pls1	NM_001033210	1.6
RIKEN cDNA 4922501C03 gene	4922501C03Rik	NM_199316	1.6
ataxin 7-like 1	Atxn7l1	NM_001033436	1.6
transmembrane protein 149	Tmem149	NM_145580	1.6
coiled-coil domain containing 92	Ccdc92	NM_144819	1.6
zinc finger, MYND domain containing 11	Zmynd11	NM_144516	1.6
serine/threonine kinase 35	Stk35	NM_183262	1.6
CUE domain containing 1	Cuedc1	NM_198013	1.6
zinc finger, matrin-like	Zfml	NM_008717	1.6
glutamate receptor, ionotropic, N-methyl D-aspartate-like 1A	Grinl1a	NM_178602	1.6
gamma-aminobutyric acid (GABA) B receptor, 1	Gabbr1	NM_019439	1.6
RIKEN cDNA 1810031K17 gene	1810031K17Rik	BC028815	1.6
striatin, calmodulin binding protein 4	Strn4	NM_133789	1.6
tetratricopeptide repeat domain 17	Ttc17	NM_183106	1.6
within bgcn homolog (Drosophila)	Wibg	BC049647	1.6
amine oxidase, copper containing 3	Aoc3	NM_009675	1.6

Additional Table 2a. Differentially expressed cardiac genes in males compared to females (p ≤0.01) with a regulation of ≥ 1.5

DAVID GeneName	Symbol	Accession	Fold Change Male/Female
interleukin 6 signal transducer	Il6st	NM_010560	1.6
spire homolog 1 (Drosophila)	Spire1	NM_194355	1.6
solute carrier family 36 (proton/amino acid symporter), member 4	Slc36a4	NM_172289	1.6
nucleoporin like 1	Nupl1	NM_170591	1.6
zinc finger with KRAB and SCAN domains 17	Zkscan17	NM_172941	1.6
two pore channel 1	Tpcn1	NM_145853	1.6
apelin receptor	Aplnr	NM_011784	1.6
ubiquitin protein ligase E3 component n-recognin 2	Ubr2	NM_146078	1.6
ras responsive element binding protein 1	Rreb1	NM_001013392	1.6
protocadherin gamma subfamily A, 5	Pcdhga5	NM_033588	1.6
BEN domain containing 3	Bend3	NM_199028	1.6
laminin B1 subunit 1	Lamb1-1	NM_008482	1.6
nucleobindin 1	Nucb1	NM_008749	1.6
sorting nexin family member 21	Snx21	NM_133924	1.6
Vcalcineurin-like phosphoesterase domain containing 1	Cpped1	NM_146067	1.6
calreticulin	Calr	NM_007591	1.6
dual specificity phosphatase 5	Dusp5	NM_001085390	1.6
Notch gene homolog 1 (Drosophila)	Notch1	NM_008714	1.6
xin actin-binding repeat containing 2	Xirp2	NM_001024618	1.6
nuclear receptor coactivator 6	Ncoa6	NM_019825	1.6
preproenkephalin	Penk	NM_001002927	1.6
RIKEN cDNA 1110018G07 gene	1110018G07Rik	NM_178065	1.6
leucine rich repeat (in FLII) interacting protein 2	Lrrfip2	NM_027742	1.6
uracil DNA glycosylase	Ung	NM_001040691	1.6
vacuolar protein sorting 39 (yeast)	Vps39	NR_027618	1.6
HLA-B associated transcript 2	Bat2	NM_020027	1.6
FAST kinase domains 5	Fastkd5	NM_198176	1.6
mitochondrial ribosomal protein S34	Mrps34	NM_023260	1.6
zinc finger protein 275	Zfp275	NM_031494	1.6
cadherin 2	Cdh2	NM_007664	1.6
regulator of calcineurin 1	Rcan1	NM_001081549	1.6
microtubule-associated protein 4	Mtap4	NM_008633	1.6
epidermal growth factor receptor	Egfr	NM_207655	1.6
protein kinase, cAMP dependent regulatory, type II alpha	Prkar2a	NM_008924	1.6
zinc finger protein 318	Zfp318	NM_207671	1.6
ring finger protein 25	Rnf25	NM_021313	1.6
apoptotic chromatin condensation inducer 1	Acin1	NR_024029	1.6
orthopedia homolog (Drosophila)	Otp	NM_011021	1.6
excision repair cross-complementing rodent repair deficiency, complementation group 1	Ercc1	NM_007948	1.6
RIKEN cDNA E130309D02 gene	E130309D02Rik	NM_172726	1.6
zinc fingr protein 551	Zfp551	NM_001033820	1.6
tubulin tyrosine ligase	Ttl	NM_027192	1.6
family with sequence similarity 155, member A	Fam155a	NM_173446	1.6
5',3'-nucleotidase, mitochondrial	Nt5m	NM_134029	1.6
autophagy-related 9A (yeast)	Atg9a	NM_001003917	1.6
PCTAIRE-motif protein kinase 3	Pctk3	NM_008795	1.6
active BCR-related gene	Abr	NM_198018	1.6
DEAD (Asp-Glu-Ala-Asp) box polypeptide 28	Ddx28	NM_028038	1.6
protocadherin beta 14	Pcdhb14	NM_053139	1.6
histone deacetylase 4	Hdac4	NM_207225	1.6

Additional Table 2a. Differentially expressed cardiac genes in males compared to females (p ≤0.01) with a regulation of ≥ 1.5

DAVID GeneName	Symbol	Accession	Fold Change Male/Female
RIKEN cDNA 1700034J05 gene	1700034J05Rik	ENSMUST00000036592	1.6
zinc finger, MYM-type 6	Zmym6	NM_177462	1.6
annexin A7	Anxa7	NM_009674	1.6
growth arrest specific 2	Gas2	NM_008087	1.6
heat shock 105kDa/110kDa protein 1	Hsph1	NM_013559	1.6
solute carrier family 7, member 6 opposite strand	Slc7a6os	NM_001007567	1.6
neuro-oncological ventral antigen 1	Nova1	ENSMUST00000021438	1.6
misshapen-like kinase 1 (zebrafish)	Mink1	NM_016713	1.6
mitogen-activated protein kinase kinase kinase kinase 2	Map4k2	NM_009006	1.6
PHD finger protein 21A	Phf21a	NM_138755	1.6
ubiquitin-like 7 (bone marrow stromal cell-derived)	Ubl7	NM_001122873	1.6
ZXD family zinc finger C	Zxdc	NM_030260	1.6
coiled-coil and C2 domain containing 2B	Cc2d2b	XM_994191	1.6
glucosidase beta 2	Gba2	NM_172692	1.6
centrosome and spindle pole associated protein 1	Cspp1	NM_026493	1.6
plexin A4	Plxna4	NM_175750	1.6
LPS-responsive beige-like anchor	Lrba	NM_030695	1.6
HEAT repeat containing 6	Heatr6	NM_145432	1.6
WD repeat domain 43	2610029G23Rik	BC035042	1.6
insulin-like growth factor 1	Igf1	NM_010512	1.6
transcription factor 3	Tcf3	NM_001079822	1.6
cDNA sequence BC049730	BC049730	BC049730	1.6
Hus1 homolog (S. pombe)	Hus1	NM_008316	1.6
mitochondrial ribosomal protein S35	Mrps35	NM_145573	1.6
cryptochrome 1 (photolyase-like)	Cry1	NM_007771	1.6
KISS1 receptor	Kiss1r	NM_053244	1.6
protein tyrosine phosphatase, receptor type, S	Ptprs	NM_011218	1.6
DCP1 decapping enzyme homolog A (S. cerevisiae)	Dcp1a	NM_133761	1.6
GDP-mannose pyrophosphorylase B	Gmppb	NM_177910	1.6
ring finger protein 31	Rnf31	NM_194346	1.6
heat shock protein 2	Hspb2	NM_024441	1.6
oviductal glycoprotein 1	Ovgp1	NM_007696	1.6
transmembrane emp24 domain containing 3	Tmed3	NM_025360	1.6
OTU domain, ubiquitin aldehyde binding 1	Otub1	NM_134150	1.6
TBC1 domain family, member 9	Tbc1d9	NM_001111304	1.6
ubiquitin-conjugating enzyme E2, J1	Ube2j1	NM_019586	1.6
aspartyl aminopeptidase	Dnpep	NM_001110831	1.6
grancalcin	Gca	NM_145523	1.6
Sec24 related gene family, member D (S. cerevisiae)	Sec24d	NM_027135	1.6
collagen, type IV, alpha 1	Col4a1	NM_009931	1.6
prune homolog (Drosophila)	Prune	NM_173347	1.6
homeo box D4	Hoxd4	NM_010469	1.6
Sp2 transcription factor	Sp2	NM_030220	1.6
RIKEN cDNA 1700081L11 gene	1700081L11Rik	BC043121	1.6
zinc finger protein 651	Zfp651	BC030045	1.6
CAP-GLY domain containing linker protein 2	Clip2	NM_009990	1.6
cellular retinoic acid binding protein I	Crabp1	NM_013496	1.6
cyclin D1	Ccnd1	NM_007631	1.6
coagulation factor IX	F9	NM_007979	1.6
family with sequence similarity 13, member C	Fam13c	NM_024244	1.6

Additional Table 2a. Differentially expressed cardiac genes in males compared to females (p ≤0.01) with a regulation of ≥ 1.5

DAVID GeneName	Symbol	Accession	Fold Change Male/Female
phosphatidylinositol-3,4,5-trisphosphate-dependent Rac exchange factor 1	Prex1	NM_177782	1.6
neurotrophic tyrosine kinase, receptor, type 1	Ntrk1	NM_001033124	1.6
RIKEN cDNA 9130011J15 gene	9130011J15Rik	BC055692	1.6
transmembrane protein 106A	Tmem106a	NM_144830	1.6
alkB, alkylation repair homolog 1 (E. coli)	Alkbh1	NM_001102565	1.6
PR domain containing 4	Prdm4	NM_181650	1.6
DIP2 disco-interacting protein 2 homolog B (Drosophila)	Dip2b	NM_001159361	1.6
kinesin family member 3C	Kif3c	NM_008445	1.6
AVL9 homolog (S. cerevisiase)	Avl9	NM_030235	1.6
phosphatidylinositol-4-phosphate 5-kinase, type 1 gamma	Pip5k1c	NM_008844	1.6
protein-O-mannosyltransferase 2	Pomt2	NM_153415	1.6
inositol polyphosphate-5-phosphatase D	Inpp5d	NM_010566	1.6
keratin 73	Krt73	NM_212485	1.6
engulfment and cell motility 2, ced-12 homolog (C. elegans)	Elmo2	NM_207705	1.6
transient receptor potential cation channel, subfamily V, member 2	Trpv2	NM_011706	1.6
predicted gene 967	Fam189a2	NM_001114174	1.6
Rap guanine nucleotide exchange factor (GEF) 1	Rapgef1	NM_001039087	1.6
phosphoglucomutase 5	Pgm5	NM_175013	1.6
protein tyrosine phosphatase, receptor type, D	Ptprd	NM_001014288	1.6
tubulin tyrosine ligase-like family, member 4	Ttll4	NM_001014974	1.6
Rho guanine nucleotide exchange factor (GEF) 12	Arhgef12	NM_027144	1.6
kinesin family member 16B	Kif16b	NM_001081133	1.6
chimerin (chimaerin) 2	Chn2	ENSMUST00000114403	1.6
cytohesin 3	Cyth3	NM_011182	1.6
glutathione peroxidase 3	Gpx3	NM_001083929	1.6
clathrin, heavy polypeptide (Hc)	Cltc	NM_001003908	1.6
RIKEN cDNA 4933426M11 gene	4933426M11Rik	BC040401	1.6
RAR-related orphan receptor gamma	Rorc	NM_011281	1.6
transmembrane channel-like gene family 6	Tmc6	NM_145439	1.6
sarcosine dehydrogenase	Sardh	NM_138665	1.6
thyrotropin releasing hormone receptor 2	Trhr2	NM_133202	1.6
NADH dehydrogenase (ubiquinone) 1 beta subcomplex 8	Ndufb8	NM_026061	1.6
chromodomain helicase DNA binding protein 7	Chd7	NM_001081417	1.6
suppression of tumorigenicity 13	St13	NM_133726	1.6
RIKEN cDNA 2310065K24 gene	2310065K24Rik	NM_028221	1.6
Rho GTPase activating protein 21	Arhgap21	NM_001128084	1.6
cDNA sequence BC021381	BC021381	NM_145382	1.6
plakophilin 4	Pkp4	NM_026361	1.6
leucine rich repeat containing 3B	Lrrc3b	NM_146052	1.6
erythrocyte protein band 4.1	Epb4.1	NM_183428	1.6
prokineticin 2	Prok2	NM_015768	1.6
eukaryotic elongation factor-2 kinase	Eef2k	NM_007908	1.6
neurobeachin	Nbea	NM_030595	1.6
acyl-CoA synthetase family member 2	Acsf2	NM_153807	1.6
cleavage stimulation factor, 3' pre-RNA, subunit 3	Cstf3	NM_145529	1.6
signal transducer and activator of transcription 5B	Stat5b	NM_001113563	1.6
angiomotin	Amot	NM_153319	1.6
transforming growth factor, beta 2	Tgfb2	NM_009367	1.6
EH-domain containing 3	Ehd3	NM_020578	1.6
N-acetyltransferase 9 (GCN5-related, putative)	Nat9	NM_025400	1.6

Additional Table 2a. Differentially expressed cardiac genes in males compared to females (p ≤0.01) with a regulation of ≥ 1.5

DAVID GeneName	Symbol	Accession	Fold Change Male/Female
phosphatidylglycerophosphate synthase 1	Pgs1	NM_133757	1.6
RIKEN cDNA 6430527G18 gene	6430527G18Rik	AF525300	1.6
yippee-like 5 (Drosophila)	Ypel5	NM_027166	1.6
lemur tyrosine kinase 2	Lmtk2	NM_001081109	1.6
TRAF3 interacting protein 2	Traf3ip2	NM_134000	1.6
LIM domain only 4	Lmo4	NM_010723	1.6
catenin beta interacting protein 1	Ctnnbip1	NM_023465	1.6
tescalcin	Tesc	NM_021344	1.6
zinc finger, FYVE domain containing 9	Zfyve9	NM_183300	1.6
DNA polymerase N	Poln	NM_181857	1.6
mediator complex subunit 24	Med24	NM_011869	1.6
erythrocyte protein band 4.1-like 2	Epb4.1l2	NM_013511	1.6
FAT tumor suppressor homolog 1 (Drosophila)	Fat1	NM_001081286	1.6
endoplasmic reticulum-golgi intermediate compartment (ERGIC) 1	Ergic1	NM_026170	1.6
RIKEN cDNA A730011L01 gene	A730011L01Rik	NM_177394	1.6
mannosidase, alpha, class 1B, member 1	Man1b1	NM_001029983	1.6
ATPase, aminophospholipid transporter (APLT), class I, type 8A, member 1	Atp8a1	NM_001038999	1.6
sorbin and SH3 domain containing 2	Sorbs2	NM_172752	1.6
ERGIC and golgi 3	Ergic3	NM_025516	1.6
solute carrier family 35, member E3	Slc35e3	NM_029875	1.6
dual-specificity tyrosine-(Y)-phosphorylation regulated kinase 1b	Dyrk1b	NM_001037957	1.6
solute carrier family 39 (metal ion transporter), member 6	Slc39a6	NM_139143	1.6
guanosine diphosphate (GDP) dissociation inhibitor 1	Gdi1	NM_010273	1.6
neurofibromatosis 1	Nf1	NM_010897	1.6
mediator complex subunit 23	Med23	NM_027347	1.6
zinc finger protein 295	Zfp295	NM_175428	1.6
kinesin family member C3	Kifc3	NM_010631	1.6
nitric oxide synthase 1 (neuronal) adaptor protein	Nos1ap	NM_001109985	1.6
RRP9, small subunit (SSU) processome component, homolog (yeast)	Rrp9	NM_145620	1.6
RAD54 like 2 (S. cerevisiae)	Rad54l2	NM_030730	1.6
splicing factor, arginine/serine-rich 15	Sfrs15	NM_178923	1.6
transmembrane protein 39b	Tmem39b	NM_199305	1.6
TRAF3 interacting protein 1	Traf3ip1	NM_028718	1.6
nascent polypeptide-associated complex alpha polypeptide	Naca	NM_001113199	1.6
DNA segment, Chr 17, ERATO Doi 663, expressed	D17Ertd663e	ENSMUST00000105044	1.5
thymoma viral proto-oncogene 1	Akt1	NM_009652	1.5
gamma-aminobutyric acid (GABA) A receptor, subunit epsilon	Gabre	NM_017369	1.5
phospholipase B domain containing 2	Plbd2	NM_023625	1.5
zinc finger CCCH type containing 11A	Zc3h11a	NM_144530	1.5
lipase, family member M	Lipm	NM_023903	1.5
mindbomb homolog 2 (Drosophila)	Mib2	NM_145124	1.5
promyelocytic leukemia	Pml	NM_008884	1.5
proteasome (prosome, macropain) 26S subunit, non-ATPase, 8	Psmd8	NM_026545	1.5
transient receptor potential cation channel, subfamily C, member 3	Trpc3	NM_019510	1.5
filamin, alpha	Flna	NM_010227	1.5
valyl-tRNA synthetase	Vars	NM_011690	1.5
NECAP endocytosis associated 1	Necap1	NM_026267	1.5
TCDD-inducible poly(ADP-ribose) polymerase	Tiparp	NM_178892	1.5
actin related protein 2/3 complex, subunit 5-like	Arpc5l	NM_028809	1.5
IQ motif and Sec7 domain 1	Iqsec1	NM_001134383	1.5

Additional Table 2a. Differentially expressed cardiac genes in males compared to females (p ≤0.01) with a regulation of ≥ 1.5

DAVID GeneName	Symbol	Accession	Fold Change Male/Female
pleckstrin homology domain containing, family A member 6	Plekha6	NM_182930	1.5
myotubularin related protein 9	Mtmr9	NM_177594	1.5
EF hand domain family A1	Efha1	NM_028643	1.5
septin 4	40425	NM_011129	1.5
predicted gene 11428	Gm11428	NM_001081957	1.5
mediator of RNA polymerase II transcription, subunit 11 homolog (S. cerevisiae)	Med11	NM_025397	1.5
rhomboid domain containing 2	Rhbdd2	NM_146002	1.5
lysine (K)-specific demethylase 2B	Kdm2b	NM_001003953	1.5
RasGEF domain family, member 1A	Rasgef1a	NM_027526	1.5
collagen, type I, alpha 1	Col1a1	NM_007742	1.5
doublesex and mab-3 related transcription factor like family C2	Dmrtc2	NM_027732	1.5
ral guanine nucleotide dissociation stimulator	Ralgds	NM_001145835	1.5
ring finger protein 126	Rnf126	NM_144528	1.5
ATPase type 13A2	Atp13a2	NM_029097	1.5
transmembrane protein 53	Tmem53	NM_026837	1.5
C-type lectin domain family 2, member d	Clec2d	NM_053109	1.5
mitogen-activated protein kinase 8 interacting protein 2	Mapk8ip2	NM_021921	1.5
Unknown	Gm9009	XR_004814	1.5
kelch-like 8 (Drosophila)	Klhl8	NM_178741	1.5
regucalcin	Rgn	NM_009060	1.5
oncostatin M receptor	Osmr	NM_011019	1.5
tripartite motif-containing 68	Trim68	NM_198012	1.5
RIKEN cDNA 4833413D08 gene	4833413D08Rik	BC047134	1.5
RAB36, member RAS oncogene family	Rab36	NM_029781	1.5
heparan sulfate 2-O-sulfotransferase 1	Hs2st1	NM_011828	1.5
pleckstrin homology-like domain, family B, member 1	Phldb1	NM_153537	1.5
growth factor receptor bound protein 2-associated protein 1	Gab1	NM_021356	1.5
myelocytomatosis oncogene	Myc	NM_010849	1.5
Lix1-like	Lix1l	ENSMUST00000062058	1.5
vacuolar protein sorting 13A (yeast)	Vps13a	NM_173028	1.5
cleft lip and palate associated transmembrane protein 1	Clptm1	NM_019649	1.5
nucleus accumbens associated 1, BEN and BTB (POZ) domain containing	Nacc1	NM_025788	1.5
cytochrome c oxidase, subunit VIb polypeptide 1	Cox6b1	NM_025628	1.5
predicted gene 6924	Gm6924	XM_893664	1.5
WD and tetratricopeptide repeats 1	Wdtc1	NM_199306	1.5
tensin like C1 domain-containing phosphatase	Tenc1	NM_153533	1.5
pleckstrin homology domain containing, family A (phosphoinositide binding specific) member 8	Plekha8	NM_001001335	1.5
RIKEN cDNA 4932418E24 gene	4932418E24Rik	NM_177841	1.5
tripartite motif-containing 24	Trim24	NM_145076	1.5
dopey family member 2	Dopey2	NM_027293	1.5
mitogen-activated protein kinase kinase kinase 8	Map3k8	NM_007746	1.5
a disintegrin-like and metallopeptidase (reprolysin type) with thrombospondin type 1 motif, 2	Adamts2	NM_175643	1.5
CDC42 binding protein kinase beta	Cdc42bpb	NM_183016	1.5
predicted gene 5737	Gm5737	XR_035619	1.5
reelin	Reln	NM_011261	1.5
zinc finger, DHHC-type containing 22	Zdhhc22	NM_001080943	1.5
synovial apoptosis inhibitor 1, synoviolin	Syvn1	NM_028769	1.5
transmembrane and coiled-coil domains 2	Tmcc2	NM_178874	1.5

Additional Table 2a. Differentially expressed cardiac genes in males compared to females (p ≤0.01) with a regulation of ≥ 1.5

DAVID GeneName	Symbol	Accession	Fold Change Male/Female
dual specificity phosphatase 6	Dusp6	NM_026268	1.5
zinc finger protein 148	Zfp148	NM_011749	1.5
ClpB caseinolytic peptidase B homolog (E. coli)	Clpb	NM_009191	1.5
phosphatidylinositol 4-kinase type 2 alpha	Pi4k2a	NM_145501	1.5
CCR4-NOT transcription complex, subunit 6-like	Cnot6l	NM_144910	1.5
exportin 6	Xpo6	NM_028816	1.5
integrin alpha V	Itgav	NM_008402	1.5
DCN1, defective in cullin neddylation 1, domain containing 4 (S. cerevisiae)	Dcun1d4	NM_178896	1.5
Unknown	Gm7614	XR_032236	1.5
splicing factor 3a, subunit 2	Sf3a2	NM_013651	1.5
G patch domain containing 4	Gpatch4	NM_025663	1.5
family with sequence similarity 181, member B	Fam181b	NM_021427	1.5
EGL nine homolog 3 (C. elegans)	Egln3	NM_028133	1.5
tetratricopeptide repeat, ankyrin repeat and coiled-coil containing 1	Tanc1	NM_198294	1.5
cartilage associated protein	Crtap	NM_019922	1.5
mitochondrial ribosomal protein L12	Mrpl12	NM_027204	1.5
Rho GTPase activating protein 10	Arhgap10	NM_030113	1.5
phospholipase C, epsilon 1	Plce1	NM_019588	1.5
p21 protein (Cdc42/Rac)-activated kinase 7	Pak7	NM_172858	1.5
zinc finger CCCH type containing 7B	Zc3h7b	NM_001081016	1.5
apoptosis antagonizing transcription factor	Aatf	NM_019816	1.5
keratin 1	Krt1	NM_008473	1.5
WD repeat domain, phosphoinositide interacting 1	Wipi1	NM_145940	1.5
GRAM domain containing 1A	Gramd1a	NM_027898	1.5
cysteine-rich with EGF-like domains 2	Creld2	NM_029720	1.5
coiled coil domain containing 88A	Ccdc88a	NM_176841	1.5
RIKEN cDNA 1700020N15 gene	Gm6812	NM_001098842	1.5
protein-kinase, interferon-inducible double stranded RNA dependent inhibitor, repressor of (P58 repressor)	Prkrir	NM_028410	1.5
frizzled-related protein	Frzb	NM_011356	1.5
myopalladin	Mypn	NM_182992	1.5
ring finger protein 216	Rnf216	NM_080561	1.5
BCL2-associated athanogene 3	Bag3	NM_013863	1.5
laminin, alpha 2	Lama2	NM_008481	1.5
iduronate 2-sulfatase	Ids	NM_010498	1.5
ATR interacting protein	Atrip	NM_172774	1.5
solute carrier family 44, member 2	Slc44a2	NM_152808	1.5
plexin A1	Plxna1	NM_008881	1.5
SRY-box containing gene 13	Sox13	NM_011439	1.5
retinoblastoma 1	Rb1	NM_009029	1.5
nuclear receptor subfamily 4, group A, member 2	Nr4a2	NM_001139509	1.5
cartilage oligomeric matrix protein	Comp	NM_016685	1.5
ryanodine receptor 3	Ryr3	BC116740	1.5
fumarylacetoacetate hydrolase domain containing 2A	Fahd2a	NM_029629	1.5
MyoD family inhibitor domain containing	Mdfic	NM_175088	1.5
carbonic anhydrase 8	Car8	NM_007592	1.5
serine (or cysteine) peptidase inhibitor, clade E, member 1	Serpine1	NM_008871	1.5
potassium voltage-gated channel, subfamily H (eag-related), member 1	Kcnh1	NM_010600	1.5
staphylococcal nuclease and tudor domain containing 1	Snd1	NM_019776	1.5
FCF1 small subunit (SSU) processome component homolog (S. cerevisiae)	Fcf1	NM_028632	1.5

Additional Table 2a. Differentially expressed cardiac genes in males compared to females (p ≤0.01) with a regulation of ≥ 1.5

DAVID GeneName	Symbol	Accession	Fold Change Male/Female
tubulin, beta 6	Tubb6	NM_026473	1.5
ubiquitin specific peptidase 1	Usp1	NM_146144	1.5
importin 8	Ipo8	NM_001081113	1.5
dishevelled, dsh homolog 1 (Drosophila)	Dvl1	NM_010091	1.5
sodium channel, voltage-gated, type VIII, alpha	Scn8a	NM_001077499	1.5
ubiquitin-conjugating enzyme E2, J2 homolog (yeast)	Ube2j2	NM_021402	1.5
SH3-domain binding protein 4	Sh3bp4	NM_133816	1.5
F-box protein 8	Fbxo8	NM_015791	1.5
zinc finger protein 652	Zfp652	NM_201609	1.5
sulfatase 1	Sulf1	NM_172294	1.5
DEAD (Asp-Glu-Ala-Asp) box polypeptide 10	Ddx10	NM_029936	1.5
sorting nexin 8	Snx8	NM_172277	1.5
thyroid adenoma associated	Thada	NM_183021	1.5
family with sequence similarity 100, member B	Gm7367	NR_003376	1.5
FK506 binding protein 3	Fkbp3	NM_013902	1.5
RIKEN cDNA A830018L16 gene	A830018L16Rik	NM_001160369	1.5
ubiquitin domain containing 1	Ubtd1	NM_145500	1.5
SEC16 homolog A (S. cerevisiae)	Sec16a	NM_153125	1.5
bromodomain containing 1	Brd1	NM_001033274	1.5
C-type lectin domain family 4, member g	Clec4g	NM_029465	1.5
syncoilin	Sync	NM_023485	1.5
receptor tyrosine kinase-like orphan receptor 1	Ror1	NM_013845	1.5
tubulin polymerization-promoting protein family member 3	Tppp3	NM_026481	1.5
nuclear receptor subfamily 1, group D, member 2	Nr1d2	NM_011584	1.5
mechanistic target of rapamycin (serine/threonine kinase)	Mtor	NM_020009	1.5
NLR family, pyrin domain containing 6	Nlrp6	NM_001081389	1.5
zinc finger protein 871	Zfp871	NM_172458	1.5
RIKEN cDNA 1700034F02 gene	1700034F02Rik	BC137683	1.5
serine/threonine kinase 38 like	Stk38l	NM_172734	1.5
RIKEN cDNA 1110008P14 gene	1110008P14Rik	NM_198001	1.5
RNA polymerase II associated protein 2	Rpap2	NM_144911	1.5
CUE domain containing 2	Cuedc2	NM_024192	1.5
Ras association (RalGDS/AF-6) domain family member 2	Rassf2	NM_175445	1.5
SET domain containing (lysine methyltransferase) 8	Setd8	NM_030241	1.5
tankyrase, TRF1-interacting ankyrin-related ADP-ribose polymerase	Tnks	NM_175091	1.5
fibroblast growth factor receptor 1	Fgfr1	NM_010206	1.5
CDC14 cell division cycle 14 homolog B (S. cerevisiae)	Cdc14b	NM_172587	1.5
pellino 3	Peli3	NM_172835	1.5
keratin 42	Krt42	NM_212483	1.5
proteasome (prosome, macropain) subunit, beta type 8 (large multifunctional peptidase 7)	Psmb8	NM_010724	1.5
presenilin 1	Psen1	NM_008943	1.5
multiple PDZ domain protein	Mpdz	NM_010820	1.5
UTP20, small subunit (SSU) processome component, homolog (yeast)	Utp20	NM_175158	1.5
myeloma overexpressed 2	Myeov2	BC147334	1.5
mitochondrial ribosomal protein L52	Mrpl52	NM_026851	1.5
Wolf-Hirschhorn syndrome candidate 1 (human)	Whsc1	NM_001081102	1.5
calcium binding protein 39	Cab39	NM_133781	1.5
DNA segment, Chr 1, ERATO Doi 448, expressed	D1Ertd448e	ENSMUST00000097783	1.5
cytochrome P450, family 2, subfamily u, polypeptide 1	Cyp2u1	NM_027816	1.5
spermidine synthase	Srm	NM_009272	1.5

Additional Table 2a. Differentially expressed cardiac genes in males compared to females (p ≤0.01) with a regulation of ≥ 1.5

DAVID GeneName	Symbol	Accession	Fold Change Male/Female
glycogen synthase kinase 3 alpha	Gsk3a	NM_001031667	1.5
matrix metallopeptidase 15	Mmp15	NM_008609	1.5
Sec1 family domain containing 2	Scfd2	NM_001114660	1.5
1-acylglycerol-3-phosphate O-acyltransferase 6 (lysophosphatidic acid acyltransferase, zeta)	Agpat6	NM_018743	1.5
solute carrier organic anion transporter family, member 2a1	Slco2a1	NM_033314	1.5
sorbin and SH3 domain containing 1	Sorbs1	NM_009166	1.5
polyhomeotic-like 3 (Drosophila)	Phc3	NM_153421	1.5
protein kinase C, delta	Prkcd	NM_011103	1.5
Unknown	Gm10334	NM_001103153	1.5
placental growth factor	Pgf	NM_008827	1.5
optineurin	Optn	NM_181848	1.5
sodium channel, voltage-gated, type I, beta	Scn1b	NM_011322	1.5
RIKEN cDNA B930041F14 gene	B930041F14Rik	ENSMUST00000099265	1.5
transforming growth factor, beta receptor III	Tgfbr3	NM_011578	1.5
ets variant gene 6 (TEL oncogene)	Etv6	NM_007961	1.5
pregnancy upregulated non-ubiquitously expressed CaM kinase	Pnck	NM_012040	1.5
suppressor of cytokine signaling 5	Socs5	NM_019654	1.5
DEAD (Asp-Glu-Ala-Asp) box polypeptide 51	Ddx51	NM_027156	1.5
prickle like 1 (Drosophila)	Prickle1	NM_001033217	1.5
expressed sequence AU021092	AU021092	BC150828	1.5
B-cell CLL/lymphoma 9-like	Bcl9l	NM_030256	1.5
poly (ADP-ribose) polymerase family, member 6	Parp6	NM_029922	1.5
excision repair cross-complementing rodent repair deficiency, complementation group 3	Ercc3	NM_133658	1.5
heat shock factor 1	Hsf1	NM_008296	1.5
solute carrier family 26 (sulfate transporter), member 2	Slc26a2	NM_007885	1.5
myosin, light chain 12B, regulatory	Myl12b	NM_023402	1.5
platelet derived growth factor receptor, beta polypeptide	Pdgfrb	NM_001146268	1.5
plasmacytoma variant translocation 1	Pvt1	NR_003368	1.5
transmembrane protein 56	Tmem56	NM_178936	1.5
legumain	Lgmn	NM_011175	1.5
adaptor-related protein complex 3, delta 1 subunit	Ap3d1	NM_007460	1.5
RIKEN cDNA 4930408O21 gene	4930408O21Rik	BC147551	1.5
RAS protein activator like 2	Rasal2	NM_177644	1.5
septin 14	1700017B05Rik	NM_028820	1.5
Unknown	Palm2	NM_172868	1.5
zinc finger protein 758	Zfp758	NM_145484	1.5
SH2B adaptor protein 3	Sh2b3	NM_008507	1.5
solute carrier family 23 (nucleobase transporters), member 2	Slc23a2	NM_018824	1.5
sprouty homolog 2 (Drosophila)	Spry2	NM_011897	1.5
ribosomal RNA processing 8, methyltransferase, homolog (yeast)	Rrp8	NM_025897	1.5
chromodomain helicase DNA binding protein 1	Chd1	NM_007690	1.5
G protein-coupled receptor 113	Gpr113	NM_001014394	1.5
spectrin alpha 2	Spna2	NM_001076554	1.5
kinesin-associated protein 3	Kifap3	NM_010629	1.5
protein tyrosine phosphatase, receptor type, B	Ptprb	NM_029928	1.5
sorting nexin 1	Snx1	NM_019727	1.5
interferon stimulated exonuclease gene 20-like 2	Isg20l2	NM_177663	1.5
GC-rich promoter binding protein 1-like 1	Gpbp1l1	NM_029868	1.5
synapse defective 1, Rho GTPase, homolog 1 (C. elegans)	Syde1	NM_027875	1.5

Additional Table 2a. Differentially expressed cardiac genes in males compared to females (p ≤0.01) with a regulation of ≥ 1.5

DAVID GeneName	Symbol	Accession	Fold Change Male/Female
Mid1 interacting protein 1 (gastrulation specific G12-like (zebrafish))	Mid1ip1	NM_026524	1.5
prefoldin 1	Pfdn1	NM_026027	1.5
Unknown	6330442E10Rik	BC079613	1.5
WD repeat domain 68	Dcaf7	NM_027946	1.5
kelch-like 18 (Drosophila)	Klhl18	NM_177771	1.5
lethal giant larvae homolog 1 (Drosophila)	Llgl1	NM_001159405	1.5
LIM domain only 2	Lmo2	NM_008505	1.5
ring finger protein 187	Rnf187	AB030190	1.5
Rho guanine nucleotide exchange factor (GEF) 10-like	Arhgef10l	NM_172415	1.5
tripartite motif-containing 30	Trim30	NM_009099	1.5
caldesmon 1	Cald1	NM_145575	1.5
expressed sequence C77080	C77080	NM_001033189	1.5
GLIS family zinc finger 2	Glis2	NM_031184	1.5
solute carrier family 4 (anion exchanger), member 4	Slc4a4	NM_018760	1.5
Fc receptor, IgG, low affinity III	Fcgr3	NM_010188	1.5
cleavage stimulation factor, 3' pre-RNA subunit 2	Cstf2	NM_133196	1.5
oxysterol binding protein-like 6	Osbpl6	NM_145525	1.5
salivary protein 1	Spt1	NM_009267	1.5
phosphatase and actin regulator 2	Phactr2	NM_001033257	1.5
solute carrier family 4 (anion exchanger), member 3	Slc4a3	NM_009208	1.5
neuropeptide FF receptor 2	Npffr2	NM_133192	1.5
purine-nucleoside phosphorylase 2	Pnp2	NM_001123371	1.5
protein disulfide isomerase associated 3	Pdia3	NM_007952	1.5
disabled homolog 2 (Drosophila)	Dab2	NM_023118	1.5
tumor necrosis factor receptor superfamily, member 13c	Tnfrsf13c	NM_028075	1.5
RIKEN cDNA 2010309E21 gene	Fam136a	BC103775	1.5
G kinase anchoring protein 1	Gkap1	NM_019832	1.5
RIKEN cDNA B230118H07 gene	B230118H07Rik	DQ112092	1.5
prolactin releasing hormone receptor	Prlhr	NM_201615	1.5
glutamate receptor interacting protein 2	Grip2	NM_001159507	1.5
nuclear receptor subfamily 5, group A, member 2	Nr5a2	NM_030676	1.5
M-phase phosphoprotein 8	Mphosph8	NM_023773	1.5
RIKEN cDNA 1700011L22 gene	1700011L22Rik	ENSMUST00000034109	1.5
WAS/WASL interacting protein family, member 1	Wipf1	NM_153138	1.5

Additional Table 2b. Genderwise differentially expressed genes (p ≤0.01) with a regulation of ≥ 1.5

DAVID GeneName	Gene Symbol	RefSeq	Fold Change Female/Males
**Relatively upregulated in females ≥3-fold**			
inactive X specific transcripts	Xist	NR_001463	55.6
interleukin 12 receptor, beta 2	Il12rb2	NM_008354	41.7
TBC1 domain family, member 30	Tbc1d30	NM_029057	28.5
renal tumor antigen	Stk30	BC039981	26.1
RIKEN cDNA 2610109H07 gene	2610109H07Rik	NM_027426	22.0
Na+/H+ exchanger domain containing 1	Nhedc1	ENSMUST00000090209	19.9
RIKEN cDNA A430105I19 gene	A430105I19Rik	NM_001001982	18.0
RIKEN cDNA 4930488N24 gene	EG635895	NM_001122977	17.6
SPHK1 interactor, AKAP domain containing	Sphkap	NM_172430	16.7
zinc finger protein 446	Zfp446	NM_175558	16.4
N-terminal EF-hand calcium binding protein 2	Necab2	NM_054095	14.4
WD repeat domain 51A	Wdr51a	NM_027354	13.7
ATP-binding cassette, sub-family A (ABC1), member 17	Abca17	NM_001031621	13.2
matrix metallopeptidase 7	Mmp7	NM_010810	13.0
caudal type homeo box 2	Cdx2	NM_007673	12.9
glycine receptor, alpha 4 subunit	Glra4	NM_010297	12.6
myosin VIIB	Myo7b	NM_032394	12.3
solute carrier family 3, member 1	Slc3a1	NM_009205	12.1
keratin 36	Krt36	NR_003957	11.8
predicted gene 10044	Gm10044	ENSMUST00000090634	11.7
Sh3 domain YSC-like 1	Sh3yl1	NM_013709	11.7
BCL2-like 14 (apoptosis facilitator)	Bcl2l14	NM_025778	11.6
villin-like	Vill	NM_011700	11.5
integrin, alpha 10	Itga10	NM_001081053	11.2
complement component 1, r subcomponent	C1rb	NM_001113356	11.1
T cell receptor associated transmembrane adaptor 1	Trat1	ENSMUST00000099715	10.5
tetratricopeptide repeat domain 21A	Ttc21a	NM_028735	10.5
THAP domain containing, apoptosis associated protein 3	Thap3	ENSMUST00000105665	10.2
glutamate receptor, metabotropic 7	Grm7	NM_177328	10.1
collectin sub-family member 10	Colec10	NM_173422	9.7
wingless related MMTV integration site 8b	Wnt8b	NM_011720	9.4
deubiquitinating enzyme 2a	Dub2a	NM_001001559	9.0
predicted gene 11190	Gm11190	ENSMUST00000068419	9.0
RIKEN cDNA 9130409I23 gene	9130409I23Rik	BC147379	8.9
Fanconi anemia, complementation group G	Fancg	NM_053081	8.9
L-amino acid oxidase 1	Lao1	NM_133892	8.8
expressed sequence C79407	C79407	BC021832	8.8
RIKEN cDNA 4930506C21 gene	4930506C21Rik	AK043779	8.7
similar to dachsous 2 isoform 2	LOC633371	XR_031743	8.6
pyroglutamylated RFamide peptide	Qrfp	NM_183424	8.5
purinergic receptor P2X, ligand-gated ion channel, 2	P2rx2	NM_153400	8.5
fibrillin 2	Fbn2	NM_010181	8.4
predicted gene 9920	Gm9920	ENSMUST00000100659	8.3
zinc fingerprotein 618	Zfp618	NM_028326	8.2
CD70 antigen	Cd70	NM_011617	8.2
transmembrane protein 132A	Tmem132a	NM_133804	8.2
chemokine (C-X-C motif) ligand 17	Cxcl17	NM_153576	8.2
butyrophilin, subfamily 2, member A2	Btn2a2	NM_175938	8.2
synaptotagmin IX	Syt9	NM_021889	8.1

Additional Table 2b. Genderwise differentially expressed genes (p ≤0.01) with a regulation of ≥ 1.5

DAVID GeneName	Gene Symbol	RefSeq	Fold Change Female/Males
expressed sequence AI428936	AI428936	NM_153577	8.0
growth arrest-specific 2 like 2	Gas2l2	NM_001013759	8.0
HMG box domain containing 4	Hmgxb4	NM_178017	7.8
hyaluronan mediated motility receptor (RHAMM)	Hmmr	NM_013552	7.7
zinc finger protein 783	Zfp783	NR_027963	7.7
RIKEN cDNA 9330154K18 gene	9330154K18Rik	AK034078	7.7
antigen p97 (melanoma associated) identified by monoclonal antibodies 133.2 and 96.5	Mfi2	NM_013900	7.7
N-acetylated alpha-linked acidic dipeptidase-like 1	Naaladl1	NM_001009546	7.6
intraflagellar transport 140 homolog (Chlamydomonas)	Ift140	NM_134126	7.6
sodium channel, voltage-gated, type III, beta	Scn3b	NM_153522	7.5
coiled-coil domain containing 78	Ccdc78	ENSMUST00000095500	7.3
hydroxy-delta-5-steroid dehydrogenase, 3 beta- and steroid delta-isomerase 2	Hsd3b2	NM_153193	7.3
tumor necrosis factor (ligand) superfamily, member 14	Tnfsf14	NM_019418	7.1
suppression inducing transmembrane adaptor 1	Sit1	NM_019436	7.1
expressed sequence AA467197	AA467197	ENSMUST00000047498	7.0
mannosidase, endo-alpha-like	Maneal	NM_001007573	7.0
nuclear receptor subfamily 1, group H, member 5	Nr1h5	NM_198658	7.0
chitinase like protein 2	Bclp2	NM_001080816	7.0
Fraser syndrome 1 homolog (human)	Fras1	NM_175473	6.9
cDNA sequence AY036118	AY036118	AY036118	6.9
S100 calcium binding protein A3	S100a3	NM_011310	6.8
RIKEN cDNA 1700011A15 gene	1700011A15Rik	BC048463	6.7
olfactory receptor 631	Olfr631	NM_146959	6.6
zinc finger CCCH type containing 12A	Zc3h12a	NM_153159	6.6
Opa interacting protein 5	Oip5	NM_001042653	6.6
zinc finger protein 354B	Zfp354b	NM_013744	6.6
desmoglein 4	Dsg4	NM_181564	6.6
Unknown	LOC100044795	XM_001473515	6.5
a disintegrin-like and metallopeptidase (reprolysin type) with thrombospondin type 1 motif, 14	Adamts14	NM_001081127	6.5
transmembrane protease, serine 11f	Tmprss11f	NM_178730	6.4
Unknown	40427	ENSMUST00000053456	6.4
predicted gene 12541	Gm12541	ENSMUST00000068009	6.4
poliovirus receptor-related 4	Pvrl4	NM_027893	6.4
predicted gene 853	Gm853	BC147395	6.4
WD repeat domain 52	Wdr52	BC100518	6.4
sphingomyelin phosphodiesterase, acid-like 3B	Smpdl3b	NM_133888	6.4
nephronectin	Npnt	NM_033525	6.3
Down syndrome cell adhesion molecule	Dscam	NM_031174	6.3
olfactory receptor 521	Olfr521	NM_146356	6.3
calcium channel, voltage-dependent, beta 1 subunit	Cacnb1	NM_145121	6.3
cDNA sequence BC055111	BC055111	NM_183182	6.2
calcyphosphine 2	Caps2	NM_178278	6.2
mucolipin 3	Mcoln3	NM_134160	6.2
small nucleolar RNA, C/D box 82	Snord82	NR_002851	6.2
kinesin family member 20B	Kif20b	NM_183046	6.2
Unknown	1700007N14Rik	ENSMUST00000111175	6.0
killer cell lectin-like receptor subfamily K, member 1	Klrk1	NM_033078	6.0
UDP glucuronosyltransferase 2 family, polypeptide A3	Ugt2a3	NM_028094	6.0

Additional Table 2b. Genderwise differentially expressed genes (p ≤0.01) with a regulation of ≥ 1.5

DAVID GeneName	Gene Symbol	RefSeq	Fold Change Female/Males
fractured callus expressed transcript 1	Fxc1	ENSMUST00000084778	6.0
G protein-coupled receptor kinase 1	Grk1	NM_011881	6.0
aristaless-like homeobox 3	Alx3	NM_007441	5.8
NLR family, pyrin domain containing 4D	Nlrp4d	AY596195	5.8
BAI1-associated protein 3	Baiap3	BC158116	5.8
fibroblast growth factor receptor 4	Fgfr4	NM_008011	5.8
HAUS augmin-like complex, subunit 1	Haus1	NM_146089	5.8
SRY-box containing gene 15	Sox15	NM_009235	5.7
IQ motif containing K	Iqck	NM_001081446	5.7
olfactory receptor 1125	Olfr1125	NM_147028	5.7
RIKEN cDNA 4930555F03 gene	4930555F03Rik	ENSMUST00000033963	5.7
docking protein 5	Dok5	NM_029761	5.6
formiminotransferase cyclodeaminase	Ftcd	NM_080845	5.6
RIKEN cDNA 2610028A01 gene	2610028A01Rik	NM_028228	5.6
aspartoacylase	Aspa	NM_023113	5.5
hepatitis A virus cellular receptor 1	Havcr1	NM_134248	5.5
family with sequence similarity 3, member B	Fam3b	NM_020622	5.5
PDZ domain containing 3	Pdzd3	NM_133226	5.5
predicted gene 13089	Gm13089	ENSMUST00000073532	5.5
Rpgrip1-like	Rpgrip1l	NM_173431	5.5
BarH-like 1 (Drosophila)	Barhl1	ENSMUST00000113847	5.5
amyotrophic lateral sclerosis 2 (juvenile) chromosome region, candidate 4	Als2cr4	NM_001033449	5.4
3-hydroxyanthranilate 3,4-dioxygenase	Haao	NM_025325	5.4
tolloid-like 2	Tll2	NM_011904	5.4
cytochrome P450, family 17, subfamily a, polypeptide 1	Cyp17a1	NM_007809	5.4
fibronectin type 3 and ankyrin repeat domains 1	Fank1	ENSMUST00000065359	5.3
M phase phosphoprotein 6	Mphosph6	NM_026758	5.3
RIKEN cDNA 2410089E03 gene	2410089E03Rik	ENSMUST00000053264	5.2
cholinergic receptor, nicotinic, gamma polypeptide	Chrng	NM_009604	5.2
protease, serine, 35	Prss35	BC075675	5.2
solute carrier family 24, member 5	Slc24a5	NM_175034	5.2
RIKEN cDNA B830028B13 gene	B830028B13Rik	NM_001143802	5.2
cholinergic receptor, nicotinic, beta polypeptide 3	Chrnb3	NM_173212	5.1
janus kinase and microtubule interacting protein 2	Jakmip2	ENSMUST00000097589	5.1
solute carrier family 39 (zinc transporter), member 12	Slc39a12	NM_001012305	5.1
histidine-rich glycoprotein	Hrg	NM_053176	5.0
potassium voltage-gated channel, subfamily Q, member 5	Kcnq5	NM_023872	5.0
carboxypeptidase A6	Cpa6	NM_177834	5.0
Unknown	LOC100047557	XM_001478429	4.9
progestin and adipoQ receptor family member V	Paqr5	NM_028748	4.9
RIKEN cDNA 2010110P09 gene	2010110P09Rik	NM_027363	4.9
cDNA sequence BC030500	BC030500	BC030500	4.9
vang-like 2 (van gogh, Drosophila)	Vangl2	NM_033509	4.8
RIKEN cDNA 4921530D09 gene	4921530D09Rik	ENSMUST00000028309	4.8
encoding X-linked lymphocyte-regulated 3C	Xlr3c	NM_011727	4.8
l(3)mbt-like 4 (Drosophila)	L3mbtl4	BC147119	4.8
RIKEN cDNA 6720416L17 gene	6720416L17Rik	ENSMUST00000100000	4.8
potassium voltage-gated channel, shaker-related subfamily, beta member 3	Kcnab3	NM_010599	4.8
F-box and leucine-rich repeat protein 13	Fbxl13	NM_177076	4.8
family with sequence similarity 62 (C2 domain containing), member C	Esyt3	NM_177775	4.8

Additional Table 2b. Genderwise differentially expressed genes (p ≤0.01) with a regulation of ≥ 1.5

DAVID GeneName	Gene Symbol	RefSeq	Fold Change Female/Males
thioredoxin domain containing 2 (spermatozoa)	Txndc2	NM_001146002	4.7
glycogen synthase 2	Gys2	NM_145572	4.7
transmembrane protein 108	Tmem108	NM_178638	4.7
zinc finger CCCH type containing 12D	Zc3h12d	NM_172785	4.7
cyclin-dependent kinase 3	Cdk3	NR_004853	4.7
stabilin 2	Stab2	NM_138673	4.7
stem-loop binding protein	Slbp	ENSMUST00000075670	4.7
anoctamin 4	Ano4	NM_178773	4.7
tryptase alpha/beta 1	Tpsab1	NM_031187	4.7
sex hormone binding globulin	Shbg	NM_011367	4.7
potassium voltage-gated channel, subfamily Q, member 4	Kcnq4	NM_001081142	4.7
sodium channel, voltage-gated, type I, alpha	Scn1a	NM_018733	4.6
SHC (Src homology 2 domain containing) family, member 4	Shc4	NM_199022	4.6
acyl-CoA synthetase medium-chain family member 4	Acsm4	NM_178414	4.6
chitinase, acidic	Chia	NM_023186	4.6
connector enhancer of kinase suppressor of Ras 2	Cnksr2	NM_177751	4.6
interferon, alpha 14	Ifna14	BC120722	4.6
Purkinje cell protein 4	Pcp4	NM_008791	4.6
collagen and calcium binding EGF domains 1	Ccbe1	NM_178793	4.5
renin 1 structural	Ren1	NM_031192	4.5
tubulin tyrosine ligase-like family, member 3	Ttll3	NM_133923	4.5
RNA binding motif protein 47	Rbm47	NM_178446	4.5
leukocyte cell-derived chemotaxin 2	Lect2	NM_010702	4.5
unkempt homolog (Drosophila)	Unk	NM_172569	4.5
small nuclear ribonucleoprotein 35 (U11/U12)	Snrnp35	NM_029532	4.5
matrix metallopeptidase 10	Mmp10	NM_019471	4.5
DNA segment, Chr 5, ERATO Doi 577, expressed	D5Ertd577e	NM_177187	4.4
Unknown	Gm16367	BC099967	4.4
Unknown	Gm7792	ENSMUST00000101023	4.4
B-box and SPRY domain containing	Bspry	NM_138653	4.4
predicted gene 10637	Gm10637	ENSMUST00000098528	4.4
tau tubulin kinase 1	Ttbk1	NM_001162864	4.4
RIKEN cDNA 1700041C02 gene	Ccdc30	NM_029286	4.4
cytochrome P450, family 1, subfamily a, polypeptide 1	Cyp1a1	NM_009992	4.3
predicted gene 9968	Gm9968	ENSMUST00000068934	4.3
predicted gene, EG624120	Gm16401	ENSMUST00000072800	4.3
adhesion molecule with Ig like domain 3	Amigo3	NM_177275	4.3
NEL-like 2 (chicken)	Nell2	NM_016743	4.3
similar to Zinc finger protein 267 (Zinc finger protein HZF2)	Gm5581	ENSMUST00000072940	4.3
Fras1 related extracellular matrix protein 2	Frem2	NM_172862	4.3
peptidylprolyl isomerase (cyclophilin) like 5	Ppil5	NM_001081406	4.3
DNA replication helicase 2 homolog (yeast)	Dna2	NM_177372	4.3
solute carrier family 25, member 35	Slc25a35	ENSMUST00000018884	4.2
predicted gene 11435	Gm11435	NM_001045543	4.2
carcinoembryonic antigen-related cell adhesion molecule 16	Ceacam16	NM_001033419	4.2
solute carrier family 16 (monocarboxylic acid transporters), member 8	Slc16a8	NM_020516	4.1
a disintegrin and metallopeptidase domain 6A	Adam6a	NM_174885	4.1
family with sequence similarity 115, member C	Fam115c	AK131169	4.1
T cell receptor alpha variable 9N-3	Trav9n-3	XM_001479640	4.1
homeo box C4	Hoxc4	NM_013553	4.1

Additional Table 2b. Genderwise differentially expressed genes (p ≤0.01) with a regulation of ≥ 1.5

DAVID GeneName	Gene Symbol	RefSeq	Fold Change Female/Males
BH3 interacting domain death agonist	Bid	NM_007544	4.1
RIKEN cDNA 4932411G14 gene	4932411G14Rik	NM_177711	4.1
Unknown	Higd1c	NM_001002900	4.1
predicted gene 14340	Gm14340	ENSMUST00000098982	4.1
RIKEN cDNA F630111L10 gene	F630111L10Rik	AK170843	4.1
zinc finger and SCAN domain containing 5B	Zscan5b	NM_133204	4.1
acetoacetyl-CoA synthetase	Aacs	NM_030210	4.1
transglutaminase 7	Tgm7	NM_001160424	4.0
cytochrome P450, family 4, subfamily f, polypeptide 13	Cyp4f13	NM_130882	4.0
small Cajal body-specific RNA 8	Scarna8	AF357402	4.0
MARVEL (membrane-associating) domain containing 3	Marveld3	NM_028584	4.0
melanoma associated antigen (mutated) 1-like 1	Mum1l1	ENSMUST00000113045	4.0
matrix metallopeptidase 12	Mmp12	NM_008605	4.0
synaptonemal complex protein 3	Sycp3	NM_011517	4.0
acyl-CoA thioesterase 12	Acot12	NM_028790	4.0
transient receptor potential cation channel, subfamily C, member 2	Trpc2	ENSMUST00000106950	3.9
galactose-3-O-sulfotransferase 3	Gal3st3	NM_001024717	3.9
transient receptor potential cation channel, subfamily M, member 3	Trpm3	NM_177341	3.9
ferric-chelate reductase 1	Frrs1	NM_001113478	3.9
cathepsin R	Ctsr	NM_020284	3.9
zinc finger protein 839	Zfp839	NM_028365	3.9
RIKEN cDNA 1300002K09 gene	1300002K09Rik	NM_028788	3.9
hemogen	Hemgn	NM_053149	3.9
ferredoxin reductase	Fdxr	NM_007997	3.9
POU domain, class 6, transcription factor 2	Pou6f2	NM_175006	3.9
signal peptide, CUB domain, EGF-like 3	Scube3	NM_001004366	3.9
protein phosphatase 4, regulatory subunit 4	Ppp4r4	NM_028980	3.8
RIKEN cDNA C630004L07 gene	C630004L07Rik	AK049864	3.8
neurexin I	Nrxn1	NM_020252	3.8
RIKEN cDNA 1700045I19 gene	1700045I19Rik	NR_003640	3.8
islet cell autoantigen 1-like	Ica1l	NM_027407	3.8
WAP four-disulfide core domain 10	Wfdc10	DQ437333	3.8
vesicle-associated membrane protein 1	Vamp1	NM_009496	3.8
RIKEN cDNA D430042O09 gene	D430042O09Rik	AK122309	3.8
cytochrome P450, family 7, subfamily b, polypeptide 1	Cyp7b1	NM_007825	3.8
Unknown	Gm2889	XR_032134	3.8
fucosyltransferase 7	Fut7	NM_013524	3.8
RNA, Y3 small cytoplasmic (associated with Ro protein)	Rny3	NR_024202	3.8
zinc finger, SWIM domain containing 2	Zswim2	NM_027964	3.8
predicted gene 1631	Gm1631	NM_201366	3.8
WD repeat domain 8	Wdr8	NM_021499	3.8
neurogenic differentiation 6	Neurod6	NM_009717	3.8
glycolipid transfer protein domain containing 1	Gltpd1	NM_024472	3.8
forkhead box P2	Foxp2	NM_053242	3.8
protein phosphatase 1, regulatory (inhibitor) subunit 16B	Ppp1r16b	ENSMUST00000052927	3.8
nitric oxide synthase 1, neuronal	Nos1	NM_008712	3.7
RIKEN cDNA B230216G23 gene	B230216G23Rik	ENSMUST00000088263	3.7
carbamoyl-phosphate synthetase 1	Cps1	BC067211	3.7
pre T-cell antigen receptor alpha	Ptcra	NM_011195	3.7
hexamthylene bis-acetamide inducible 2	Hexim2	NM_027658	3.7

Additional Table 2b. Genderwise differentially expressed genes (p ≤0.01) with a regulation of ≥ 1.5

DAVID GeneName	Gene Symbol	RefSeq	Fold Change Female/Males
ankyrin repeat domain 53	Ankrd53	NM_029245	3.7
signal transducer and activator of transcription 4	Stat4	NM_011487	3.7
complement component 1, q subcomponent-like 2	C1ql2	NM_207233	3.7
amyloid beta (A4) precursor protein-binding, family A, member 2	Apba2	NM_007461	3.7
predicted gene 5840	Gm5840	NM_001080811	3.7
GINS complex subunit 1 (Psf1 homolog)	Gins1	ENSMUST00000028948	3.6
secretory leukocyte peptidase inhibitor	Slpi	NM_011414	3.6
alkB, alkylation repair homolog 4 (E. coli)	Alkbh4	ENSMUST00000041100	3.6
RIKEN cDNA 4930432K21 gene	4930432K21Rik	BC075658	3.6
Unknown	Gm5098	ENSMUST00000104904	3.6
Williams-Beuren syndrome chromosome region 17 homolog (human)	Wbscr17	NM_145218	3.6
RIKEN cDNA 4933434I20 gene	4933434I20Rik	NM_026233	3.6
TNF receptor-associated factor 3	Traf3	NM_011632	3.6
PDZ and LIM domain 2	Pdlim2	NM_145978	3.6
cytoplasmic polyadenylated homeobox	Cphx	NM_175342	3.6
C-type lectin domain family 2, member e	Clec2e	NM_153506	3.6
prostaglandin D2 synthase 2, hematopoietic	Hpgds	NM_019455	3.6
complement component 8, gamma polypeptide	C8g	NM_027062	3.6
solute carrier family 7 (cationic amino acid transporter, y+ system), member 4	Slc7a4	NM_144852	3.6
chemokine (C-C motif) receptor 10	Ccr10	NM_007721	3.6
tripartite motif-containing 17	Trim17	NM_031172	3.6
RIKEN cDNA 5730460C07 gene	5730460C07Rik	ENSMUST00000081193	3.5
kinesin family member 6	Kif6	AK083760	3.5
ENTH domain containing 1	Enthd1	ENSMUST00000096347	3.5
RIKEN cDNA 4930512M02 gene	4930512M02Rik	ENSMUST00000077905	3.5
RIKEN cDNA 1700042B14 gene	1700042B14Rik	BC100353	3.5
phenylalanine hydroxylase	Pah	NM_008777	3.5
microtubule associated serine/threonine kinase 1	Mast1	NM_019945	3.5
TEA domain family member 2	Tead2	NM_011565	3.5
DnaJ (Hsp40) homolog, subfamily C, member 6	Dnajc6	ENSMUST00000038207	3.5
zinc finger protein 112	Zfp112	NM_021307	3.5
C-type lectin domain family, member l	Clec2l	NM_001101507	3.5
galanin	Gal	NM_010253	3.5
RIKEN cDNA 1700094C09 gene	1700094C09Rik	BC048522	3.5
hypothetical protein LOC100045792	Ect2l	ENSMUST00000095817	3.5
RIKEN cDNA 1700025H01 gene	1700025H01Rik	ENSMUST00000061675	3.5
regulator of G protein signaling 7	Rgs7	NM_011880	3.5
runt-related transcription factor 1	Runx1t1	NM_001111027	3.5
Rho-guanine nucleotide exchange factor	Rgnef	NM_012026	3.5
platelet-derived growth factor receptor-like	Pdgfrl	NM_026840	3.4
non-metastatic cells 4, protein expressed in	Nme4	NM_019731	3.4
mannosyl (alpha-1,3-)-glycoprotein beta-1,4-N-acetylglucosaminyltransferase, isozyme C (putative)	Mgat4c	NM_001162369	3.4
dpy-19-like 2 (C. elegans)	Dpy19l2	AK029497	3.4
cDNA sequence BC027072	BC027072	BC046516	3.4
ectodysplasin-A	Eda	NM_010099	3.4
NK2 transcription factor related, locus 6 (Drosophila)	Nkx2-6	NM_010920	3.4
transmembrane protein 190	Tmem190	ENSMUST00000013235	3.4
dynein, axonemal, heavy chain 14	Dnahc14	ENSMUST00000060610	3.4

Additional Table 2b. Genderwise differentially expressed genes (p ≤0.01) with a regulation of ≥ 1.5

DAVID GeneName	Gene Symbol	RefSeq	Fold Change Female/Males
kinesin family member 23	Kif23	NM_024245	3.4
RIKEN cDNA 2900083I11 gene	2900083I11Rik	NM_021403	3.4
predicted gene 5708	Gm5708	XR_032892	3.4
basic leucine zipper transcription factor, ATF-like 2	Batf2	NM_028967	3.4
helicase, POLQ-like	Helq	NM_001081107	3.3
RIKEN cDNA 9130008F23 gene	9130008F23Rik	NM_027834	3.3
sodium channel, nonvoltage-gated 1 beta	Scnn1b	NM_011325	3.3
hemolytic complement	Hc	NM_010406	3.3
nuclear receptor subfamily 1, group I, member 3	Nr1i3	NM_009803	3.3
coagulation factor C homolog (Limulus polyphemus)	Coch	NM_007728	3.3
RIKEN cDNA 4930403N07 gene	4930403N07Rik	ENSMUST00000021494	3.3
cytochrome P450, family 2, subfamily d, polypeptide 10	Cyp2d10	NM_010005	3.3
RIKEN cDNA B230315N10 gene	B230315N10Rik	BC040349	3.3
BAH domain and coiled-coil containing 1	Bahcc1	NM_198423	3.3
A kinase (PRKA) anchor protein 3	Akap3	NM_009650	3.3
RIKEN cDNA 2810422O20 gene	2810422O20Rik	BC050143	3.3
olfactory receptor 1216	Olfr1216	NM_146893	3.3
RAS-related C3 botulinum substrate 3	Rac3	NM_133223	3.3
serine (or cysteine) peptidase inhibitor, clade B, member 5	Serpinb5	NM_009257	3.3
Eph receptor A5	Epha5	NM_007937	3.3
fibroblast growth factor receptor substrate 3	Frs3	NM_144939	3.3
parathyroid hormone 2 receptor	Pth2r	NM_139270	3.3
phosphoinositide-3-kinase, regulatory subunit 5, p101	Pik3r5	NM_177320	3.3
4-nitrophenylphosphatase domain and non-neuronal SNAP25-like protein homolog 1 (C. elegans)	Nipsnap1	NM_008698	3.3
dolichyl-phosphate mannosyltransferase polypeptide 3	Dpm3	BC037761	3.3
stathmin-like 3	Stmn3	NM_009133	3.3
histocompatibility 2, M region locus 2	H2-M2	NM_008204	3.2
RIKEN cDNA D430018E03 gene	D430018E03Rik	ENSMUST00000014133	3.2
RIKEN cDNA 4930404A10 gene	4930404A10Rik	NM_029105	3.2
Unknown	Gm5188	ENSMUST00000037962	3.2
Rho guanine nucleotide exchange factor (GEF) 1	Arhgef1	NM_001130150	3.2
solute carrier family 25, member 28	Slc25a28	NM_145156	3.2
cDNA sequence BC024139	BC024139	NM_001142968	3.2
carcinoembryonic antigen-related cell adhesion molecule	Ceacam3	ENSMUST00000108491	3.2
late cornified envelope 1B	Lce1b	NM_026822	3.2
glial cells missing homolog 1 (Drosophila)	Gcm1	NM_008103	3.2
collagen, type IV, alpha 6	Col4a6	NM_053185	3.2
zona pellucida glycoprotein 3	Zp3	NM_011776	3.2
small nucleolar RNA host gene 11 (non-protein coding)	Snhg11	NM_175692	3.2
neural cell adhesion molecule 2	Ncam2	NM_001113208	3.2
transmembrane protease, serine 9	Tmprss9	NM_001081688	3.2
coiled-coil domain containing 120	Ccdc120	NM_207202	3.2
musculin	Msc	NM_010827	3.2
Fas apoptotic inhibitory molecule 2	Faim2	NM_028224	3.2
ets variant gene 3-like	Etv3l	XM_909234	3.2
retinol dehydrogenase 1 (all trans)	Rdh1	NM_080436	3.2
family with sequence similarity 83, member F	Fam83f	NM_145986	3.2
predicted gene 10785	Gm10785	ENSMUST00000114029	3.2
GLI-Kruppel family member GLI1	Gli1	NM_010296	3.2

Additional Table 2b. Genderwise differentially expressed genes (p ≤0.01) with a regulation of ≥ 1.5

DAVID GeneName	Gene Symbol	RefSeq	Fold Change Female/Males
RIKEN cDNA 1700030J22 gene	1700030J22Rik	BC100501	3.1
cyclic nucleotide gated channel beta 1	Cngb1	BC045114	3.1
GTP binding protein 2	Gtpbp2	NM_019581	3.1
acyl-coenzyme A amino acid N-acyltransferase 2	Acnat2	NM_145368	3.1
trichoplein, keratin filament binding	Tchp	NM_029992	3.1
carboxypeptidase Z	Cpz	NM_153107	3.1
RIKEN cDNA 4930564C03 gene	4930564C03Rik	ENSMUST00000052701	3.1
cyclic nucleotide gated channel alpha 3	Cnga3	NM_009918	3.1
glycerol kinase-like 1	Gykl1	NM_010293	3.1
contactin 2	Cntn2	NM_177129	3.1
inter-alpha trypsin inhibitor, heavy chain 3	Itih3	NM_008407	3.1
threonine aldolase 1	Tha1	NM_027919	3.1
Bcl2-like 10	Bcl2l10	NM_013479	3.1
family with sequence similarity 176, member B	Fam176b	BC029204	3.1
Gm4833	Gm4833	XR_031272	3.1
toll-like receptor adaptor molecule 1	Ticam1	NM_174989	3.1
discs, large (Drosophila) homolog-associated protein 5	Dlgap5	NM_144553	3.1
Unknown	BC027582	BC027582	3.1
epithelial membrane protein 3	Emp3	NM_010129	3.1
death-associated protein kinase 2	Dapk2	NM_010019	3.1
WD repeat domain 63	Wdr63	NM_172864	3.0
ceroid lipofuscinosis, neuronal 3, juvenile (Batten, Spielmeyer-Vogt disease)	Cln3	NM_001146311	3.0
vomeronasal 2, receptor 110	Vmn2r110	NM_001104572	3.0
RIKEN cDNA 9430015G10 gene	9430015G10Rik	NM_177205	3.0
bruno-like 4, RNA binding protein (Drosophila)	Brunol4	NM_001146292	3.0
mucin 6, gastric	Muc6	NM_181729	3.0
glucagon receptor	Gcgr	NM_008101	3.0
dynein, axonemal, heavy chain 2	Dnahc2	NM_001081330	3.0
reproductive homeobox 12	Rhox12	NM_001025083	3.0
receptor-interacting serine-threonine kinase 4	Ripk4	NM_023663	3.0
acyl-CoA thioesterase 3	Acot3	NM_134246	3.0
olfactory marker protein	Omp	NM_011010	3.0
RIKEN cDNA 4930471G03 gene	4930471G03Rik	NR_015571	3.0
RNA binding motif protein 4	Rbm4	NM_009032	3.0
lanosterol synthase	Lss	NM_146006	3.0
ethanol induced 1	Etohi1	ENSMUST00000098999	3.0
grainyhead-like 1 (Drosophila)	Grhl1	NM_001161406	3.0
RAB4B, member RAS oncogene family	Rab4b	NM_029391	3.0
tyrosine aminotransferase	Tat	NM_146214	3.0
FERM domain containing 7	Frmd7	ENSMUST00000060650	3.0
cysteine-rich protein 3	Crip3	NM_181664	3.0
inhibitor of DNA binding 4	Id4	NM_031166	3.0
a disintegrin and metallopeptidase domain 3 (cyritestin)	Adam3	NM_009619	3.0
predicted gene 1467	Gm1467	XM_357906	3.0
crumbs homolog 1 (Drosophila)	Crb1	NM_133239	3.0
Unknown	C130092O11Rik	AK173231	3.0
bromodomain and WD repeat domain containing 3	Brwd3	NM_001081477	3.0
RIKEN cDNA E230008N13 gene	E230008N13Rik	ENSMUST00000043958	3.0
PARK2 co-regulated	Pacrg	NM_027032	3.0
DSN1, MIND kinetochore complex component, homolog (S. cerevisiae)	Dsn1	NM_025853	3.0

Additional Table 2b. Genderwise differentially expressed genes (p ≤0.01) with a regulation of ≥ 1.5

DAVID GeneName	Gene Symbol	RefSeq	Fold Change Female/Males
**Relatively upregulated in females ≥2.5 - fold**			
interleukin 17F	Il17f	NM_145856	2.9
angiotensin I converting enzyme (peptidyl-dipeptidase A) 2	Ace2	NM_001130513	2.9
retinol dehydrogenase 7	Rdh7	NM_001150749	2.9
human immunodeficiency virus type I enhancer binding protein 3	Hivep3	NM_010657	2.9
RIKEN cDNA 2900011O08 gene	2900011O08Rik	BC022741	2.9
protein tyrosine phosphatase, non-receptor type 13	Ptpn13	NM_011204	2.9
RIKEN cDNA 1700028J19 gene	1700028J19Rik	ENSMUST00000037220	2.9
dihydropyrimidinase	Dpys	NM_022722	2.9
Parkinson disease 7 domain containing 1	Pddc1	NM_172116	2.9
reprimo, TP53 dependent G2 arrest mediator candidate	Rprm	NM_023396	2.9
MAM domain containing 4	Mamdc4	NM_001081199	2.9
receptor (TNFRSF)-interacting serine-threonine kinase 2	Ripk2	NM_138952	2.9
zinc finger, DHHC domain containing 6	Zdhhc6	NM_001033573	2.9
ral guanine nucleotide dissociation stimulator-like 3	Rgl3	NM_023622	2.9
IQ motif containing G	Iqcg	NM_178378	2.9
B-cell leukemia/lymphoma 3	Bcl3	NM_033601	2.9
seminal vesicle antigen-like 2	Sval2	NM_032542	2.9
RIKEN cDNA 1700029F09 gene	1700029F09Rik	BC119032	2.9
predicted gene 9888	Gm9888	ENSMUST00000064735	2.9
junctional sarcoplasmic reticulum protein 1	Jsrp1	NM_028001	2.9
distal-less homeobox 6	Dlx6	NM_010057	2.9
pleckstrin and Sec7 domain containing 2	Psd2	NM_028707	2.9
Ca2+-dependent secretion activator	Cadps	NM_012061	2.9
NADH-ubiquinone oxidoreductase chain 4L	ND4L	ENSMUST00000084013	2.9
chemokine (C-X-C motif) ligand 10	Cxcl10	NM_021274	2.9
transmembrane protein 60	Tmem60	NM_177601	2.9
ropporin, rhophilin associated protein 1	Ropn1	NM_030744	2.9
Unknown	Fam38b2	ENSMUST00000046860	2.9
matrix metallopeptidase 8	Mmp8	NM_008611	2.9
gastrokine 1	Gkn1	NM_025466	2.9
phospholipase D family, member 5	Pld5	NM_176916	2.9
transmembrane protein 184a	Tmem184a	NM_001161548	2.9
ubiquilin 3	Ubqln3	NM_198623	2.8
predicted gene 10535	Gm10535	ENSMUST00000097567	2.8
zinc finger and BTB domain containing 5	Zbtb5	NM_173399	2.8
kelch-like 25 (Drosophila)	Klhl25	NM_029652	2.8
chromatin modifying protein 4C	Chmp4c	NM_025519	2.8
ELOVL family member 6, elongation of long chain fatty acids (yeast)	Elovl6	BC098492	2.8
homeo box A2	Hoxa2	NM_010451	2.8
napsin A aspartic peptidase	Napsa	NM_008437	2.8
defensin beta 23	Defb23	NM_001037933	2.8
CD33 antigen	Cd33	AK046303	2.8
potassium channel tetramerisation domain containing 19	Kctd19	NM_177791	2.8
RAS-like, family 11, member A	Rasl11a	NM_026864	2.8
predicted gene 7582	Gm7582	XM_979163	2.8
WNK lysine deficient protein kinase 3	Wnk3	ENSMUST00000096285	2.8
proline rich 5 like	Prr5l	NM_001083810	2.8
predicted gene 6577	Gm6577	NM_001037924	2.8

Additional Table 2b. Genderwise differentially expressed genes (p ≤0.01) with a regulation of ≥ 1.5

DAVID GeneName	Gene Symbol	RefSeq	Fold Change Female/Males
CKLF-like MARVEL transmembrane domain containing 5	Cmtm5	NM_026066	2.8
RIKEN cDNA 2810039B14 gene	2810039B14Rik	XM_001481291	2.8
RIKEN cDNA E130114P18 gene	E130114P18Rik	ENSMUST00000107065	2.8
GPI anchored molecule like protein	Gml	ENSMUST00000089693	2.8
RIKEN cDNA 0610010O12 gene	0610010O12Rik	BC028765	2.8
RIKEN cDNA A430072C10 gene	A430072C10Rik	AK040169	2.8
complement component 1, r subcomponent-like	C1rl	NM_181344	2.8
calcium channel, voltage-dependent, beta 3 subunit	Cacnb3	NM_007581	2.8
breast cancer 1	Brca1	NM_009764	2.8
Unknown	4833430A08Rik	AK132601	2.8
T-cell acute lymphocytic leukemia 1	Tal1	ENSMUST00000030489	2.8
TD and POZ domain containing 1	Tdpoz1	NM_148949	2.8
zinc finger, DHHC domain containing 23	Zdhhc23	NM_001007460	2.8
myogenic differentiation 1	Myod1	NM_010866	2.8
inhibitor of growth family, member 2	Ing2	NM_023503	2.8
predicted gene 14149	Gm14149	ENSMUST00000087474	2.8
platelet-activating factor receptor	Ptafr	NM_001081211	2.8
predicted gene 1082	Gm1082	BC065044	2.8
cDNA sequence BC031441	BC031441	BC094043	2.8
interaction protein for cytohesin exchange factors 1	Ipcef1	NM_001033391	2.8
zinc finger protein 707	Zfp707	NM_001081065	2.7
coagulation factor XIII, beta subunit	F13b	NM_031164	2.7
EDAR (ectodysplasin-A receptor)-associated death domain	Edaradd	NM_133643	2.7
predicted gene 8304	Gm8304	XM_001477007	2.7
MYST histone acetyltransferase 1	Myst1	NM_026370	2.7
predicted gene 7372	Gm7372	XR_031550	2.7
RIKEN cDNA 2610017I09 gene	2610017I09Rik	NR_027826	2.7
CUB and Sushi multiple domains 3	Csmd3	NM_001081391	2.7
RIKEN cDNA 1700007G11 gene	1700007G11Rik	BC049770	2.7
pleckstrin and Sec7 domain containing 4	Psd4	NM_177611	2.7
expressed sequence AI593442	AI593442	NM_178906	2.7
arrestin domain containing 5	Arrdc5	NM_029799	2.7
keratin 9	Krt9	NM_201255	2.7
Unknown	Gm9568	XR_032493	2.7
forkhead box B2	Foxb2	NM_008023	2.7
ATP-binding cassette, sub-family C (CFTR/MRP), member 3	Abcc3	NM_029600	2.7
mannose-binding lectin (protein A) 1	Mbl1	NM_010775	2.7
carcinoembryonic antigen-related cell adhesion molecule 12	Ceacam12	NM_026087	2.6
chromodomain protein, Y chromosome-like	Cdyl	NM_009881	2.6
gulonolactone (L-) oxidase	Gulo	NM_178747	2.6
tripartite motif-containing 50	Trim50	NM_178240	2.6
BCL2-like 2	Bcl2l2	NM_007537	2.6
hedgehog interacting protein-like 1	Hhipl1	NM_001044380	2.6
IAP promoted placental gene	Ipp	NM_008389	2.6
PDZK1 interacting protein 1	Pdzk1ip1	NM_026018	2.6
CD200 receptor 3	Cd200r3	NM_001128132	2.6
predicted gene 5087	Gm5087	NM_177813	2.6
Unknown	5033413D22Rik	ENSMUST00000019955	2.6
anterior gradient homolog 3 (Xenopus laevis)	Agr3	ENSMUST00000042101	2.6
E2F transcription factor 7	E2f7	NM_178609	2.6

Additional Table 2b. Genderwise differentially expressed genes (p ≤0.01) with a regulation of ≥ 1.5

DAVID GeneName	Gene Symbol	RefSeq	Fold Change Female/Males
Unknown	Gm12528	ENSMUST00000107544	2.6
coiled coil domain containing 28B	Ccdc28b	AK031278	2.6
tripartite motif-containing 31	Trim31	NM_146077	2.6
glycoprotein hormones, alpha subunit	Cga	NM_009889	2.6
tet oncogene family member 3	Tet3	ENSMUST00000056191	2.6
membrane-spanning 4-domains, subfamily A, member 2	Ms4a2	NM_013516	2.6
kallikrein 1-related peptidase b27	Klk1b27	NM_020268	2.6
RIKEN cDNA 1810063B05 gene	1810063B05Rik	NM_174987	2.6
polymerase (DNA-directed), delta 4	Pold4	NM_027196	2.6
phospholipase C, delta 3	Plcd3	NM_152813	2.6
Myb-related transcription factor, partner of profilin	Mypop	NM_145579	2.6
thrombopoietin	Thpo	NM_009379	2.6
Unknown	LOC100045342	XR_030763	2.6
cation channel, sperm associated 1	Catsper1	NM_139301	2.6
solute carrier family 14 (urea transporter), member 1	Slc14a1	NM_028122	2.6
RIKEN cDNA 1700028K03 gene	1700028K03Rik	NM_182745	2.6
family with sequence similarity 65, member C	Fam65c	BC130272	2.6
thymidine kinase 1	Tk1	NM_009387	2.6
predicted gene 15623	Gm15623	AK037770	2.6
glucose-fructose oxidoreductase domain containing 2	Gfod2	NM_027469	2.6
Unknown	LOC100045547	XM_001474499	2.6
potassium channel, subfamily V, member 2	Kcnv2	NM_183179	2.6
katanin p60 subunit A-like 2	Katnal2	BC056963	2.6
solute carrier family 35, member F1	Slc35f1	NM_178675	2.6
catechol-O-methyltransferase domain containing 1	Comtd1	NM_026965	2.6
homeo box B8	Hoxb8	NM_010461	2.5
Williams-Beuren syndrome chromosome region 16 homolog (human)	Wbscr16	NM_033572	2.5
guanylate cyclase 1, soluble, beta 2	Gucy1b2	NM_172810	2.5
ADAMTS-like 4	Adamtsl4	NM_144899	2.5
cytochrome P450, family 26, subfamily c, polypeptide 1	Cyp26c1	BC151098	2.5
CUB domain containing protein 2	Cdcp2	NM_172873	2.5
coiled-coil domain containing 39	Ccdc39	NM_026222	2.5
armadillo repeat containing 4	Armc4	NM_001081393	2.5
pleckstrin homology-like domain, family A, member 2	Phlda2	NM_009434	2.5
aminoadipate-semialdehyde dehydrogenase-phosphopantetheinyl transferase	Aasdhppt	NM_026276	2.5
phospholipase C, eta 2	Plch2	NM_175556	2.5
pirin	Pir	NM_027153	2.5
glutamate receptor, ionotropic, kainate 3	Grik3	NM_001081097	2.5
WD repeat domain 49	Wdr49	XM_143339	2.5
GAR1 ribonucleoprotein homolog (yeast)	Gar1	NM_026578	2.5
leucine rich repeat containing 46	Lrrc46	NM_027026	2.5
Musashi homolog 1(Drosophila)	Msi1	NM_008629	2.5
cornichon homolog 2 (Drosophila)	Cnih2	NM_009920	2.5
predicted gene 757	Gm757	BC147426	2.5
family with sequence similarity 129, member C	Fam129c	ENSMUST00000073799	2.5
RIKEN cDNA A630038E17 gene	A630038E17Rik	BC111819	2.5
deubiquitinating enzyme 1	Dub1	NM_007887	2.5
RIKEN cDNA 1700040I03 gene	1700040I03Rik	BC115452	2.5
fibroblast growth factor 4	Fgf4	NM_010202	2.5

Additional Table 2b. Genderwise differentially expressed genes (p ≤0.01) with a regulation of ≥ 1.5

DAVID GeneName	Gene Symbol	RefSeq	Fold Change Female/Males
contactin associated protein-like 5B	Cntnap5b	NM_172851	2.5
calcium channel, voltage-dependent, gamma subunit 7	Cacng7	NM_133189	2.5
WD repeat domain 16	Wdr16	NM_027963	2.5
serine (or cysteine) peptidase inhibitor, clade B (ovalbumin), member 12	Serpinb12	NM_027971	2.5
epithelial splicing regulatory protein 1	Esrp1	NM_194055	2.5
serine (or cysteine) peptidase inhibitor, clade A, member 3B	Serpina3b	NM_173024	2.5
zinc finger, FYVE domain containing 28	Zfyve28	BC042774	2.5
nudix (nucleoside diphosphate linked moiety X)-type motif 18	Nudt18	BC036718	2.5
NudC domain containing 2	Nudcd2	NM_026023	2.5
tumor necrosis factor receptor superfamily, member 11b (osteoprotegerin)	Tnfrsf11b	NM_008764	2.5
RIKEN cDNA 2610301B20 gene	2610301B20Rik	NM_026005	2.5
kinesin family member 17	Kif17	NM_010623	2.5
nicotinamide nucleotide adenylyltransferase 2	Nmnat2	NM_175460	2.5
RIKEN cDNA 3110070M22 gene	3110070M22Rik	NR_027974	2.5
SRY-box containing gene 5	Sox5	NM_011444	2.5
cyclin G2	Ccng2	NM_007635	2.5
tachykinin receptor 3	Tacr3	NM_021382	2.5
hepatitis A virus cellular receptor 2	Havcr2	NM_134250	2.5
phosphodiesterase 3B, cGMP-inhibited	Pde3b	NM_011055	2.5
engulfment and cell motility 3, ced-12 homolog (C. elegans)	Elmo3	NM_172760	2.5
RIKEN cDNA 2700097O09 gene	2700097O09Rik	BC056967	2.5
aquaporin 2	Aqp2	NM_009699	2.5
GULP, engulfment adaptor PTB domain containing 1	Gulp1	NM_028450	2.5
mitogen-activated protein kinase kinase kinase 9	Map3k9	ENSMUST00000035987	2.5
protein phosphatase 1J	Ppm1j	NM_027982	2.5
predicted gene 10742	Gm10742	ENSMUST00000099198	2.5
cytidine deaminase	Cda	NM_028176	2.5
achaete-scute complex homolog 1 (Drosophila)	Ascl1	NM_008553	2.5
potassium inwardly-rectifying channel, subfamily J, member 10	Kcnj10	NM_001039484	2.5
immunoglobulin superfamily, member 9B	Igsf9b	NM_001129787	2.5
**Relatively upregulated in females ≥2-fold**			
chromobox homolog 8 (Drosophila Pc class)	Cbx8	NM_013926	2.4
expressed sequence AU017193	AU017193	ENSMUST00000088076	2.4
DNA segment, Chr 1, Pasteur Institute 1	D1Pas1	NM_033077	2.4
retinol dehydrogenase 14 (all-trans and 9-cis)	Rdh14	NM_023697	2.4
phosphoprotein associated with glycosphingolipid microdomains 1	Pag1	NM_053182	2.4
expressed sequence AW822252	AW822252	AK082553	2.4
dual-specificity tyrosine-(Y)-phosphorylation regulated kinase 3	Dyrk3	NM_145508	2.4
predicted gene 7691	Gm7691	XM_001473789	2.4
CKLF-like MARVEL transmembrane domain containing 8	Cmtm8	NM_027294	2.4
predicted gene 4977	Gm4977	XR_032517	2.4
meiosis-specific nuclear structural protein 1	Mns1	NM_008613	2.4
UDP-Gal:betaGlcNAc beta 1,4-galactosyltransferase, polypeptide 3	B4galt3	NM_020579	2.4
general transcription factor II A, 2	Gtf2a2	NM_001039519	2.4
RIKEN cDNA 1700013D24 gene	1700013D24Rik	BC120816	2.4
sorting nexin 10	Snx10	NM_028035	2.4
predicted gene 4063	Gm4063	XR_034248	2.4
slit homolog 1 (Drosophila)	Slit1	NM_015748	2.4
RIKEN cDNA 1810007M14 gene	1810007M14Rik	NM_026110	2.4
retinaldehyde binding protein 1-like 1	Rlbp1l1	NM_028940	2.4

Additional Table 2b. Genderwise differentially expressed genes (p ≤0.01) with a regulation of ≥ 1.5

DAVID GeneName	Gene Symbol	RefSeq	Fold Change Female/Males
polymerase (DNA directed), iota	Poli	NM_011972	2.4
PITPNM family member 3	Pitpnm3	NM_001024927	2.4
forkhead box G1	Foxg1	ENSMUST00000110746	2.4
neuronatin	Nnat	AB004048	2.4
solute carrier family 6 (neurotransmitter transporter, dopamine), member 3	Slc6a3	NM_010020	2.4
acylphosphatase 2, muscle type	Acyp2	NM_029344	2.4
CD8 antigen, alpha chain	Cd8a	NM_001081110	2.4
RIKEN cDNA E230019M04 gene	E230019M04Rik	BC119078	2.4
RIKEN cDNA 2810021G02 gene	2810021G02Rik	NM_001162922	2.4
tumor rejection antigen P1A	Trap1a	NM_011635	2.4
chromodomain helicase DNA binding protein 1-like	Chd1l	NM_026539	2.4
glycine C-acetyltransferase (2-amino-3-ketobutyrate-coenzyme A ligase)	Gcat	NM_013847	2.4
G protein-coupled receptor 179	Gpr179	NM_001081220	2.4
growth differentiation factor 11	Gdf11	NM_010272	2.4
calcium channel, voltage-dependent, P/Q type, alpha 1A subunit	Cacna1a	NM_007578	2.4
triggering receptor expressed on myeloid cells 1	Trem1	NM_021406	2.4
ES cell-expressed Ras	Eras	NM_181548	2.4
RIKEN cDNA 5830418K08 gene	5830418K08Rik	NM_176976	2.4
ATP synthase, H+ transporting, mitochondrial F0 complex, subunit s	Atp5s	NM_026536	2.4
chloride channel calcium activated 5	Clca5	NM_178697	2.4
netrin G1	Ntng1	NM_030699	2.4
nuclear RNA export factor 2	Nxf2	NM_031259	2.4
RIKEN cDNA C130046B21 gene	C130046B21Rik	AK048278	2.4
decay accelerating factor 2	Daf2	NM_007827	2.4
ABO blood group (transferase A, alpha 1-3-N-acetylgalactosaminyltransferase, transferase B, alpha 1-3-galactosyltransferase)	Abo	NM_030718	2.4
transmembrane 4 L six family member 20	Tm4sf20	NM_025453	2.4
Wiskott-Aldrich syndrome homolog (human)	Was	NM_009515	2.4
RIKEN cDNA 2010109I03 gene	2010109I03Rik	NM_025929	2.3
ankyrin repeat and SOCS box-containing 9	Asb9	NM_027027	2.3
sciellin	Scel	NM_022886	2.3
DnaJ (Hsp40) homolog, subfamily C, member 17	Dnajc17	NM_139139	2.3
ribonuclease P 14 subunit (human)	Rpp14	NM_025938	2.3
NIPA-like domain containing 4	Nipal4	NM_172524	2.3
suppressor of fused homolog (Drosophila)	Sufu	NM_015752	2.3
predicted gene 5746	Gm5746	XR_034898	2.3
cDNA sequence BC031781	BC031781	BC031781	2.3
RIKEN cDNA B430212C06 gene	B430212C06Rik	BC028804	2.3
TAF13 RNA polymerase II, TATA box binding protein (TBP)-associated factor	Taf13	NM_025444	2.3
integral membrane protein 2A	Itm2a	NM_008409	2.3
adrenomedullin	Adm	NM_009627	2.3
potassium inwardly-rectifying channel, subfamily J, member 6	Kcnj6	NM_001025584	2.3
mucin 20	Muc20	NM_001145874	2.3
endothelin converting enzyme-like 1	Ecel1	NM_021306	2.3
proprotein convertase subtilisin/kexin type 9	Pcsk9	NM_153565	2.3
spermatogenesis associated 22	Spata22	NM_001045531	2.3
sterile alpha motif domain containing 7	Samd7	NM_029489	2.3
G protein-coupled receptor kinase 4	Grk4	NM_019497	2.3

Additional Table 2b. Genderwise differentially expressed genes (p ≤0.01) with a regulation of ≥ 1.5

DAVID GeneName	Gene Symbol	RefSeq	Fold Change Female/Males
peptidyl-tRNA hydrolase 2	Ptrh2	NM_175004	2.3
RIKEN cDNA 4922505E12 gene	4922505E12Rik	NM_001033547	2.3
GINS complex subunit 3 (Psf3 homolog)	Gins3	NM_030198	2.3
fibroblast growth factor 17	Fgf17	NM_008004	2.3
sema domain, immunoglobulin domain (Ig), TM domain, and short cytoplasmic domain	Sema4f	NM_011350	2.3
predicted gene 885	Gm885	NM_001033435	2.3
family with sequence similarity 100, member B	Fam100b	NM_176902	2.3
Unknown	3100002L24Rik	U62393	2.3
phosphatidylinositol glycan anchor biosynthesis, class H	Pigh	NM_029988	2.3
fusion, derived from t(12; 16) malignant liposarcoma (human)	Fus	NM_139149	2.3
myosin, heavy polypeptide 4, skeletal muscle	Myh4	NM_010855	2.3
predicted gene 8565	Gm8565	XR_032570	2.3
mitogen-activated protein kinase kinase kinase kinase 1	Map4k1	NM_008279	2.3
SV2 related protein	Svop	NM_026805	2.3
heat-responsive protein 12	Hrsp12	NM_008287	2.3
DnaJ (Hsp40) related, subfamily B, member 13	Dnajb13	NM_153527	2.3
neurensin 1	Nrsn1	NM_009513	2.3
RIKEN cDNA 9430002A10 gene	9430002A10Rik	ENSMUST00000074808	2.3
predicted gene 1043	Gm1043	ENSMUST00000094840	2.3
poly(rC) binding protein 1	Pcbp1	NM_011865	2.3
solute carrier family 35, member F2	Slc35f2	NM_028060	2.3
potassium channel, subfamily U, member 1	Kcnu1	NM_008432	2.3
T-cell lymphoma invasion and metastasis 2	Tiam2	NM_011878	2.3
ataxin 7-like 2	Atxn7l2	NM_175183	2.3
solute carrier family 28 (sodium-coupled nucleoside transporter), member 3	Slc28a3	NM_022317	2.3
GATA binding protein 2	Gata2	NM_008090	2.3
hydrocephalus inducing	Hydin	NM_172916	2.3
5-hydroxytryptamine (serotonin) receptor 3A	Htr3a	NM_013561	2.3
ATP-binding cassette, sub-family A (ABC1), member 13	Abca13	NM_178259	2.3
integrin beta 6	Itgb6	NM_001159564	2.3
neurotrophin 5	Ntf5	NM_198190	2.3
RIKEN cDNA 4930435E12 gene	4930435E12Rik	BC115688	2.3
mature T-cell proliferation 1	Mtcp1	NM_010839	2.3
cathepsin 3	Cts3	NM_026906	2.3
kit oncogene	Kit	NM_001122733	2.3
ribonuclease, RNase A family, 1 (pancreatic)	Rnase1	NM_011271	2.3
tetratricopeptide repeat domain 26	Ttc26	NM_153600	2.3
ankyrin repeat domain 45	Ankrd45	BC049713	2.3
TNFAIP3 interacting protein 3	Tnip3	NM_001001495	2.3
asparagine-linked glycosylation 11 homolog (yeast, alpha-1,2-mannosyltransferase)	Alg11	NM_183142	2.3
phosducin-like	Pdcl	NM_026176	2.3
RIKEN cDNA 4921507P07 gene	4921507P07Rik	BC055110	2.3
RAB13, member RAS oncogene family	Rab13	NM_026677	2.3
RIKEN cDNA 2300009A05 gene	2300009A05Rik	NM_027090	2.3
FIP1 like 1 (S. cerevisiae)	Fip1l1	NM_001159573	2.3
PR domain containing 6	Prdm6	NM_001033281	2.3
double C2, beta	Doc2b	NM_007873	2.3
small nuclear ribonucleoprotein E	Snrpe	NM_009227	2.2

Additional Table 2b. Genderwise differentially expressed genes (p ≤0.01) with a regulation of ≥ 1.5

DAVID GeneName	Gene Symbol	RefSeq	Fold Change Female/Males
tyrosine 3-monooxygenase/tryptophan 5-monooxygenase activation protein, thet	Ywhaq	NM_011739	2.2
RIKEN cDNA 4932414N04 gene	4932414N04Rik	NM_183113	2.2
a disintegrin-like and metallopeptidase (reprolysin type) with thrombospondin type 1 motif, 19	Adamts19	NM_175506	2.2
interferon, alpha-inducible protein 27 like 1	Ifi27l1	NM_026790	2.2
sodium channel, nonvoltage-gated 1 gamma	Scnn1g	NM_011326	2.2
leucine rich repeat containing 28	Lrrc28	NM_175124	2.2
Unknown	'MUSG00000016609	NM_001100416	2.2
Unknown	100043387	NM_001099327	2.2
RIKEN cDNA 1700066J24 gene	1700066J24Rik	ENSMUST00000038960	2.2
histidine triad nucleotide binding protein 1	Hint1	NM_008248	2.2
BUD13 homolog (yeast)	Bud13	NM_146000	2.2
a disintegrin-like and metallopeptidase (reprolysin type) with thrombospondin type 1 motif, 4	Adamts4	NM_172845	2.2
mitochondrial ribosomal protein L47	Mrpl47	NM_029017	2.2
glucosamine-phosphate N-acetyltransferase 1	Gnpnat1	NM_019425	2.2
arrestin domain containing 2	Arrdc2	NM_027560	2.2
retina and anterior neural fold homeobox	Rax	NM_013833	2.2
hairy and enhancer of split 1 (Drosophila)	Hes1	NM_008235	2.2
esterase 1	Es1	NM_007954	2.2
gap junction protein, beta 3	Gjb3	NM_008126	2.2
solute carrier family 17 (anion/sugar transporter), member 5	Slc17a5	NM_172773	2.2
mucin 4	Muc4	NM_080457	2.2
gamma-aminobutyric acid (GABA) A receptor, subunit theta	Gabrq	NM_020488	2.2
kinesin family member 5A	Kif5a	NM_008447	2.2
myosin, light polypeptide 6B	Myl6b	NM_172259	2.2
complement factor H-related 1	Cfhr1	NM_015780	2.2
diacylglycerol kinase, gamma	Dgkg	NM_138650	2.2
RIKEN cDNA 1700084C06 gene	1700084C06Rik	AK006986	2.2
insulin-like growth factor binding protein-like 1	Igfbpl1	NM_018741	2.2
RIKEN cDNA 6330416L07 gene	6330416L07Rik	NM_176962	2.2
ectodysplasin-A receptor	Edar	NM_010100	2.2
R-spondin homolog (Xenopus laevis)	Rspo1	NM_138683	2.2
predicted gene 5460	Gm5460	NM_001034880	2.2
T-box 1	Tbx1	NM_011532	2.2
predicted gene 5064; cold shock domain containing E1, RNA binding	Gm5064	XR_032683	2.2
acylphosphatase 1, erythrocyte (common) type	Acyp1	NM_025421	2.2
zinc finger and BTB domain containing 6	Zbtb6	NM_146253	2.2
RIKEN cDNA 4833412L08 gene	4833412L08Rik	ENSMUST00000091218	2.2
coiled-coil domain containing 66	Ccdc66	NM_177111	2.2
centromere protein J	Cenpj	NM_001014996	2.2
F-box protein 48	Fbxo48	NM_176982	2.2
ATPase family, AAA domain containing 4	Atad4	NM_146026	2.2
lipocalin 5	Lcn5	NM_007947	2.2
target of EGR1, member 1 (nuclear)	Toe1	NM_026654	2.2
potassium voltage-gated channel, shaker-related subfamily, beta member 1	Kcnab1	ENSMUST00000049230	2.2
kelch-like 28 (Drosophila)	Klhl28	NM_025707	2.2
predicted gene 10648	Gm10648	ENSMUST00000098619	2.2
transmembrane protease, serine 5 (spinesin)	Tmprss5	NM_030709	2.2
NIMA (never in mitosis gene a)-related expressed kinase 2	Nek2	NM_010892	2.2

Additional Table 2b. Genderwise differentially expressed genes (p ≤0.01) with a regulation of ≥ 1.5

DAVID GeneName	Gene Symbol	RefSeq	Fold Change Female/Males
envoplakin	Evpl	NM_025276	2.2
glycosyltransferase 6 domain containing 1	Glt6d1	NM_001039095	2.2
sideroflexin 5	Sfxn5	NM_178639	2.2
ubiquitin specific peptidase 9, Y chromosome	Usp9y	NM_148943	2.2
acyl-CoA synthetase medium-chain family member 1	Acsm1	NM_054094	2.2
wingless-related MMTV integration site 7A	Wnt7a	NM_009527	2.2
apolipoprotein L 8	Apol8	BC129972	2.2
integrin beta 3	Itgb3	NM_016780	2.2
predicted gene 9493	Gm8560	XR_031229	2.2
coiled-coil domain containing 114	Ccdc114	ENSMUST00000117998	2.2
ATP-binding cassette, sub-family G (WHITE), member 3	Abcg3	NM_030239	2.2
predicted gene 14403	Gm14403	ENSMUST00000108947	2.2
F-box protein 16	Fbxo16	NM_015795	2.2
melanoma antigen family B, 18	Mageb18	NM_173783	2.2
outer dense fiber of sperm tails 3-like 1	Odf3l1	NM_198673	2.2
olfactory receptor 1427	Olfr1427	NM_146679	2.2
pleiotropic regulator 1, PRL1 homolog (Arabidopsis)	Plrg1	NM_016784	2.2
transmembrane emp24 protein transport domain containing	Tmed11	NM_026109	2.2
zinc finger protein 113	Zfp113	NM_019747	2.2
SRY-box containing gene 17	Sox17	NM_011441	2.2
transferrin receptor 2	Trfr2	NM_015799	2.2
RCC1 domain containing 1	Rccd1	NM_173445	2.2
polyhomeotic-like 2 (Drosophila)	Phc2	ENSMUST00000106080	2.2
transmembrane protein 80	Tmem80	NM_027797	2.2
DDRGK domain containing 1	Ddrgk1	NM_029832	2.2
signal recognition particle 14	Srp14	NM_009273	2.2
ring finger and CHY zinc finger domain containing 1	Rchy1	NM_026557	2.2
phosphoserine aminotransferase 1	Psat1	NM_177420	2.1
synemin, intermediate filament protein	Synm	ENSMUST00000056808	2.1
kelch-like 20 (Drosophila)	Klhl20	NM_001039482	2.1
predicted gene 6718	Gm6718	XM_905375	2.1
small nucleolar RNA, C/D box 8	Snord8	AF357382	2.1
BR serine/threonine kinase 2	Brsk2	NM_001009930	2.1
predicted gene 5893	Gm5893	ENSMUST00000073390	2.1
predicted gene 11961	Gm11961	NR_027798	2.1
asparagine-linked glycosylation 5 homolog (yeast, dolichyl-phosphate beta-glucosyltransferase)	Alg5	NM_025442	2.1
apolipoprotein B mRNA editing enzyme, catalytic polypeptide 1	Apobec1	NM_031159	2.1
chaperonin containing Tcp1, subunit 6b (zeta)	Cct6b	NM_009839	2.1
deleted in azoospermia-like	Dazl	NM_010021	2.1
solute carrier family 16 (monocarboxylic acid transporters), member 6	Slc16a6	NM_001029842	2.1
dynein, axonemal, light chain 1	Dnalc1	NM_028821	2.1
Rh blood group, D antigen	Rhd	NM_011270	2.1
predicted gene 5210	Gm5210	XR_031132	2.1
RIKEN cDNA E430018J23 gene	E430018J23Rik	BC010442	2.1
vestigial like 1 homolog (Drosophila)	Vgll1	NM_133251	2.1
sorting nexin 26	Snx26	NM_178252	2.1
cDNA sequence BC003267	BC003267	NM_145591	2.1
synaptogyrin 3	Syngr3	NM_011522	2.1
phosphopantothenoylcysteine decarboxylase	Ppcdc	NM_176831	2.1

Additional Table 2b. Genderwise differentially expressed genes (p ≤0.01) with a regulation of ≥ 1.5

DAVID GeneName	Gene Symbol	RefSeq	Fold Change Female/Males
fumarylacetoacetate hydrolase domain containing 1	Fahd1	NM_023480	2.1
protein tyrosine phosphatase, receptor type, F	Ptprf	NM_011213	2.1
suprabasin	Sbsn	NM_172205	2.1
SEC14-like 3 (S. cerevisiae)	Sec14l3	NM_001029937	2.1
protein C	Proc	NM_001042767	2.1
angiotensin II receptor, type 1b	Agtr1b	NM_175086	2.1
NADH dehydrogenase (ubiquinone) 1, subcomplex unknown, 2	Ndufc2	NM_024220	2.1
apoptosis-inducing factor, mitochondrion-associated 3	Aifm3	NM_175178	2.1
predicted gene 5294	Gm5294	XM_001000706	2.1
ferredoxin-fold anticodon binding domain containing 1	Fdxacb1	NM_198675	2.1
transmembrane protein 167	Tmem167	NM_025335	2.1
SEC16 homolog B (S. cerevisiae)	Sec16b	NR_027641	2.1
tweety homolog 1 (Drosophila)	Ttyh1	NM_021324	2.1
megakaryocyte-associated tyrosine kinase	Matk	NM_010768	2.1
RIKEN cDNA 2010016I18 gene	2010016I18Rik	AK140363	2.1
NFU1 iron-sulfur cluster scaffold homolog (S. cerevisiae)	Nfu1	NM_020045	2.1
signal recognition particle 9	Srp9	NM_012058	2.1
YTH domain family 3	Ythdf3	NR_027375	2.1
von Willebrand factor C and EGF domains	Vwce	NM_027913	2.1
NFKB activating protein-like	Nkapl	NM_025719	2.1
fucokinase	Fuk	NM_172283	2.1
mutS homolog 4 (E. coli)	Msh4	NM_031870	2.1
predicted gene 14127	1110005A03Rik	NM_028865	2.1
RIKEN cDNA 4930432O21 gene	4930432O21Rik	NM_001025373	2.1
SCO-spondin	Sspo	AJ491857	2.1
tyrosine kinase, non-receptor, 1	Tnk1	NM_031880	2.1
pappalysin 2	Pappa2	NM_001085376	2.1
RIKEN cDNA 2810055G20 gene	2810055G20Rik	AK148800	2.1
RIKEN cDNA A830010M20 gene	A830010M20Rik	AK122444	2.1
heterogeneous nuclear ribonucleoprotein M	Hnrnpm	NM_029804	2.1
rhomboid domain containing 3	Rhbdd3	NM_177370	2.1
predicted gene 6983	Gm6983	XR_033422	2.1
DEAD (Asp-Glu-Ala-Asp) box polypeptide 4	Ddx4	NM_001145885	2.1
expressed sequence AI597479	AI597479	BC006931	2.1
a disintegrin and metallopeptidase domain 28	Adam28	NM_010082	2.1
RIKEN cDNA A430078G23 gene	A430078G23Rik	NM_001033378	2.1
predicted gene 11733	Gm11733	ENSMUST00000092394	2.1
RASD family, member 2	Rasd2	NM_029182	2.1
DAZ interacting protein 1	Dzip1	NM_025943	2.1
pancreatic polypeptide	Ppy	NM_008918	2.1
C-type lectin domain family 4, member f	Clec4f	NM_016751	2.1
sialic acid binding Ig-like lectin G	Siglecg	NM_172900	2.1
interleukin 19	Il19	NM_001009940	2.1
RIKEN cDNA 5730409K12 gene	5730409K12Rik	AK134852	2.1
Unknown	2210418O10Rik	BC080301	2.1
calpain 12	Capn12	NM_001110807	2.1
cDNA sequence BC022687	BC022687	BC090976	2.1
UDP-GlcNAc:betaGal beta-1,3-N-acetylglucosaminyltransferase 4	B3gnt4	NM_198611	2.1
zinc finger protein 128	Zfp128	NM_153802	2.1
sulfotransferase family 1A, phenol-preferring, member 1	Sult1a1	NM_133670	2.1

Additional Table 2b. Genderwise differentially expressed genes (p ≤0.01) with a regulation of ≥ 1.5

DAVID GeneName	Gene Symbol	RefSeq	Fold Change Female/Males
regulator of G-protein signaling 3	Rgs3	NM_019492	2.1
small nuclear ribonucleoprotein 48 (U11/U12)	Snrnp48	NM_026382	2.1
G protein-coupled receptor 111	Gpr111	NM_001033493	2.1
RIKEN cDNA 8430419L09 gene	8430419L09Rik	BC137674	2.1
gene trap ROSA b-geo 22	Gtrgeo22	NM_148934	2.1
similar to CDCrel-1AI; septin 5	LOC100044138	XM_001480793	2.1
advillin	Avil	NM_009635	2.1
RIKEN cDNA 4922505G16 gene	4922505G16Rik	NM_001039557	2.1
cyclin-dependent kinase inhibitor 2C (p18, inhibits CDK4)	Cdkn2c	NM_007671	2.1
zinc finger, CCHC domain containing 13	Zcchc13	NM_029158	2.1
protein kinase, cGMP-dependent, type II	Prkg2	L12460	2.1
solute carrier family 13 (sodium-dependent dicarboxylate transporter), member 2	Slc13a2	NM_022411	2.1
coiled-coil domain containing 60	Ccdc60	ENSMUST00000086483	2.1
elastase 2, neutrophil	Ela2	NM_015779	2.1
coagulation factor II	F2	NM_010168	2.1
fibroblast growth factor 11	Fgf11	ENSMUST00000108637	2.1
coiled-coil domain containing 51	Ccdc51	NM_025689	2.0
discs, large homolog 4 (Drosophila)	Dlg4	NM_007864	2.0
serum/glucocorticoid regulated kinase 2	Sgk2	NM_013731	2.0
ubiquitin-conjugating enzyme E2C binding protein	Ube2cbp	NM_027394	2.0
RIKEN cDNA 1810032O08 gene	1810032O08Rik	NR_027821	2.0
predicted gene 447	Gm447	BC025881	2.0
tetratricopeptide repeat domain 32	Ttc32	NM_029321	2.0
predicted gene 9804	Gm9804	ENSMUST00000056234	2.0
prolyl 4-hydroxylase, transmembrane (endoplasmic reticulum)	P4htm	NM_028944	2.0
tetratricopeptide repeat domain 14	Ttc14	NM_025978	2.0
biogenesis of lysosome-related organelles complex-1, subunit 2	Bloc1s2	BC065806	2.0
RIKEN cDNA 4831426I19 gene	4831426I19Rik	NM_001042699	2.0
RIKEN cDNA 1700022A21 gene	1700022A21Rik	NR_003953	2.0
zinc finger, CCHC domain containing 9	Zcchc9	NM_145453	2.0
FCH and double SH3 domains 1	Fchsd1	NM_175684	2.0
gamma-glutamyl cyclotransferase	Ggct	NM_026637	2.0
CD72 antigen	Cd72	NM_001110320	2.0
solute carrier family 26, member 6	Slc26a6	NM_134420	2.0
double homeobox	Dux	NM_001081954	2.0
nuclear distribution gene C homolog (Aspergillus)	Nudc	NM_010948	2.0
electron transferring flavoprotein, alpha polypeptide	Etfa	NM_145615	2.0
queuine tRNA-ribosyltransferase domain containing 1	Qtrtd1	NM_029128	2.0
coiled-coil-helix-coiled-coil-helix domain containing 5	Chchd5	NM_025395	2.0
transmembrane and tetratricopeptide repeat containing 4	Tmtc4	NM_028651	2.0
Ras homolog enriched in brain	Rheb	NM_053075	2.0
cold inducible RNA binding protein	Cirbp	NM_007705	2.0
phosphodiesterase 11A	Pde11a	NM_001081033	2.0
lipoma HMGIC fusion partner-like 1	Lhfpl1	NM_178358	2.0
PHD finger protein 5A	Phf5a	NM_026737	2.0
CASP2 and RIPK1 domain containing adaptor with death domain	Cradd	ENSMUST00000099332	2.0
cytochrome b5 domain containing 2	Cyb5d2	NM_001024926	2.0
additional sex combs like 3 (Drosophila)	Asxl3	ENSMUST00000062608	2.0
acetylcholinesterase	Ache	NM_009599	2.0

Additional Table 2b. Genderwise differentially expressed genes (p ≤0.01) with a regulation of ≥ 1.5

DAVID GeneName	Gene Symbol	RefSeq	Fold Change Female/Males
ADP-ribosyltransferase 3	Art3	NM_181728	2.0
membrane-spanning 4-domains, subfamily A, member 15	Ms4a15	NM_001034898	2.0
DENN/MADD domain containing 2D	Dennd2d	NM_028110	2.0
galectin-related inter-fiber protein	Grifin	NM_030022	2.0
kelch-like 4 (Drosophila)	Klhl4	NM_172781	2.0
RRS1 ribosome biogenesis regulator homolog (S. cerevisiae)	Rrs1	NM_021511	2.0
crystallin, beta B1	Crybb1	NM_023695	2.0
solute carrier family 25 (mitochondrial carrier, phosphate carrier), member 26	Slc25a26	NM_026255	2.0
IMP1 inner mitochondrial membrane peptidase-like (S. cerevisiae)	Immp1l	NM_028260	2.0
ectodysplasin A2 isoform receptor	Eda2r	NM_001161432	2.0
blocked early in transport 1 homolog (S. cerevisiae)-like	Bet1l	NM_018742	2.0
carboxylesterase 1	Ces1	NM_021456	2.0
RAB9, member RAS oncogene family	Rab9	NM_019773	2.0
E2F transcription factor 8	E2f8	ENSMUST00000119223	2.0
PIF1 5'-to-3' DNA helicase homolog (S. cerevisiae)	Pif1	NM_172453	2.0
predicted gene 609	Gm609	BC054854	2.0
phosphodiesterase 6C, cGMP specific, cone, alpha prime	Pde6c	NM_033614	2.0
predicted gene 1070	Gm1070	BC147402	2.0
olfactory receptor 558	Olfr558	NM_147093	2.0
predicted gene 879	Shisa6	NM_001034874	2.0
RIKEN cDNA 1700084E18 gene	1700084E18Rik	BC062268	2.0
upper zone of growth plate and cartilage matrix associated	Ucma	NM_026754	2.0
Unknown	Gm7520	XR_032099	2.0
predicted gene 10762	Gm10762	ENSMUST00000099385	2.0
RIKEN cDNA 9430031J16 gene	9430031J16Rik	BC082310	2.0
coiled-coil domain containing 38	Ccdc38	NM_175488	2.0
RIKEN cDNA 1190007I07 gene	1190007I07Rik	NM_001135567	2.0
naked cuticle 2 homolog (Drosophila)	Nkd2	NM_028186	2.0
dedicator of cyto-kinesis 2	Dock2	NM_033374	2.0
clusterin associated protein 1	Cluap1	NM_029738	2.0
arylacetamide deacetylase (esterase)	Aadac	NM_023383	2.0
caspase 6	Casp6	NM_009811	2.0
growth factor receptor bound protein 2	Grb2	NM_008163	2.0
zona pellucida like domain containing 1	Zpld1	NM_178720	2.0
RIKEN cDNA B230104I21 gene	B230104I21Rik	AK140029	2.0
LSM8 homolog, U6 small nuclear RNA associated (S. cerevisiae)	Lsm8	NM_133939	2.0
PWP1 homolog (S. cerevisiae)	Pwp1	NM_133993	2.0
calcium-binding tyrosine-(Y)-phosphorylation regulated (fibrousheathin 2)	Cabyr	NM_027687	2.0
MAM domain containing 2	Mamdc2	NM_174857	2.0
RAB34, member of RAS oncogene family	Rab34	NM_033475	2.0
predicted gene 2173	Gm2173	XM_001472530	2.0
glycerol kinase	Gyk	NM_008194	2.0
paired related homeobox protein-like 1	Prrxl1	EU670677	2.0
RIKEN cDNA A930039A15 gene; zinc finger and BTB domain containing 6	A930039A15Rik	AK080751	2.0
olfactory receptor 1033	Olfr1033	NM_146578	2.0
WD repeat and FYVE domain containing 2	Wdfy2	NM_175546	2.0
cDNA sequence BC030867	BC030867	ENSMUST00000036548	2.0
diacylglycerol kinase, iota	Dgki	NM_001081206	2.0
RIKEN cDNA 1700013F07 gene	1700013F07Rik	BC116223	2.0

Additional Table 2b. Genderwise differentially expressed genes (p ≤0.01) with a regulation of ≥ 1.5

DAVID GeneName	Gene Symbol	RefSeq	Fold Change Female/Males
ATPase, Na+/K+ transporting, alpha 3 polypeptide	Atp1a3	NM_144921	2.0
inhibitor of DNA binding 3	Id3	NM_008321	2.0
pleckstrin homology domain containing, family M, member 3	Plekhm3	NM_001039493	2.0
programmed cell death 5	Pdcd5	NM_019746	2.0
adaptor protein complex AP-1, gamma 2 subunit	Ap1g2	NM_007455	2.0
histamine receptor H1	Hrh1	NM_008285	2.0
missing oocyte, meiosis regulator, homolog (Drosophila)	Mios	NM_145374	2.0
sclerostin	Sost	NM_024449	2.0
relaxin/insulin-like family peptide receptor 1	Rxfp1	NM_212452	2.0
tRNA-histidine guanylyltransferase 1-like (S. cerevisiae)	Thg1l	NM_001080969	2.0
carbonic anhydrase 5b, mitochondrial	Car5b	NM_181315	2.0
predicted gene 12696	Gm12696	XR_004941	2.0
prenylcysteine oxidase 1 like	Pcyox1l	NM_172832	2.0
echinoderm microtubule associated protein like 5	Eml5	NM_001081191	2.0
Unknown	2310051M13Rik	BC147423	2.0
coiled-coil domain containing 138	Ccdc138	NM_001162956	2.0
paired-like homeodomain transcription factor 2	Pitx2	NM_001042502	2.0
**Relatively upregulated in females ≥1.5-fold**			
EF hand calcium binding domain 1	Efcab1	ENSMUST00000090277	1.9
ATP/GTP binding protein-like 1	Agbl1	DQ867033	1.9
RIKEN cDNA 2510048L02 gene	2510048L02Rik	NM_025977	1.9
CD63 antigen	Cd63	NM_001042580	1.9
collagen, type VIII, alpha 2	Col8a2	NM_199473	1.9
methylenetetrahydrofolate dehydrogenase (NADP+ dependent) 2-like	Mthfd2l	NM_026788	1.9
C-type lectin domain family 4, member b2	Clec4b2	NM_001004159	1.9
RIKEN cDNA 6530409C15 gene	6530409C15Rik	ENSMUST00000060288	1.9
RIKEN cDNA 4933413N12 gene	4933413N12Rik	NM_001162901	1.9
transmembrane protein 191C	Tmem191c	NM_177473	1.9
RIKEN cDNA 2510049J12 gene	2510049J12Rik	NM_001101431	1.9
hydroxysteroid (17-beta) dehydrogenase 12	Hsd17b12	NM_019657	1.9
protein disulfide isomerase associated 5	Pdia5	NM_028295	1.9
insulin-like growth factor binding protein 3	Igfbp3	NM_008343	1.9
SRY-box containing gene 2	Sox2	NM_011443	1.9
hematological and neurological expressed sequence 1	Hn1	NM_008258	1.9
lysophosphatidylcholine acyltransferase 4	Lpcat4	NM_207206	1.9
RIKEN cDNA 0610007C21 gene	0610007C21Rik	NM_027855	1.9
RNA terminal phosphate cyclase domain 1	Rtcd1	NM_025517	1.9
phenylalanyl-tRNA synthetase, alpha subunit	Farsa	NM_025648	1.9
signal peptidase complex subunit 2 homolog (S. cerevisiae)	Spcs2	NM_025668	1.9
cyclin T2	Ccnt2	NM_028399	1.9
ST6 (alpha-N-acetyl-neuraminyl-2,3-beta-galactosyl-1,3)-N-acetylgalactosaminide alpha-2,6-sialyltransferase 1	St6galnac1	NM_011371	1.9
glucosamine-6-phosphate deaminase 2	Gnpda2	NM_001038015	1.9
uroporphyrinogen decarboxylase	Urod	NM_009478	1.9
guanine nucleotide binding protein, alpha 15	Gna15	NM_010304	1.9
cholinergic receptor, nicotinic, alpha polypeptide 3	Chrna3	NM_145129	1.9
nudix (nucleoside diphosphate linked moiety X)-type motif 15	Nudt15	NM_172527	1.9
adaptor-related protein complex AP-4, mu 1	Ap4m1	NM_021392	1.9
bol, boule-like (Drosophila)	Boll	NM_029267	1.9
phosphoglycerate mutase 1	Pgam1	NM_023418	1.9

Additional Table 2b. Genderwise differentially expressed genes (p ≤0.01) with a regulation of ≥ 1.5

DAVID GeneName	Gene Symbol	RefSeq	Fold Change Female/Males
zinc finger protein 872	Zfp872	NM_001033813	1.9
interleukin 17 receptor A	Il17ra	NM_008359	1.9
RIKEN cDNA 4933413J09 gene	4933413J09Rik	ENSMUST00000059648	1.9
ChaC, cation transport regulator homolog 2 (E. coli)	Chac2	NM_026527	1.9
calcium/calmodulin-dependent protein kinase II, beta	Camk2b	BC080273	1.9
transmembrane protein 126B	Tmem126b	NM_026734	1.9
proprotein convertase subtilisin/kexin type 1	Pcsk1	NM_013628	1.9
predicted gene 14964	Gm14964	ENSMUST00000063955	1.9
COX11 homolog, cytochrome c oxidase assembly protein (yeast)	Cox11	NM_199008	1.9
olfactory receptor 555	Olfr555	NM_147103	1.9
unc-50 homolog (C. elegans)	Unc50	NM_026123	1.9
sema domain, seven thrombospondin repeats (type 1 and type 1-like), transmembrane domain (TM) and short cytoplasmic domain, (semaphorin) 5B	Sema5b	BC052397	1.9
synaptojanin 2 binding protein	Cox16	ENSMUST00000002757	1.9
hypocretin (orexin) receptor 1	Hcrtr1	NM_198959	1.9
FERM domain containing 8	Frmd8	NM_026169	1.9
RAS guanyl releasing protein 1	Rasgrp1	NM_011246	1.9
predicted gene 14325	Gm14325	ENSMUST00000108939	1.9
phospholipase A2, group XV	Pla2g15	NM_133792	1.9
metallothionein 2	Mt2	NM_008630	1.9
tripartite motif-containing 62	Trim62	NM_178110	1.9
olfactory receptor 586	Olfr586	NM_147111	1.9
aggrecan	Acan	NM_007424	1.9
apoptotic peptidase activating factor 1	Apaf1	NM_001042558	1.9
predicted gene 6322	Gm6322	XR_031468	1.9
PR domain containing 11	Prdm11	ENSMUST00000111274	1.9
C1D nuclear receptor co-repressor	C1d	NM_020558	1.9
reproductive homeobox 3B	Rhox1	NM_001025084	1.9
churchill domain containing 1	Churc1	NM_206534	1.9
family with sequence similarity 174, member A	Fam174a	NM_026321	1.9
aldehyde dehydrogenase family 1, subfamily A3	Aldh1a3	NM_053080	1.9
RNA polymerase II associated protein 3	Rpap3	NM_028003	1.9
matrix metallopeptidase 16	Mmp16	NM_019724	1.9
predicted pseudogene 10072	Gm10072	ENSMUST00000076473	1.9
coiled-coil domain containing 117	Ccdc117	NM_134033	1.9
FERM and PDZ domain containing 1	Frmpd1	NM_001081172	1.9
tubulin tyrosine ligase-like family, member 10	Ttll10	NM_029264	1.9
transcription factor 19	Tcf19	NM_025674	1.9
RIKEN cDNA 1110058L19 gene	1110058L19Rik	NM_026503	1.9
heat shock protein family B (small), member 11	Hspb11	ENSMUST00000106749	1.9
retinitis pigmentosa 1 (human)	Rp1	NM_011283	1.9
prion protein dublet	Prnd	NM_023043	1.9
dynactin 3	Dctn3	NM_016890	1.9
zinc finger protein 52	Zfp52	NM_144515	1.9
RIKEN cDNA 6030429G01 gene	6030429G01Rik	BC150983	1.9
RIKEN cDNA 4930539E08 gene	4930539E08Rik	BC117930	1.9
RIKEN cDNA C030002C11 gene	C030002C11Rik	BC058715	1.9
proprotein convertase subtilisin/kexin type 4	Pcsk4	NM_008793	1.9
ATP-binding cassette, sub-family D (ALD), member 4	Abcd4	NM_008992	1.9

Additional Table 2b. Genderwise differentially expressed genes (p ≤0.01) with a regulation of ≥ 1.5

DAVID GeneName	Gene Symbol	RefSeq	Fold Change Female/Males
ras homolog gene family, member J	Rhoj	NM_023275	1.9
Eph receptor A3	Epha3	NM_010140	1.9
RIKEN cDNA 1700021F07 gene	1700021F07Rik	BC050790	1.9
carboxylesterase 7	Ces7	NM_001003951	1.9
selectin, platelet	Selp	NM_011347	1.9
lymphocyte antigen 6 complex, locus C2	Ly6c2	D86232	1.9
zinc finger protein 367	Zfp367	NM_175494	1.9
KiSS-1 metastasis-suppressor	Kiss1	NM_178260	1.9
phospholipase A2 receptor 1	Pla2r1	NM_008867	1.9
ring finger protein 5	Rnf5	NM_019403	1.9
CD3 antigen, epsilon polypeptide	Cd3e	NM_007648	1.9
mitochondrial ribosomal protein S14	Mrps14	NM_025474	1.9
dipeptidylpeptidase 6	Dpp6	NM_001136060	1.9
Src-like-adaptor 2	Sla2	NM_029983	1.9
RIKEN cDNA 2010003O02 gene	2010003O02Rik	ENSMUST00000030114	1.9
eukaryotic translation initiation factor 1B	Eif1b	NM_026892	1.9
glutathione S-transferase, theta 1	Gstt1	NM_008185	1.9
PQ loop repeat containing 1	Pqlc1	NM_025861	1.9
keratin associated protein 1-4	Krtap1-4	NM_001039502	1.9
cyclin-dependent kinase 5, regulatory subunit 1 (p35)	Cdk5r1	NM_009871	1.9
COX assembly mitochondrial protein homolog (S. cerevisiae)	Cmc1	NM_026442	1.9
Unknown	9130227C08Rik	BC116260	1.9
predicted gene 9866	Gm9866	ENSMUST00000063754	1.9
homeodomain interacting protein kinase 4	Hipk4	NM_001033315	1.9
THO complex 6 homolog (Drosophila)	Thoc6	NM_001008425	1.9
hyaluronan and proteoglycan link protein 2	Hapln2	NM_022031	1.9
zinc finger, BED domain containing 3	Zbed3	NM_028106	1.9
purinergic receptor P2X, ligand-gated ion channel, 6	P2rx6	NM_011028	1.9
RIKEN cDNA 4930438A08 gene	4930438A08Rik	ENSMUST00000108834	1.9
cysteine conjugate-beta lyase 2	Ccbl2	NM_173763	1.9
profilin 2	Pfn2	NM_019410	1.9
H2A histone family, member Y3	H2afy3	NR_003523	1.9
polymerase (DNA directed), alpha 1	Pola1	NM_008892	1.9
solute carrier family 45, member 2	Slc45a2	NM_053077	1.9
family with sequence similarity 96, member A	Fam96a	NM_026635	1.9
tripartite motif-containing 13	Trim13	NM_023233	1.9
RNA binding motif protein 3	Rbm3	NM_016809	1.9
motile sperm domain containing 2	Mospd2	NM_029730	1.9
SV2 related protein homolog (rat)-like	Svopl	NM_177200	1.9
cathepsin O	Ctso	NM_177662	1.9
ATPase, Cu++ transporting, beta polypeptide	Atp7b	NM_007511	1.9
sarcospan	Sspn	NM_010656	1.9
protocadherin 9	Pcdh9	NM_001081377	1.9
myotubularin related protein 6	Mtmr6	NM_144843	1.9
RIKEN cDNA 2310039D24 gene	2310039D24Rik	ENSMUST00000098499	1.9
Unknown	H2-Q1	NM_010390	1.9
glutaredoxin 2 (thioltransferase)	Glrx2	NM_001038592	1.9
proteasome (prosome, macropain) subunit, alpha type 3	Psma3	NM_011184	1.9
homeo box C5	Hoxc5	NM_175730	1.9
proline-rich protein MP5	Prpmp5	NM_001024705	1.9

Additional Table 2b. Genderwise differentially expressed genes (p ≤0.01) with a regulation of ≥ 1.5

DAVID GeneName	Gene Symbol	RefSeq	Fold Change Female/Males
NADH dehydrogenase (ubiquinone) flavoprotein 3	Ndufv3	NM_030087	1.9
cDNA sequence BC016201	BC016201	BC016201	1.9
melanocyte proliferating gene 1	Myg1	NM_021713	1.9
LysM, putative peptidoglycan-binding, domain containing 4	Lysmd4	NM_175215	1.9
RIKEN cDNA 9930038B18 gene	9930038B18Rik	ENSMUST00000062469	1.9
DEAH (Asp-Glu-Ala-His) box polypeptide 36	Dhx36	NM_028136	1.9
stannin	Snn	NM_009223	1.9
RIKEN cDNA 2310005E10 gene	Akr1b10	NM_172398	1.9
solute carrier family 17 (sodium-dependent inorganic phosphate cotransporter), member 6	Slc17a6	NM_080853	1.9
similar to Unknown (protein for IMAGE:4910858)	ND6	ENSMUST00000082419	1.9
chemokine (C-X-C motif) receptor 7	Cxcr7	NM_007722	1.9
cathepsin G	Ctsg	NM_007800	1.9
keratinocyte associated protein 2	Krtcap2	NM_025327	1.9
rabaptin, RAB GTPase binding effector protein 2	Rabep2	NM_030566	1.9
RIKEN cDNA 2610018G03 gene	2610018G03Rik	NM_133729	1.9
slowmo homolog 1 (Drosophila)	Slmo1	NM_144867	1.8
transmembrane 4 superfamily member 5	Tm4sf5	NM_029360	1.8
pol polyprotein	LOC280487	X16670	1.8
predicted gene 4638	Gm4638	XM_001480931	1.8
Unknown	Dnaja1	NM_008298	1.8
protease, serine, 16 (thymus)	Prss16	NM_019429	1.8
receptor accessory protein 4	Reep4	NM_180588	1.8
pyrophosphatase (inorganic) 1	Ppa1	NM_026438	1.8
RIKEN cDNA 4930538K18 gene	4930538K18Rik	BC048569	1.8
zinc finger protein of the cerebellum 1	Zic1	NM_009573	1.8
SH3/ankyrin domain gene 2	Shank2	ENSMUST00000105902	1.8
polymerase (RNA) II (DNA directed) polypeptide F	Polr2f	NM_027231	1.8
transmembrane protein 72	Tmem72	NM_178768	1.8
transcription elongation factor A (SII)-like 8	Tceal8	NM_025703	1.8
RIKEN cDNA 1810074P20 gene	1810074P20Rik	NM_026194	1.8
aldehyde dehydrogenase family 3, subfamily A2	Aldh3a2	NM_007437	1.8
progestin and adipoQ receptor family member VII	Paqr7	NM_027995	1.8
RIKEN cDNA 6530418L21 gene	6530418L21Rik	BC052371	1.8
adenylosuccinate synthetase, non muscle	Adss	NM_007422	1.8
PRP31 pre-mRNA processing factor 31 homolog (yeast)	Prpf31	NM_027328	1.8
RAB27A, member RAS oncogene family	Rab27a	NM_023635	1.8
Unknown	Gm15542	XM_001480612	1.8
interleukin 18 receptor accessory protein	Il18rap	NM_010553	1.8
HtrA serine peptidase 2	Htra2	NM_019752	1.8
activin A receptor, type IC	Acvr1c	NM_001111030	1.8
leucine rich repeat and fibronectin type III domain containing 3	Lrfn3	NM_175478	1.8
protease, serine, 22	Prss22	NM_133731	1.8
fibroblast growth factor 14	Fgf14	NM_010201	1.8
neuroglobin	Ngb	NM_022414	1.8
bruno-like 5, RNA binding protein (Drosophila)	Brunol5	NM_176954	1.8
acidic (leucine-rich) nuclear phosphoprotein 32 family, member E	Anp32e	NM_023210	1.8
phosducin	Pdc	NM_001159730	1.8
gamma-aminobutyric acid (GABA) A receptor, subunit alpha 4	Gabra4	NM_010251	1.8
phosphoribosyl pyrophosphate synthetase 1	Gm5081	ENSMUST00000021358	1.8

Additional Table 2b. Genderwise differentially expressed genes (p ≤0.01) with a regulation of ≥ 1.5

DAVID GeneName	Gene Symbol	RefSeq	Fold Change Female/Males
mediator of RNA polymerase II transcription, subunit 31 homolog (yeast)	Med31	NM_026068	1.8
wingless related MMTV integration site 2b	Wnt2b	NM_009520	1.8
nuclear transcription factor-Y beta	Nfyb	NM_010914	1.8
akirin 1	Akirin1	NM_023423	1.8
methyltransferase like 14	Mettl14	NM_201638	1.8
hypothetical protein LOC100233175	LOC100233175	NM_001142938	1.8
gasdermin C2	Gsdmc2	BC113153	1.8
aldehyde oxidase 4	Aox4	NM_023631	1.8
leucine rich repeat containing 26	Lrrc26	NM_146117	1.8
fibroblast growth factor 18	Fgf18	NM_008005	1.8
ubiquitin-like domain containing CTD phosphatase 1	Ublcp1	ENSMUST00000102795	1.8
predicted gene 8357	Gm8357	XR_033155	1.8
SH2 domain containing 4A	Sh2d4a	NM_028182	1.8
RIKEN cDNA 1700086O06 gene	1700086O06Rik	NR_027904	1.8
selenoprotein K pseudogene	LOC100134990	NR_004857	1.8
HECT, C2 and WW domain containing E3 ubiquitin protein ligase 1	Hecw1	NM_001081348	1.8
RIKEN cDNA 4932425I24 gene	4932425I24Rik	NM_001081025	1.8
mitochondrial ribosomal protein L24	Mrpl24	NM_026591	1.8
4lysine (K)-specific demethylase 6A	Kdm6a	NM_009483	1.8
predicted gene 498	Gm498	ENSMUST00000033411	1.8
eukaryotic translation initiation factor 3, subunit J	Eif3j	NM_144545	1.8
olfactory receptor 646	Olfr646	NM_147056	1.8
transmembrane protease, serine 3	Tmprss3	NM_080727	1.8
Unknown	Gm6155	XM_884867	1.8
Shc SH2-domain binding protein 1	Shcbp1	NM_011369	1.8
zinc finger protein 640	Zfp640	ENSMUST00000071320	1.8
angiopoietin-like 3	Angptl3	NM_013913	1.8
RIKEN cDNA 1700001C19 gene	1700001C19Rik	BC028263	1.8
RIKEN cDNA 2310007B03 gene	2310007B03Rik	NM_172411	1.8
translocase of inner mitochondrial membrane 9 homolog (yeast)	Timm9	NM_013896	1.8
hairless	Hr	NM_021877	1.8
forkhead box C1	Foxc1	NM_008592	1.8
WW domain-containing oxidoreductase	Wwox	NM_019573	1.8
YKT6 homolog (S. Cerevisiae)	Ykt6	NM_019661	1.8
jagunal homolog 1 (Drosophila)	Jagn1	NM_026365	1.8
isocitrate dehydrogenase 1 (NADP+), soluble	Idh1	NM_001111320	1.8
transmembrane protein 164	Tmem164	ENSMUST00000054263	1.8
major facilitator superfamily domain containing 9	Mfsd9	NM_172499	1.8
predicted gene 9108	Gm9108	XR_034404	1.8
tetratricopeptide repeat domain 39A	Ttc39a	NM_001145948	1.8
zinc finger protein	Zfp229	ENSMUST00000065871	1.8
zinc finger protein 819	Zfp819	NM_028913	1.8
expressed sequence AU018778	AU018778	BC013479	1.8
family with sequence similarity 82, member B	Fam82b	NM_025476	1.8
microspherule protein 1	Mcrs1	NM_016766	1.8
ATPase, Ca++ transporting, plasma membrane 2	Atp2b2	NM_009723	1.8
nudix (nucleoside diphosphate linked moiety X)-type motif 12	Nudt12	NM_026497	1.8
G protein-coupled receptor 85	Gpr85	NM_145066	1.8
mitochondrial carrier homolog 2 (C. elegans)	Mtch2	NM_019758	1.8
coatomer protein complex, subunit zeta 1	Copz1	NM_019817	1.8

Additional Table 2b. Genderwise differentially expressed genes (p ≤0.01) with a regulation of ≥ 1.5

DAVID GeneName	Gene Symbol	RefSeq	Fold Change Female/Males
solute carrier family 30 (zinc transporter), member 3	Slc30a3	NM_011773	1.8
cytochrome c oxidase, subunit VIIa 2	Cox7a2	NM_009945	1.8
CD302 antigen	Cd302	NM_025422	1.8
acid phosphatase 1, soluble	Acp1	NM_001110239	1.8
glyceraldehyde-3-phosphate dehydrogenase, spermatogenic	Gapdhs	NM_008085	1.8
tissue factor pathway inhibitor	Tfpi	NM_011576	1.8
guanine nucleotide binding protein (G protein), gamma 5	Gng5	NM_010318	1.8
baculoviral IAP repeat-containing 5	Birc5	NM_009689	1.8
THO complex 7 homolog (Drosophila)	Thoc7	NM_025435	1.8
LYR motif containing 1	Lyrm1	NM_029610	1.8
RIKEN cDNA 1110003E01 gene	1110003E01Rik	BC009097	1.8
copine VI	Cpne6	NM_001136057	1.8
zinc binding alcohol dehydrogenase, domain containing 2	Zadh2	NM_146090	1.8
RIKEN cDNA 1700104A03 gene	1700104A03Rik	ENSMUST00000098759	1.8
family with sequence similarity 57, member A	Fam57a	NM_027773	1.8
predicted gene 13242	Gm13242	NM_001103158	1.8
gasdermin A3	Gsdma3	NM_001007461	1.8
NLR family, apoptosis inhibitory protein 1	Naip1	NM_008670	1.8
RIKEN cDNA 4933436C20 gene	4933436C20Rik	ENSMUST00000034183	1.8
expressed sequence AI132487	AI132487	NM_001012310	1.8
serine peptidase inhibitor, Kazal type 2	Spink2	NM_183284	1.8
ZW10 interactor	Zwint	ENSMUST00000020081	1.8
predicted gene 13937	Gm13937	XR_034534	1.8
RIKEN cDNA 1700012B15 gene	1700012B15Rik	BC060685	1.8
basonuclin 1	Bnc1	NM_007562	1.8
deafness, autosomal recessive 59 (human)	Dfnb59	NM_001080711	1.8
protease, serine, 36	Prss36	NM_001081374	1.8
expressed sequence AI462493	AI462493	NM_001160356	1.8
sterile alpha motif domain containing 3	Samd3	NM_001115154	1.8
V-set and transmembrane domain containing 2B	Vstm2b	NM_021387	1.8
KTI12 homolog, chromatin associated (S. cerevisiae)	Kti12	NM_029571	1.8
small nuclear ribonucleoprotein polypeptide F	Snrpf	BC100499	1.8
limb region 1	Lmbr1	NM_020295	1.8
calcium channel, voltage-dependent, L type, alpha 1D subunit	Cacna1d	NM_028981	1.8
calmodulin binding transcription activator 1	Camta1	NM_001081557	1.8
cannabinoid receptor interacting protein 1	Cnrip1	NM_029861	1.8
GRB10 interacting GYF protein 1	Gigyf1	NM_031408	1.8
kinesin family member 21B	Kif21b	NM_001039472	1.8
cDNA sequence BC048355	BC048355	NM_207161	1.8
predicted gene 501	Gm501	XM_146277	1.8
proteasome (prosome, macropain) 26S subunit, non-ATPase, 7	Psmd7	NM_010817	1.8
neuropilin (NRP) and tolloid (TLL)-like 1	Neto1	NM_144946	1.8
endothelin converting enzyme 2	Ece2	NM_025462	1.8
progestin and adipoQ receptor family member VIII	Paqr8	AK006107	1.8
RAD21 homolog (S. pombe)	Rad21	NM_009009	1.8
TNF receptor-associated factor 1	Traf1	NM_009421	1.8
zinc finger protein 820	Zfp820	NM_029281	1.8
heat shock protein 1 (chaperonin)	Hspd1	NM_010477	1.8
expressed sequence AW146154	AW146154	NM_001033530	1.8
RIKEN cDNA 9030607L17 gene	9030607L17Rik	BC057953	1.8

Additional Table 2b. Genderwise differentially expressed genes (p ≤0.01) with a regulation of ≥ 1.5

DAVID GeneName	Gene Symbol	RefSeq	Fold Change Female/Males
harbinger transposase derived 1	Harbi1	NM_178724	1.8
AU RNA binding protein/enoyl-coenzyme A hydratase	Auh	NM_016709	1.8
predicted gene 4968	Gm4968	XM_145024	1.8
transmembrane protein 68	Tmem68	NM_028097	1.8
RIKEN cDNA 2810026P18 gene	2810026P18Rik	BC032970	1.8
similar to crooked legs CG14938-PB	4930522L14Rik	BC065392	1.8
predicted gene 14134	Gm14134	NM_001085521	1.8
magnesium transporter 1	Magt1	NM_025952	1.8
methyltransferase like 4, pseudogene 1	Mettl4-ps1	ENSMUST00000024852	1.8
RIKEN cDNA 4933434M16 gene	4933434M16Rik	ENSMUST00000020950	1.8
ubiquitin-conjugating enzyme E2A, RAD6 homolog (S. cerevisiae)	Ube2a	NM_019668	1.8
Wilms tumor 1 homolog	Wt1	NM_144783	1.8
peroxisomal biogenesis factor 5	Pex5	NM_008995	1.8
solute carrier family 22 (organic anion/cation transporter), member 12	Slc22a12	NM_009203	1.8
ring finger protein 34	Rnf34	NM_030564	1.8
predicted gene 9264	Gm9264	XR_034445	1.8
collagen, type XII, alpha 1	Col12a1	NM_007730	1.8
solute carrier family 9 (sodium/hydrogen exchanger), member 5	Slc9a5	NM_001081332	1.8
proteasome (prosome, macropain) subunit, alpha type, 8	Psma8	ENSMUST00000040860	1.8
RIKEN cDNA 2610001J05 gene	2610001J05Rik	NR_024619	1.8
gap junction protein, gamma 2	Gjc2	NM_080454	1.8
chromatin assembly factor 1, subunit A (p150)	Chaf1a	NM_013733	1.8
syndecan binding protein	Sdcbp	NM_001098227	1.8
transmembrane and coiled-coil domains 3	Tmco3	NM_172282	1.8
mago-nashi homolog, proliferation-associated (Drosophila)	Magoh	NM_010760	1.8
sema domain, immunoglobulin domain (Ig), transmembrane domain (TM) and short cytoplasmic domain, (semaphorin) 4B	Sema4b	NM_013659	1.8
sedoheptulokinase	Shpk	NM_029031	1.8
DNA segment, human D4S114	D0H4S114	NM_053078	1.8
fibroblast growth factor 22	Fgf22	NM_023304	1.8
solute carrier family 2 (facilitated glucose transporter), member 2	Slc2a2	NM_031197	1.8
RIKEN cDNA 2400001E08 gene	2400001E08Rik	NM_025605	1.8
nuclear factor of kappa light polypeptide gene enhancer in B-cells inhibitor-like 2	Nfkbil2	NM_183091	1.8
F-box and leucine-rich repeat protein 15	Fbxl15	NM_133694	1.8
DNA segment, Chr 4, Wayne State University 53, expressed	D4Wsu53e	BC043057	1.8
inosine 5'-phosphate dehydrogenase 2	Impdh2	NM_011830	1.8
FK506 binding protein 14	Fkbp14	NM_153573	1.8
transmembrane protein 184b	Tmem184b	NM_172608	1.8
nuclear factor of activated T-cells, cytoplasmic, calcineurin-dependent 4	Nfatc4	NM_023699	1.8
RIKEN cDNA 2310015B20 gene	2310015B20Rik	ENSMUST00000020090	1.8
uromodulin	Umod	NM_009470	1.8
RIKEN cDNA 1810037I17 gene	1810037I17Rik	NM_024461	1.8
proline dehydrogenase	Prodh	NM_011172	1.8
sterile alpha and HEAT/Armadillo motif containing 1	Sarm1	ENSMUST00000108287	1.8
defensin related sequence cryptdin peptide (paneth cells)	AY761184	AY761184	1.8
Sfi1 homolog, spindle assembly associated (yeast)	Sfi1	NM_030207	1.8
RIKEN cDNA 4631416L12 gene	4631416L12Rik	NM_001081295	1.7
defensin, alpha, 21	Defa21	NM_183253	1.7
eukaryotic translation initiation factor 1A, Y-linked	Eif1ay	NM_025437	1.7

Additional Table 2b. Genderwise differentially expressed genes (p ≤0.01) with a regulation of ≥ 1.5

DAVID GeneName	Gene Symbol	RefSeq	Fold Change Female/Males
high mobility group nucleosomal binding domain 1	Hmgn1	NM_008251	1.7
RAD51 homolog c (S. cerevisiae)	Rad51c	NM_053269	1.7
neutrophil cytosolic factor 1	Ncf1	NM_010876	1.7
cytochrome b-5	Cyb5	NM_025797	1.7
G protein-coupled receptor 133	Gpr133	NM_001081342	1.7
cAMP responsive element modulator	Crem	NM_001110854	1.7
geranylgeranyl diphosphate synthase 1	Ggps1	NM_010282	1.7
hydroxyacyl-Coenzyme A dehydrogenase/3-ketoacyl-Coenzyme A thiolase/enoyl-Coenzyme A hydratase (trifunctional protein), beta subunit	Hadhb	NM_145558	1.7
RIKEN cDNA 1500011H22 gene	1500011H22Rik	BC019498	1.7
zinc finger, FYVE domain containing 1	Zfyve1	NM_183154	1.7
cDNA sequence BC037703	BC037703	BC037703	1.7
non-SMC element 2 homolog (MMS21, S. cerevisiae)	Nsmce2	NM_026746	1.7
DEAD (Asp-Glu-Ala-Asp) box polypeptide 47	Ddx47	NM_026360	1.7
transient receptor potential cation channel, subfamily M, member 1	Trpm1	NM_018752	1.7
expressed sequence AI314976	AI314976	BC022574	1.7
proteasome (prosome, macropain) 26S subunit, non-ATPase, 6	Psmd6	NM_025550	1.7
arginine vasopressin receptor 1A	Avpr1a	NM_016847	1.7
asparagine synthetase domain containing 1	Asnsd1	NM_133728	1.7
LYR motif containing 2	Lyrm2	NM_175364	1.7
short coiled-coil protein	Scoc	NM_001039137	1.7
regulatory factor X, 5 (influences HLA class II expression)	Rfx5	NM_017395	1.7
RIKEN cDNA 4833422F24 gene	4833422F24Rik	BC020160	1.7
glutamate receptor, ionotropic, NMDA2C (epsilon 3)	Grin2c	NM_010350	1.7
RIKEN cDNA 5430419D17 gene	5430419D17Rik	NM_175166	1.7
enoyl Coenzyme A hydratase domain containing 2	Echdc2	NM_026728	1.7
general transcription factor II H, polypeptide 4	Gtf2h4	NM_010364	1.7
myosin XV	Myo15	NM_010862	1.7
zinc finger and BTB domain containing 48	Zbtb48	NM_133879	1.7
5-hydroxytryptamine (serotonin) receptor 1D	Htr1d	NM_008309	1.7
Unknown	Gm8779	XR_032228	1.7
armadillo repeat containing 6	Armc6	NM_133972	1.7
serine/threonine kinase 32A	Stk32a	BC055002	1.7
PTC7 protein phosphatase homolog (S. cerevisiae)	Pptc7	NM_177242	1.7
solute carrier family 7 (cationic amino acid transporter, y+ system), member 2	Slc7a2	NM_007514	1.7
predicted gene 4894	Gm4894	NM_177701	1.7
seryl-aminoacyl-tRNA synthetase	Sars	NM_011319	1.7
spermatogenesis associated 20	Spata20	NM_144827	1.7
myelin-associated oligodendrocytic basic protein	Mobp	NM_008614	1.7
RIKEN cDNA 2900010J23 gene	2900010J23Rik	BC021748	1.7
G protein-coupled receptor 22	Gpr22	NM_175191	1.7
D-aspartate oxidase	Ddo	NM_027442	1.7
zona pellucida glycoprotein 2	Zp2	NM_011775	1.7
Unknown	Rpl10	NM_052835	1.7
sorting nexin 11	Snx11	NM_028965	1.7
protein phosphatase 1, regulatory (inhibitor) subunit 8	Ppp1r8	NM_146154	1.7
sulfatase modifying factor 1	Sumf1	NM_145937	1.7
syntaxin 5A	Stx5a	NM_019829	1.7
phosphoglycerate kinase 2	Pgk2	NM_031190	1.7

Additional Table 2b. Genderwise differentially expressed genes (p ≤0.01) with a regulation of ≥ 1.5

DAVID GeneName	Gene Symbol	RefSeq	Fold Change Female/Males
splicing factor, arginine/serine-rich 3 (SRp20)	Sfrs3	NM_013663	1.7
matrix metallopeptidase 19	Mmp19	NM_021412	1.7
small Cajal body-specific RNA 17	Scarna17	AF357342	1.7
RIB43A domain with coiled-coils 2	Ribc2	NM_026357	1.7
tektin 1	Tekt1	NM_011569	1.7
peroxisomal biogenesis factor 5-like	Pex5l	NM_021483	1.7
coagulation factor XI	F11	NM_028066	1.7
ubiquitin-fold modifier conjugating enzyme 1	Ufc1	NM_025388	1.7
cDNA sequence BC011426	BC011426	NM_145490	1.7
trafficking protein particle complex 2	Trappc2	NM_025432	1.7
family with sequence similarity 3, member C	Fam3c	NM_138587	1.7
RIKEN cDNA 1810030O07 gene	1810030O07Rik	BC137660	1.7
predicted gene 6653	Gm6653	AK132809	1.7
zinc finger SCAN domains 29	Zscan29	NM_178889	1.7
RIKEN cDNA 2210406O10 gene	2210406O10Rik	ENSMUST00000044964	1.7
predicted gene 10605	Gm10605	ENSMUST00000098265	1.7
phospholipase A2, group VII (platelet-activating factor acetylhydrolase, plasma)	Pla2g7	NM_013737	1.7
phosphoribosyl pyrophosphate synthetase-associated protein 1	Prpsap1	BC029621	1.7
ubiquitin-associated protein 1	Ubap1	NM_023305	1.7
predicted gene 4976	Gm4976	NM_001013764	1.7
Ellis van Creveld syndrome 2 homolog (human)	Evc2	NM_145920	1.7
olfactory receptor 750	Olfr750	NM_207558	1.7
ATPase, H+ transporting, lysosomal V1 subunit G1	Atp6v1g1	NM_024173	1.7
heparan sulfate (glucosamine) 3-O-sulfotransferase 3B1	Hs3st3b1	NM_018805	1.7
TM2 domain containing 2	Tm2d2	NM_027194	1.7
RIKEN cDNA 5730469M10 gene	5730469M10Rik	BC056635	1.7
zinc finger protein 780B	Zfp780b	NM_001081021	1.7
telomerase reverse transcriptase	Tert	NM_009354	1.7
phosphomannomutase 1	Pmm1	NM_013872	1.7
predicted gene 10158	Gm10158	ENSMUST00000084967	1.7
cytochrome b5 domain containing 1	Cyb5d1	NM_001045525	1.7
GTP binding protein 3	Gtpbp3	NM_032544	1.7
cytotoxic and regulatory T cell molecule	Crtam	NM_019465	1.7
family with sequence similarity 46, member B	Fam46b	NM_175307	1.7
RIKEN cDNA 2610002J02 gene	2610002J02Rik	BC117034	1.7
annexin A3	Anxa3	NM_013470	1.7
interferon induced transmembrane protein 7	Ifitm7	NM_028968	1.7
nebulin	Neb	NM_010889	1.7
ADP-ribosylation factor-like 13B	Arl13b	NM_026577	1.7
delta-like 2 homolog (Drosophila)	Dlk2	NM_207666	1.7
eukaryotic translation initiation factor 2, subunit 3, structural gene X-linked	Eif2s3x	NM_012010	1.7
G1 to S phase transition 2	Gspt2	NM_008179	1.7
MOB1, Mps One Binder kinase activator-like 3 (yeast)	Mobkl3	NM_025283	1.7
defender against cell death 1	Dad1	NM_001113358	1.7
gem (nuclear organelle) associated protein 6	Gemin6	NM_026053	1.7
proliferating cell nuclear antigen	Pcna	NM_011045	1.7
predicted gene 13152	Gm13152	NM_001039209	1.7
unc-93 homolog A (C. elegans)	Unc93a	NM_199252	1.7
cadherin 10	Cdh10	NM_009865	1.7

Additional Table 2b. Genderwise differentially expressed genes (p ≤0.01) with a regulation of ≥ 1.5

DAVID GeneName	Gene Symbol	RefSeq	Fold Change Female/Males
PHD and ring finger domains 1	Phrf1	NM_001081118	1.7
alpha-methylacyl-CoA racemase	Amacr	NM_008537	1.7
tumor necrosis factor receptor superfamily, member 9	Tnfrsf9	NM_011612	1.7
arachidonate 12-lipoxygenase, 12R type	Alox12b	NM_009659	1.7
bombesin-like receptor 3	Brs3	NM_009766	1.7
McKusick-Kaufman syndrome protein	Mkks	NM_021527	1.7
predicted gene 14391	Gm14391	NM_001099308	1.7
Unknown	Gm14434	NM_001101804	1.7
GTP-binding protein 8 (putative)	Gtpbp8	NM_025332	1.7
RIKEN cDNA 2900064A13 gene	2900064A13Rik	BC115477	1.7
RIKEN cDNA 4933402N03 gene	4933402N03Rik	BC115646	1.7
StAR-related lipid transfer (START) domain containing 5	Stard5	NM_023377	1.7
H3 histone, family 3B	H3f3b	NM_008211	1.7
contactin 5	Cntn5	NM_001033359	1.7
luteinizing hormone/choriogonadotropin receptor	Lhcgr	NM_013582	1.7
protein tyrosine phosphatase-like (proline instead of catalytic arginine), member b	Ptplb	NM_023587	1.7
zinc finger protein 346	Zfp346	NM_012017	1.7
zinc finger and BTB domain containing 33	Zbtb33	NM_020256	1.7
biliverdin reductase A	Blvra	NM_026678	1.7
tetratricopeptide repeat domain 9C	Ttc9c	NM_027412	1.7
PFTAIRE protein kinase 2	Pftk2	NM_001033373	1.7
heat shock protein 1 (chaperonin 10), related sequence 1	Hspe1	NM_008303	1.7
RIKEN cDNA 2010300C02 gene	2010300C02Rik	BC072639	1.7
inhibitor of DNA binding 2	Id2	NM_010496	1.7
heme binding protein 1	Hebp1	NM_013546	1.7
zinc finger protein 13	Zfp13	NM_011747	1.7
guanylate cyclase 2c	Gucy2c	NM_001127318	1.7
nei endonuclease VIII-like 1 (E. coli)	Neil1	NM_028347	1.7
cytochrome P450, family 2, subfamily d, polypeptide 26	Cyp2d26	NM_029562	1.7
ATPase, class V, type 10A	Atp10a	NM_009728	1.7
hydroxy-delta-5-steroid dehydrogenase, 3 beta- and steroid delta-isomerase 7	Hsd3b7	NM_133943	1.7
RIKEN cDNA 1700034I23 gene	1700034I23Rik	BC117739	1.7
troponin I, skeletal, fast 2	Tnni2	NM_009405	1.7
EP300 interacting inhibitor of differentiation 1	Eid1	NM_025613	1.7
NLR family, CARD domain containing 5	Nlrc5	FJ889356	1.7
zinc finger protein 826	Zfp826	BC086681	1.7
glutamyl aminopeptidase	Enpep	NM_007934	1.7
RNA binding motif protein 17	Rbm17	NM_152824	1.7
zinc finger protein 426	Zfp426	NM_146221	1.7
interleukin 11 receptor, alpha chain 1	Il11ra1	NM_010549	1.7
RIKEN cDNA 1700037C18 gene	1700037C18Rik	ENSMUST00000006139	1.7
FUN14 domain containing 2	Fundc2	NM_026126	1.7
RIKEN cDNA 1700116B05 gene	1700116B05Rik	ENSMUST00000023314	1.7
predicted gene 12298	Gm12298	ENSMUST00000101039	1.7
predicted gene 11472	Gm11472	XM_001005073	1.7
HECT domain and ankyrin repeat containing, E3 ubiquitin protein ligase 1	Hace1	NM_172473	1.7
coiled-coil-helix-coiled-coil-helix domain containing 2	Chchd2	NM_024166	1.7
ring finger protein 114	Rnf114	NM_030743	1.7
intraflagellar transport 57 homolog (Chlamydomonas)	Ift57	NM_028680	1.7

Additional Table 2b. Genderwise differentially expressed genes (p ≤0.01) with a regulation of ≥ 1.5

DAVID GeneName	Gene Symbol	RefSeq	Fold Change Female/Males
asparagine-linked glycosylation 6 homolog (yeast, alpha-1,3,-glucosyltransferase)	Alg6	NM_001081264	1.7
predicted gene 11349	Gm11349	XR_033316	1.7
tRNA nucleotidyl transferase, CCA-adding, 1	Trnt1	NM_027296	1.7
predicted gene 9132	Gm9132	XR_002170	1.7
G protein-coupled receptor 150	Gpr150	NM_175495	1.7
SAC1 (suppressor of actin mutations 1, homolog)-like (S. cerevisiae)	Sacm1l	NM_030692	1.7
acyl-Coenzyme A binding domain containing 5	Acbd5	NM_001102437	1.7
N-acetylated alpha-linked acidic dipeptidase 2	Naalad2	NM_028279	1.7
Bardet-Biedl syndrome 9 (human)	Bbs9	NM_178415	1.7
pleckstrin and Sec7 domain containing	Psd	NM_028627	1.7
CKLF-like MARVEL transmembrane domain containing 6	Cmtm6	NM_026036	1.7
proteasome (prosome, macropain) 26S subunit, non-ATPase, 14	Psmd14	NM_021526	1.7
DEAD (Asp-Glu-Ala-Asp) box polypeptide 19b	Ddx19b	NM_172284	1.7
transcription elongation factor A (SII)-like 1	Tceal1	NM_146236	1.7
solute carrier family 30 (zinc transporter), member 5	Slc30a5	NM_022885	1.7
scaffold attachment factor B2	Safb2	NM_001029979	1.7
expressed sequence AU019823	AU019823	NM_001134902	1.7
transmembrane protein 38B	Tmem38b	NM_028053	1.7
myosin, heavy chain 15	Myh15	XM_001002743	1.7
mortality factor 4 like 2	Gm5521	XR_034247	1.7
ladinin	Lad1	NM_133664	1.7
predicted gene 4961	Gm4961	XR_001540	1.7
DIRAS family, GTP-binding RAS-like 1	Diras1	NM_145217	1.7
MORN repeat containing 4	Morn4	NM_198108	1.7
budding uninhibited by benzimidazoles 1 homolog, beta (S. cerevisiae)	Bub1b	NM_009773	1.7
BAI1-associated protein 2-like 1	Baiap2l1	NM_025833	1.7
transient receptor potential cation channel, subfamily V, member 5	Trpv5	NM_001007572	1.7
malignant T cell amplified sequence 1	Mcts1	NM_026902	1.7
integrin beta 8	Itgb8	NM_177290	1.7
predicted gene 2736	Gm2736	XR_031811	1.7
RIKEN cDNA E130309F12 gene	E130309F12Rik	NM_178756	1.7
Ras and Rab interactor-like	Rinl	NM_177158	1.7
general transcription factor II H, polypeptide 2	Gtf2h2	NM_022011	1.7
predicted gene 11536	Gm11536	ENSMUST00000100525	1.7
RIKEN cDNA 2310016M24 gene	2310016M24Rik	NM_183256	1.7
ankyrin repeat and SOCS box-containing 11	Asb11	NM_026853	1.7
serine/threonine kinase 36 (fused homolog, Drosophila)	Stk36	NM_175031	1.7
zinc finger protein 207	Zfp207	NM_001130169	1.7
TIP41, TOR signalling pathway regulator-like (S. cerevisiae)	Tiprl	NM_145513	1.7
spermine synthase	Gm7270	XR_030809	1.7
Ngfi-A binding protein 1	Nab1	NM_008667	1.7
PRP6 pre-mRNA splicing factor 6 homolog (yeast)	Prpf6	NM_133701	1.7
RIKEN cDNA 4930449I04 gene	4930449I04Rik	AK015432	1.7
family with sequence similarity 105, member B	Fam105b	NM_001013792	1.7
basic helix-loop-helix domain containing, class B9	Bhlhb9	NM_198161	1.7
ATPase inhibitory factor 1	Atpif1	NM_007512	1.7
RIKEN cDNA 4833442J19 gene	4833442J19Rik	BC046911	1.7
carboxylesterase 8 (putative)	Ces8	NM_146213	1.7
zinc finger protein 326	Zfp326	NM_018759	1.7

Additional Table 2b. Genderwise differentially expressed genes (p ≤0.01) with a regulation of ≥ 1.5

DAVID GeneName	Gene Symbol	RefSeq	Fold Change Female/Males
RIKEN cDNA 2210021J22 gene	2210021J22Rik	BC025858	1.7
keratin associated protein 5-3	Krtap5-3	ENSMUST00000084414	1.7
RIKEN cDNA 2410004B18 gene	2410004B18Rik	NM_025555	1.7
family with sequence similarity 108, member B	Fam108b	NM_146096	1.7
TATA box binding protein (Tbp)-associated factor, RNA polymerase I, C	Taf1c	NM_021441	1.7
LIM homeobox protein 6	Lhx6	NM_008500	1.7
RIKEN cDNA 1810022C23 gene	1810022C23Rik	BC014724	1.7
eukaryotic translation initiation factor 3, subunit M	Eif3m	NM_145380	1.7
Ras-related GTP binding C	Rragc	NM_017475	1.7
kielin/chordin-like protein	Kcp	NM_001029985	1.7
zinc finger protein 90	Zfp90	NM_011764	1.7
RIKEN cDNA 9330129D05 gene	9330129D05Rik	ENSMUST00000041252	1.7
minichromosome maintenance deficient 10 (S. cerevisiae)	Mcm10	NM_027290	1.7
autophagy-related 10 (yeast)	Atg10	NM_025770	1.7
nucleolar protein 7	Nol7	NM_023554	1.7
nuclear fragile X mental retardation protein interacting protein 1	Nufip1	NM_013745	1.6
Unknown	LOC626711	XR_004836	1.6
LYR motif containing 5	Lyrm5	NM_133688	1.6
Unknown	LOC100047042	XM_001477268	1.6
glutaryl-Coenzyme A dehydrogenase	Gcdh	NM_008097	1.6
mutS homolog 2 (E. coli)	Msh2	NM_008628	1.6
similar to Ornithine decarboxylase (ODC)	LOC677259	XR_033749	1.6
protease, serine, 2	Prss2	NM_009430	1.6
sphingomyelin phosphodiesterase 3, neutral	Smpd3	NM_021491	1.6
Unknown	Bag5	NM_027404	1.6
RIKEN cDNA 4931408A02 gene	4931408A02Rik	ENSMUST00000099548	1.6
mediator complex subunit 21	Med21	NM_025315	1.6
TSC22 domain family, member 1	Tsc22d1	NM_207652	1.6
RIKEN cDNA 1600014C10 gene	1600014C10Rik	NM_001085385	1.6
predicted gene 13688	Gm13688	XR_033267	1.6
predicted gene 5502	Gm5502	XR_032770	1.6
golgi autoantigen, golgin subfamily a, 7	Golga7	NM_020585	1.6
coiled-coil domain containing 102A	Ccdc102a	NM_001033533	1.6
stimulated by retinoic acid gene 8	Stra8	NM_009292	1.6
ribosomal protein L5	Rpl5	NM_016980	1.6
SRY-box containing gene 10	Sox10	NM_011437	1.6
general transcription factor IIB	Gtf2b	NM_145546	1.6
monoamine oxidase B	Maob	NM_172778	1.6
RIKEN cDNA 2410002O22 gene	2410002O22Rik	NM_025879	1.6
vav 3 oncogene	Vav3	NM_020505	1.6
BCL2-like 11 (apoptosis facilitator)	Bcl2l11	NM_207680	1.6
solute carrier family 35 (UDP-N-acetylglucosamine (UDP-GlcNAc) transporter), member 3	Slc35a3	NM_144902	1.6
ring finger protein 220	Rnf220	NM_025739	1.6
RIKEN cDNA 2010107E04 gene	2010107E04Rik	NM_027360	1.6
family with sequence similarity 119, member A	Fam119a	BC026952	1.6
parathyroid hormone 1 receptor	Pth1r	NM_011199	1.6
RIKEN cDNA C030006K11 gene	C030006K11Rik	NM_145472	1.6
activating transcription factor 1	Gm1862	XM_001478226	1.6
hydrolethalus syndrome 1	Hyls1	NM_029762	1.6

Additional Table 2b. Genderwise differentially expressed genes (p ≤0.01) with a regulation of ≥ 1.5

DAVID GeneName	Gene Symbol	RefSeq	Fold Change Female/Males
mediator complex subunit 20	Med20	NM_020048	1.6
WD repeat domain containing 82	Wdr82	NM_029896	1.6
RIKEN cDNA 4921513I03 gene	4921513I03Rik	ENSMUST00000056994	1.6
poly(rC) binding protein 3	Pcbp3	NM_021568	1.6
X-ray repair complementing defective repair in Chinese hamster cells 2	Xrcc2	NM_020570	1.6
mercaptopyruvate sulfurtransferase	Mpst	NM_138670	1.6
transferrin receptor	Tfrc	NM_011638	1.6
transcription factor 15	Tcf15	ENSMUST00000089112	1.6
exportin 1, CRM1 homolog (yeast)	Xpo1	NM_134014	1.6
serine (or cysteine) peptidase inhibitor, clade C (antithrombin), member 1	Serpinc1	NM_080844	1.6
RIKEN cDNA 5730522E02 gene	5730522E02Rik	NR_027973	1.6
zinc finger protein 97	Zfp97	NM_011765	1.6
RIKEN cDNA 4930583K01 gene	4930583K01Rik	NR_027879	1.6
small nuclear ribonucleoprotein 27 (U4/U6.U5)	Snrnp27	NM_025665	1.6
Yip1 domain family, member 7	Yipf7	NM_023784	1.6
predicted gene 3771	Gm3771	XR_033804	1.6
transmembrane protein 213	Tmem213	BC104374	1.6
regulating synaptic membrane exocytosis 4	Rims4	NM_183023	1.6
Rho GTPase activating protein 8	Arhgap8	NM_028455	1.6
cadherin-like 24	Cdh24	NM_199470	1.6
Unknown	Gm13777	XM_205095	1.6
major facilitator superfamily domain containing 8	Mfsd8	NM_028140	1.6
NOP56 ribonucleoprotein homolog (yeast)	Nop56	NM_024193	1.6
AIG2-like domain 1	A2ld1	NM_145466	1.6
intraflagellar transport 122 homolog (Chlamydomonas)	Ift122	NM_031177	1.6
family with sequence similarity 26, member E	Fam26e	NM_178908	1.6
ficolin A	Fcna	NM_007995	1.6
3-hydroxybutyrate dehydrogenase, type 1	Bdh1	NM_175177	1.6
mevalonate kinase	Mvk	NM_023556	1.6
coiled-coil domain containing 125	Ccdc125	NM_183115	1.6
MTOR associated protein, LST8 homolog (S. cerevisiae)	Mlst8	NM_019988	1.6
brachyury 2	T2	NM_001161832	1.6
unc-119 homolog B (C. elegans)	Unc119b	NM_175352	1.6
splicing factor, arginine/serine-rich 7	Sfrs7	NM_146083	1.6
expressed sequence AI597468	AI597468	NM_001013028	1.6
RIKEN cDNA 6330409N04 gene	6330409N04Rik	NM_025697	1.6
leucine-rich repeats and immunoglobulin-like domains 2	Lrig2	NM_001025067	1.6
predicted gene 5779	Gm5779	BC082775	1.6
RIKEN cDNA A630089N07 gene	A630089N07Rik	ENSMUST00000099483	1.6
serine dehydratase	Sds	NM_145565	1.6
leucine rich repeat and Ig domain containing 4	Lingo4	NM_177250	1.6
Ras-related GTP binding B	Rragb	NM_001004154	1.6
RIKEN cDNA A930001N09 gene	A930001N09Rik	BC113191	1.6
transformed mouse 3T3 cell double minute 4	Mdm4	NM_008575	1.6
golgi transport 1 homolog B (S. cerevisiae)	Golt1b	NM_025872	1.6
cyclin-dependent kinase inhibitor 2A	Cdkn2a	NM_009877	1.6
RIKEN cDNA 3632451O06 gene	3632451O06Rik	BC023359	1.6
uridine phosphorylase 2	Upp2	ENSMUST00000071543	1.6
succinate dehydrogenase complex, subunit B, iron sulfur (Ip)	Sdhb	NM_023374	1.6
lymphocyte antigen 6 complex, locus F	Ly6f	NM_008530	1.6

Additional Table 2b. Genderwise differentially expressed genes (p ≤0.01) with a regulation of ≥ 1.5

DAVID GeneName	Gene Symbol	RefSeq	Fold Change Female/Males
claudin 5	Cldn5	NM_013805	1.6
RIKEN cDNA 2410129H14 gene	2410129H14Rik	BC117005	1.6
chloride channel 4-2	Clcn4-2	NM_011334	1.6
family with sequence similarity 114, member A2	Fam114a2	BC019518	1.6
armadillo repeat containing, X-linked 5	Armcx5	NM_001009575	1.6
RIKEN cDNA 4933430I17 gene	4933430I17Rik	BC116650	1.6
zinc finger protein 191	Zfp191	NM_021559	1.6
purinergic receptor P2X, ligand-gated ion channel, 1	P2rx1	NM_008771	1.6
leucine rich repeat containing 29	Lrrc29	BC034290	1.6
shugoshin-like 1 (S. pombe)	Sgol1	NM_028232	1.6
protein phosphatase 1D magnesium-dependent, delta isoform	Ppm1d	NM_016910	1.6
RIKEN cDNA 6720456H20 gene	6720456H20Rik	NM_172600	1.6
TBK1 binding protein 1	Tbkbp1	NM_198100	1.6
vacuolar protein sorting 37C (yeast)	Vps37c	NM_181403	1.6
polymerase (RNA) II (DNA directed) polypeptide D	Polr2d	NM_027002	1.6
Unknown	LOC433770	XR_031848	1.6
microtubule-associated protein 2	Mtap2	NM_001039934	1.6
NADH-ubiquinone oxidoreductase chain 3	ND3	ENSMUST00000082411	1.6
zinc finger protein 781	Zfp781	NM_199062	1.6
sarcoglycan, gamma (dystrophin-associated glycoprotein)	Sgcg	NM_011892	1.6
FERM and PDZ domain containing 3	Frmpd3	ENSMUST00000050767	1.6
transmembrane protein 168	Tmem168	NM_028990	1.6
carboxymethylenebutenolidase-like (Pseudomonas)	Cmbl	NM_181588	1.6
motor neuron and pancreas homeobox 1	Mnx1	ENSMUST00000001608	1.6
ADP-ribosylation factor-like 1	Arl1	NM_025859	1.6
NADH dehydrogenase (ubiquinone) 1 beta subcomplex, 2	Ndufb2	NM_026612	1.6
vasoactive intestinal peptide receptor 1	Vipr1	NM_011703	1.6
cytochrome P450, family 46, subfamily a, polypeptide 1	Cyp46a1	NM_010010	1.6
protein-L-isoaspartate (D-aspartate) O-methyltransferase 1	Pcmt1	NM_008786	1.6
endosulfine alpha	Ensa	NM_001026212	1.6
predicted gene 5972	Gm5972	NR_003650	1.6
nicolin 1	Nicn1	NM_025449	1.6
phosphatidylserine decarboxylase	Pisd	NM_177298	1.6
forkhead box O3	Foxo3	ENSMUST00000105502	1.6
mitochondrial ribosomal protein L32	Mrpl32	NM_029271	1.6
stratifin	Sfn	NM_018754	1.6
claspin homolog (Xenopus laevis)	Clspn	NM_175554	1.6
aspartyl-tRNA synthetase 2 (mitochondrial)	Dars2	NM_172644	1.6
multiple EGF-like-domains 10	Megf10	NM_001001979	1.6
esterase 22	Es22	NM_133660	1.6
mitochondrial ribosomal protein L42	Gm6419	XM_887794	1.6
ER degradation enhancer, mannosidase alpha-like 1	Edem1	NM_138677	1.6
leucine rich repeat and sterile alpha motif containing 1	Lrsam1	NM_199302	1.6
chloride channel, nucleotide-sensitive, 1A	Clns1a	NM_023671	1.6
Kruppel-like factor 4 (gut)	Klf4	NM_010637	1.6
glutathione S-transferase, mu 4	Gstm4	NM_026764	1.6
Kruppel-like factor 14	Klf14	NM_001135093	1.6
solute carrier family 2 (facilitated glucose transporter), member 1	Slc2a1	NM_011400	1.6
kelch domain containing 7A	Klhdc7a	NM_173427	1.6
peroxisomal biogenesis factor 7	Pex7	NM_008822	1.6

Additional Table 2b. Genderwise differentially expressed genes (p ≤0.01) with a regulation of ≥ 1.5

DAVID GeneName	Gene Symbol	RefSeq	Fold Change Female/Males
EMI domain containing 2	Emid2	NM_024474	1.6
RIKEN cDNA 6330407J23 gene	6330407J23Rik	NM_026138	1.6
DNA segment, Chr 2, ERATO Doi 750, expressed	D2Ertd750e	NM_026412	1.6
GPI anchor attachment protein 1	Gpaa1	NM_010331	1.6
Tctex1 domain containing 4	Tctex1d4	NM_175030	1.6
EF-hand calcium binding domain 7	Efcab7	ENSMUST00000097959	1.6
Unknown	Hist1h4d	NM_175654	1.6
UDP-GlcNAc:betaGal beta-1,3-N-acetylglucosaminyltransferase 2	B3gnt2	NM_016888	1.6
arginine vasopressin	Avp	NM_009732	1.6
zinc finger, matrin type 2	Zmat2	NM_025594	1.6
zinc finger protein 92	Zfp92	NM_009566	1.6
protein kinase C substrate 80K-H	Prkcsh	NM_008925	1.6
RIKEN cDNA 1810010K12 gene	1810010K12Rik	ENSMUST00000070378	1.6
apolipoprotein D	Apod	NM_007470	1.6
APOBEC1 complementation factor	A1cf	NM_001081074	1.6
RAB20, member RAS oncogene family	Rab20	NM_011227	1.6
SEH1-like (S. cerevisiae	Seh1l	NM_001039088	1.6
anoctamin 10	Ano10	NM_133979	1.6
RIKEN cDNA 8030411F24 gene	8030411F24Rik	NM_030135	1.6
Ts translation elongation factor, mitochondrial	Tsfm	NM_025537	1.6
lymphoid enhancer binding factor 1	Lef1	NM_010703	1.6
WD repeat domain 23	Dcaf11	NM_133734	1.6
formin 1	Fmn1	NM_010230	1.6
protein phosphatase 2, regulatory subunit B'', gamma	Ppp2r3c	NM_021529	1.6
galactosidase, beta 1	Glb1	NM_009752	1.6
similar to odorant response abnormal 4	BC003331	NM_145511	1.6
timeless interacting protein	Tipin	NM_025372	1.6
GC-rich promoter binding protein 1	Gpbp1	NM_001122963	1.6
autophagy-related 3 (yeast)	Atg3	NM_026402	1.6
interleukin 5 receptor, alpha	Il5ra	NM_008370	1.6
AT rich interactive domain 5A (MRF1-like)	Arid5a	NM_145996	1.6
fibronectin type III and SPRY domain containing 1-like	Fsd1l	NM_176966	1.6
predicted gene 14115	Gm14115	XR_002266	1.6
PRELI domain containing 2	Prelid2	NM_029942	1.6
yippee-like 1 (Drosophila)	Ypel1	NM_023249	1.6
cytotoxic T lymphocyte-associated protein 2 beta	Ctla2b	NM_007797	1.6
zinc finger and BTB domain containing 43	Zbtb43	NM_027947	1.6
mitochondrial ribosomal protein S33	Mrps33	NM_010270	1.6
glutamine repeat protein 1	Glrp1	NM_008132	1.6
progesterone receptor membrane component 2	Pgrmc2	NM_027558	1.6
RIKEN cDNA 4921501E09 gene	4921501E09Rik	NM_001009544	1.6
eosinophil peroxidase	Epx	NM_007946	1.6
mediator complex subunit 30	Med30	NM_027212	1.6
ASF1 anti-silencing function 1 homolog A (S. cerevisiae)	Asf1a	NM_025541	1.6
eukaryotic translation initiation factor 4H	Eif4h	NM_033561	1.6
tryptophan hydroxylase 1	Tph1	NM_009414	1.6
osteoclast stimulating factor 1	Ostf1	NM_017375	1.6
NOL1/NOP2/Sun domain family, member 4	Nsun4	NM_028142	1.6
cleavage and polyadenylation factor subunit homolog (S. cerevisiae)	Pcf11	NM_029078	1.6
ATPase, H+ transporting, lysosomal V1 subunit C1	Atp6v1c1	NM_025494	1.6

Additional Table 2b. Genderwise differentially expressed genes (p ≤0.01) with a regulation of ≥ 1.5

DAVID GeneName	Gene Symbol	RefSeq	Fold Change Female/Males
ATPase, H+ transporting, lysosomal V0 subunit E2	Atp6v0e2	NM_133764	1.6
keratin 15	Krt15	NM_008469	1.6
zinc ribbon domain containing, 1	Znrd1	NM_023162	1.6
predicted gene 9876	Gm9876	ENSMUST00000064248	1.6
Unknown	Gm11263	XR_031917	1.6
mitochondrial ribosomal protein S27	Mrps27	NM_173757	1.6
ubiquitin-conjugating enzyme E2B, RAD6 homology (S. cerevisiae)	Ube2b	NM_009458	1.6
ProSAPiP1 protein	RP23-100C5.8	NM_197945	1.6
TNF receptor-associated factor 6	Traf6	NM_009424	1.6
3-phosphoglycerate dehydrogenase	Phgdh	NM_016966	1.6
mannosidase 2, alpha B1	Man2b1	NM_010764	1.6
mitochondrial ribosomal protein L50	Mrpl50	NM_178603	1.6
sema domain, immunoglobulin domain (Ig), short basic domain, secreted, (semaphorin) 3A	Sema3a	NM_009152	1.6
protein phosphatase 1B, magnesium dependent, beta isoform	Ppm1b	NM_001159496	1.6
PC4 and SFRS1 interacting protein 1	Psip1	NM_133948	1.6
RIKEN cDNA 1110013L07 gene	Fam189b	BC088990	1.6
UDP-GlcNAc:betaGal beta-1,3-N-acetylglucosaminyltransferase 5	B3gnt5	NM_001159407	1.6
DNA segment, Chr 6, Wayne State University 163, expressed	D6Wsu163e	BC016429	1.6
sterol carrier protein 2, liver	Scp2	NM_011327	1.6
alanyl-tRNA synthetase domain containing 1	1700113I22Rik	NM_026865	1.6
dihydrouridine synthase 4-like (S. cerevisiae)	Dus4l	NM_028002	1.6
aryl-hydrocarbon receptor	Ahr	NM_013464	1.6
phosphatidic acid phosphatase type 2A	Ppap2a	NM_008247	1.6
RIKEN cDNA 5430411K18 gene	5430411K18Rik	AK122536	1.6
mitogen-activated protein kinase kinase 3	Map2k3	NM_008928	1.6
complement component (3b/4b) receptor 1-like	Cr1l	NM_013499	1.6
neuropeptide FF-amide peptide precursor	Npff	NM_018787	1.6
histone aminotransferase 1	Hat1	NM_026115	1.6
C1q and tumor necrosis factor related protein 2	C1qtnf2	NM_026979	1.6
N-acetylneuraminic acid phosphatase	Nanp	NM_026086	1.6
T cell receptor alpha variable 13D-4	Trav13d-4	ENSMUST00000103607	1.6
RIKEN cDNA C030048H21 gene	C030048H21Rik	XM_923021	1.6
D-2-hydroxyglutarate dehydrogenase	D2hgdh	NM_178882	1.6
serine/arginine repetitive matrix 1	Srrm1	NM_016799	1.6
myosin VIIA and Rab interacting protein	Myrip	NM_144557	1.6
signal transducer and activator of transcription 2	Stat2	NM_019963	1.6
armadillo repeat containing, X-linked 4	Armcx4	ENSMUST00000055842	1.6
tectorin alpha	Tecta	NM_009347	1.6
cardiotrophin 1	Ctf1	NM_007795	1.6
dual oxidase 2	Duox2	NM_177610	1.6
Bardet-Biedl syndrome 2 (human)	Bbs2	NM_026116	1.6
synaptosomal-associated protein, 47	Snap47	NM_144521	1.6
ArfGAP with FG repeats 2	Agfg2	NM_145566	1.6
keratin 77	Krt77	NM_001003667	1.6
cDNA sequence BC050254	BC050254	BC050254	1.6
predicted gene 7290	Gm7290	XM_976302	1.6
RIKEN cDNA 5730419I09 gene	5730419I09Rik	NM_029081	1.6
ADP-ribosylation factor 2	Arf2	NM_007477	1.6
heat shock protein 70 family, member 13	Hspa13	NR_027492	1.6

Additional Table 2b. Genderwise differentially expressed genes (p ≤0.01) with a regulation of ≥ 1.5

DAVID GeneName	Gene Symbol	RefSeq	Fold Change Female/Males
SAR1 gene homolog A (S. cerevisiae)	Sar1a	NM_009120	1.6
coiled-coil domain containing 61	Ccdc61	NM_001033314	1.6
CCAAT/enhancer binding protein (C/EBP), epsilon	Cebpe	NM_207131	1.6
mitochondrial ribosomal protein L30	Mrpl30	NM_027098	1.6
DEK oncogene (DNA binding)	Dek	NM_025900	1.6
fibroblast growth factor binding protein 3	Fgfbp3	NM_028263	1.6
family with sequence similarity 122, member B	Fam122b	NM_030167	1.6
sterile alpha motif domain containing 9-like	Samd9l	NM_010156	1.6
spermatogenesis associated 5	Spata5	NM_021343	1.6
activity regulated cytoskeletal-associated protein	Arc	NM_018790	1.6
mannoside acetylglucosaminyltransferase 5, isoenzyme B	Mgat5b	NM_172948	1.6
transmembrane protein 91	Tmem91	NM_177102	1.6
apolipoprotein O	Apoo	NM_026673	1.6
homeo box D10	Hoxd10	NM_013554	1.6
CCR4-NOT transcription complex, subunit 2	Cnot2	NR_024327	1.6
predicted gene 6534	Gm6534	NM_001037923	1.6
tetratricopeptide repeat domain 37	Ttc37	NM_001081352	1.6
dihydrouridine synthase 3-like (S. cerevisiae)	Dus3l	NM_144858	1.6
cDNA sequence BC005764	BC005764	NM_181681	1.6
SKI-like	Skil	NM_011386	1.6
poly (ADP-ribose) polymerase family, member 11	Parp11	NM_181402	1.6
interferon-induced protein with tetratricopeptide repeats 1	Ifit1	NM_008331	1.6
suppressor of variegation 4-20 homolog 2 (Drosophila)	Suv420h2	NM_146177	1.6
RIKEN cDNA 2410017P07 gene	2410017P07Rik	NM_029132	1.6
SERTA domain containing 4	Sertad4	NM_198247	1.6
ring finger protein, transmembrane 1	Rnft1	NM_029788	1.6
RIKEN cDNA E130203B14 gene	E130203B14Rik	NM_178791	1.6
muted	Muted	NM_139063	1.6
predicted gene 9955	Gm9955	ENSMUST00000067987	1.6
speckle-type POZ protein	Spop	NM_025287	1.6
bromodomain containing 9	Brd9	NM_001024508	1.6
mesenchyme homeobox 2	Meox2	NM_008584	1.6
DNA segment, Chr 10, Wayne State University 102, expressed	D10Wsu102e	NM_026579	1.6
apolipoprotein O-like	Apool	NM_026565	1.6
G protein-coupled receptor 135	Gpr135	NM_181752	1.6
gremlin 1	Grem1	NM_011824	1.6
apolipoprotein L domain containing 1	Apold1	NM_001109914	1.6
ATP/GTP binding protein-like 5	Agbl5	NM_001048192	1.6
methionine aminopeptidase 2	Metap2	NM_019648	1.6
RIKEN cDNA 9330182L06 gene	9330182L06Rik	BC132290	1.6
zinc finger protein 422, related sequence 1	Zfp422-rs1	NM_029952	1.6
zinc finger protein 94	Zfp94	NM_009568	1.6
Der1-like domain family, member 3	Derl3	NM_024440	1.6
predicted gene 9028	Gm9028	XR_034415	1.6
inhibitor of growth family, member 5	Ing5	NM_025454	1.6
prefoldin 4	Pfdn4	NM_001110152	1.6
von Willebrand factor A domain containing 5B2	Vwa5b2	NM_001144953	1.6
cation channel, sperm associated 2	Catsper2	NM_153075	1.6
RIKEN cDNA 4921511C04 gene	Vwa3b	BC050813	1.6
hydroxypyruvate isomerase homolog (E. coli)	Hyi	NR_003946	1.6

Additional Table 2b. Genderwise differentially expressed genes (p ≤0.01) with a regulation of ≥ 1.5

DAVID GeneName	Gene Symbol	RefSeq	Fold Change Female/Males
pleckstrin homology domain containing, family F (with FYVE domain) member 1	Plekhf1	NM_024413	1.6
adenosine kinase	Adk	NM_134079	1.6
mitochondrial ribosomal protein L1	Mrpl1	NM_053158	1.6
TAF9B RNA polymerase II, TATA box binding protein (TBP)-associated factor	Taf9b	NM_001001176	1.6
EST AA881470	AA881470	NM_172724	1.6
myc target 1	Myct1	NM_026793	1.6
potassium channel, subfamily K, member 15	Kcnk15	BC147256	1.6
transmembrane protein 222	Tmem222	NM_025667	1.6
major facilitator superfamily domain containing 7A	Mfsd7a	NM_172883	1.6
calcium channel, voltage-dependent, L type, alpha 1S subunit	Cacna1s	NM_014193	1.6
ferredoxin 1	Fdx1	NM_007996	1.6
brain protein 44	Brp44	NM_027430	1.6
vesicle-associated membrane protein 3	Vamp3	NM_009498	1.6
EBNA1 binding protein 2	Ebna1bp2	NM_026932	1.6
predicted gene 15653	Gm15653	XR_034092	1.6
tryptophan rich basic protein	Wrb	NM_207301	1.6
mbt domain containing 1	Mbtd1	BC064014	1.6
transient receptor potential cation channel, subfamily C, member 4	Trpc4	NM_016984	1.6
TYRO3 protein tyrosine kinase 3	Tyro3	NM_019392	1.5
dysbindin (dystrobrevin binding protein 1) domain containing 1	Dbndd1	NM_028146	1.5
serine/threonine kinase 19	Stk19	NM_019442	1.5
RAB1, member RAS oncogene family	Rab1	NM_008996	1.5
tripartite motif-containing 21	Trim21	NM_009277	1.5
RIKEN cDNA 2810021B07 gene	2810021B07Rik	NM_025479	1.5
galactosylceramidase	Galc	NM_008079	1.5
Unknown	Gm4607	XR_034166	1.5
cytoplasmic polyadenylation element binding protein 3	Cpeb3	NM_198300	1.5
par-6 partitioning defective 6 homolog gamma (C. elegans)	Pard6g	NM_053117	1.5
phosphopantothenoylcysteine synthetase	Ppcs	NM_026494	1.5
nudix (nucleoside diphosphate linked moiety X)-type motif 17	Nudt17	NM_030094	1.5
caveolin 1, caveolae protein	Cav1	NM_007616	1.5
thromboxane A synthase 1, platelet	Tbxas1	NM_011539	1.5
5-methyltetrahydrofolate-homocysteine methyltransferase	Mtr	NM_001081128	1.5
RIKEN cDNA 2810410L24 gene	2810410L24Rik	ENSMUST00000100157	1.5
cellular nucleic acid binding protein	Cnbp	NM_013493	1.5
tetratricopeptide repeat domain 23	Ttc23	NM_025905	1.5
RIKEN cDNA 6330408A02 gene	6330408A02Rik	NM_177312	1.5
synapsin I	Syn1	NM_013680	1.5
Unknown	Gm8174	XR_001896	1.5
cytochrome P450, family 4, subfamily a, polypeptide 10	Cyp4a10	NM_010011	1.5
interferon regulatory factor 6	Irf6	NM_016851	1.5
phosphodiesterase 6H, cGMP-specific, cone, gamma	Pde6h	NM_023898	1.5
RIKEN cDNA C920025E04 gene	C920025E04Rik	ENSMUST00000079918	1.5
RNA binding motif protein 12B	Rbm12b	NM_028226	1.5
vestigial like 4 (Drosophila)	Vgll4	NM_177683	1.5
inhibitor of growth family, member 1	Ing1	NM_011919	1.5
alpha 1 microglobulin/bikunin	Ambp	NM_007443	1.5
N-acetylglutamate synthase	Nags	NM_145829	1.5
enoyl Coenzyme A hydratase domain containing 1	Echdc1	NM_025855	1.5
Unknown	Rpl15	NM_025586	1.5

Additional Table 2b. Genderwise differentially expressed genes (p ≤0.01) with a regulation of ≥ 1.5

DAVID GeneName	Gene Symbol	RefSeq	Fold Change Female/Males
chordin-like 2	Chrdl2	NM_133709	1.5
embryonic ectoderm development	Eed	NM_021876	1.5
neurturin	Nrtn	NM_008738	1.5
fibronectin type III domain containing 4	Fndc4	NM_022424	1.5
exoribonuclease 1	Eri1	NM_026067	1.5
ADP-ribosylation factor-like 6 interacting protein 1	Arl6ip1	NM_019419	1.5
glutaredoxin 3	Glrx3	NM_023140	1.5
zinc finger and BTB domain containing 34	Zbtb34	NM_001085507	1.5
Bmi1 polycomb ring finger oncogene	Bmi1	NM_007552	1.5
selenium binding protein 2	Selenbp2	NM_019414	1.5
nicotinamide nucleotide adenylyltransferase 1	Nmnat1	NM_133435	1.5
cholinergic receptor, nicotinic, delta polypeptide	Chrnd	NM_021600	1.5
RIKEN cDNA 2610044O15 gene	2610044O15Rik	NM_153780	1.5
RIKEN cDNA 2010107G23 gene	2010107G23Rik	BC024943	1.5
UDP-GlcNAc:betaGal beta-1,3-N-acetylglucosaminyltransferase 1	B3gnt1	NM_175383	1.5
aldo-keto reductase family 1, member C20	Akr1c20	NM_054080	1.5
neugrin, neurite outgrowth associated	Ngrn	NM_031375	1.5
aspartate dehydrogenase domain containing	Aspdh	NM_026690	1.5
RIKEN cDNA 1110001A16 gene	1110001A16Rik	ENSMUST00000063817	1.5
transmembrane protein 47	Tmem47	NM_175771	1.5
enhancer of mRNA decapping 4	Edc4	NM_181594	1.5
integrin beta 1 binding protein 1	Itgb1bp1	NM_008403	1.5
leucine-rich repeat LGI family, member 3	Lgi3	NM_145219	1.5
CDC like kinase 4	Clk4	NM_007714	1.5
RIKEN cDNA 3110007F17 gene	3110007F17Rik	BC027572	1.5
arginyltransferase 1	Ate1	NM_013799	1.5
GPN-loop GTPase 3	Gpn3	NM_024216	1.5
N-acetyltransferase 12	Nat12	NM_001081430	1.5
axin 1	Axin1	NM_001159598	1.5
FAD-dependent oxidoreductase domain containing 1	Foxred1	NM_172291	1.5
acyl-CoA thioesterase 1	Acot1	NM_012006	1.5
steroid 5 alpha-reductase 2-like 2	Tecrl	NM_153801	1.5
even skipped homeotic gene 1 homolog	Evx1	NM_007966	1.5
F-box and leucine-rich repeat protein 5	Fbxl5	NM_001159963	1.5
RIKEN cDNA I830127L07 gene	I830127L07Rik	XM_909906	1.5
predicted gene 9104	Gm9104	NM_001111313	1.5
predicted gene 6614	Gm6614	NM_001081318	1.5
transmembrane emp24 protein transport domain containing 5	Tmed5	NM_028876	1.5
solute carrier family 6 (neurotransmitter transporter, betaine/GABA), member 12	Slc6a12	NM_133661	1.5
lymphocyte antigen 6 complex, locus G6F	Ly6g6f	ENSMUST00000038507	1.5
NADH dehydrogenase (ubiquinone) 1, subcomplex unknown, 1	Ndufc1	NM_025523	1.5
zinc finger, AN1 type domain 2B	Zfand2b	NM_026846	1.5
MIS12 homolog (yeast)	Mis12	NM_025993	1.5
NADH dehydrogenase (ubiquinone) 1 alpha subcomplex, 2	Ndufa2	NM_010885	1.5
prolyl endopeptidase-like	Prepl	NM_145984	1.5
Unknown	Gm10713	XM_001474680	1.5
SPRY domain containing 4	Spryd4	NM_025716	1.5
hypothetical protein LOC100048632	Gm1604b	BC147041	1.5
Unknown	1110020C03Rik	BC062805	1.5

Additional Table 2b. Genderwise differentially expressed genes (p ≤0.01) with a regulation of ≥ 1.5

DAVID GeneName	Gene Symbol	RefSeq	Fold Change Female/Males
cysteine and histidine-rich domain (CHORD)-containing, zinc-binding protein 1	Chordc1	NM_025844	1.5
potassium channel tetramerisation domain containing 12b	Kctd12b	NM_175429	1.5
C-type lectin domain family 14, member a	Clec14a	NM_025809	1.5
phospholipase C, beta 2	Plcb2	NM_177568	1.5
peptidase (mitochondrial processing) alpha	Pmpca	NM_173180	1.5
neurexin II	Nrxn2	NM_020253	1.5
platelet endothelial aggregation receptor 1	Pear1	NM_028460	1.5
cell division cycle 20 homolog B (S. cerevisiae)	Cdc20b	ENSMUST00000109244	1.5
EPM2A (laforin) interacting protein 1	Epm2aip1	NM_175266	1.5
ankyrin repeat domain 6	Ankrd6	NM_001012450	1.5
runt related transcription factor 2	Runx2	NM_001146038	1.5
solute carrier family 19 (sodium/hydrogen exchanger), member 1	Slc19a1	NM_031196	1.5
phosphatidylinositol glycan anchor biosynthesis, class K	Pigk	NM_025662	1.5
mastermind like 3 (Drosophila)	Maml3	NM_001004176	1.5
kelch-like 22 (Drosophila)	Klhl22	NM_145479	1.5
origin recognition complex, subunit 5-like (S. cerevisiae)	Orc5l	NM_011959	1.5
hematopoietic SH2 domain containing	Hsh2d	NM_197944	1.5
synaptotagmin XIII	Syt13	NM_030725	1.5
destrin	Dstn	NM_019771	1.5
RIKEN cDNA F630043A04 gene	F630043A04Rik	NM_198605	1.5
solute carrier family 31, member 1	Slc31a1	NM_175090	1.5
mitochondrial ribosomal protein S30	Mrps30	NM_021556	1.5
dopey family member 1	Dopey1	NM_177208	1.5
RIKEN cDNA 2210010B09 gene	Zfp846	NM_172919	1.5
RIKEN cDNA 4922502D21 gene	4922502D21Rik	BC089479	1.5
alpha 1,4-galactosyltransferase	A4galt	NM_001004150	1.5
protein tyrosine phosphatase, receptor type, R	Ptprr	NM_011217	1.5
asteroid homolog 1 (Drosophila)	Aste1	NM_025651	1.5
IQ motif containing E	Iqce	NM_028833	1.5
HNF1 homeobox B	Hnf1b	BC025189	1.5
RB-associated KRAB repressor	Rbak	NM_021326	1.5
mitochondrial ribosomal protein L35	Mrpl35	NM_025430	1.5
adenomatosis polyposis coli 2	Apc2	NM_011789	1.5
regulatory factor X-associated protein	Rfxap	NM_133231	1.5
alcohol dehydrogenase 5 (class III), chi polypeptide	Adh5	NM_007410	1.5
small VCP/p97-interacting protein	Svip	NM_001160345	1.5
BTB (POZ) domain containing 17	Btbd17	NM_028055	1.5
poly(A) polymerase gamma	Papolg	NM_172555	1.5
WD repeat domain 67	Wdr67	NM_001081396	1.5
a disintegrin-like and metallopeptidase (reprolysin type) with thrombospondin type 1 motif, 7	Adamts7	NM_001003911	1.5
small nuclear ribonucleoprotein D3	Snrpd3	NM_026095	1.5
predicted gene 9372	Gm9372	XM_980593	1.5
general transcription factor IIH, polypeptide 5	Gtf2h5	NM_181392	1.5
receptor (calcitonin) activity modifying protein 1	Ramp1	NM_016894	1.5
WAP four-disulfide core domain 5	Wfdc5	NM_145369	1.5
kelch domain containing 4	Klhdc4	ENSMUST00000045884	1.5
ring finger protein 113A2	Rnf113a2	NM_025525	1.5
cancer susceptibility candidate 1	Casc1	NM_177222	1.5

Additional Table 2b. Genderwise differentially expressed genes (p ≤0.01) with a regulation of ≥ 1.5

DAVID GeneName	Gene Symbol	RefSeq	Fold Change Female/Males
RecQ protein-like	Recql	NM_023042	1.5
Unknown	Mpv17	NM_008622	1.5
WSC domain containing 2	Wscd2	NM_177292	1.5
mediator of RNA polymerase II transcription, subunit 12 homolog (yeast)	Med12	NM_021521	1.5
Unknown	LOC633944	XR_032386	1.5
ARP10 actin-related protein 10 homolog (S. cerevisiae)	Actr10	NM_019785	1.5
zinc finger protein 788	Zfp788	NM_023363	1.5
macrophage receptor with collagenous structure	Marco	NM_010766	1.5
RIKEN cDNA 1500041N16 gene	1500041N16Rik	NM_026399	1.5
mitochondrial ribosomal protein S28	Mrps28	NM_025434	1.5
zinc finger protein 322A	Zfp322a	NM_001111107	1.5
Bardet-Biedl syndrome 7 (human)	Bbs7	NM_027810	1.5
RIKEN cDNA C330018D20 gene	C330018D20Rik	ENSMUST00000025488	1.5
bone morphogenetic protein 4	Bmp4	NM_007554	1.5
spermatid perinuclear RNA binding protein	Strbp	NM_009261	1.5
FUN14 domain containing 1	Fundc1	NM_028058	1.5
family with sequence similarity 126, member B	Fam126b	NM_172513	1.5
RUN and SH3 domain containing 1	Rusc1	NM_028188	1.5
blocked early in transport 1 homolog (S. cerevisiae)	Bet1	NM_009748	1.5
FERM, RhoGEF and pleckstrin domain protein 2	Farp2	NM_145519	1.5
predicted gene 10786	Gm10786	ENSMUST00000099550	1.5
Luc7 homolog (S. cerevisiae)-like	Luc7l	BC055875	1.5
cyclin M1	Cnnm1	NM_031396	1.5
aldehyde dehydrogenase family 3, subfamily A1	Aldh3a1	NM_007436	1.5
predicted gene 5039	Gm5039	NR_003647	1.5
ankyrin repeat and SOCS box-containing 13	Asb13	NM_178283	1.5
prostaglandin D2 synthase (brain)	Ptgds	NM_008963	1.5
inositol polyphosphate-5-phosphatase F	Inpp5f	NM_178641	1.5
polymerase (DNA directed), delta 1, catalytic subunit	Pold1	NM_011131	1.5
slowmo homolog 2 (Drosophila)	Slmo2	NM_025531	1.5
Unknown	Gm4929	XR_031578	1.5
family with sequence similarity 43, member A	Fam43a	NM_177632	1.5
AF4/FMR2 family, member 3	Aff3	NM_010678	1.5
WD repeat and SOCS box-containing 1	Wsb1	NM_019653	1.5
RIKEN cDNA 2410003K15 gene	2410003K15Rik	ENSMUST00000114500	1.5
dehydrogenase/reductase (SDR family) member 7C	Dhrs7c	NM_001013013	1.5
suppressor of Ty 4 homolog 1 (S. cerevisiae)	Supt4h1	NM_009296	1.5
tectonic family member 2	Tctn2	NM_026486	1.5
a disintegrin and metallopeptidase domain 19 (meltrin beta)	Adam19	NM_009616	1.5
SWI/SNF related, matrix associated, actin dependent regulator of chromatin, subfamily e, member 1	Smarce1	NM_020618	1.5
cytochrome P450, family 2, subfamily s, polypeptide 1	Cyp2s1	NM_028775	1.5
REX2, RNA exonuclease 2 homolog (S. cerevisiae)	Rexo2	NM_024233	1.5
hect domain and RLD 4	Herc4	NM_026101	1.5
tissue factor pathway inhibitor 2	Tfpi2	NM_009364	1.5
endo-beta-N-acetylglucosaminidase	Engase	NM_172573	1.5
predicted gene 7560	Gm7560	XR_032183	1.5
lysophospholipase 1	Lypla1	NM_008866	1.5
ubiquitin-conjugating enzyme E2G 1 (UBC7 homolog, C. elegans)	Ube2g1	NM_025985	1.5
methyltransferase like 8	Mettl8	NM_145524	1.5

Additional Table 2b. Genderwise differentially expressed genes (p ≤0.01) with a regulation of ≥ 1.5

DAVID GeneName	Gene Symbol	RefSeq	Fold Change Female/Males
RIKEN cDNA E130009J12 gene	E130009J12Rik	NM_001008973	1.5
UFM1-specific peptidase 1	Ufsp1	NM_027356	1.5
RIKEN cDNA 5830416P10 gene	5830416P10Rik	ENSMUST00000025996	1.5
retinoic acid receptor, beta	Rarb	NM_011243	1.5
a disintegrin and metallopeptidase domain 5	Adam5	NM_007401	1.5
leucine rich repeat containing 1	Lrrc1	NM_001146048	1.5
N-glycanase 1	Ngly1	NM_021504	1.5
cytochrome c oxidase subunit IV isoform 2	Cox4i2	NM_053091	1.5
LIM homeobox protein 8	Lhx8	NM_010713	1.5
ubiquinol-cytochrome c reductase binding protein	Uqcrb	NM_026219	1.5
glycoprotein m6b	Gpm6b	NM_023122	1.5
similar to eukaryotic translation elongation factor 1 gamma	LOC100047986	XR_033846	1.5
interferon-related developmental regulator 1	Ifrd1	NM_013562	1.5
Unknown	Gm5070	XR_034004	1.5
SH3-binding domain glutamic acid-rich protein	Sh3bgr	NM_015825	1.5
phosphatidic acid phosphatase type 2 domain containing 1A	Ppapdc1a	NM_001080963	1.5
selectin, platelet (p-selectin) ligand	Selplg	NM_009151	1.5
synaptojanin 2 binding protein	Synj2bp	NM_025292	1.5
uroporphyrinogen III synthase	Uros	NM_009479	1.5
peroxisomal biogenesis factor 13	Pex13	NM_023651	1.5
integrin beta 4	Itgb4	NM_001005608	1.5
