# Supplementary material for: Sex-dimorphism in Cardiac Nutrigenomics: effect of Trans fat and/or Monosodium Glutamate consumption
Source: BMC Genomics. 2011 Nov 12;12:555. doi: 10.1186/1471-2164-12-555 (PMC3238303; doi:10.1186/1471-2164-12-555)
Supplement: Additional file 3 — Table S3. Gene ontologies & pathways enriched for differentially expressed genes comparing all males to all females. [file 1471-2164-12-555-S3.PDF]

Additional Table 3. Gene ontologies &pathways enriched for differentially expressed genes comparing all males to all females regardless of diet.

Gene Ontology ID	Category / Pathway	No. of genes	*P* Value
**Ratio > 1.5 (relatively upregulated in males)**			
**Biological Processes**			
GO:0043170	Macromolecule metabolic process	75	0.04
GO:0044249	Cellular biosynthetic process	51	0.01
GO:0006807	Nitrogen compound metabolic process	51	0.03
GO:0009058	Biosynthetic process	51	0.01
GO:0032502	Developmental process	47	0.01
GO:0006139	Nucleobase, nucleoside, nucleotide and nucleic acid metabolic process	46	0.04
GO:0010468	Regulation of gene expression	43	0.01
GO:0048856	Anatomical structure development	43	<.01
GO:0001568	Blood vessel development	8	0.03
GO:0030029	Actin filament-based process	7	0.02
GO:0003007	Heart morphogenesis	5	0.01
**Cellular Component**			
GO:0005622	Intracellular	136	0.01
GO:0005856	Cytoskeleton	22	0.02
GO:0044424	Intracellular part	129	0.02
GO:0044459	Plasma membrane part	29	0.03
**KEGG PATHWAY**			
mmu04810	Regulation of actin cytoskeleton	6	0.04
mmu04612	Antigen processing and presentation	4	0.04
**Ratio <1.5 (relatively upregulated in females)**			
**Biological Processes**			
GO:0009987	Cellular process	86	<.001
GO:0008152	Metabolic process	68	0.003
GO:0006807	Nitrogen compound metabolic process	37	0.01
GO:0042180	Cellular ketone metabolic process	9	0.03
GO:0006520	Cellular amino acid metabolic process	7	0.003
GO:0006487	Protein amino acid N-linked glycosylation	3	0.01
**Cellular Component**			
GO:0005737	Cytoplasm	58	0.05
**Molecular Function**			
GO:0004857	Enzyme inhibitor activity	8	<.01
GO:0005488	Binding	95	0.02
**KEGG PATHWAY**			
mmu00270	Cysteine and methionine metabolism	3	0.03
