# Supplementary material for: Sex-dimorphism in Cardiac Nutrigenomics: effect of Trans fat and/or Monosodium Glutamate consumption
Source: BMC Genomics. 2011 Nov 12;12:555. doi: 10.1186/1471-2164-12-555 (PMC3238303; doi:10.1186/1471-2164-12-555)
Supplement: Additional file 4 — Table S4. Differentially expressed genes in either males or females for the comparison TFA vs Control with respect to diet and sex (P < 0.01) and fold change ≥ ± 1.5. [file 1471-2164-12-555-S4.PDF]

Additional Table 4. Fold Change ≥ 1.5 in either males or females for the comparison TFA vs Control amongst the differentially expressed genes with respect to diet as well as sex (P<0.01).

Gene Names	Gene Symbol	RefSeq	Fold Change TFA / Control (Males)	Fold Change TFA / Control (Females)
fibrillin 2	Fbn2	NM_010181	13.0	1.6
dihydropyrimidinase	Dpys	NM_022722	7.7	1.5
tumor necrosis factor (ligand) superfamily, member 14	Tnfsf14	NM_019418	7.5	1.9
methyl-CpG binding domain protein 4	Mbd4	NM_010774	6.4	2.0
cDNA sequence BC027072	BC027072	BC046516	4.8	2.2
polymerase (DNA directed), iota	Poli	NM_011972	4.6	1.6
claspin homolog (Xenopus laevis)	Clspn	NM_175554	4.3	1.5
amyotrophic lateral sclerosis 2 (juvenile) chromosome region, candidate 4	Als2cr4	NM_001033449	4.2	1.5
secretory leukocyte peptidase inhibitor	Slpi	NM_011414	4.0	1.1
tyrosine aminotransferase	Tat	NM_146214	4.0	1.4
calcium/calmodulin-dependent protein kinase II, beta	Camk2b	BC080273	3.9	2.0
sclerostin	Sost	NM_024449	3.5	-1.0
calcyphosphine 2	Caps2	NM_178278	3.5	1.6
PDZ domain containing 3	Pdzd3	NM_133226	3.2	1.6
dysbindin (dystrobrevin binding protein 1) domain containing 1	Dbndd1	NM_028146	3.2	1.5
small nuclear ribonucleoprotein 35 (U11/U12)	Snrnp35	NM_029532	3.1	1.9
RIKEN cDNA 1700041C02 gene	Ccdc30	NM_029286	3.0	1.5
formiminotransferase cyclodeaminase	Ftcd	NM_080845	3.0	1.6
predicted gene 14325	Gm14325	ENSMUST00000108939	3.0	1.3
zinc finger protein 13	Zfp13	NM_011747	3.0	1.3
Unknown	3100002L24Rik	U62393	2.9	1.1
Unknown	MUSG00000016609	NM_001100416	2.9	1.1
Unknown	100043387	NM_001099327	2.9	1.1
Ethanol induced 1	Etohi1	ENSMUST00000098999	2.9	1.1
phosphoserine aminotransferase 1	Psat1	NM_177420	2.8	1.5
copine II	Cpne2	NM_153507	2.8	1.3
RIKEN cDNA 6720416L17 gene	6720416L17Rik	ENSMUST00000100000	2.6	1.6
platelet-derived growth factor receptor-like	Pdgfrl	NM_026840	2.6	1.6
transmembrane protease, serine 11f	Tmprss11f	NM_178730	2.6	1.8
chemokine (C-X3-C) receptor 1	Cx3cr1	NM_009987	2.5	1.7
zinc finger protein 97	Zfp97	NM_011765	2.4	1.1
myosin 1H	Myo1h	BC144867	2.4	2.8
tachykinin receptor 3	Tacr3	NM_021382	2.4	1.0
plastin 3 (T-isoform)	Pls3	NM_145629	2.3	-1.0
amylase 1, salivary	Amy1	NM_007446	2.3	1.3
predicted gene 10828	Gm10828	ENSMUST00000100068	2.3	1.1
apoptosis-inducing factor, mitochondrion-associated 3	Aifm3	NM_175178	2.2	1.6
heme binding protein 1	Hebp1	NM_013546	2.2	1.2
hairy and enhancer of split 2 (Drosophila)	Hes2	NM_008236	2.2	1.2
dynein, axonemal, light chain 1	Dnalc1	NM_028821	2.2	1.7
predicted gene 14403	Gm14403	ENSMUST00000108947	2.2	1.1
inhibitor of growth family, member 4	Ing4	NM_133345	2.2	1.2
non-SMC condensin I complex, subunit H	Ncaph	NM_144818	2.2	1.7
chromatin modifying protein 5	Chmp5	NM_029814	2.1	1.1
cyclin-dependent kinase inhibitor 2A	Cdkn2a	NM_009877	2.1	-1.0
BR serine/threonine kinase 2	Brsk2	NM_001009930	2.1	1.4
integral membrane protein 2A	Itm2a	NM_008409	2.1	-1.1
magnesium transporter 1	Magt1	NM_025952	2.1	1.2

Additional Table 4. Fold Change ≥ 1.5 in either males or females for the comparison TFA vs Control amongst the differentially expressed genes with respect to diet as well as sex (P<0.01).

Gene Names	Gene Symbol	RefSeq	Fold Change TFA / Control (Males)	Fold Change TFA / Control (Females)
BCL2-interacting killer	Bik	NM_007546	2.1	2.5
aryl-hydrocarbon receptor	Ahr	NM_013464	2.1	1.7
asparagine-linked glycosylation 6 homolog (yeast, alpha-1,3,-glucosyltransferase)	Alg6	NM_001081264	2.1	1.1
5-methyltetrahydrofolate-homocysteine methyltransferase	Mtr	NM_001081128	2.0	-1.1
glia maturation factor, beta	Gmfb	NM_022023	2.0	-1.0
limb expression 1 homolog (chicken)	Lix1	NM_025681	2.0	-1.4
RNA binding motif protein 4	Rbm4	NM_009032	2.0	1.0
predicted gene 13089	Gm13089	ENSMUST00000073532	2.0	2.4
CUB domain containing protein 2	Cdcp2	NM_172873	2.0	1.2
glutamate receptor, ionotropic, kainate 3	Grik3	NM_001081097	1.9	1.3
Unknown	Gm5188	ENSMUST00000037962	1.9	1.4
RIKEN cDNA 2900083I11 gene	2900083I11Rik	NM_021403	1.9	1.5
coiled-coil domain containing 39	Ccdc39	NM_026222	1.9	1.9
ATPase, class V, type 10B	Atp10b	ENSMUST00000056678	1.9	1.3
cytochrome P450, family 20, subfamily A, polypeptide 1	Cyp20a1	NM_030013	1.9	1.4
tetraspanin 6	Tspan6	NM_019656	1.9	-1.4
RIKEN cDNA 2010305A19 gene	2010305A19Rik	BC012391	1.9	1.2
small Cajal body-specific RNA 17	Scarna17	AF357342	1.8	1.5
fructose bisphosphatase 2	Fbp2	NM_007994	1.8	1.6
S1 RNA binding domain 1	Srbd1	NM_030133	1.8	1.5
serine/threonine kinase 36 (fused homolog, Drosophila)	Stk36	NM_175031	1.8	2.5
spondin 2, extracellular matrix protein	Spon2	NM_133903	1.8	-1.1
predicted gene 7278	Gm7278	XR_034437	1.8	1.6
transmembrane protein 69	Tmem69	NM_177670	1.8	1.1
TATA box binding protein (Tbp)-associated factor, RNA polymerase I, A	Taf1a	NM_021466	1.8	1.3
asporin	Aspn	NM_025711	1.8	1.2
glutathione S-transferase, mu 4	Gstm4	NM_026764	1.8	1.0
RIKEN cDNA B230354K17 gene	B230354K17Rik	ENSMUST00000097319	1.8	1.3
general transcription factor II H, polypeptide 2	Gtf2h2	NM_022011	1.8	1.3
3-hydroxy-3-methylglutaryl-Coenzyme A synthase 2	Hmgcs2	NM_008256	1.8	1.6
forkhead box B2	Foxb2	NM_008023	1.8	1.5
RIKEN cDNA 2810021B07 gene	2810021B07Rik	NM_025479	1.8	1.5
galectin-related inter-fiber protein	Grifin	NM_030022	1.8	1.6
hyperpolarization-activated, cyclic nucleotide-gated K+ 1	Hcn1	NM_010408	1.8	-1.9
progestin and adipoQ receptor family member V	Paqr5	NM_028748	1.8	2.6
Bmi1 polycomb ring finger oncogene	Bmi1	NM_007552	1.8	-1.1
transmembrane protein 190	Tmem190	ENSMUST00000013235	1.8	2.3
coiled-coil domain containing 60	Ccdc60	ENSMUST00000086483	1.8	1.0
WD repeat and FYVE domain containing 2	Wdfy2	NM_175546	1.7	1.6
cullin 5	Cul5	NM_027807	1.7	1.1
integrin beta 3	Itgb3	NM_016780	1.7	-1.0
RIKEN cDNA 4933408B17 gene	4933408B17Rik	NM_177773	1.7	-2.3
NADH dehydrogenase (ubiquinone) 1, subcomplex unknown, 1	Ndufc1	NM_025523	1.7	1.1
mediator of RNA polymerase II transcription, subunit 31 homolog (yeast)	Med31	NM_026068	1.7	-1.1
RIKEN cDNA 2210406O10 gene	2210406O10Rik	ENSMUST00000044964	1.7	1.8
aldehyde dehydrogenase family 1, subfamily A3	Aldh1a3	NM_053080	1.7	1.8
enhancer of yellow 2 homolog (Drosophila)	Eny2	NM_175009	1.7	1.3

Additional Table 4. Fold Change ≥ 1.5 in either males or females for the comparison TFA vs Control amongst the differentially expressed genes with respect to diet as well as sex (P<0.01).

Gene Names	Gene Symbol	RefSeq	Fold Change TFA / Control (Males)	Fold Change TFA / Control (Females)
solute carrier family 22 (organic cation transporter), member 3	Slc22a3	NM_011395	1.7	1.2
TIP41, TOR signalling pathway regulator-like (S. cerevisiae)	Tiprl	NM_145513	1.7	1.1
sine oculis-related homeobox 4 homolog (Drosophila)	Six4	NM_011382	1.7	1.2
protein disulfide isomerase associated 4	Pdia4	NM_009787	1.7	-1.3
topoisomerase (DNA) II binding protein 1	Topbp1	NM_176979	1.7	-1.0
CCR4-NOT transcription complex, subunit 8	Cnot8	NM_026949	1.7	1.1
malignant T cell amplified sequence 2	Mcts2	NM_025543	1.7	1.2
family with sequence similarity 76, member B	Fam76b	BC043120	1.7	-1.1
NEDD4 binding protein 2	N4bp2	NM_001024917	1.7	-1.1
exosome component 8	Exosc8	NM_027148	1.6	-1.1
serine (or cysteine) peptidase inhibitor, clade B, member 5	Serpinb5	NM_009257	1.6	2.7
stearoyl-coenzyme A desaturase 4	Scd4	NM_183216	1.6	1.4
microtubule-associated protein, RP/EB family, member 1	Mapre1	NM_007896	1.6	-1.1
cornichon homolog 4 (Drosophila)	Cnih4	NM_030131	1.6	-1.1
zinc finger protein 640	Zfp640	ENSMUST00000071320	1.6	1.1
leucine rich repeat containing 46	Lrrc46	NM_027026	1.6	1.6
dynein light chain Tctex-type 3	Dynlt3	NM_025975	1.6	-1.1
zinc finger protein 819	Zfp819	NM_028913	1.6	1.5
secretogranin V	Scg5	NM_009162	1.6	1.4
uroporphyrinogen decarboxylase	Urod	NM_009478	1.6	1.1
THO complex 2	Thoc2	NM_001033422	1.6	1.1
poly (ADP-ribose) polymerase family, member 14	Parp14	NM_001039530	1.6	1.2
non-SMC element 1 homolog (S. cerevisiae)	Nsmce1	NM_026330	1.6	1.4
cholinergic receptor, nicotinic, beta polypeptide 3	Chrnb3	NM_173212	1.6	1.8
vacuolar protein sorting 13A (yeast)	Vps13a	NM_173028	1.6	1.2
keratin 75	Krt75	NM_133357	1.6	-1.1
RIKEN cDNA 2410015M20 gene	2410015M20Rik	BC056164	1.6	1.1
phospholipase B domain containing 1	Plbd1	NM_025806	1.6	1.3
aspartylglucosaminidase	Aga	NM_001005847	1.6	-1.1
G0/G1 switch gene 2	G0s2	NM_008059	1.6	1.1
related RAS viral (r-ras) oncogene homolog 2	Rras2	NM_025846	1.6	-1.0
mitochondrial ribosomal protein S14	Mrps14	NM_025474	1.6	-1.2
solute carrier family 45, member 2	Slc45a2	NM_053077	1.6	-1.1
RIKEN cDNA I830127L07 gene	I830127L07Rik	XM_909906	1.6	1.0
F-box protein 30	Fbxo30	NM_027968	1.6	1.3
dopey family member 1	Dopey1	NM_177208	1.6	1.3
predicted gene 5532	Gm5532	ENSMUST00000097531	1.6	1.4
ribosomal protein S11	Gm6394	XM_915925	1.6	1.2
inter-alpha trypsin inhibitor, heavy chain 3	Itih3	NM_008407	1.5	2.0
histone deacetylase 2	Hdac2	NM_008229	1.5	-1.4
unc-93 homolog A (C. elegans)	Unc93a	NM_199252	1.5	1.4
tryptophan hydroxylase 1	Tph1	NM_009414	1.5	2.3
LSM6 homolog, U6 small nuclear RNA associated (S. cerevisiae)	Lsm6	NM_030145	1.5	-1.0
RIKEN cDNA 2310001A20 gene	2310001A20Rik	AJ310638	1.5	-1.1
guanine nucleotide binding protein (G protein), beta 4	Gnb4	NM_013531	1.5	1.3
RIKEN cDNA 4631405J19 gene	4631405J19Rik	BC100298	1.5	1.4
LAS1-like (S. cerevisiae)	Las1l	NM_152822	1.5	-1.0
protein phosphatase 3, regulatory subunit B, alpha isoform (calcineurin B, type I)	Ppp3r1	NM_024459	1.5	-1.2

Additional Table 4. Fold Change ≥ 1.5 in either males or females for the comparison TFA vs Control amongst the differentially expressed genes with respect to diet as well as sex (P<0.01).

Gene Names	Gene Symbol	RefSeq	Fold Change TFA / Control (Males)	Fold Change TFA / Control (Females)
glucose-fructose oxidoreductase domain containing 2	Gfod2	NM_027469	1.5	2.0
proteasome (prosome, macropain) 26S subunit, non-ATPase, 14	Psmd14	NM_021526	1.5	1.1
5-hydroxytryptamine (serotonin) receptor 6	Htr6	NM_021358	1.5	1.0
dehydrogenase/reductase (SDR family) member 7	Dhrs7	NM_025522	1.5	-1.1
hydroxy-delta-5-steroid dehydrogenase, 3 beta- and steroid delta-isomerase 7	Hsd3b7	NM_133943	1.5	-1.2
staufen (RNA binding protein) homolog 2 (Drosophila)	Stau2	NM_001111272	1.5	1.4
phosphodiesterase 3B, cGMP-inhibited	Pde3b	NM_011055	1.4	-1.9
heat-responsive protein 12	Hrsp12	NM_008287	1.4	1.7
RIKEN cDNA 6720456H20 gene	6720456H20Rik	NM_172600	1.4	1.6
KDEL (Lys-Asp-Glu-Leu) endoplasmic reticulum protein retention receptor 3	Kdelr3	NM_134090	1.4	-1.7
coiled-coil domain containing 138	Ccdc138	NM_001162956	1.4	1.8
guanylate cyclase 1, soluble, alpha 2	Gucy1a2	NM_001033322	1.3	1.5
elastin microfibril interfacer 1	Emilin1	NM_133918	1.3	1.8
proteoglycan 4 (megakaryocyte stimulating factor, articular superficial zone protein)	Prg4	NM_021400	1.3	-2.6
nebulin	Neb	NM_010889	1.3	1.8
protease, serine, 23	Prss23	NM_029614	1.3	-1.9
transient receptor potential cation channel, subfamily V, member 5	Trpv5	NM_001007572	1.3	1.9
tripartite motif-containing 25	Trim25	NM_009546	1.2	1.6
RAS, guanyl releasing protein 2	Rasgrp2	NM_011242	1.2	2.2
dolichyl pyrophosphate phosphatase 1	Dolpp1	NM_020329	1.2	-1.5
midkine	Mdk	NM_010784	1.2	-1.5
bromodomain and WD repeat domain containing 1	Brwd1	NM_145125	1.2	1.6
G protein-coupled receptor kinase 4	Grk4	NM_019497	1.2	1.8
cation channel, sperm associated 1	Catsper1	NM_139301	1.2	1.6
Luc7 homolog (S. cerevisiae)-like	Luc7l	BC055875	1.2	1.7
predicted gene 11435	Gm11435	NM_001045543	1.2	1.7
zinc finger protein 239	Zfp239	NM_001001792	1.2	2.7
2-4-dienoyl-Coenzyme A reductase 2, peroxisomal	Decr2	NM_011933	1.1	1.7
cryptochrome 1 (photolyase-like)	Cry1	NM_007771	1.1	-2.1
GRB10 interacting GYF protein 1	Gigyf1	NM_031408	1.1	1.8
signal transducer and activator of transcription 2	Stat2	NM_019963	1.1	1.7
hemopoietic cell kinase	Hck	NM_010407	1.1	-5.6
patatin-like phospholipase domain containing 3	Pnpla3	NM_054088	1.0	-2.1
transcription elongation factor A (SII)-like 8	Tceal8	NM_025703	1.0	-2.1
protein tyrosine phosphatase-like (proline instead of catalytic arginir	Ptplb	NM_023587	1.0	-1.7
serine dehydratase	Sds	NM_145565	1.0	1.7
ADP-ribosylation factor-like 4D	Arl4d	NM_025404	-1.0	-1.7
branched chain aminotransferase 1	Bcat1	NM_001024468	-1.0	-1.7
pleckstrin homology domain containing, family H (with MyTH4 domain) member 1	Plekhh1	AK122464	-1.1	1.9
membrane-spanning 4-domains, subfamily A, member 2	Ms4a2	NM_013516	-1.1	1.9
intermediate filament family orphan 2	Iffo2	NM_183148	-1.1	1.9
THAP domain containing, apoptosis associated protein 2	Thap2	NM_025780	-1.1	-1.8
interferon inducible GTPase 1	Iigp1	NM_021792	-1.1	-1.8
potassium voltage-gated channel, shaker-related subfamily, member 5	Kcna5	NM_145983	-1.1	1.5
RIKEN cDNA A830039N20 gene	A830039N20Rik	BC038501	-1.1	1.6

Additional Table 4. Fold Change ≥ 1.5 in either males or females for the comparison TFA vs Control amongst the differentially expressed genes with respect to diet as well as sex (P<0.01).

Gene Names	Gene Symbol	RefSeq	Fold Change TFA / Control (Males)	Fold Change TFA / Control (Females)
acyl-CoA thioesterase 12	Acot12	NM_028790	-1.1	3.5
predicted gene 5127	Gm5127	NM_001033541	-1.1	1.5
myosin VIIA	Myo7a	NM_008663	-1.1	1.5
leucine rich repeat containing 26	Lrrc26	NM_146117	-1.1	1.7
olfactory receptor 146	Olfr146	NM_146747	-1.2	1.6
Rap guanine nucleotide exchange factor (GEF) 3	Rapgef3	NM_144850	-1.2	1.5
RIKEN cDNA 1700020N15 gene	Gm6812	NM_001098842	-1.2	-2.0
threonine synthase-like 2 (bacterial)	Thnsl2	NM_178413	-1.3	-2.2
growth arrest-specific 2 like 1	Gas2l1	NM_030228	-1.3	-1.7
LAG1 homolog, ceramide synthase 4	Lass4	NM_026058	-1.3	-1.5
RIKEN cDNA 4932418E24 gene	4932418E24Rik	NM_177841	-1.3	-1.9
integrin alpha 2b	Itga2b	NM_010575	-1.4	-1.9
inositol polyphosphate 5-phosphatase J	Inpp5j	NM_172439	-1.4	-1.9
insulin-like growth factor binding protein 3	Igfbp3	NM_008343	-1.4	-2.0
preproenkephalin	Penk	NM_001002927	-1.4	-1.9
asparagine-linked glycosylation 10 homolog B (yeast, alpha-1,2-glucosyltransferase)	Alg10b	NM_001033441	-1.5	-1.1
nuclear factor I/C	Nfic	NM_008688	-1.5	1.3
myosin light chain kinase 3	Mylk3	NM_175441	-1.5	1.0
kin of IRRE like 3 (Drosophila)	Kirrel3	BC063072	-1.5	1.4
tubulin polymerization-promoting protein family member 3	Tppp3	NM_026481	-1.5	-1.7
NIMA (never in mitosis gene a)-related expressed kinase 11	Nek11	NM_172461	-1.5	1.0
thiosulfate sulfurtransferase, mitochondrial	Tst	NM_009437	-1.5	-1.2
RIKEN cDNA 8030423F21 gene	8030423F21Rik	ENSMUST00000064097	-1.5	-1.3
prospero-related homeobox 1	Prox1	NM_008937	-1.5	-1.3
gene trap locus F3b	Gtlf3b	NM_025294	-1.5	1.2
dystroglycan 1	Dag1	NM_010017	-1.5	1.1
G protein-coupled receptor 161	Gpr161	NM_001081126	-1.5	-1.0
protein kinase domain containing, cytoplasmic	Pkdcc	NM_134117	-1.5	-2.2
synaptosomal-associated protein 29	Snap29	NM_023348	-1.5	-2.0
serine/arginine repetitive matrix 2	Srrm2	NM_175229	-1.5	1.0
HLA-B-associated transcript 3	Bat3	NM_057171	-1.5	-1.0
upstream binding protein 1	Ubp1	NM_001083319	-1.5	1.0
cAMP responsive element binding protein 5	Creb5	ENSMUST00000114409	-1.5	-1.5
predicted gene 10397	Gm10397	ENSMUST00000100823	-1.5	-1.1
adenosine A1 receptor	Adora1	NM_001008533	-1.5	-1.5
leucine rich repeat containing 61	Lrrc61	NM_177736	-1.5	-1.2
forkhead box J2	Foxj2	NM_021899	-1.5	1.0
pericentrin (kendrin)	Pcnt	NM_008787	-1.5	1.1
lipase, endothelial	Lipg	NM_010720	-1.5	-1.3
protocadherin gamma subfamily A, 1	Pcdhga1	NM_033584	-1.5	-1.1
single-stranded DNA binding protein 3	Ssbp3	NM_023672	-1.5	1.1
LIM domain binding 1	Ldb1	NM_001113408	-1.5	-1.4
cryptochrome 2 (photolyase-like)	Cry2	NM_009963	-1.5	-1.1
heparan sulfate 6-O-sulfotransferase 1	Hs6st1	NM_015818	-1.5	1.0
HD domain containing 2	Hddc2	NM_027168	-1.5	-1.8
paxillin	Pxn	NM_011223	-1.5	1.2
G protein-coupled receptor kinase-interactor 1	Git1	NM_001004144	-1.5	1.2
carbonic anhydrase 5b, mitochondrial	Car5b	NM_181315	-1.5	-7.0

Additional Table 4. Fold Change ≥ 1.5 in either males or females for the comparison TFA vs Control amongst the differentially expressed genes with respect to diet as well as sex (P<0.01).

Gene Names	Gene Symbol	RefSeq	Fold Change TFA / Control (Males)	Fold Change TFA / Control (Females)
ring finger protein 126	Rnf126	NM_144528	-1.5	-1.1
hexokinase 1	Hk1	NM_001146100	-1.5	-1.3
pleckstrin homology-like domain, family A, member 1	Phlda1	NM_009344	-1.5	-1.0
SAPS domain family, member 2	Saps2	NM_026813	-1.6	1.2
guanine nucleotide binding protein, alpha transducing 2	Gnat2	NM_008141	-1.6	-1.1
transcription factor E3	Tcfe3	NM_172472	-1.6	-1.4
mediator of RNA polymerase II transcription, subunit 12 homolog (yeast)-like	Med12l	NM_177855	-1.6	-2.4
RIKEN cDNA C030046I01 gene	C030046I01Rik	NM_177994	-1.6	1.1
homeo box B3	Hoxb3	NM_001079869	-1.6	1.0
ubiquitin associated protein 2-like	Ubap2l	NM_028475	-1.6	-1.1
hairy/enhancer-of-split related with YRPW motif-like	Heyl	NM_013905	-1.6	-1.1
LIM and cysteine-rich domains 1	Lmcd1	NM_144799	-1.6	-1.0
synaptopodin 2-like	Synpo2l	NM_175132	-1.6	-1.3
family with sequence similarity 120, member A	Fam120a	NM_001033268	-1.6	1.1
component of oligomeric golgi complex 1	Cog1	NM_013581	-1.6	-1.4
heat shock protein family, member 7 (cardiovascular)	Hspb7	NM_013868	-1.6	-1.0
predicted gene 10524	Gm10524	ENSMUST00000097503	-1.6	-1.0
transforming growth factor beta regulated gene 4	Tbrg4	NM_134011	-1.6	1.1
purine rich element binding protein A	Pura	NM_008989	-1.6	1.0
zinc finger, CCHC domain containing 14	Zcchc14	NM_080855	-1.6	-1.1
ADAMTS-like 3	Adamtsl3	XM_984557	-1.6	1.3
Notch gene homolog 4 (Drosophila)	Notch4	NM_010929	-1.6	-1.1
KDEL (Lys-Asp-Glu-Leu) endoplasmic reticulum protein retention receptor 1	Kdelr1	NM_133950	-1.6	-1.1
IQ motif containing H	Iqch	NM_030068	-1.6	1.1
ATPase, Cu++ transporting, beta polypeptide	Atp7b	NM_007511	-1.6	2.1
pleckstrin homology-like domain, family B, member 3	Phldb3	NM_001102613	-1.6	1.2
endothelin converting enzyme 1	Ece1	NM_199307	-1.6	-1.1
zinc finger protein 295	Zfp295	NM_175428	-1.6	-1.0
DCN1, defective in cullin neddylation 1, domain containing 4 (S.	Dcun1d4	NM_178896	-1.6	-1.5
interferon regulatory factor 2 binding protein 2	Irf2bp2	BC048951	-1.6	-1.1
RNA binding protein with multiple splicing 2	Rbpms2	NM_028030	-1.6	-1.4
netrin 1	Ntn1	NM_008744	-1.6	1.2
ATPase, aminophospholipid transporter (APLT), class I, type 8A, member 1	Atp8a1	NM_001038999	-1.6	1.1
interferon regulatory factor 2 binding protein 1	Irf2bp1	NM_178757	-1.6	1.2
integrin alpha FG-GAP repeat containing 2	Itfg2	NM_133927	-1.6	-1.1
F-box protein 31	Fbxo31	NM_133765	-1.6	1.0
katanin p60 (ATPase-containing) subunit A1	Katna1	NM_011835	-1.6	1.0
G protein-coupled receptor 85	Gpr85	NM_145066	-1.6	1.4
zinc finger protein 362	Zfp362	NM_001081098	-1.6	-1.2
pellino 1	Peli1	NM_023324	-1.7	-1.5
lysophosphatidic acid receptor 5	Lpar5	AK131863	-1.7	-1.1
splicing factor 1	Sf1	NM_001110791	-1.7	-1.1
transmembrane protein 127	Tmem127	NM_175145	-1.7	-1.2
Unknown	AU042671	ENSMUST00000065641	-1.7	-1.1
potassium inwardly-rectifying channel, subfamily J, member 12	Kcnj12	NM_010603	-1.7	-1.4
phospholipase C, delta 1	Plcd1	NM_019676	-1.7	1.1
predicted gene 1614	Gm1614	ENSMUST00000097619	-1.7	1.1

Additional Table 4. Fold Change ≥ 1.5 in either males or females for the comparison TFA vs Control amongst the differentially expressed genes with respect to diet as well as sex (P<0.01).

Gene Names	Gene Symbol	RefSeq	Fold Change TFA / Control (Males)	Fold Change TFA / Control (Females)
transmembrane protein 131	Tmem131	NM_018872	-1.7	1.0
myosin, heavy polypeptide 8, skeletal muscle, perinatal	Myh8	NM_177369	-1.7	-1.2
GH3 domain containing	Ghdc	NM_031871	-1.7	-1.2
FK506 binding protein 8	Fkbp8	NM_001111066	-1.7	1.1
potassium voltage-gated channel, subfamily H (eag-related), member 1	Kcnh1	NM_010600	-1.7	1.1
oxysterol binding protein-like 6	Osbpl6	NM_145525	-1.7	1.1
unc-5 homolog C (C. elegans)-like	Unc5cl	NM_152823	-1.7	1.0
polymerase (DNA-directed), delta interacting protein 2	Poldip2	NM_026389	-1.7	1.1
branched chain ketoacid dehydrogenase kinase	Bckdk	NM_009739	-1.7	-1.2
solute carrier family 6 (neurotransmitter transporter, noradrenalin), member 2	Slc6a2	NM_009209	-1.7	-1.5
RIKEN cDNA 1700054N08 gene	1700054N08Rik	BC036300	-1.7	1.1
xenotropic and polytropic retrovirus receptor 1	Xpr1	NM_011273	-1.7	1.2
REX1, RNA exonuclease 1 homolog (S. cerevisiae)	Rexo1	NM_025852	-1.7	1.1
HEAT repeat containing 6	Heatr6	NM_145432	-1.7	1.3
mesoderm posterior 1	Mesp1	NM_008588	-1.7	-1.0
zinc finger protein 358	Zfp358	NM_080461	-1.7	-1.1
dehydrogenase/reductase (SDR family) member 11	Dhrs11	NM_177564	-1.7	-1.0
neuronal pentraxin 1	Nptx1	NM_008730	-1.7	1.0
myotubularin related protein 12	Mtmr12	NM_172958	-1.7	1.1
proviral integration site 3	Pim3	NM_145478	-1.8	1.1
HLA-B associated transcript 2-like	Bat2l	NM_001159634	-1.8	1.0
heat shock factor 1	Hsf1	NM_008296	-1.8	1.1
PDX1 C-terminal inhibiting factor 1	Pcif1	NM_146129	-1.8	-1.0
mediator of DNA damage checkpoint 1	Mdc1	NM_001010833	-1.8	-1.4
Sp2 transcription factor	Sp2	NM_030220	-1.8	-1.2
similar to Unknown (protein for IMAGE:4910858)	ND6	ENSMUST00000082419	-1.8	1.2
GATS protein-like 2	Gatsl2	NM_030719	-1.8	-1.7
Unknown	AF362573	AF362573	-1.8	-1.1
coronin, actin binding protein 1B	Coro1b	NM_011778	-1.8	1.1
RAR-related orphan receptor gamma	Rorc	NM_011281	-1.8	1.1
potassium voltage-gated channel, subfamily H (eag-related), membe	Kcnh2	NM_013569	-1.8	-1.2
RGM domain family, member A	Rgma	NM_177740	-1.8	-1.1
BEN domain containing 3	Bend3	NM_199028	-1.8	-1.2
sparc/osteonectin, cwcv and kazal-like domains proteoglycan 2	Spock2	NM_052994	-1.8	-1.3
PCTAIRE-motif protein kinase 1	Pctk1	NM_011049	-1.8	1.1
Unknown	LOC670326	XR_031858	-1.8	-1.1
G protein-coupled receptor 146	Gpr146	NM_030258	-1.8	1.2
cyclin M3	Cnnm3	NM_053186	-1.9	1.2
Jun dimerization protein 2	Jdp2	AB034697	-1.9	1.1
SR-related CTD-associated factor 1	Scaf1	NM_001008422	-1.9	1.1
xin actin-binding repeat containing 1	Xirp1	NM_001081339	-1.9	-1.1
myosin, heavy polypeptide 14	Myh14	NM_028021	-1.9	1.3
potassium inwardly-rectifying channel, subfamily J, member 9	Kcnj9	NM_008429	-1.9	1.1
RIKEN cDNA E130309D14 gene	E130309D14Rik	BC150886	-1.9	1.1
GATA binding protein 4	Gata4	NM_008092	-1.9	-1.2
zinc finger, DHHC domain containing 3	Zdhhc3	NM_026917	-1.9	1.0
eukaryotic translation initiation factor 4E binding protein 2	Eif4ebp2	NM_010124	-1.9	-1.2

Additional Table 4. Fold Change ≥ 1.5 in either males or females for the comparison TFA vs Control amongst the differentially expressed genes with respect to diet as well as sex (P<0.01).

Gene Names	Gene Symbol	RefSeq	Fold Change TFA / Control (Males)	Fold Change TFA / Control (Females)
golgi associated, gamma adaptin ear containing, ARF binding protein 1	Gga1	NM_145929	-1.9	1.2
ring finger protein 113A2	Rnf113a2	NM_025525	-1.9	-1.0
GRAM domain containing 4	Gramd4	NM_172611	-1.9	-1.0
angiomotin-like 1	Amotl1	NM_001081395	-1.9	1.2
predicted gene 5577	Gm5577	AK020089	-1.9	-1.5
ski sarcoma viral oncogene homolog (avian)	Ski	NM_011385	-1.9	-1.1
homeo box C10	Hoxc10	NM_010462	-1.9	-1.1
solute carrier family 7 (cationic amino acid transporter, y+ system), member 1	Slc7a1	NM_007513	-1.9	-1.2
polymerase (RNA) II (DNA directed) polypeptide A	Polr2a	NM_009089	-1.9	-1.3
forkhead box P4	Foxp4	NM_001110824	-1.9	-1.3
myocyte enhancer factor 2D	Mef2d	NM_133665	-1.9	1.1
uridine-cytidine kinase 2	Uck2	NM_030724	-1.9	1.1
phosphodiesterase 6A, cGMP-specific, rod, alpha	Pde6a	NM_146086	-1.9	-1.7
potassium channel tetramerisation domain containing 17	Kctd17	NM_001081367	-1.9	1.0
sprouty homolog 1 (Drosophila)	Spry1	NM_011896	-1.9	1.1
nuclear receptor subfamily 1, group H, member 2	Nr1h2	NM_009473	-1.9	-1.1
arginyl aminopeptidase (aminopeptidase B)-like 1	Rnpepl1	NM_181405	-1.9	1.0
TRAF3 interacting protein 2	Traf3ip2	NM_134000	-2.0	-1.5
dual specificity phosphatase 5	Dusp5	NM_001085390	-2.0	-2.9
RIKEN cDNA 5430437P03 gene	5430437P03Rik	BC005692	-2.0	-1.0
SH3-domain binding protein 4	Sh3bp4	NM_133816	-2.0	-1.0
pleiomorphic adenoma gene-like 1	Plagl1	NM_009538	-2.0	-2.4
serine/threonine kinase 38 like	Stk38l	NM_172734	-2.0	1.3
sodium channel, voltage-gated, type V, alpha	Scn5a	NM_021544	-2.0	-1.0
bromodomain containing 4	Brd4	NM_020508	-2.0	-1.0
ST6 (alpha-N-acetyl-neuraminyl-2,3-beta-galactosyl-1,3)-N-acetylgalactosaminide alpha-2,6-sialyltransferase 4	St6galnac4	NM_011373	-2.0	-1.3
mitogen-activated protein kinase kinase 4	Map2k4	NM_009157	-2.0	-1.2
PHD finger protein 2	Phf2	NM_011078	-2.0	1.2
salt inducible kinase 1	Sik1	NM_010831	-2.0	1.3
transcription factor 3	Tcf3	NM_001079822	-2.0	-1.3
coactosin-like 1 (Dictyostelium)	Cotl1	NM_028071	-2.1	-1.6
fibrosin	Fbrs	NM_010183	-2.1	1.1
septin 14	1700017B05Rik	NM_028820	-2.1	-1.1
midnolin	Midn	NM_021565	-2.1	1.1
LIM-domain containing, protein kinase	Limk1	NM_010717	-2.1	1.0
RGP1 retrograde golgi transport homolog (S. cerevisiae)	Rgp1	NM_172866	-2.1	1.1
CDC42 effector protein (Rho GTPase binding) 1	Cdc42ep1	NM_027219	-2.1	-1.0
zinc finger protein 42	Zfp42	NM_009556	-2.2	1.3
predicted gene 12824	Gm12824	NM_001085549	-2.2	1.1
leucine rich repeat containing 3B	Lrrc3b	NM_146052	-2.2	-2.2
tribbles homolog 1 (Drosophila)	Trib1	NM_144549	-2.2	1.1
RIKEN cDNA 4432412L15 gene	4432412L15Rik	NM_028877	-2.2	-1.1
v-rel reticuloendotheliosis viral oncogene homolog A (avian)	Rela	NM_009045	-2.2	-1.4
TRAF3 interacting protein 1	Traf3ip1	NM_028718	-2.3	1.0
RIKEN cDNA C230052I12 gene	C230052I12Rik	NM_178643	-2.3	-1.7
glycerophosphodiester phosphodiesterase domain containing 5	Gdpd5	NM_201352	-2.3	-1.2

Additional Table 4. Fold Change ≥ 1.5 in either males or females for the comparison TFA vs Control amongst the differentially expressed genes with respect to diet as well as sex (P<0.01).

Gene Names	Gene Symbol	RefSeq	Fold Change TFA / Control (Males)	Fold Change TFA / Control (Females)
stathmin 1	Stmn1	NM_019641	-2.3	-1.0
epsin 3	Epn3	NM_027984	-2.3	-1.2
HLA-B associated transcript 2	Bat2	NM_020027	-2.3	-1.1
WW domain binding protein 2	Wbp2	NM_016852	-2.3	-1.1
B-cell CLL/lymphoma 9	Bcl9	NM_029933	-2.3	-1.5
male enhanced antigen 1	Mea1	NM_010787	-2.3	-1.1
calcium/calmodulin-dependent protein kinase kinase 1, alpha	Camkk1	NM_018883	-2.3	-1.2
DEP domain containing 1B	Depdc1b	NM_178683	-2.4	1.1
vesicle-associated membrane protein 2	Vamp2	NM_009497	-2.4	-1.2
bridging integrator 3	Bin3	NM_021328	-2.4	1.1
zinc finger and BTB domain containing 7C	Zbtb7c	NM_145356	-2.5	1.2
sterile alpha motif domain containing 4B	Samd4b	NM_175021	-2.5	-1.2
family with sequence similarity 125, member B	Fam125b	BC049129	-2.5	1.1
4-aminobutyrate aminotransferase	Abat	NM_172961	-2.5	-1.8
zinc finger CCCH-type, antiviral 1-like	Zc3hav1l	NM_172467	-2.6	-1.2
microtubule-associated protein 6	Mtap6	NM_010837	-2.6	-1.0
proline rich 7 (synaptic)	Prr7	NM_001030296	-2.6	1.2
tripartite motif-containing 3	Trim3	NM_018880	-2.6	-1.2
period homolog 1 (Drosophila)	Per1	NM_011065	-2.6	-1.1
arylsulfatase B	Arsb	NM_009712	-2.6	-1.3
guanylate cyclase activator 1a (retina)	Guca1a	NM_008189	-2.6	1.0
TSC22 domain family, member 4	Tsc22d4	NM_023910	-2.7	-1.1
keratin 13	Krt13	NM_010662	-2.8	-1.4
histocompatibility 2, M region locus 10.3	H2-M10.3	NM_201608	-2.8	-1.9
predicted gene 88	Gm88	BC147714	-2.9	-1.4
dual-specificity tyrosine-(Y)-phosphorylation regulated kinase 1b	Dyrk1b	NM_001037957	-2.9	-1.0
solute carrier family 41, member 1	Slc41a1	NM_173865	-3.0	-1.5
acetoacetyl-CoA synthetase	Aacs	NM_030210	-3.2	-3.4
ankyrin repeat domain 34A	Ankrd34a	NM_001024851	-3.4	1.1
mitogen-activated protein kinase kinase kinase 15	Map3k15	ENSMUST00000033665	-3.5	-1.3
sterol regulatory element binding factor 2	Srebf2	NM_033218	-3.5	-1.7
PH domain and leucine rich repeat protein phosphatase 2	Phlpp2	NM_001122594	-3.7	-1.1
UDP-N-acetyl-alpha-D-galactosamine:polypeptide N-acetylgalactosaminyltransferase 10	Galnt10	NM_134189	-3.7	1.0
actin-like 6B	Actl6b	NM_031404	-3.8	-1.2
T-cell acute lymphocytic leukemia 2	Tal2	NM_009317	-3.9	1.1
proteasome (prosome, macropain) 26S subunit, ATPase 2	Psmc2	NM_011188	-3.9	-1.5
RIKEN cDNA 6330549D23 gene	6330549D23Rik	NR_003619	-4.0	-1.2
histocompatibility 2, T region locus 10	H2-T10	NM_010395	-4.4	-1.0
kelch-like 3 (Drosophila)	Klhl3	ENSMUST00000091583	-4.9	1.1
Ras association (RalGDS/AF-6) domain family (N-terminal) member 7	Rassf7	NM_025886	-4.9	1.1
sodium channel, voltage-gated, type IX, alpha	Scn9a	NM_018852	-5.3	-3.3
heat shock transcription factor 4	Hsf4	NM_011939	-9.8	-1.3
