# Supplementary material for: Sex-dimorphism in Cardiac Nutrigenomics: effect of Trans fat and/or Monosodium Glutamate consumption
Source: BMC Genomics. 2011 Nov 12;12:555. doi: 10.1186/1471-2164-12-555 (PMC3238303; doi:10.1186/1471-2164-12-555)
Supplement: Additional file 5 — Table S5. Differentially expressed genes in either males or females for the comparison MSG vs Control with respect to diet and sex (P < 0.01) and fold change ≥ ± 1.5. [file 1471-2164-12-555-S5.PDF]

Additional Table 5. Fold Change ≥ 1.5 in either males or females for the comparison MSG vs Control amongst the differentially expressed genes with respect to diet as well as sex (P<0.01).

Gene Names	Gene Symbol	RefSeq	Fold Change MSG / Control (Males)	Fold Change MSG /Control (Females)
tumor necrosis factor (ligand) superfamily, member 14	Tnfsf14	NM_019418	7.9	2.2
dysbindin (dystrobrevin binding protein 1) domain containing 1	Dbndd1	NM_028146	3.0	1.6
claspin homolog (Xenopus laevis)	Clspn	NM_175554	2.9	1.8
solute carrier family 45, member 2	Slc45a2	NM_053077	2.8	1.6
copine II	Cpne2	NM_153507	2.8	1.4
integrin beta 3	Itgb3	NM_016780	2.8	1.5
ATPase, class V, type 10B	Atp10b	ENSMUST00000056678	2.6	1.3
non-SMC condensin I complex, subunit H	Ncaph	NM_144818	2.6	2.2
glutamate receptor, ionotropic, kainate 3	Grik3	NM_001081097	2.5	1.7
phosphoserine aminotransferase 1	Psat1	NM_177420	2.4	1.8
nebulin	Neb	NM_010889	2.3	1.8
RIKEN cDNA E130309F12 gene	E130309F12Rik	NM_178756	2.2	2.0
predicted gene 5127	Gm5127	NM_001033541	2.2	2.0
RIKEN cDNA I830127L07 gene	I830127L07Rik	XM_909906	2.1	1.1
predicted gene 14325	Gm14325	ENSMUST00000108939	2.1	1.3
pancreatic lipase	Pnlip	NM_026925	2.1	1.0
glutathione S-transferase, mu 4	Gstm4	NM_026764	2.0	1.1
RIKEN cDNA 4930539E08 gene	4930539E08Rik	BC117930	2.0	2.0
polymerase (DNA directed), iota	Poli	NM_011972	2.0	1.8
heme binding protein 1	Hebp1	NM_013546	2.0	1.4
transient receptor potential cation channel, subfamily V, member 5	Trpv5	NM_001007572	2.0	3.1
zinc finger protein 239	Zfp239	NM_001001792	1.9	2.7
calcium/calmodulin-dependent protein kinase II, beta	Camk2b	BC080273	1.9	1.6
MYCBP associated protein	Mycbpap	NM_170671	1.9	1.8
general transcription factor II H, polypeptide 2	Gtf2h2	NM_022011	1.9	1.3
cytotoxic and regulatory T cell molecule	Crtam	NM_019465	1.9	2.2
aryl-hydrocarbon receptor	Ahr	NM_013464	1.9	1.7
zinc finger protein 42	Zfp42	NM_009556	1.9	1.4
similar to novel KRAB box and zinc finger, C2H2 type domain containing protein	Etohi1	ENSMUST00000098999	1.9	1.1
unc-93 homolog A	Unc93a	NM_199252	1.9	1.9
multiple EGF-like-domains 10	Megf10	NM_001001979	1.8	1.6
BR serine/threonine kinase 2	Brsk2	NM_001009930	1.8	1.7
zinc finger protein 13	Zfp13	NM_011747	1.8	1.6
predicted gene 10828	Gm10828	ENSMUST00000100068	1.8	1.1
cytochrome P450, family 20, subfamily A, polypeptide 1	Cyp20a1	NM_030013	1.8	1.7
serine/threonine kinase 36 (fused homolog, Drosophila)	Stk36	NM_175031	1.8	4.1
acetyl-Coenzyme A acetyltransferase 3	Acat3	NM_153151	1.8	1.2
inhibitor of growth family, member 4	Ing4	NM_133345	1.8	-1.3
DAZ interacting protein 1	Dzip1	NM_025943	1.8	1.6
progestin and adipoQ receptor family member V	Paqr5	NM_028748	1.8	3.3
tryptophan hydroxylase 1	Tph1	NM_009414	1.7	2.5
predicted gene 14403	Gm14403	ENSMUST00000108947	1.7	-1.1
BRCA2 and CDKN1A interacting protein	Bccip	NM_025392	1.7	1.2
RIKEN cDNA 2010305A19 gene	2010305A19Rik	BC012391	1.7	1.2
5-hydroxytryptamine (serotonin) receptor 6	Htr6	NM_021358	1.7	1.3
Unknown	USG00000016609	NM_001100416	1.7	1.1
Unknown	100043387	NM_001099327	1.7	1.1

Additional Table 5. Fold Change ≥ 1.5 in either males or females for the comparison MSG vs Control amongst the differentially expressed genes with respect to diet as well as sex (P<0.01).

Gene Names	Gene Symbol	RefSeq	Fold Change MSG / Control (Males)	Fold Change MSG /Control (Females)
phosphodiesterase 6C, cGMP specific, cone, alpha prime	Pde6c	NM_033614	1.7	1.4
apoptosis-inducing factor, mitochondrion-associated 3	Aifm3	NM_175178	1.7	2.1
forkhead box B2	Foxb2	NM_008023	1.7	1.8
chemokine (C-X3-C) receptor 1	Cx3cr1	NM_009987	1.7	2.3
Luc7 homolog (S. cerevisiae)-like	Luc7l	BC055875	1.7	1.8
matrix metallopeptidase 28 (epilysin)	Mmp28	NM_080453	1.7	1.1
a disintegrin-like and metallopeptidase (reprolysin type) with thrombospondin type 1 motif, 7	Adamts7	NM_001003911	1.7	1.4
melanoma associated antigen (mutated) 1-like 1	Mum1l1	ENSMUST00000113045	1.7	1.9
Unknown	3100002L24Rik	U62393	1.7	1.1
cyclin-dependent kinase inhibitor 2A	Cdkn2a	NM_009877	1.7	2.1
small Cajal body-specific RNA 17	Scarna17	AF357342	1.7	1.4
Unknown	Gm9568	XR_032493	1.7	1.2
galectin-related inter-fiber protein	Grifin	NM_030022	1.7	2.6
transmembrane protein 69	Tmem69	NM_177670	1.6	1.0
RAS, guanyl releasing protein 2	Rasgrp2	NM_011242	1.6	1.4
Bmi1 polycomb ring finger oncogene	Bmi1	NM_007552	1.6	1.1
protein phosphatase 3, regulatory subunit B, alpha isoform (calcineurin B, type I)	Ppp3r1	NM_024459	1.6	-1.1
RIKEN cDNA 1700041C02 gene	Ccdc30	NM_029286	1.6	2.0
chymotrypsinogen B1	Ctrb1	NM_025583	1.6	1.2
solute carrier family 6 (neurotransmitter transporter, noradrenalin), member 2	Slc6a2	NM_009209	1.6	1.2
estrogen related receptor, beta	Esrrb	NM_011934	1.6	1.3
zinc finger protein 640	Zfp640	ENSMUST00000071320	1.6	1.2
RIKEN cDNA 4933408B17 gene	4933408B17Rik	NM_177773	1.6	2.5
amylase 1, salivary	Amy1	NM_007446	1.6	1.1
cation channel, sperm associated 2	Catsper2	NM_153075	1.6	2.0
related RAS viral (r-ras) oncogene homolog 2	Rras2	NM_025846	1.6	1.2
signal transducer and activator of transcription 2	Stat2	NM_019963	1.6	1.7
RIKEN cDNA 5430416O09 gene	5430416O09Rik	BC147345	1.6	1.2
basic leucine zipper and W2 domains 2	Bzw2	NM_025840	1.5	1.1
predicted gene 4248	Gm4248	XM_001479553	1.5	1.5
COP9 (constitutive photomorphogenic) homolog, subunit 3 (Arabidopsis thaliana)	Cops3	NM_011991	1.5	1.0
zinc finger protein 97	Zfp97	NM_011765	1.5	-1.0
F-box protein 41	Fbxo41	NM_001001160	1.5	1.0
non-SMC element 1 homolog (S. cerevisiae)	Nsmce1	NM_026330	1.5	1.2
homeo box C10	Hoxc10	NM_010462	1.5	1.1
dihydropyrimidinase	Dpys	NM_022722	1.5	1.9
tripartite motif-containing 25	Trim25	NM_009546	1.5	1.4
block of proliferation 1	Bop1	NM_013481	1.5	1.1
fibrillin 2	Fbn2	NM_010181	1.5	2.1
predicted gene 8594	Gm8594	XR_033768	1.5	1.5
tyrosine aminotransferase	Tat	NM_146214	1.5	1.4
uroporphyrinogen decarboxylase	Urod	NM_009478	1.5	1.3
secretogranin V	Scg5	NM_009162	1.5	1.3
patatin-like phospholipase domain containing 3	Pnpla3	NM_054088	1.5	-1.1
catechol-O-methyltransferase 1	Comt1	NM_001111062	1.5	1.1
syntaxin 2	Stx2	NM_007941	1.5	1.3

Additional Table 5. Fold Change ≥ 1.5 in either males or females for the comparison MSG vs Control amongst the differentially expressed genes with respect to diet as well as sex (P<0.01).

Gene Names	Gene Symbol	RefSeq	Fold Change MSG / Control (Males)	Fold Change MSG /Control (Females)
target of myb1 homolog (chicken)	Tom1	NM_011622	1.5	1.2
RIKEN cDNA F630111L10 gene	F630111L10Rik	AK170843	1.5	2.3
S1 RNA binding domain 1	Srbd1	NM_030133	1.5	1.3
RRP9, small subunit (SSU) processome component, homolog (yeast)	Rrp9	NM_145620	1.5	1.5
RIKEN cDNA 2610018G03 gene	2610018G03Rik	NM_133729	1.4	3.0
absent in melanoma 1	Aim1	NM_172393	1.4	1.6
GRB10 interacting GYF protein 1	Gigyf1	NM_031408	1.4	2.2
RIKEN cDNA 6720456H20 gene	6720456H20Rik	NM_172600	1.4	1.6
G protein-coupled receptor 85	Gpr85	NM_145066	1.4	2.2
elastase 2, neutrophil	Ela2	NM_015779	1.4	2.1
calcyphosphine 2	Caps2	NM_178278	1.4	3.6
sodium channel, voltage-gated, type IX, alpha	Scn9a	NM_018852	1.4	-2.5
RIKEN cDNA 2210406O10 gene	2210406O10Rik	ENSMUST00000044964	1.4	2.1
autophagy/beclin 1 regulator 1	Ambra1	NM_172669	1.4	1.5
coiled-coil domain containing 138	Ccdc138	NM_001162956	1.3	2.0
cAMP responsive element binding protein 5	Creb5	ENSMUST00000114409	1.3	-2.0
plasmacytoma variant translocation 1	Pvt1	NR_003368	1.3	1.6
engulfment and cell motility 3, ced-12 homolog (C. elegans)	Elmo3	NM_172760	1.3	1.8
galanin-like peptide	Galp	NM_178028	1.3	1.9
kin of IRRE like 3 (Drosophila)	Kirrel3	BC063072	1.3	1.6
Unknown	Gm5188	ENSMUST00000037962	1.3	1.7
BEN domain containing 3	Bend3	NM_199028	1.3	1.9
CUB domain containing protein 2	Cdcp2	NM_172873	1.2	2.0
protocadherin 8	Pcdh8	NM_021543	1.2	1.6
WD repeat and FYVE domain containing 2	Wdfy2	NM_175546	1.2	1.7
myosin VIIA	Myo7a	NM_008663	1.2	2.0
intermediate filament family orphan 2	Iffo2	NM_183148	1.2	2.4
solute carrier family 26, member 4	Slc26a4	NM_011867	1.2	1.7
predicted gene 7278	Gm7278	XR_034437	1.2	1.7
predicted gene 11435	Gm11435	NM_001045543	1.2	3.8
small nuclear ribonucleoprotein 35 (U11/U12)	Snrnp35	NM_029532	1.2	2.5
nudix (nucleoside diphosphate linked moiety X)-type motif 17	Nudt17	NM_030094	1.2	2.1
membrane-spanning 4-domains, subfamily A, member 2	Ms4a2	NM_013516	1.2	3.8
cholinergic receptor, nicotinic, beta polypeptide 3	Chrnb3	NM_173212	1.2	2.9
integrin alpha 2b	Itga2b	NM_010575	1.2	1.7
RIKEN cDNA 2010109I03 gene	2010109I03Rik	NM_025929	1.2	2.6
mevalonate kinase	Mvk	NM_023556	1.2	1.8
zinc finger protein 819	Zfp819	NM_028913	1.2	1.5
olfactory receptor 146	Olfr146	NM_146747	1.1	1.7
serine (or cysteine) peptidase inhibitor, clade B, member 5	Serpinb5	NM_009257	1.1	5.0
dual oxidase 2	Duox2	NM_177610	1.1	1.5
cation channel, sperm associated 1	Catsper1	NM_139301	1.1	2.6
interleukin 18 receptor accessory protein	Il18rap	NM_010553	1.1	1.6
RNA binding motif protein 4	Rbm4	NM_009032	1.1	2.1
REX1, RNA exonuclease 1 homolog (S. cerevisiae)	Rexo1	NM_025852	1.1	-1.5
RIKEN cDNA A830039N20 gene	A830039N20Rik	BC038501	1.1	1.9
RecQ protein-like	Recql	NM_023042	1.1	1.6

Additional Table 5. Fold Change ≥ 1.5 in either males or females for the comparison MSG vs Control amongst the differentially expressed genes with respect to diet as well as sex (P<0.01).

Gene Names	Gene Symbol	RefSeq	Fold Change MSG / Control (Males)	Fold Change MSG /Control (Females)
cDNA sequence BC027072	BC027072	BC046516	1.1	2.7
elastin microfibril interfacer 1	Emilin1	NM_133918	1.1	1.5
phospholipase A2, group XV	Pla2g15	NM_133792	1.1	1.7
solute carrier family 13 (sodium-dependent dicarboxylate transporter), member 2	Slc13a2	NM_022411	1.1	1.7
NIMA (never in mitosis gene a)-related expressed kinase 11	Nek11	NM_172461	1.1	1.5
RIKEN cDNA C230052I12 gene	C230052I12Rik	NM_178643	1.1	-1.6
WAP four-disulfide core domain 5	Wfdc5	NM_145369	1.1	2.2
glucose-fructose oxidoreductase domain containing 2	Gfod2	NM_027469	1.1	2.2
RIKEN cDNA 6720416L17 gene	6720416L17Rik	ENSMUST00000100000	1.0	2.2
predicted gene 13089	Gm13089	ENSMUST00000073532	1.0	2.7
coiled-coil domain containing 39	Ccdc39	NM_026222	1.0	1.8
secretory leukocyte peptidase inhibitor	Slpi	NM_011414	1.0	1.7
nudix (nucleoside diphosphate linked moiety X)-type motif 2	Nudt2	NM_025539	1.0	1.6
a disintegrin and metallopeptidase domain 5	Adam5	NM_007401	1.0	1.5
ArfGAP with RhoGAP domain, ankyrin repeat and PH domain 3	Arap3	NM_139206	1.0	1.6
formiminotransferase cyclodeaminase	Ftcd	NM_080845	-1.0	2.4
HD domain containing 2	Hddc2	NM_027168	-1.0	-2.3
spondin 2, extracellular matrix protein	Spon2	NM_133903	-1.0	-1.7
lin-54 homolog (C. elegans)	Lin54	NM_001115010	-1.1	-1.9
eukaryotic translation initiation factor 4E binding protein 2	Eif4ebp2	NM_010124	-1.1	-1.6
C1q and tumor necrosis factor related protein 1	C1qtnf1	NM_019959	-1.1	-1.5
RIKEN cDNA 2900083I11 gene	2900083I11Rik	NM_021403	-1.1	1.8
integrin alpha FG-GAP repeat containing 2	Itfg2	NM_133927	-1.1	-1.5
T-cell acute lymphocytic leukemia 2	Tal2	NM_009317	-1.1	-2.0
RIKEN cDNA 2810021B07 gene	2810021B07Rik	NM_025479	-1.1	1.7
coiled-coil domain containing 60	Ccdc60	ENSMUST00000086483	-1.1	2.7
forkhead box P4	Foxp4	NM_001110824	-1.1	-1.5
aldehyde dehydrogenase family 1, subfamily A3	Aldh1a3	NM_053080	-1.1	1.6
leucine rich repeat containing 46	Lrrc46	NM_027026	-1.1	1.7
leucine rich repeat containing 26	Lrrc26	NM_146117	-1.1	2.4
transmembrane protein 190	Tmem190	ENSMUST00000013235	-1.1	2.4
peptidase D	Pepd	NM_008820	-1.2	-1.6
heat shock transcription factor 4	Hsf4	NM_011939	-1.2	-1.7
sterol regulatory element binding factor 2	Srebf2	NM_033218	-1.2	-1.9
ATPase, Cu++ transporting, beta polypeptide	Atp7b	NM_007511	-1.2	4.6
versican	Vcan	NM_001081249	-1.2	-1.8
predicted gene 88	Gm88	BC147714	-1.2	-1.7
proteoglycan 4 (megakaryocyte stimulating factor, articular superficial zone protein)	Prg4	NM_021400	-1.2	-1.6
GH3 domain containing	Ghdc	NM_031871	-1.2	-1.6
PH domain and leucine rich repeat protein phosphatase 2	Phlpp2	NM_001122594	-1.2	-1.5
serine dehydratase	Sds	NM_145565	-1.2	1.8
oxysterol binding protein-like 6	Osbpl6	NM_145525	-1.2	-1.7
hydroxy-delta-5-steroid dehydrogenase, 3 beta- and steroid delta-isomerase 7	Hsd3b7	NM_133943	-1.2	-2.1
lysyl oxidase-like 1	Loxl1	NM_010729	-1.2	-1.9
methyl-CpG binding domain protein 4	Mbd4	NM_010774	-1.2	1.8
TRAF3 interacting protein 2	Traf3ip2	NM_134000	-1.2	-1.5

Additional Table 5. Fold Change ≥ 1.5 in either males or females for the comparison MSG vs Control amongst the differentially expressed genes with respect to diet as well as sex (P<0.01).

Gene Names	Gene Symbol	RefSeq	Fold Change MSG / Control (Males)	Fold Change MSG /Control (Females)
phosphodiesterase 3B, cGMP-inhibited	Pde3b	NM_011055	-1.2	-3.2
dolichyl pyrophosphate phosphatase 1	Dolpp1	NM_020329	-1.2	-1.6
pleckstrin homology domain containing, family H (with MyTH4 domain) member 1	Plekhh1	AK122464	-1.3	1.9
LIM-domain containing, protein kinase	Limk1	NM_010717	-1.3	-1.9
pellino 1	Peli1	NM_023324	-1.3	-1.5
cartilage associated protein	Crtap	NM_019922	-1.3	-1.6
chemokine-like receptor 1	Cmklr1	NM_008153	-1.3	-1.5
cysteine-rich with EGF-like domains 2	Creld2	NM_029720	-1.3	-1.7
protein disulfide isomerase associated 4	Pdia4	NM_009787	-1.3	-1.7
inositol polyphosphate 5-phosphatase J	Inpp5j	NM_172439	-1.3	-1.9
dynein, axonemal, light chain 1	Dnalc1	NM_028821	-1.3	1.9
integrin alpha V	Itgav	NM_008402	-1.4	-1.5
acyl-CoA thioesterase 12	Acot12	NM_028790	-1.4	3.5
protein tyrosine phosphatase-like (proline instead of catalytic arginine), member b	Ptplb	NM_023587	-1.4	-2.7
glutaredoxin	Glrx	NM_053108	-1.4	-1.5
family with sequence similarity 78, member B	Fam78b	NM_001160262	-1.4	-1.7
growth arrest-specific 2 like 1	Gas2l1	NM_030228	-1.4	-1.7
protease, serine, 23	Prss23	NM_029614	-1.4	-2.0
calreticulin	Calr	NM_007591	-1.5	-1.3
hairy/enhancer-of-split related with YRPW motif-like	Heyl	NM_013905	-1.5	-1.0
cyclin M3	Cnnm3	NM_053186	-1.5	1.0
zinc finger protein 362	Zfp362	NM_001081098	-1.5	-1.2
platelet-derived growth factor receptor-like	Pdgfrl	NM_026840	-1.5	1.8
THAP domain containing, apoptosis associated protein 2	Thap2	NM_025780	-1.5	-1.4
procollagen C-endopeptidase enhancer protein	Pcolce	NM_008788	-1.5	-1.2
glycerophosphodiester phosphodiesterase domain containing 5	Gdpd5	NM_201352	-1.5	1.3
interferon regulatory factor 2 binding protein 2	Irf2bp2	BC048951	-1.5	-1.2
retinoic acid induced 14	Rai14	NM_030690	-1.5	-1.2
potassium inwardly-rectifying channel, subfamily J, member 12	Kcnj12	NM_010603	-1.5	-1.3
annexin A1	Anxa1	NM_010730	-1.5	-1.4
salt inducible kinase 1	Sik1	NM_010831	-1.5	-1.2
Ras association (RalGDS/AF-6) domain family (N-terminal) member 7	Rassf7	NM_025886	-1.5	1.5
RIKEN cDNA 6330549D23 gene	6330549D23Rik	NR_003619	-1.5	1.4
protein kinase domain containing, cytoplasmic	Pkdcc	NM_134117	-1.5	-1.7
peptidase inhibitor 16	Pi16	NM_023734	-1.5	-1.6
guanylate cyclase activator 1a (retina)	Guca1a	NM_008189	-1.5	1.3
epithelial membrane protein 1	Emp1	NM_010128	-1.5	-1.6
axin 1	Axin1	NM_001159598	-1.6	-1.1
integral membrane protein 2A	Itm2a	NM_008409	-1.6	-1.2
arylsulfatase B	Arsb	NM_009712	-1.6	-1.7
ski sarcoma viral oncogene homolog (avian)	Ski	NM_011385	-1.6	-1.2
phosphodiesterase 6A, cGMP-specific, rod, alpha	Pde6a	NM_146086	-1.6	-1.4
cyclin D3	Ccnd3	NM_007632	-1.6	-1.5
PDZ domain containing 3	Pdzd3	NM_133226	-1.6	1.7
histone cluster 1, H1e	Hist1h1e	NM_015787	-1.6	-1.1

Additional Table 5. Fold Change ≥ 1.5 in either males or females for the comparison MSG vs Control amongst the differentially expressed genes with respect to diet as well as sex (P<0.01).

Gene Names	Gene Symbol	RefSeq	Fold Change MSG / Control (Males)	Fold Change MSG /Control (Females)
mediator of RNA polymerase II transcription, subunit 12 homolog (yeast)-like	Med12l	NM_177855	-1.6	-1.8
tribbles homolog 1 (Drosophila)	Trib1	NM_144549	-1.6	-1.4
IQ motif containing H	Iqch	NM_030068	-1.7	-1.1
myotubularin related protein 12	Mtmr12	NM_172958	-1.7	-1.3
fibulin 1	Fbln1	NM_010180	-1.7	-1.6
coactosin-like 1 (Dictyostelium)	Cotl1	NM_028071	-1.7	-2.6
insulin-like growth factor binding protein 3	Igfbp3	NM_008343	-1.7	-1.3
folate receptor 2 (fetal)	Folr2	NM_008035	-1.7	-1.7
UDP-N-acetyl-alpha-D-galactosamine:polypeptide N-acetylgalactosaminyltransferase 10	Galnt10	NM_134189	-1.7	-1.0
cryptochrome 1 (photolyase-like)	Cry1	NM_007771	-1.8	-3.7
unc-5 homolog C (C. elegans)-like	Unc5cl	NM_152823	-1.8	-1.0
BMP and activin membrane-bound inhibitor, homolog (Xenopus laevis)	Bambi	NM_026505	-1.8	-1.3
HEAT repeat containing 6	Heatr6	NM_145432	-1.8	-1.0
abhydrolase domain containing 12B	Abhd12b	ENSMUST00000095662	-1.8	1.0
NLR family, apoptosis inhibitory protein 1	Naip1	NM_008670	-1.9	-2.1
sterile alpha motif domain containing 4B	Samd4b	NM_175021	-1.9	-1.0
ADAMTS-like 3	Adamtsl3	XM_984557	-1.9	-1.7
histocompatibility 2, T region locus 10	H2-T10	NM_010395	-1.9	1.2
kelch-like 3 (Drosophila)	Klhl3	ENSMUST00000091583	-2.0	1.3
collagen, type III, alpha 1	Col3a1	NM_009930	-2.0	-1.5
midkine	Mdk	NM_010784	-2.0	-2.2
transmembrane protease, serine 11f	Tmprss11f	NM_178730	-2.1	3.5
tubulin polymerization-promoting protein family member 3	Tppp3	NM_026481	-2.5	-1.9
pleiomorphic adenoma gene-like 1	Plagl1	NM_009538	-2.5	-1.7
hyperpolarization-activated, cyclic nucleotide-gated K+ 1	Hcn1	NM_010408	-2.6	-6.3
carbonic anhydrase 5b, mitochondrial	Car5b	NM_181315	-2.7	-9.0
Unknown	AF362573	AF362573	-2.9	-1.0
similar to Unknown (protein for IMAGE:4910858)	ND6	ENSMUST00000082419	-4.3	1.4
