# Supplementary material for: Sex-dimorphism in Cardiac Nutrigenomics: effect of Trans fat and/or Monosodium Glutamate consumption
Source: BMC Genomics. 2011 Nov 12;12:555. doi: 10.1186/1471-2164-12-555 (PMC3238303; doi:10.1186/1471-2164-12-555)
Supplement: Additional file 6 — Table S6. Gene ontologies enriched for differentially expressed genes comparing MSG to Control diet in males and females. [file 1471-2164-12-555-S6.PDF]

Additional Table 6. Gene ontologies enriched for differentially expressed genes comparing MSG to Control diet in males and females.

Term ID	Category	No. of genes	*P* Value
**Ratio ≥1.5 (upregulated in MSG males vs Control)**			
**Biological Process**			
GO:0009987	**cellular process**	61	0.006
GO:0008152	**metabolic process**	49	0.01
GO:0034641	**cellular nitrogen compound metabolic process**	30	<.001
GO:0006139	**nucleobase, nucleoside, nucleotide and nucleic acid metabolic process**	25	0.01
GO:0009058	**biosynthetic process**	25	0.02
GO:0044249	**cellular biosynthetic process**	24	0.03
GO:0032502	**developmental process**	23	0.02
GO:0007275	**multicellular organismal development**	21	0.03
GO:0045449	**regulation of transcription**	19	0.04
GO:0051716	**cellular response to stimulus**	10	0.002
GO:0051726	**regulation of cell cycle**	5	0.02
GO:0051270	**regulation of cell motion**	4	0.01
**Cellular Component**			
GO:0005634	**nucleus**	31	0.01
**Molecular Function**			
GO:0005488	**binding**	70	0.01
GO:0043167	**ion binding**	38	<.0001
GO:0043169	**Select calcium binding protein**	38	<.0001
GO:0046872	**metal ion binding**	38	<.0001
**Ratio < - 1.5 (downregulated in MSG Males vs Control)**			
**Biological Process**			
GO:0006796	**phosphate metabolic process**	7	0.03
GO:0016310	**phosphorylation**	6	0.05
**Molecular Function**			
GO:0030234	**enzyme regulator activity**	8	0.003
GO:0030249	**guanylate cyclase regulator activity**	2	0.008
GO:0005515	**protein binding**	23	0.04
MF00188	**Select calcium binding protein**	4	0.03

Additional Table 6. Gene ontologies enriched for differentially expressed genes comparing MSG to Control diet in males and females.

Term ID	Category	No. of genes	*P* Value
**Ratio ≥1.5 (upregulated in MSG females vs Control)**			
**Biological Process**			
GO:0032502	**developmental process**	26	0.002
GO:0032879	**regulation of localization**	8	0.005
GO:0043065	**positive regulation of apoptosis**	6	0.009
GO:0006816	**calcium ion transport**	4	0.02
GO:0006909	**phagocytosis**	3	0.02
GO:0048856	**anatomical structure development**	19	0.02
GO:0009308	**amine metabolic process**	6	0.03
GO:0006811	**ion transport**	9	0.03
GO:0019752	**carboxylic acid metabolic process**	7	0.03
GO:0048513	**organ development**	15	0.04
**Cellular Component**			
GO:0031226	**intrinsic to plasma membrane**	8	0.02
GO:0045177	**apical part of cell**	4	0.03
**Molecular Function**			
GO:0004857	**enzyme inhibitor activity**	7	0.002
GO:0005488	**binding**	75	0.003
GO:0030234	**enzyme regulator activity**	11	0.006
GO:0016841	**ammonia-lyase activity**	2	0.02
GO:0000287	**magnesium ion binding**	7	0.02
GO:0043167	**ion binding**	31	0.02
GO:0048037	**cofactor binding**	5	0.03
GO:0016840	**carbon-nitrogen lyase activity**	2	0.04
GO:0005262	**calcium channel activity**	3	0.05
**Ratio < - 1.5 (downregulated in MSG females vs Control)**			
**Cellular Component**			
GO:0031012	**extracellular matrix**	6	0.001
GO:0005783	**endoplasmic reticulum**	9	0.002
GO:0005576	**extracellular region**	12	0.007
**Molecular Function**			
MF00180	**Extracellular matrix glycoprotein**	4	0.002
MF00178	**Extracellular matrix**	6	0.003
