# Supplementary material for: Sex-dimorphism in Cardiac Nutrigenomics: effect of Trans fat and/or Monosodium Glutamate consumption
Source: BMC Genomics. 2011 Nov 12;12:555. doi: 10.1186/1471-2164-12-555 (PMC3238303; doi:10.1186/1471-2164-12-555)
Supplement: Additional file 7 — Table S7. Differentially expressed genes in either males or females for the comparison TFA+MSG vs TFA with respect to diet and sex (P < 0.01) and fold Change ≥ ± 1.5. [file 1471-2164-12-555-S7.PDF]

Additional Table 7. Fold Change ≥ 1.5 in either males or Females for the comparison TFA+MSG vs TFA amongst the differentially expressed genes with respect to diet as well as sex (P≤0.01).

Gene Names	Gene Symbol	RefSeq	Fold Change TFA+MSG/TFA (Males)	Fold Change TFA+MSG/TFA (Females)
heat shock transcription factor 4	Hsf4	NM_011939	5.4	3.1
sodium channel, voltage-gated, type IX, alpha	Scn9a	NM_018852	4.2	4.0
T-cell acute lymphocytic leukemia 2	Tal2	NM_009317	2.9	8.2
coactosin-like 1 (Dictyostelium)	Cotl1	NM_028071	2.7	1.3
branched chain aminotransferase 1, cytosolic	Bcat1	NM_001024468	2.5	2.1
NLR family, apoptosis inhibitory protein 1	Naip1	NM_008670	2.4	-1.2
uridine-cytidine kinase 2	Uck2	NM_030724	2.4	1.4
actin-like 6B	Actl6b	NM_031404	2.3	1.6
histocompatibility 2, M region locus 10.3	H2-M10.3	NM_201608	2.3	4.9
homeo box C10	Hoxc10	NM_010462	2.3	2.8
sterile alpha motif domain containing 4B	Samd4b	NM_175021	2.2	1.5
sterol regulatory element binding factor 2	Srebf2	NM_033218	2.2	2.4
Ras association (RalGDS/AF-6) domain family (N-terminal) member 7	Rassf7	NM_025886	2.1	1.4
family with sequence similarity 125, member B	Fam125b	BC049129	2.1	1.2
solute carrier family 41, member 1	Slc41a1	NM_173865	2.1	1.3
acyl-CoA thioesterase 12	Acot12	NM_028790	2.0	-3.2
BEN domain containing 3	Bend3	NM_199028	2.0	1.0
REX1, RNA exonuclease 1 homolog (S. cerevisiae)	Rexo1	NM_025852	2.0	-1.1
proteasome (prosome, macropain) 26S subunit, ATPase 2	Psmc2	NM_011188	2.0	2.4
TSC22 domain family, member 4	Tsc22d4	NM_023910	1.9	1.4
dual-specificity tyrosine-(Y)-phosphorylation regulated kinase 1b	Dyrk1b	NM_001037957	1.9	1.1
arylsulfatase B	Arsb	NM_009712	1.9	-1.0
PH domain and leucine rich repeat protein phosphatase 2	Phlpp2	NM_001122594	1.9	1.7
WW domain binding protein 2	Wbp2	NM_016852	1.9	1.1
tripartite motif-containing 3	Trim3	NM_018880	1.9	1.3
vesicle-associated membrane protein 2	Vamp2	NM_009497	1.9	1.2
nuclear receptor subfamily 1, group H, member 2	Nr1h2	NM_009473	1.9	1.2
forkhead box P4	Foxp4	NM_001110824	1.9	1.7
G protein-coupled receptor 85	Gpr85	NM_145066	1.9	-1.2
transcription factor 3	Tcf3	NM_001079822	1.9	1.3
integrin alpha FG-GAP repeat containing 2	Itfg2	NM_133927	1.9	1.4
AKT1 substrate 1 (proline-rich)	Akt1s1	NM_026270	1.9	1.2
predicted gene 88	Gm88	BC147714	1.8	1.5
suppressor of Ty 3 homolog (S. cerevisiae)	Supt3h	NM_178652	1.8	-1.1
v-rel reticuloendotheliosis viral oncogene homolog A (avian)	Rela	NM_009045	1.8	1.3
period homolog 1 (Drosophila)	Per1	NM_011065	1.8	1.1
midnolin	Midn	NM_021565	1.8	-1.1
calcium/calmodulin-dependent protein kinase kinase 1, alpha	Camkk1	NM_018883	1.8	2.0
synaptopodin 2-like	Synpo2l	NM_175132	1.8	1.1
GH3 domain containing	Ghdc	NM_031871	1.8	1.3
predicted gene 12824	Gm12824	NM_001085549	1.8	-1.3
polymerase (DNA-directed), delta interacting protein 2	Poldip2	NM_026389	1.8	1.1
proteasome (prosome, macropain) 26S subunit, non-ATPase, 9	Psmd9	NM_026000	1.7	1.0
RGP1 retrograde golgi transport homolog (S. cerevisiae)	Rgp1	NM_172866	1.7	-1.0
CDC42 effector protein (Rho GTPase binding) 1	Cdc42ep1	NM_027219	1.7	-1.0
ankyrin repeat domain 34A	Ankrd34a	NM_001024851	1.7	1.4
HLA-B associated transcript 2	Bat2	NM_020027	1.7	1.2
transmembrane protein 150	Tmem150	NM_144916	1.7	1.0

Additional Table 7. Fold Change ≥ 1.5 in either males or Females for the comparison TFA+MSG vs TFA amongst the differentially expressed genes with respect to diet as well as sex (P≤0.01).

Gene Names	Gene Symbol	RefSeq	Fold Change TFA+MSG/TFA (Males)	Fold Change TFA+MSG/TFA (Females)
male enhanced antigen 1	Mea1	NM_010787	1.7	1.3
zinc finger, DHHC domain containing 18	Zdhhc18	NM_001017968	1.7	-1.1
LIM-domain containing, protein kinase	Limk1	NM_010717	1.7	2.1
LIM domain only 4	Lmo4	NM_010723	1.7	1.2
keratin 13	Krt13	NM_010662	1.6	2.2
mediator of DNA damage checkpoint 1	Mdc1	NM_001010833	1.6	1.9
zinc finger CCCH-type, antiviral 1-like	Zc3hav1l	NM_172467	1.6	1.8
cAMP responsive element binding protein 5	Creb5	ENSMUST00000114409	1.6	1.7
guanine nucleotide binding protein, alpha transducing 2	Gnat2	NM_008141	1.6	1.3
sodium channel, voltage-gated, type V, alpha	Scn5a	NM_021544	1.6	1.2
sphingosine-1-phosphate phosphatase 1	Sgpp1	NM_030750	1.6	1.2
transmembrane protein 53	Tmem53	NM_026837	1.6	1.2
predicted gene 5127	Gm5127	NM_001033541	1.6	1.7
tripartite motif-containing 11	Trim11	NM_053168	1.6	1.1
PHD finger protein 2	Phf2	NM_011078	1.6	-1.0
proviral integration site 3	Pim3	NM_145478	1.6	-1.0
RGM domain family, member A	Rgma	NM_177740	1.6	1.1
zinc finger protein 42	Zfp42	NM_009556	1.6	2.2
sprouty homolog 1 (Drosophila)	Spry1	NM_011896	1.6	1.1
splicing factor 1	Sf1	NM_001110791	1.6	1.1
heat shock factor 1	Hsf1	NM_008296	1.6	-1.1
HD domain containing 2	Hddc2	NM_027168	1.6	2.0
ST6 (alpha-N-acetyl-neuraminyl-2,3-beta-galactosyl-1,3)-N-acetylgalactosaminide alpha-2,6-sialyltransferase 4	St6galnac4	NM_011373	1.6	1.2
KDEL (Lys-Asp-Glu-Leu) endoplasmic reticulum protein retention receptor 1	Kdelr1	NM_133950	1.6	1.2
zinc finger protein 362	Zfp362	NM_001081098	1.6	1.1
RIKEN cDNA C230052I12 gene	C230052I12Rik	NM_178643	1.6	4.0
fibrosin	Fbrs	NM_010183	1.6	1.4
carbonic anhydrase 5b, mitochondrial	Car5b	NM_181315	1.6	1.2
lysyl oxidase-like 1	Loxl1	NM_010729	1.6	1.1
heparan sulfate 6-O-sulfotransferase 1	Hs6st1	NM_015818	1.6	-1.0
G protein-coupled receptor 146	Gpr146	NM_030258	1.5	1.2
NF-kappaB repressing factor	Nkrf	NM_029891	1.5	-1.1
epsin 3	Epn3	NM_027984	1.5	1.0
TAF12 RNA polymerase II, TATA box binding protein (TBP)-associated factor	Taf12	NM_025579	1.5	1.3
ATPase, Cu++ transporting, beta polypeptide	Atp7b	NM_007511	1.5	-3.0
hexokinase 1	Hk1	NM_001146100	1.5	1.3
C1q and tumor necrosis factor related protein 1	C1qtnf1	NM_019959	1.5	-1.0
stathmin 1	Stmn1	NM_019641	1.5	1.3
tribbles homolog 1 (Drosophila)	Trib1	NM_144549	1.5	1.0
transmembrane protein 106A	Tmem106a	NM_144830	1.5	1.1
proline rich 7 (synaptic)	Prr7	NM_001030296	1.5	1.0
COP9 (constitutive photomorphogenic) homolog, subunit 8 (Arabidopsis thaliana)	Cops8	NM_133805	1.5	1.6
GATS protein-like 2	Gatsl2	NM_030719	1.5	1.2
nudix (nucleoside diphosphate linked moiety X)-type motif 17	Nudt17	NM_030094	1.5	-1.4
microtubule-associated protein 6	Mtap6	NM_010837	1.5	1.2
zinc finger protein 295	Zfp295	NM_175428	1.5	1.4
SR-related CTD-associated factor 1	Scaf1	NM_001008422	1.5	-1.1

Additional Table 7. Fold Change ≥ 1.5 in either males or Females for the comparison TFA+MSG vs TFA amongst the differentially expressed genes with respect to diet as well as sex (P≤0.01).

Gene Names	Gene Symbol	RefSeq	Fold Change TFA+MSG/TFA (Males)	Fold Change TFA+MSG/TFA (Females)
GATA binding protein 4	Gata4	NM_008092	1.5	1.2
leucine rich repeat containing 61	Lrrc61	NM_177736	1.5	1.4
septin 14;RIKEN cDNA 1700017B05 gene	1700017B05Rik	NM_028820	1.5	1.3
serine/threonine kinase 24 (STE20 homolog, yeast)	Stk24	NM_145465	1.5	1.0
homeo box B9	Hoxb9	NM_008270	1.5	2.0
asparagine-linked glycosylation 10 homolog B (yeast, alpha-1,2-glucosyltransferase)	Alg10b	NM_001033441	1.5	1.2
UDP-N-acetyl-alpha-D-galactosamine:polypeptide N-acetylgalactosaminyltransferase 10	Galnt10	NM_134189	1.5	1.1
RIKEN cDNA 2310022B05 gene	2310022B05Rik	BC058626	1.5	1.0
Sp2 transcription factor	Sp2	NM_030220	1.5	1.1
glucosamine-phosphate N-acetyltransferase 1	Gnpnat1	NM_019425	1.5	1.6
bromodomain containing 4	Brd4	NM_020508	1.5	1.1
progestin and adipoQ receptor family member IX	Paqr9	NM_198414	1.5	1.1
RAS, guanyl releasing protein 2	Rasgrp2	NM_011242	1.5	-1.2
zinc finger protein 239	Zfp239	NM_001001792	1.5	1.2
solute carrier family 7 (cationic amino acid transporter, y+ system), member 1	Slc7a1	NM_007513	1.5	1.2
peroxiredoxin 6	Prdx6	NM_007453	1.5	1.0
guanine nucleotide binding protein (G protein), beta 2	Gnb2	NM_010312	1.5	1.1
mitogen-activated protein kinase kinase kinase kinase 2	Map4k2	NM_009006	1.5	1.4
DCN1, defective in cullin neddylation 1, domain containing 4 (S. cerevisiae)	Dcun1d4	NM_178896	1.5	1.4
GRAM domain containing 4	Gramd4	NM_172611	1.5	1.2
myosin VIIA	Myo7a	NM_008663	1.4	2.0
acetyl-Coenzyme A acetyltransferase 3	Acat3	NM_153151	1.4	2.2
inositol polyphosphate 5-phosphatase J	Inpp5j	NM_172439	1.4	1.5
ATPase, class V, type 10B	Atp10b	ENSMUST00000056678	1.4	1.5
dual specificity phosphatase 5	Dusp5	NM_001085390	1.4	2.4
synaptosomal-associated protein 29	Snap29	NM_023348	1.3	1.6
leucine rich repeat containing 3B	Lrrc3b	NM_146052	1.3	1.8
RIKEN cDNA F630111L10 gene	F630111L10Rik	AK170843	1.3	-1.8
G0/G1 switch gene 2	G0s2	NM_008059	1.3	-1.5
folate receptor 2 (fetal)	Folr2	NM_008035	1.3	1.6
nuclear transcription factor-Y alpha	Nfya	NM_001110832	1.3	1.7
bridging integrator 3	Bin3	NM_021328	1.3	1.5
RIKEN cDNA E130309F12 gene	E130309F12Rik	NM_178756	1.2	1.6
potassium voltage-gated channel, subfamily H (eag-related), member 1	Kcnh1	NM_010600	1.2	1.5
proteoglycan 4 (megakaryocyte stimulating factor, articular superficial zone protein)	Prg4	NM_021400	1.2	2.3
protein kinase domain containing, cytoplasmic	Pkdcc	NM_134117	1.2	1.5
zinc finger and BTB domain containing 7C	Zbtb7c	NM_145356	1.2	1.8
KDEL (Lys-Asp-Glu-Leu) endoplasmic reticulum protein retention receptor 3	Kdelr3	NM_134090	1.2	1.6
neuronal pentraxin 1	Nptx1	NM_008730	1.2	1.6
pancreatic lipase	Pnlip	NM_026925	1.2	3.7
RIKEN cDNA I830127L07 gene	I830127L07Rik	XM_909906	1.2	2.0
copine II	Cpne2	NM_153507	1.1	1.6
patatin-like phospholipase domain containing 3	Pnpla3	NM_054088	1.1	1.9
guanylate cyclase activator 1a (retina)	Guca1a	NM_008189	1.1	-1.6
collagen, type III, alpha 1	Col3a1	NM_009930	1.1	1.5

Additional Table 7. Fold Change ≥ 1.5 in either males or Females for the comparison TFA+MSG vs TFA amongst the differentially expressed genes with respect to diet as well as sex (P≤0.01).

Gene Names	Gene Symbol	RefSeq	Fold Change TFA+MSG/TFA (Males)	Fold Change TFA+MSG/TFA (Females)
IQ motif containing H	Iqch	NM_030068	1.1	-1.5
THAP domain containing, apoptosis associated protein 2	Thap2	NM_025780	1.1	1.8
aryl-hydrocarbon receptor	Ahr	NM_013464	1.1	-1.6
mediator of RNA polymerase II transcription, subunit 12 homolog (yeast)-like	Med12l	NM_177855	1.1	1.6
Bmi1 polycomb ring finger oncogene	Bmi1	NM_007552	1.1	1.6
RIKEN cDNA 2610018G03 gene	2610018G03Rik	NM_133729	1.0	-1.8
Unknown	Gm9568	XR_032493	1.0	1.8
spondin 2, extracellular matrix protein	Spon2	NM_133903	1.0	1.7
pleckstrin homology domain containing, family H (with MyTH4 domain) member 1	Plekhh1	AK122464	1.0	-2.2
centrosomal protein 76	Cep76	NM_001081073	1.0	1.6
heme binding protein 1	Hebp1	NM_013546	1.0	-1.5
axin 1	Axin1	NM_001159598	-1.0	-1.6
predicted gene 11435	Gm11435	NM_001045543	-1.0	-1.8
tryptophan hydroxylase 1	Tph1	NM_009414	-1.0	-1.6
threonine synthase-like 2 (bacterial)	Thnsl2	NM_178413	-1.1	1.7
transmembrane protein 56	Tmem56	NM_178936	-1.1	-1.7
serine dehydratase	Sds	NM_145565	-1.2	-2.0
membrane-spanning 4-domains, subfamily A, member 2	Ms4a2	NM_013516	-1.2	-2.9
nudix (nucleoside diphosphate linked moiety X)-type motif 2	Nudt2	NM_025539	-1.2	-1.7
methyl-CpG binding domain protein 4	Mbd4	NM_010774	-1.2	-2.1
zinc finger protein 292	Zfp292	NM_013889	-1.3	-1.5
RIKEN cDNA 4930539E08 gene	4930539E08Rik	BC117930	-1.3	-1.6
glucose-fructose oxidoreductase domain containing 2	Gfod2	NM_027469	-1.3	-2.0
per-hexamer repeat gene 4	Phxr4	X12806	-1.3	-1.5
phospholipase A2, group XV	Pla2g15	NM_133792	-1.3	-2.0
zinc finger protein 819	Zfp819	NM_028913	-1.3	-1.5
signal transducer and activator of transcription 2	Stat2	NM_019963	-1.3	-1.7
phosphoserine aminotransferase 1	Psat1	NM_177420	-1.3	-1.8
zinc finger protein 13	Zfp13	NM_011747	-1.3	-1.9
interleukin 18 receptor accessory protein	Il18rap	NM_010553	-1.3	-1.8
transient receptor potential cation channel, subfamily V, member 5	Trpv5	NM_001007572	-1.3	-1.7
interferon inducible GTPase 1	Iigp1	NM_021792	-1.3	1.6
leucine rich repeat containing 26	Lrrc26	NM_146117	-1.4	-2.2
G protein-coupled receptor kinase 4	Grk4	NM_019497	-1.4	-2.0
a disintegrin and metallopeptidase domain 5	Adam5	NM_007401	-1.4	-1.9
RIKEN cDNA 2010109I03 gene	2010109I03Rik	NM_025929	-1.4	-1.9
serine/threonine kinase 36 (fused homolog, Drosophila)	Stk36	NM_175031	-1.4	-1.8
a disintegrin-like and metallopeptidase (reprolysin type) with thrombospondin type 1 motif, 7	Adamts7	NM_001003911	-1.4	-1.7
phosphodiesterase 6C, cGMP specific, cone, alpha prime	Pde6c	NM_033614	-1.4	-1.7
vacuolar protein sorting 13A (yeast)	Vps13a	NM_173028	-1.5	-1.4
engulfment and cell motility 3, ced-12 homolog (C. elegans)	Elmo3	NM_172760	-1.5	-1.2
5-methyltetrahydrofolate-homocysteine methyltransferase	Mtr	NM_001081128	-1.5	1.4
galectin-related inter-fiber protein	Grifin	NM_030022	-1.5	-1.7
predicted gene 7278	Gm7278	XR_034437	-1.5	-1.5
general transcription factor II H, polypeptide 2	Gtf2h2	NM_022011	-1.5	1.0
homer homolog 1 (Drosophila)	Homer1	NM_152134	-1.5	1.0
CUB domain containing protein 2	Cdcp2	NM_172873	-1.5	-1.3

Additional Table 7. Fold Change ≥ 1.5 in either males or Females for the comparison TFA+MSG vs TFA amongst the differentially expressed genes with respect to diet as well as sex (P≤0.01).

Gene Names	Gene Symbol	RefSeq	Fold Change TFA+MSG/TFA (Males)	Fold Change TFA+MSG/TFA (Females)
BR serine/threonine kinase 2	Brsk2	NM_001009930	-1.5	-1.6
olfactory receptor 146	Olfr146	NM_146747	-1.5	-1.4
progestin and adipoQ receptor family member V	Paqr5	NM_028748	-1.5	-4.5
histone cluster 1, H1e	Hist1h1e	NM_015787	-1.5	-1.3
inositol polyphosphate-4-phosphatase, type I	Inpp4a	NM_030266	-1.5	-1.2
WAP four-disulfide core domain 5	Wfdc5	NM_145369	-1.5	-1.6
Meis homeobox 2	Meis2	NM_001136072	-1.5	-1.1
Unknown	BC063263	BC063263	-1.5	1.1
inter-alpha trypsin inhibitor, heavy chain 3	Itih3	NM_008407	-1.5	1.5
secretory leukocyte peptidase inhibitor	Slpi	NM_011414	-1.5	-2.0
NIMA (never in mitosis gene a)-related expressed kinase 11	Nek11	NM_172461	-1.6	-1.3
cyclin-dependent kinase inhibitor 2A	Cdkn2a	NM_009877	-1.6	-2.1
forkhead box B2	Foxb2	NM_008023	-1.6	-1.7
NEDD4 binding protein 2	N4bp2	NM_001024917	-1.6	-1.2
dystrophin, muscular dystrophy	Dmd	NM_007868	-1.6	-1.0
DAZ interacting protein 1	Dzip1	NM_025943	-1.6	-1.2
RNA binding motif protein 28	Rbm28	NM_133925	-1.6	-1.4
zinc finger protein 97	Zfp97	NM_011765	-1.6	-1.0
RIKEN cDNA 4921501E09 gene	4921501E09Rik	NM_001009544	-1.6	-1.0
RNA binding motif protein 4	Rbm4	NM_009032	-1.6	-1.2
enhancer of zeste homolog 1 (Drosophila)	Ezh1	NM_007970	-1.6	1.1
cation channel, sperm associated 1	Catsper1	NM_139301	-1.6	-2.1
solute carrier family 6 (neurotransmitter transporter, noradrenalin), member 2	Slc6a2	NM_009209	-1.7	1.6
RIKEN cDNA 4933408B17 gene	4933408B17Rik	NM_177773	-1.7	1.3
histone deacetylase 2	Hdac2	NM_008229	-1.7	-1.0
phosphodiesterase 6A, cGMP-specific, rod, alpha	Pde6a	NM_146086	-1.7	1.2
cytotoxic and regulatory T cell molecule	Crtam	NM_019465	-1.7	1.0
glutamate receptor, ionotropic, kainate 3	Grik3	NM_001081097	-1.7	-1.5
coiled-coil domain containing 138	Ccdc138	NM_001162956	-1.7	-1.6
tumor necrosis factor (ligand) superfamily, member 14	Tnfsf14	NM_019418	-1.8	1.3
RIKEN cDNA A830039N20 gene	A830039N20Rik	BC038501	-1.8	-1.4
limb expression 1 homolog (chicken)	Lix1	NM_025681	-1.8	1.5
apoptosis-inducing factor, mitochondrion-associated 3	Aifm3	NM_175178	-1.8	-1.9
serine (or cysteine) peptidase inhibitor, clade B, member 5	Serpinb5	NM_009257	-1.9	-4.4
Unknown	Gm5188	ENSMUST00000037962	-1.9	-2.4
solute carrier family 13 (sodium-dependent dicarboxylate transporter), member 2	Slc13a2	NM_022411	-1.9	-1.8
tyrosine aminotransferase	Tat	NM_146214	-1.9	-2.2
cholinergic receptor, nicotinic, beta polypeptide 3	Chrnb3	NM_173212	-1.9	-4.8
dynein, axonemal, light chain 1	Dnalc1	NM_028821	-1.9	-1.6
leucine rich repeat containing 46	Lrrc46	NM_027026	-1.9	-2.1
calcyphosphine 2	Caps2	NM_178278	-2.0	-2.4
ral guanine nucleotide dissociation stimulator-like 3	Rgl3	NM_023622	-2.0	-1.9
plastin 3 (T-isoform)	Pls3	NM_145629	-2.0	1.2
DNA-damage-inducible transcript 4-like	Ddit4l	NM_030143	-2.0	1.2
coiled-coil domain containing 60	Ccdc60	ENSMUST00000086483	-2.1	-1.2
sclerostin	Sost	NM_024449	-2.1	-1.7
WD repeat and FYVE domain containing 2	Wdfy2	NM_175546	-2.1	-1.7
predicted gene 13089	Gm13089	ENSMUST00000073532	-2.1	-3.7

Additional Table 7. Fold Change ≥ 1.5 in either males or Females for the comparison TFA+MSG vs TFA amongst the differentially expressed genes with respect to diet as well as sex (P≤0.01).

Gene Names	Gene Symbol	RefSeq	Fold Change TFA+MSG/TFA (Males)	Fold Change TFA+MSG/TFA (Females)
small nuclear ribonucleoprotein 35 (U11/U12)	Snrnp35	NM_029532	-2.1	-2.0
platelet-derived growth factor receptor-like	Pdgfrl	NM_026840	-2.1	-2.4
topoisomerase (DNA) II binding protein 1	Topbp1	NM_176979	-2.2	-1.4
polymerase (DNA directed), iota	Poli	NM_011972	-2.2	-2.3
dopey family member 1	Dopey1	NM_177208	-2.3	-1.6
calcium/calmodulin-dependent protein kinase II, beta	Camk2b	BC080273	-2.3	-2.1
solute carrier family 45, member 2	Slc45a2	NM_053077	-2.5	1.3
RIKEN cDNA 2900083I11 gene	2900083I11Rik	NM_021403	-2.5	-1.9
tachykinin receptor 3	Tacr3	NM_021382	-2.5	-1.4
RIKEN cDNA 2810021B07 gene	2810021B07Rik	NM_025479	-2.7	-1.7
coiled-coil domain containing 39	Ccdc39	NM_026222	-2.8	-4.1
RIKEN cDNA 1700041C02 gene	Ccdc30	NM_029286	-2.8	-2.5
RIKEN cDNA 6720416L17 gene	6720416L17Rik	ENSMUST00000100000	-2.8	-2.8
aldehyde dehydrogenase family 1, subfamily A3	Aldh1a3	NM_053080	-2.9	-2.3
PDZ domain containing 3	Pdzd3	NM_133226	-3.1	-2.3
melanoma associated antigen (mutated) 1-like 1	Mum1l1	ENSMUST00000113045	-3.1	-3.3
transmembrane protein 190	Tmem190	ENSMUST00000013235	-3.1	-4.1
formiminotransferase cyclodeaminase	Ftcd	NM_080845	-3.2	-2.7
mitogen-activated protein kinase kinase kinase 15	Map3k15	ENSMUST00000033665	-3.5	-1.3
similar to Unknown (protein for IMAGE:4910858)	ND6	ENSMUST00000082419	-3.6	-2.2
transmembrane protease, serine 11f	Tmprss11f	NM_178730	-3.8	-4.1
neuropeptide FF receptor 2	Npffr2	NM_133192	-4.2	-2.2
cDNA sequence BC027072	BC027072	BC046516	-4.7	-4.2
dihydropyrimidinase	Dpys	NM_022722	-4.8	-2.7
fibrillin 2	Fbn2	NM_010181	-6.5	-2.9
