# Supplementary material for: Sex-dimorphism in Cardiac Nutrigenomics: effect of Trans fat and/or Monosodium Glutamate consumption
Source: BMC Genomics. 2011 Nov 12;12:555. doi: 10.1186/1471-2164-12-555 (PMC3238303; doi:10.1186/1471-2164-12-555)
Supplement: Additional file 8 — Table S8. Gene ontologies enriched for differentially expressed genes comparing TFA+MSG to TFA diet in males and females. [file 1471-2164-12-555-S8.PDF]

Additional Table 8. Gene ontologies enriched for differentially expressed genes comparing TFA+MSG to TFA diet in males and females.

	Term ID	Category	No. of genes	*P* Value
**Ratio > 1.5 (upregulated in TFA+ MSG males vs TFA)**				
**Biological Process**				
	GO:0009987	**cellular process**	61	0.02
	GO:0008152	**metabolic process**	51	0.008
	GO:0006807	**nitrogen compound metabolic process**	30	0.002
	GO:0009058	**biosynthetic process**	30	0.001
	GO:0019222	**regulation of metabolic process**	27	0.007
	GO:0006350	**transcription**	24	<.0001
	GO:0043687	**post-translational protein modification**	12	0.03
	GO:0032774	**RNA biosynthetic process**	5	0.005
	GO:0006468	**protein amino acid phosphorylation**	9	0.02
	GO:0032583	**regulation of gene-specific transcription**	3	0.05
	BP00031	**Nucleoside, nucleotide and nucleic acid metabolism**	28	0.004
	BP00040	**mRNA transcription**	22	<.001
**Cellular Component**				
	GO:0005576	**extracellular region**	11	0.05
	GO:0005622	**intracellular**	77	0.001
	GO:0019898	**extrinsic to membrane**	10	0.002
**Molecular Function**				
	GO:0005488	**binding**	78	0.007
	GO:0046914	**transition metal ion binding**	25	0.01
	GO:0003677	**DNA binding**	21	0.002
	GO:0008270	**zinc ion binding**	21	0.01
	GO:0016740	**transferase activity**	17	0.04
	GO:0030528	**transcription regulator activity**	16	0.004
**Pathway**				
	P00040	**Metabotropic glutamate receptor group II pathway**	3	0.04
**Ratio ≤ -1.5 (downregulated in TFA+ MSG males vs TFA)**				
**Biological Process**				
	GO:0032502	**developmental process**	17	0.03
	GO:0009308	**amine metabolic process**	5	0.03
	GO:0051049	**regulation of transport**	5	0.02
	GO:0022898	**regulation of transmembrane transporter activity**	2	0.04
**Molecular Function**				
	GO:0016740	**transferase activity**	12	0.04
	GO:0004857	**enzyme inhibitor activity**	7	<.001
	GO:0004866	**endopeptidase inhibitor activity**	4	0.02
	GO:0030594	**neurotransmitter receptor activity**	3	0.03
	MF00016	**Signaling molecule**	8	0.01
	MF00250	**Serine protease inhibitor**	4	0.007

Additional Table 8. Gene ontologies enriched for differentially expressed genes comparing TFA+MSG to TFA diet in males and females.

Term ID	Category	No. of genes	*P* Value
**Ratio >1.5 (upregulated in TFA+ MSG females vs TFA)**			
**Biological Process**			
GO:0008152	**metabolic process**	29	0.02
GO:0006807	**nitrogen compound metabolic process**	19	0.003
GO:0080090	**regulation of primary metabolic process**	14	0.04
GO:0045449	**regulation of transcription**	13	0.02
GO:0009791	**post-embryonic development**	3	0.02
GO:0048265	**response to pain**	2	0.05
BP00040	**mRNA transcription**	11	0.04
**Cellular Compartment**			
GO:0005634	**nucleus**	20	0.042
**Molecular Function**			
GO:0030528	**transcription regulator activity**	10	0.016
GO:0005516	**calmodulin binding**	3	0.05
GO:0004806	**triacylglycerol lipase activity**	2	0.04
MF00139	**Acyltransferase**	3	0.03
**Ratio ≤ -1.5 (downregulated in TFA+ MSG females vs TFA)**			
**Biological Process**			
GO:0007275	**multicellular organismal development**	15	0.05
GO:0006082	**organic acid metabolic process**	8	0.001
GO:0042180	**cellular ketone metabolic process**	8	0.001
GO:0006811	**ion transport**	7	0.04
GO:0009308	**amine metabolic process**	6	0.006
GO:0042981	**regulation of apoptosis**	6	0.04
BP00102	**Signal transduction**	21	0.02
BP00013	**Amino acid metabolism**	5	0.006
**Molecular function**			
GO:0004857	**enzyme inhibitor activity**	5	0.01
GO:0048037	**cofactor binding**	5	0.01
GO:0019842	**vitamin binding**	4	0.01
GO:0016597	**amino acid binding**	3	0.008
GO:0070279	**vitamin B6 binding**	3	0.02
GO:0010851	**cyclase regulator activity**	2	0.01
GO:0016841	**ammonia-lyase activity**	2	0.01
GO:0030249	**guanylate cyclase regulator activity**	2	0.01
MF00141	**Hydrolase**	7	0.04
MF00108	**Protein kinase**	6	0.04
