# Supplementary material for: Sex-dimorphism in Cardiac Nutrigenomics: effect of Trans fat and/or Monosodium Glutamate consumption
Source: BMC Genomics. 2011 Nov 12;12:555. doi: 10.1186/1471-2164-12-555 (PMC3238303; doi:10.1186/1471-2164-12-555)
Supplement: Additional file 9 — Table S9. Intensities of genes/ESTs differentially regulated only in males with a fold change of ≥ ± 1.5 for either of the comparisons Control vs MSG, Control vs TFA, or TFA vs TFA+MSG. [file 1471-2164-12-555-S9.PDF]

Additional Table 9. Intensities of genes/ESTs differentially regulated only in males with a fold change of ≥ ± 1.5 for either of the comparisons Control vs MSG, Control vs TFA, Control vs TFA+MSG, or TFA vs TFA+MSG.

Name	Symbol	Accesssion #	Control	MSG	TFA	TFA+ MSG
NADH dehydrogenase (ubiquinone) 1, subcomplex unknown, 1, 6kDa	Ndufc1	NM_025523	1732.1	2223.5	2994.3	2945.6
protein disulfide isomerase associated 4	Pdia4	NM_009787	642.5	487.9	1080.0	976.5
magnesium transporter 1	Magt1	NM_025952	307.5	413.2	636.9	642.8
5-methyltetrahydrofolate-homocysteine methyltransferase	Mtr	NM_001081128	605.8	679.9	1239.4	843.5
thioredoxin 1	Txn1	NM_011660	1073.9	957.9	1333.4	1688.9
histone deacetylase 2	Hdac2	NM_008229	1278.2	1312.0	1955.7	1173.9
stearoyl-coenzyme A desaturase 4	Scd4	NM_183216	634.8	953.6	603.4	991.8
asporin	Aspn	NM_025711	315.0	294.1	565.4	407.4
plastin 3 (T-isoform)	Pls3	NM_145629	963.0	1138.3	2244.4	1129.1
cullin 5	Cul5	NM_027807	1102.6	1513.7	1916.8	1492.7
tropomyosin 4	Tpm4	NM_001001491	1925.4	1641.2	2560.5	2889.4
DnaJ (Hsp40) homolog, subfamily A, member1	Dnaja1	NM_008298	132.2	55.2	303.3	200.2
epithelial membrane protein 1	Emp1	NM_010128	211.9	137.8	306.0	390.7
general transcription factor II H, polypeptide 2	Gtf2h2	NM_022011	172.1	331.5	305.9	204.1
glia maturation factor, beta	Gmfb	NM_022023	281.3	297.5	562.9	507.4
topoisomerase (DNA) II binding protein 1	Topbp1	NM_176979	261.1	220.0	437.7	202.0
dystrophin, muscular dystrophy	Dmd	NM_007868	3883.0	4230.5	4157.2	2621.6
dynein light chain Tctex-type 3	Dynlt3	NM_025975	380.7	464.6	617.6	635.8
microtubule-associated protein, RP/EB family, member 1	Mapre1	NM_007896	529.0	654.4	869.1	752.5
proteoglycan 4 (megakaryocyte stimulating factor)	Prg4	NM_021400	647.8	538.9	819.1	992.4
phospholipase B domain containing 1	Plbd1	NM_025806	424.0	524.3	667.7	737.4
enhancer of zeste homolog 1 (Drosophila)	Ezh1	NM_007970	1729.0	1695.6	1407.5	866.7
THO complex 2	Thoc2	NM_001033422	728.6	937.6	1163.0	1038.6
protein phosphatase 3 (formerly 2B), regulatory subunit B, alpha isoform	Ppp3r1	NM_024459	609.1	988.4	914.1	1032.6
predicted gene 14391	Gm14391	NM_001099308	766.5	1278.5	1627.5	1517.8
protein tyrosine phosphatase 4a2	Ptp4a2	NM_008974	1547.7	1631.5	2389.4	1950.8
basic leucine zipper & W2 domains 2	Bzw2	NM_025840	1366.8	2111.4	1917.4	1964.7
vacuolar protein sorting 13A (yeast)	Vps13a	NM_173028	879.7	994.2	1390.8	952.8
COP9 constitutive photomorphogenic homolog subunit 3 (Arabidopsis)	Cops3	NM_011991	985.7	1516.3	1277.4	1481.0
chemokine (C-C motif) lig& 9	Ccl9	NM_011338	1157.1	1025.5	1178.2	1831.8
Bmi1 polycomb ring finger oncogene	Bmi1	NM_007552	566.8	921.4	997.8	1053.3
Meis homeobox 2	Meis2	NM_001136072	892.6	901.1	987.8	646.0
cornichon homolog 4 (Drosophila)	Cnih4	NM_030131	510.6	696.1	834.4	849.1
RNA binding motif protein 28	Rbm28	NM_133925	703.8	751.8	820.8	517.0
related RAS viral (r-ras) oncogene homolog 2	Rras2	NM_025846	522.3	817.8	818.7	838.5
non-SMC element 1 homolog (S. cerevisiae)	Nsmce1	NM_026330	513.1	779.4	815.6	803.3
inositol polyphosphate-4-phosphatase, type I	Inpp4a	NM_030266	553.9	545.5	790.6	518.4
family with sequence similarity 96, member A	Fam96a	NM_026635	556.4	626.2	786.8	863.4
LAS1-like (S. cerevisiae)	Las1l	NM_152822	506.3	694.6	761.3	727.5
amylase 1, salivary	Amy1	NM_007446	305.5	480.1	697.8	571.3
F-box protein 30	Fbxo30	NM_027968	435.4	625.2	676.4	589.3
poly (ADP-ribose) polymerase family, member 14	Parp14	NM_001039530	406.8	519.5	647.7	528.7
aspartylglucosaminidase	Aga	NM_001005847	385.1	410.4	606.1	551.1
NEDD4 binding protein 2	N4bp2	NM_001024917	355.1	389.1	586.8	371.3
RIKEN cDNA 2310001A20 gene	2310001A20Rik	AJ310638	381.3	478.2	576.0	647.6

Additional Table 9. Intensities of genes/ESTs differentially regulated only in males with a fold change of ≥ ± 1.5 for either of the comparisons Control vs MSG, Control vs TFA, Control vs TFA+MSG, or TFA vs TFA+MSG.

Name	Symbol	Accesssion #	Control	MSG	TFA	TFA+ MSG
ER lipid raft associated 1	Erlin1	NM_145502	316.0	410.2	574.7	476.0
TIP41, TOR signalling pathway regulator-like (S. cerevisiae)	Tiprl	NM_145513	326.7	351.9	549.8	540.1
F-box protein 41	Fbxo41	NM_001001160	466.2	711.7	549.8	431.9
zinc finger protein 97	Zfp97	NM_011765	225.8	346.3	546.3	338.8
chromatin modifying protein 5	Chmp5	NM_029814	246.1	339.6	522.3	468.8
proteasome (prosome, macropain) 26S subunit, non-ATPase, 14	Psmd14	NM_021526	347.6	477.1	514.6	608.7
versican	Vcan	NM_001081249	358.7	300.4	507.2	554.8
uroporphyrinogen decarboxylase	Urod	NM_009478	315.4	471.0	504.6	500.4
CCR4-NOT transcription complex, subunit 8	Cnot8	NM_026949	294.5	375.6	493.5	421.8
phosphoglycerate mutase 1	Pgam1	NM_023418	370.9	442.8	455.0	640.9
PH domain & leucine rich repeat protein phosphatase 2	Fxyd6	NM_022004	359.9	302.4	449.2	572.0
reticulocalbin 1	Rcn1	NM_009037	322.6	353.7	448.3	568.7
BRCA2 & CDKN1A interacting protein	Bccip	NM_025392	325.8	565.6	435.9	504.7
transmembrane protein 69	Tmem69	NM_177670	241.3	397.2	435.1	479.0
solute carrier family 35, member B1	Slc35b1	NM_016752	315.5	425.8	428.5	505.3
Rap guanine nucleotide exchange factor (GEF) 6	Rapgef6	NM_175258	379.3	441.6	422.6	587.7
inhibitor of growth family, member 4	Ing4	NM_133345	188.8	336.9	408.3	369.3
Family member 76b	Fam76b	BC043120	242.6	314.7	405.9	304.9
histone cluster 1, H1e	Hist1h1e	NM_015787	546.2	333.0	405.5	266.0
YY1 associated factor 2	Yaf2	NM_024189	272.4	352.3	391.6	426.9
TATA box binding protein (Tbp)-associated factor, RNA polymerase I, A	Taf1a	NM_021466	214.9	305.4	386.5	336.0
MAPKinase3 member 15	Map3k15	ENSMUST00000033665	260.4	199.8	375.6	108.7
enhancer of yellow 2 homolog (Drosophila)	Eny2	NM_175009	220.1	301.9	374.3	273.5
ribosomal protein S11	Gm6394	XM_915925	238.3	235.8	369.4	267.7
ethanol induced 1	Etohi1	ENSMUST00000098999	128.3	241.3	367.8	286.4
general transcription factor IIB	Gtf2b	NM_145546	120.2	185.7	362.4	211.6
recombination signal binding protein for immunoglobulin kappa J region-like	Rbpjl	NM_009036	365.9	584.7	345.6	339.8
block of proliferation 1	Bop1	NM_013481	378.0	568.3	340.2	468.0
coenzyme Q10 homolog B (S. cerevisiae)	Coq10b	NM_001039710	242.4	317.7	338.0	388.0
cysteine-rich with EGF-like domains 2	Creld2	NM_029720	257.1	195.4	334.0	397.9
integral membrane protein 2A	Itm2a	NM_008409	144.6	92.0	302.0	234.5
mitochondrial ribosomal protein L35	Mrpl35	NM_025430	233.6	272.7	298.6	352.2
guanine nucleotide binding protein (G protein), beta 4	Gnb4	NM_013531	196.1	197.8	296.2	283.4
dicarbonyl L-xylulose reductase	Dcxr	NM_026428	312.2	480.4	272.0	371.2
tetraspanin 6	Tspan6	NM_019656	145.8	145.9	270.0	277.2
reticulocalbin 3, EF-h& calcium binding domain	Rcn3	NM_026555	173.4	153.7	268.0	287.6
glutathione S-transferase, mu 4	Gstm4	NM_026764	144.9	293.6	260.0	256.9
mediator of RNA polymerase II transcription, subunit 31	Med31	NM_026068	150.8	148.7	260.0	249.2
mitochondrial ribosomal protein S14	Mrps14	NM_025474	164.7	155.9	257.3	286.2
cartilage oligomeric matrix protein	Comp	NM_016685	140.1	87.5	244.6	304.2
keratin 75	Krt75	NM_133357	148.1	123.0	234.1	193.7
transmembrane protein 106A	Tmem106a	NM_144830	184.3	241.6	226.4	341.5
tachykinin receptor 3	Tacr3	NM_021382	91.6	94.7	217.9	87.1
phosphodiesterase 3B, cGMP-inhibited	Pde3b	NM_011055	144.2	117.5	205.5	249.8
hairy & enhancer of split 2 (Drosophila)	Hes2	NM_008236	91.3	121.2	203.6	169.6
eukaryotic translation initiation factor 2 alpha kinase 4	Eif2ak4	AF193344	105.7	255.7	200.0	168.9

Additional Table 9. Intensities of genes/ESTs differentially regulated only in males with a fold change of ≥ ± 1.5 for either of the comparisons Control vs MSG, Control vs TFA, Control vs TFA+MSG, or TFA vs TFA+MSG.

Name	Symbol	Accesssion #	Control	MSG	TFA	TFA+ MSG
exosome component 8	Exosc8	NM_027148	120.5	141.0	198.6	216.0
ribosomal protein S27-like	Rps27l	NM_026467	169.1	131.0	178.7	255.2
proteasome (prosome, macropain) 26S subunit, non-ATPase, 9	Psmd9	NM_026000	160.9	210.0	168.6	290.7
phosphodiesterase 6A, cGMP-specific, rod, alpha	Pde6a	NM_146086	321.3	199.6	166.0	99.1
LSM6 homolog, U6 small nuclear RNA associated (S. cerevisiae)	Lsm6	NM_030145	109.1	101.2	164.9	142.5
sine oculis-related homeobox 4 homolog (Drosophila)	Six4	NM_011382	84.2	106.0	141.6	119.5
hyperpolarization-activated, cyclic nucleotide-gated K+ 1	Hcn1	NM_010408	76.7	29.0	135.1	138.9
asparagine-linked glycosylation 6 homolog (yeast, alpha-1,3,-glucosyltransferase)	Alg6	NM_001081264	62.3	68.4	128.4	101.2
secretogranin V	Scg5	NM_009162	75.7	112.6	121.5	90.5
activity regulated cytoskeletal-associated protein	Arc	NM_018790	79.9	102.5	120.2	138.2
histone cluster 1, H2ak	Hist1h2ak	NM_178183	77.0	83.6	119.9	116.0
Unknown	OTTMUSG00000016609	NM_001100416	130.9	225.0	384.0	284.9
Unknown	Gm14305	NM_001099327	130.9	225.0	384.0	284.9
Unknown	Gm14403	ENSMUST00000108947	203.4	355.3	442.7	309.1
Unknown	Gm10828	ENSMUST00000100068	212.3	382.6	478.5	403.2
Unknown	Gm14325	ENSMUST00000108939	217.9	456.5	654.1	518.9
Unknown	3100002L24Rik	U62393	239.0	399.3	701.3	520.4
Unknown	2410015M20Rik	BC056164	567.3	638.8	895.2	862.4
Unknown	Gm5532	ENSMUST00000097531	552.9	787.4	857.4	914.0
Unknown	Gm14434	NM_001101804	766.5	1278.5	1627.5	1517.8
