# Supplementary material for: Sex-dimorphism in Cardiac Nutrigenomics: effect of Trans fat and/or Monosodium Glutamate consumption
Source: BMC Genomics. 2011 Nov 12;12:555. doi: 10.1186/1471-2164-12-555 (PMC3238303; doi:10.1186/1471-2164-12-555)
Supplement: Additional file 10 — Table S10. Intensities of genes/ESTs differentially regulated only in females with a fold change of ≥ ± 1.5 for either of the comparisons Control vs MSG, Control vs TFA, or TFA vs TFA+MSG. [file 1471-2164-12-555-S10.PDF]

Additional Table 10. Intensities of genes/ESTs differentially regulated only in females with a fold change of ≥ ± 1.5 for either of the comparisons Control vs MSG, Control vs TFA, Control vs TFA+MSG, or TFA vs TFA+MSG.

Name	Symbol	Accesssion #	Control	MSG	TFA	TFA+ MSG
acyl-CoA synthetase family member 2	Acsf2	NM_153807	344.4	379.2	560.1	515.5
2-4-dienoyl-Coenzyme A reductase 2, peroxisomal	Decr2	NM_011933	282.6	299.9	471.4	408.6
salt inducible kinase 1	Sik1	NM_010831	875.9	744.1	1135.5	1531.0
myosin VIIA	Myo7a	NM_008663	187.2	370.9	286.2	563.4
heat-responsive protein 12	Hrsp12	NM_008287	163.1	226.7	278.5	340.1
hydroxy-delta-5-steroid dehydrogenase, 3 beta- & steroid delta-isomerase 7	Nudt2	NM_025539	541.7	841.4	649.2	372.0
Rap guanine nucleotide exchange factor (GEF) 3	Rapgef3	NM_144850	428.2	600.7	658.2	576.8
pleckstrin homology domain containing, family H	Plekhh1	AK122464	525.6	977.1	978.1	454.5
lectin, galactose binding, soluble 4	Lgals4	NM_010706	748.6	1329.0	1289.2	963.6
bromodomain & WD repeat domain containing 1	Brwd1	NM_145125	460.7	556.9	760.1	536.6
potassium voltage-gated channel, subfamily H , member 1	Kcnh1	NM_010600	146.4	203.1	166.9	257.7
hemicentin 2	Hmcn2	ENSMUST00000074390	93.6	69.8	163.3	180.1
dual oxidase 2	Duox2	NM_177610	164.3	246.9	140.5	121.5
uridine-cytidine kinase 2	Uck2	NM_030724	343.8	306.5	372.5	526.9
proteasome (prosome, macropain) 26S subunit, ATPase 2	Psmc2	NM_011188	529.7	376.1	364.0	865.8
zinc finger protein 292	Zfp292	NM_013889	524.4	550.8	632.0	420.3
guanylate cyclase 1, soluble, alpha 2	Gucy1a2	NM_001033322	377.4	478.1	583.5	411.9
GRB10 interacting GYF protein 1	Gigyf1	NM_031408	301.8	656.8	545.7	508.5
RecQ protein-like	Recql	NM_023042	340.4	531.6	506.5	380.1
RIKEN cDNA 6720456H20 gene	6720456H20Rik	NM_172600	290.7	467.6	451.6	360.8
DEAD (Asp-Glu-Ala-Asp) box polypeptide 19b	Ddx19b	NM_172284	250.8	492.1	384.0	253.1
potassium voltage-gated channel, shaker-related subfamily, member 5	Kcna5	NM_145983	251.1	353.0	377.3	379.2
kin of IRRE like 3 (Drosophila).	Kirrel3	BC063072	265.2	436.8	372.8	355.1
ArfGAP with RhoGAP domain, ankyrin repeat & PH domain 3	Arap3	NM_139206	259.7	411.0	363.8	370.1
SCO-spondin	Sspo	AJ491857	253.8	425.8	358.7	279.6
guanylate cyclase activator 1a (retina)	Guca1a	NM_008189	344.1	455.0	348.4	222.1
interleukin 18 receptor accessory protein	Il18rap	NM_010553	234.2	369.3	328.9	187.0
engulfment & cell motility 3, ced-12 homolog (C. elegans)	Elmo3	NM_172760	193.8	355.2	284.8	243.3
bridging integrator 3	Bin3	NM_021328	254.4	261.7	282.8	428.9
axin 1	Axin1	NM_001159598	194.5	171.5	272.8	175.0
autophagy/beclin 1 regulator 1	Ambra1	NM_172669	210.4	315.9	272.4	292.8
serine/threonine kinase 35	Stk35	NM_183262	239.8	288.1	266.5	373.7
RIKEN cDNA 2300009A05 gene	2300009A05Rik	NM_027090	244.0	214.3	265.6	381.5
elastase 2, neutrophil	Ela2	NM_015779	193.2	401.4	260.0	261.1
serine dehydratase	Sds	NM_145565	152.7	273.4	259.7	132.8
similar to Glyceraldehyde-3-phosphate	LOC633944	XR_032386	206.3	293.9	253.9	313.6
WNK lysine deficient protein kinase 3	Wnk3	ENSMUST00000096285	223.4	410.2	247.9	377.6
RIKEN cDNA A630033E08 gene	A630033E08Rik	NM_001110254	212.3	336.4	238.4	239.8
IQ motif containing H	Iqch	NM_030068	184.2	170.1	201.3	133.9
Yy2 transcription factor	Yy2	NM_001098723	132.3	204.3	200.7	135.3
phospholipase A2, group XV	Pla2g15	NM_133792	135.9	228.1	199.3	102.0
protocadherin 8	Pcdh8	NM_021543	125.2	201.3	185.7	152.0
mevalonate kinase	Mvk	NM_023556	136.9	249.3	156.5	105.1
transmembrane channel-like gene family 6	Tmc6	NM_145439	172.2	134.0	140.4	282.3

Additional Table 10. Intensities of genes/ESTs differentially regulated only in females with a fold change of ≥ ± 1.5 for either of the comparisons Control vs MSG, Control vs TFA, Control vs TFA+MSG, or TFA vs TFA+MSG.

Name	Symbol	Accesssion #	Control	MSG	TFA	TFA+ MSG
a disintegrin & metallopeptidase domain 5	Adam5	NM_007401	133.0	201.6	138.8	74.6
absent in melanoma 1	Aim1	NM_172393	103.7	166.1	133.7	140.1
galanin-like peptide	Galp	NM_178028	87.2	166.0	123.2	86.1
glucose-fructose oxidoreductase domain containing 2	Gfod2	NM_027469	62.1	136.7	122.1	62.1
DEP domain containing 1B	Depdc1b	NM_178683	104.2	104.7	113.5	166.8
membrane-spanning 4-domains, subfamily A, member 2	Ms4a2	NM_013516	58.4	223.6	111.6	38.7
neuronal pentraxin 1	Nptx1	NM_008730	106.6	145.7	110.6	173.2
INO80 complex subunit E	Ino80e	NM_153580	85.0	68.6	110.5	128.7
nuclear transcription factor-Y alpha	Nfya	NM_001110832	115.8	108.6	109.0	189.0
cDNA sequence BC019943	BC019943	NM_001112729	101.5	99.5	107.8	227.8
intermediate filament family orphan 2	Iffo2	NM_183148	52.1	123.0	99.3	72.1
LIM-domain containing, protein kinase	Limk1	NM_010717	93.4	49.3	95.7	199.4
centrosomal protein 76	Cep76	NM_001081073	95.3	83.9	94.4	150.4
nuclear receptor subfamily 4, group A, member 2	Nr4a2	NM_001139509	86.3	105.2	92.3	134.3
RIKEN cDNA 2610018G03 gene	2610018G03Rik	NM_133729	87.7	262.7	85.4	48.1
PH domain & leucine rich repeat protein phosphatase 2	Phlpp2	NM_001122594	85.7	56.6	79.0	131.5
predicted gene 6614	Gm6614	NM_001081318	54.9	128.6	78.9	52.3
per-hexamer repeat gene 4	Phxr4	X12806	62.8	85.9	70.8	46.3
integrin alpha 2b	Itga2b	NM_010575	108.6	188.7	58.2	57.0
T-cell acute lymphocytic leukemia 2	Tal2	NM_009317	30.2	15.3	33.1	272.6
