# Supplementary material for: Sex-dimorphism in Cardiac Nutrigenomics: effect of Trans fat and/or Monosodium Glutamate consumption
Source: BMC Genomics. 2011 Nov 12;12:555. doi: 10.1186/1471-2164-12-555 (PMC3238303; doi:10.1186/1471-2164-12-555)
Supplement: Additional File 11 — Table S11. Primers for genes selected for confirmatory QRT-PCRs. [file 1471-2164-12-555-S11.PDF]

Additional Table 11. PCR primers for qRT-PCR

Gene name	Gene Symbol	Accession number	Forward primer	Reverse primer
3-hydroxy-3-methylglutaryl-Coenzyme A synthase 2	Hmgcs2	NM_008256	5' GGCTGTCAAAACAGTGCTCA 3'	5' GCAATGTCACCACAGACCAC 3'
fructose bisphosphatase 2	Fbp2	NM_007994	5' CAGAAGCCCCTTTGAGACAG 3'	5' CAGCACGTCCAGTTTCTTCA 3'
cytochrome P450, family 2, subfamily e, polypeptide 1	Cyp2e1	NM_021282	5' GATATGGGCTCCTGATTCTC 3'	5' TCTCGGGTTGCTTCGT 3'
myosin, heavy polypeptide 7, cardiac muscle, beta	Myh7	NM_080728	5' CTTCCAGAAGCCTCGAAATG 3'	5' CTTTCTTTGCCTTGCCTTTG 3'
complement factor D (adipsin)	Cfd	NM_013459	5' GCTCTGCGCGTGGCTGGTT 3'	5' CCCTGCGGTTGCTCTCTG 3'
histone cluster 1, H1c	Hist1h1c	NM_015786	5' CGGCTCCTTCAAACTCAACA 3'	5' CTTGGGCTTGGTGACCTTC 3'
insulin-like growth factor binding protein 6	Igfbp6	NM_008344	5' GATTCCCAGGAACAAAGCAA 3'	5' CCTTCCAGAGAGTCCAGTGC 3'
cell death-inducing DFFA-like effector c	Cidec	NM_178373	5' AGCTAGCCCTTTCCCAGAAG 3'	5' CCTTGTAGCAGTGCAGGTCA 3'
insulin induced gene 1	Insig1	NM_153526	5' GTGGAGCTTGCAATCTGTGA 3'	5' CTTCTCCGGAATAGCTCGTG 3'
nudix (nucleoside diphosphate linked moiety X)-type motif 4	Loc100048122	NM_027722	5' AGCCTGTCCACGCAGAGTAT 3'	5' CAGCAGCAGTCAGGTGTGTT 3'
bone morphogenetic protein 10	Bmp10	NM_009756	5' ACTGCTGCAGAGCATGAAGA 3'	5' GAGAGGATATTTCCGGAGCC 3'
carbonic anhydrase 5b, mitochondrial	Car5b	NM_181315	5' AATGGCTTGGCTGTGATAGG 3'	5' GGTCACGGTCAACCTCAACT 3'
pyruvate dehydrogenase kinase, isoenzyme 2	Pdk2	NM_133667	5' CTGGACCGCTTCTACCTCAG 3'	5' GTTGGTGGCATTGACTTCCT 3'
plexin A2	Plxna2	NM_008882	5' GTCTGTGGCCGAGAAGATGC 3'	5' CGATCTGCTGCCGGATGAG 3'
lumican	Lum	NM_008524	5' AAGAGTGTGCCAATGGTTCC 3'	5' GGACTCGGTCAGGTTGTTGT 3'
plastin 3 (T-isoform)	Pls3	NM_145629	5' AATGAAGCACTGGCAGCTTT 3'	5' GTGCGATTTGATTGAGCAGA 3'
tuberous sclerosis 2	Tsc2	NM_011647	5' CGGAACTCAAGACCATCGT 3'	5' GGACTGGGCTCTGTAGGATA 3'
protein disulfide isomerase associated 4	Pdia4	NM_009787	5' ATCGCCAAGATGGATGCTAC 3'	5' CTTGGTCCTGCTCCTCTTTG 3'
